# Thiazolyl-Methylthio-1,3,4-Thiadiazole Hybrids as Halicin Analogues with Antimicrobial and Antibiofilm Activities: Chemical Development, Biological Assessment, and 2D-QSAR Study

**DOI:** 10.3390/antibiotics15050448

**Published:** 2026-04-29

**Authors:** Daniel Ungureanu, Gabriel Marc, Mihaela Niculina Duma, Dan Cristian Vodnar, Gheorghe-Adrian Martău, Laurian Vlase, Adrian Pîrnău, Brîndușa Tiperciuc, Cristina Moldovan, Ioana Ionuț, Anca Stana, Ilioara Oniga, Ovidiu Oniga

**Affiliations:** 1 Department of Pharmaceutical Chemistry, “Iuliu Hațieganu” University of Medicine and Pharmacy, 41 Victor Babeș Street, 400012 Cluj-Napoca, Romania; daniel.ungureanu@elearn.umfcluj.ro (D.U.); btiperciuc@umfcluj.ro (B.T.); cmoldovan@umfcluj.ro (C.M.); ionut.ioana@umfcluj.ro (I.I.); stana.anca@umfcluj.ro (A.S.); ooniga@umfcluj.ro (O.O.); 2 Department of Organic Chemistry, “Iuliu Hațieganu” University of Medicine and Pharmacy, 41 Victor Babeș Street, 400012 Cluj-Napoca, Romania; 3 State Veterinary Laboratory for Animal Health and Safety, 1 Piața Mărăști Street, 400609 Cluj-Napoca, Romania; duma.mihaela-cj@ansvsa.ro; 4 Institute of Life Sciences, University of Agricultural Sciences and Veterinary Medicine, 3-5 Calea Mănăștur, 400372 Cluj-Napoca, Romania; dan.vodnar@usamvcluj.ro (D.C.V.); adrian.martau@usamvcluj.ro (G.-A.M.); 5 Faculty of Food Science and Technology, University of Agricultural Sciences and Veterinary Medicine, 3-5 Calea Mănăștur, 400372 Cluj-Napoca, Romania; 6 Department of Pharmaceutical Technology and Biopharmacy, “Iuliu Hațieganu” University of Medicine and Pharmacy, 41 Victor Babeș Street, 400012 Cluj-Napoca, Romania; laurian.vlase@umfcluj.ro; 7 National Institute for Research and Development of Isotopic and Molecular Technologies, 67-103 Donath Street, 400293 Cluj-Napoca, Romania; apirnau@itim-cj.ro; 8 Department of Pharmacognosy, “Iuliu Hațieganu” University of Medicine and Pharmacy, 12 Ion Creangă Street, 400010 Cluj-Napoca, Romania; ioniga@umfcluj.ro

**Keywords:** thiazole, thiadiazole, coumarin, hybrid compounds, antimicrobial, antibiofilm, Free-Wilson

## Abstract

**Background/Objectives**: The purpose of this study was the chemical design, synthesis, and evaluation of the antimicrobial and antibiofilm potentials of 20 novel thiazolyl-methylthio-thiadiazole hybrid compounds (**6a**–**j** and **8a**–**j**). **Methods**: The compounds were designed as structural analogues of halicin with two points of variation and were synthesized through a process with multiple condensation steps. The compounds were evaluated in vitro through MIC determinations for the antimicrobial activity and percentage of biofilm inhibition, and in silico, respectively, through molecular docking, druggability, and ADMETox prediction. A 2D-QSAR study was conducted for antimicrobial activity using the Free-Wilson model. **Results**: In terms of antibacterial activity, all compounds displayed important activity on the tested strains (MICs = 15.62–250 μg/mL), except against *Staphylococcus aureus*. Regarding the antifungal activity, the effect against *Candida albicans* was similar to fluconazole in most cases (MIC = 15.62 μg/mL). With respect to the antibiofilm activity, the most effective activity was registered against the *Pseudomonas aeruginosa* biofilm. The in vitro results for the antibacterial activity against *Escherichia coli* were correlated with the observations drawn in the molecular docking study on the ATPase domain of the GyrB subunit of *E. coli*. The in silico predictions of the molecular properties concluded that all compounds have good druggability properties, while the ADMETox predictions concluded that the compounds could have low gastrointestinal absorption and blood–brain barrier permeation capacity, but raised safety flags (e.g., hepatotoxicity and high acute oral toxicity). The 2D-QSAR study concluded that the thiazolyl-methylthio-thiadiazole scaffold had the highest contribution to antimicrobial activity in almost all cases. **Conclusions**: The two series of compounds highlight the impact of structural modulations of the scaffold and its substituents on the investigated biological activities.

## 1. Introduction

The antimicrobial resistance (AMR) represents an emerging threat against global public health and approximately 1.27 million deaths per year were caused by AMR [1]. At this rate, it is estimated that by 2050 there could be at least 10 million deaths per year, if no effective solution is brought up soon. Therefore, AMR has been regarded as a “silent” pandemic [1,2].

**Figure 1:**
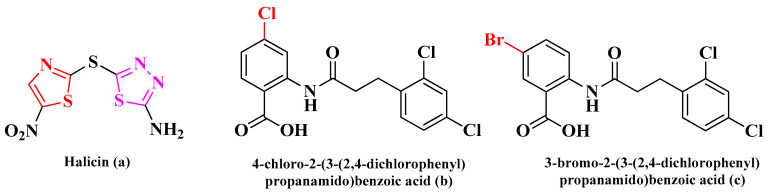
Examples of compounds discovered through deep learning approaches using AI.

Besides the traditional strategies applied in medicinal chemistry for AMR, such as the total synthesis of new compounds, the optimization of already existing drugs, or the targeting of various virulence factors, a new powerful tool has emerged and that is artificial intelligence (AI) [3,4]. AI has the advantage of rapid virtual screening of thousands of compounds from various databases and identifies only a few compounds that are the most promising candidates for the development of novel antimicrobials or other drugs, depending on the case. Among the most notable examples of antimicrobials discovered through deep learning approaches are halicin (**a**), a repurposed antidiabetic as antibacterial, and two propanamido-benzoic acid derivatives (**b** and **c**) as potent antibacterials against methicillin-resistant *Staphylococcus aureus* (MRSA) and vancomycin-resistant enterococci. In both cases, the compounds were selected from large compound libraries (Figure 1) [5,6].

Halicin (**a**) is a siderophore with broad antibacterial activity against a large variety of bacterial strains, including *S. aureus* ATCC BA A-977, *Escherichia coli* ATCC 25922, *Acinetobacter baumannii* ATCC BAA-747, ATCC 19606, and MDR 3086, *Pseudomonas aeruginosa* ATCC 27853, or MRSA ATCC 43300 and BNCC 337371 [4,7,8]. Structurally, it is a hybrid compound containing a 5-nitrothiazole and a 2-amino-1,3,4-thiadiazole moiety, linked through a sulfur bond.

Besides the significant antimicrobial activity, there are several reports in the literature that have also evaluated the antibiofilm potential of halicin. It showed antibiofilm activity against mature and immature *S. aureus* biofilms, along with potent activity against some biofilms of antimicrobial resistant bacterial strains like *A. baumannii* and *E. coli*. Moreover, halicin maintained its antibiofilm activity on orthopedically relevant biofilm surfaces such as muscles, bones, alloys, and polymers [9,10,11].

Taking into consideration the broad spectrum of a structurally simple molecule, the aim of this paper was to design two novel series of antimicrobial and antibiofilm drugs as analogues of halicin. The differences between the two scaffolds are the linking positions of the thiazole to the thiadiazole ring and the substituents grafted on both heterocycles. In halicin, the heterocycles are linked by a 2-5′ sulfur bridge and the thiazole heterocycle is 5-nitro substituted, while the thiadiazole is 2′-amino substituted.

**Figure 2:**
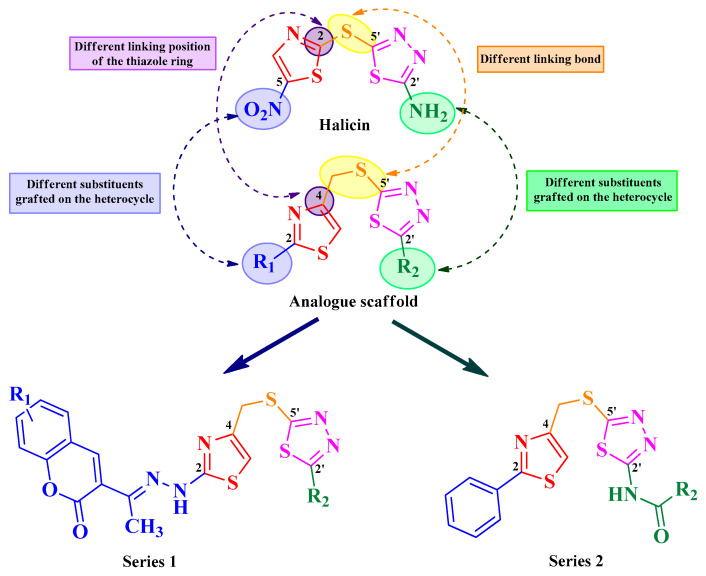
The development of series 1 and 2 of compounds starting from halicin.

Our main scaffold is represented by a thiazole linked to the thiadiazole heterocycle by a 4-5′-methylene-thio linker. Notable antimicrobials that contain a thiazole heterocycle linked in position 4 to the rest of the structure include aztreonam, several cephalosporins (ceftriaxone, cefixime, cefditoren, ceftazidime, cefdinir, cefiderocol), and several antifungals (abafungin, isavuconazole, and ravuconazole) [12]. The introduction of a coumarin heterocycle in the first series of compounds brings additional value due to its antimicrobial properties [13,14]. We kept the position 2 from thiazole and position 2′ from thiadiazole as substituting positions. Therefore, two series were developed: series 1 as coumarinyl-thiazolyl-methylthio-thiadiazoles and series 2 as phenyl-thiazolyl-methylthio-thiadiazoles (Figure 2).

Herein, we present the design, chemical synthesis, in silico and in vitro evaluation, and QSAR of two series of thiazolyl-methylthio-1,3,4-thiadiazole hybrid compounds. In silico evaluations of these compounds included druggability, ADMETox predictions, and molecular docking. The antimicrobial potential was evaluated against Gram-positive and Gram-negative bacterial strains, Candida, and Aspergillus strains. The antibiofilm potential was tested against several bacterial biofilms. A quantitative structure–relationship (QSAR) study using the 2D-QSAR Free-Wilson model was performed for the antimicrobial activity. In the end, the results were compared to halicin, to see if the scaffold had a contribution to the activity or it was mostly influenced by the grafted substituents.

## 2. Results

### 2.1. Chemical Synthesis

**Figure 3:**
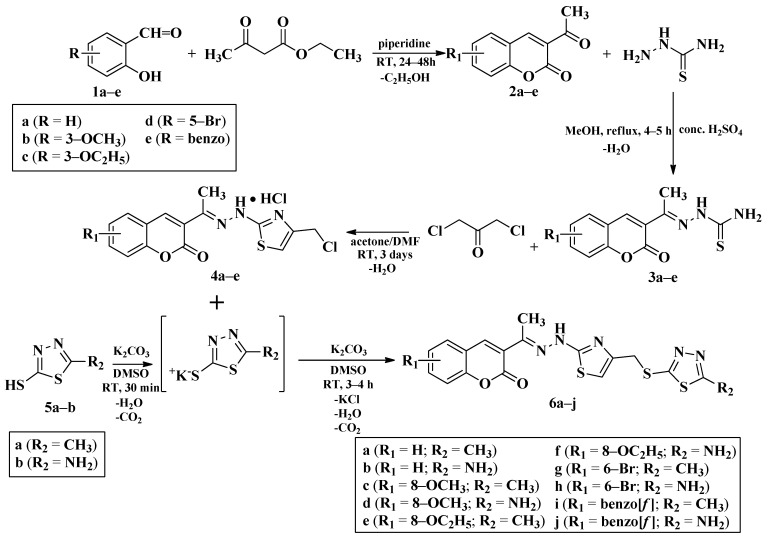
The general synthetic process for compounds **6a**–**j**. Legend: RT—room temperature; MeOH—methanol; conc.—concentrated; DMF—dimethylformamide; DMSO—dimethyl sulfoxide. R and R_1_ denote the same set of substituents, assigned to different positions.

A total of 20 final compounds, divided into two series **6a**–**j** and **8a**–**j**, were obtained through two different chemical processes. The synthesis of the first series **6a**–**j** started with the condensation of ethyl acetoacetate with salicylaldehydes **1a**–**e**, followed by further derivatization of the resulting 3-acetylcoumarins **2a**–**e** with thiosemicarbazide. The coumarin-3-yl-thiosemicarbazones **3a**–**e** were then condensed with 1,3-dichloroacetone. Finally, the newly obtained 2-(coumarin-3-yl)-hydrazono-4-chloromethylthiazole salts **4a**–**e** were condensed with the potassium salts of 5-methyl-1,3,4-thiadiazole-2-thiol (**5a**) or 5-amino-1,3,4-thiadiazole-2-thiol (**5b**), to obtain the coumarinyl-hydrazono-thiazolyl-methylthio-5-thiadiazoles (**6a**–**j**) (Figure 3).

**Figure 4:**
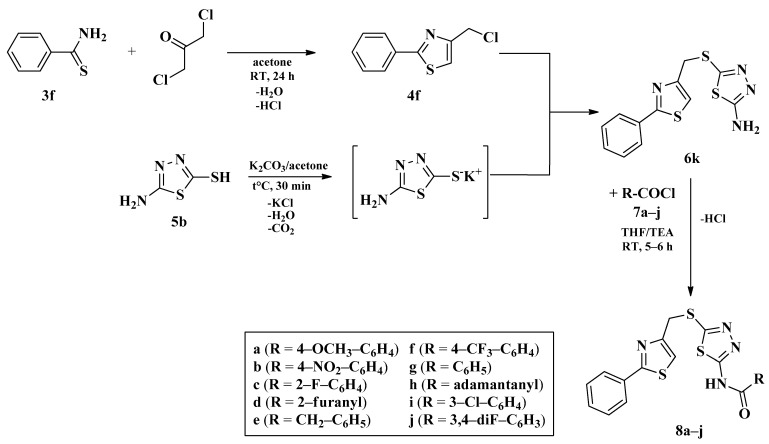
The general synthetic process for compounds **8a**–**j**. Legend: THF—tetrahydrofuran; TEA—triethylamine.

The second series (compounds **8a**–**j**) was obtained starting from the condensation of thiobenzamide **3f** with 1,3-dichloroacetone, which yielded 2-phenyl-4-chloromethylthiazole **4f**. This intermediate was next derivatized to 4-(2-phenyl)-thiazolyl-methylthio-5′-aminothiadiazole **6k**, through condensation in basic conditions with the potassium salt of **5b**. In the final step, the amino group of the thiazolyl-methylthio-thiadiazole **6k** was then acylated with various acyl chlorides (**7a**–**j**) to compounds **8a**–**j** (Figure 4).

The novel compounds were obtained in various yields (15–94%) and their structural identity was confirmed through IR, MS, ^1^H-, and ^13^C-NMR spectral analysis. The progress of reactions was monitored using thin layer chromatography (TLC) and retardation factors (R_f_).

The graphical presentations of all recorded spectra for the compounds **6a**–**j**, **8a**–**j**, and their intermediates are illustrated in Figures S1–S104 from the Supplementary Materials.

### 2.2. In Silico Evaluation

#### 2.2.1. Druggability and ADMETox Predictions

Early stages of drug design benefit from the virtual prediction of the druggability, and the pharmacokinetic and toxicological properties of potential candidates, providing a time sparing method to select and eliminate unsuitable compounds [15].

The considered physicochemical descriptors to determine the druggability of compounds **6a**–**j** and **8a**–**j** were molecular weight (MW), number of rotatable bonds (RB), number of hydrogen bond acceptors (HBA), number of hydrogen bond donors (HBD), topological surface area (TPSA), octanol–water partition coefficient implemented by Moriguchi (MLogP), estimated solubility (ESOL), and number of violations of Lipinski’s rule of five (Table 1) [16,17,18,19,20,21].

The pharmacokinetic descriptors computed for compounds **6a**–**j** and **8a**–**j** were gastrointestinal (GI) absorption, blood–brain barrier (BBB) permeation, P-glycoprotein (P-gp) substrate, and inhibition potential of several cytochrome P450 (CYP450) isoenzymes, particularly CYP1A2, CYP2C19, CYP2C9, CYP2D6, and CYP3A4 [22,23].

**Figure 5:**
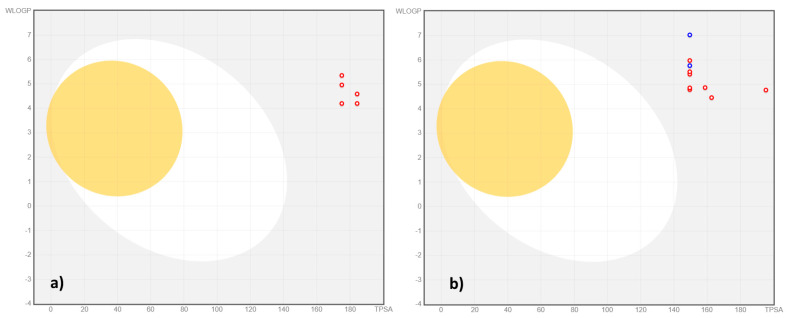
BOILED-Egg graphs for the prediction of GI absorption and BBB penetration capacity. The yolk represents BBB penetration capacity, while the egg white represents the GI absorption: (**a**) prediction for compounds **6a**–**j** (red circles—no P-gp substrates); (**b**) prediction for compounds **8a**–**j** (blue circles—P-gp substrates).

The toxicological descriptors computed for compounds **6a**–**j** and **8a**–**j** were carcinogenicity, eye and skin irritation, hepatotoxicity, respiratory toxicity, reproductive toxicity, mitochondrial toxicity, nephrotoxicity, and acute oral toxicity [24,25,26,27,28,29,30,31]. Two BOILED-Egg graphs (Figure 5) were generated to illustrate the prediction of GI absorption and BBB permeation of compounds **6a**–**j** and **8a**–**j**.

All compounds **6a**–**j** and **8a**–**j** were predicted to have low GI absorption and BBB permeation capacity (Figure 5). None of the compounds, excepting **8f** and **8h**, were predicted as a potential P-gp substrate (Figure 5). Regarding the inhibition potential of CYP_450_ isoenzymes, the computed descriptors revealed the following predictions: compounds **8c**–**e**, **8g**, **8i**, **8j** as CYP1A2 inhibitors; all compounds, excepting **6e** and **6h**, as CYP2C19 inhibitors; compounds **8a** and **8c**–**j** as CYP2D6 inhibitors; all compounds **6a**–**j** and **8a**–**j** as CYP2C9 and CYP3A4 inhibitors (Table S1 from the Supplementary Materials).

According to the computed toxicological descriptors, none of the compounds were predicted to cause skin irritation. With some exceptions, compounds **6a**–**j** and **8a**–**j** were not predicted to cause carcinogenicity (excepting **8b**), respiratory toxicity (excepting **8h**) and reproductive toxicity (excepting **8a** and **8h**). On the other hand, all compounds were predicted as hepatotoxic and with high acute oral toxicity (class III). Most of the compounds could cause eye irritation (excepting **8a**–**c**, **8e**, **8f**, and **8i**) and could be nephrotoxic (excepting **8a**, **8e**, and **8h**) (Table S2 from the Supplementary Materials).

According to the Cramer rules, the class III acute oral toxicity was attributed to the presence or absence of the following functional groups in the tested compounds: lactone fused to another ring (compounds **6a**–**f**, **6i**, and **6j**), bromine substitution (compounds **6g** and **6h**), lack of sufficient sulphonate groups (compounds **8a**, **8d**, **8e**, **8g**, and **8h**), nitro substitution (compound **8b**), fluorine substitutions (compounds **8c**, **8f**, and **8j**), and chlorine substitution (compound **8i**) [31]. Hepatotoxicity is a model-based in silico alert and cannot be assigned to a specific functional group [27,30].

#### 2.2.2. Density Functional Theory (DFT) Calculations

DFT studies are important in medicinal chemistry since they can be used to predict several electronic properties of potential drug candidates, such as conformational energies, electronic affinities, binding and ionization energies, or molecular geometrics [32,33]. Herein, to explain at electronic level the obtained protein–ligand complexes observed in the following molecular docking study, we conducted some DFT calculations, using the energies of the frontier molecular orbitals HOMO (Highest Occupied Molecular Orbital) and LUMO (Lowest Unoccupied Molecular Orbital). HOMO energies refer to the tendency of a compound to donate electrons, while LUMO energies refer to its tendency to accept electrons [34,35]. The energy levels of HOMO and LUMO in vacuum, nonpolar solvent, polar solvent, and water are presented in Table 2. The HOMO and LUMO visualizations, along with the electrostatic potential map for each of the compounds **6a**–**j** and **8a**–**j** are available in the Supplementary Materials (Tables S3 and S4).

#### 2.2.3. Molecular Docking Studies

The molecular docking study was performed on the ATPase domain of the bacterial DNA gyrase subunit B (GyrB) 24 kDa from *E. coli*. The selection of this target was motivated by the structural similarity of compounds **6a**–**j** with novobiocin, which was a potent GyrB inhibitor, both containing an aminocoumarin moiety. Moreover, there are literature reports of azole compounds that can target DNA gyrase as a purposed mechanism of action [36,37]. Additionally, the lack of authorized antibacterial GyrB inhibitors makes this target promising against bacterial strains that have developed antimicrobial resistance against common drugs, such as fluoroquinolones [38,39].

The results of the predicted binding affinity (BA) of compounds **6a**–**j** and **8a**–**j** to the ATPase domain of GyrB are presented in Table 3.

**Figure 6:**
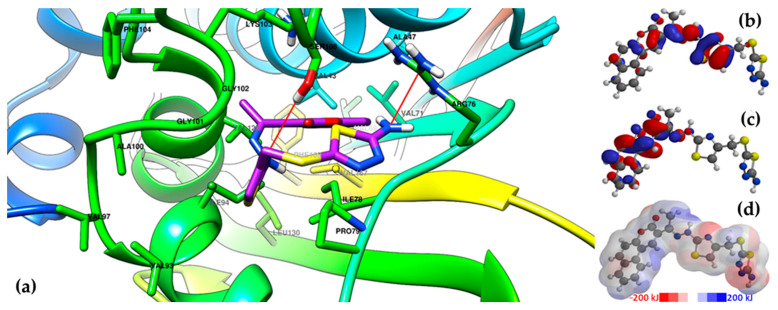
(**a**) The predicted binding pose of compound **6j** in the ATPase domain of GyrB. The sidechain of Ser108 is predicted to act as a HBD (red line) to one of the nitrogen atoms of the hydrazone linker, while the benzo[*f*]coumarin fits in a hydrophobic binding pocket comprising Val71, Val69, Val43, Phe169, Val167, and Val120. The positively charged Arg76 sidechain is predicted to be involved in a polar contact (red line) with the nitrogen atom of the 5-amine group from the thiadiazole heterocycle. The following coloring scheme was used: purple for carbon atoms, red for oxygen atoms, blue for nitrogen atoms, white for hydrogen atoms, and yellow for sulfur atoms; (**b**) graphical depiction of the localization of the HOMO frontier orbital in compound **6j**; (**c**) graphical depiction of the localization of the LUMO frontier orbital in compound **6j**; and (**d**) graphical depiction of the electrostatic potential map of compound **6j**. Red represents the electron-rich regions, while blue represents the electron-depleted regions.

**Figure 7:**
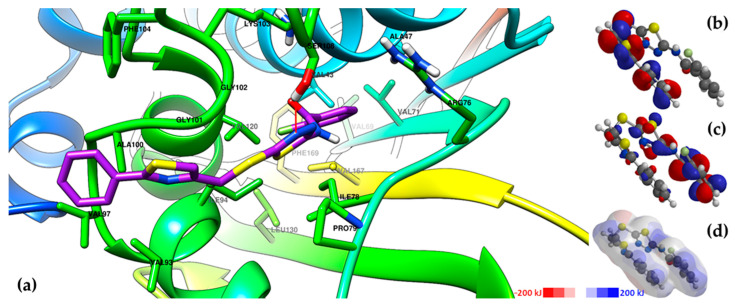
(**a**) The predicted binding pose of compound **8c** in the ATPase domain of GyrB. The sidechain of Ser108 is predicted to act as a HBD (red line) to one of the nitrogen atoms of the thiadiazole heterocycle. The phenyl-thiazole (left side) is located in a hydrophobic region comprising Val97, Val93, Ile94, Phe104, and Ala100. The following coloring scheme was used: purple for carbon atoms, red for oxygen atoms, blue for nitrogen atoms, white for hydrogen atoms, and yellow for sulfur atoms; (**b**) graphical depiction of the localization of the HOMO frontier orbital in compound **8c**; (**c**) graphical depiction of the localization of the LUMO frontier orbital in compound **8c**; and (**d**) graphical depiction of the electrostatic potential map of compound **8c**. Red represents the electron-rich regions, while blue represents the electron-depleted regions.

The graphical depiction of the interaction between compounds **6j** and **8c**, which were the compounds with the best binding affinities, and the ATPase domain of GyrB is presented in Figure 6 and Figure 7. Additional graphical depictions for the compounds **6i** and **8f**, which also registered some of the best binding affinities, are presented in Figures S105 and S106 from the Supplementary Materials.

The graphical depiction of compounds **6i**, **6j**, **8c**, and **8f** in the active site of the ATPase domain of GyrB displayed as surfaces is illustrated in Figures S107–S110 from the Supplementary Materials.

### 2.3. Antimicrobial Evaluation

Compounds **6a**–**j** and **8a**–**j** were tested for their antimicrobial activity, quantified as minimal inhibitory concentrations (MICs). The antibacterial activity was tested against Gram-negative bacterial strains, such as *E. coli* (ATCC 25922), *Salmonella enteritidis* (ATCC 13076), *S. typhimurium* (ATCC 14028), *S. typhimurium* (isolated from food source), *S. derby* (isolated from food source), and *P. aeruginosa* (ATCC 27853), and Gram-positive bacterial strains like *Enterococcus faecalis* (ATCC 29212) and *S. aureus* (ATCC 6538P), using ciprofloxacin as antibacterial reference. The antifungal activity was tested against *C. albicans* (ATCC 102321) and *A. brasiliensis* (ATCC 16404), using fluconazole as antifungal reference. The results are presented in Table 4 and Table 5.

### 2.4. Antibiofilm Activity

This assay reflects the inhibition of biofilm formation. Twelve compounds with the most notable antibacterial activity (**6b**, **6c**, **6i**, **6j**, **8a**, **8b**, and **8e**–**j**) were further tested against *E. faecalis* (ATCC 29212), *P. aeruginosa* (ATCC 27853), *E. coli* (ATCC 25922), and *S. typhimurium* (ATCC 14028) bacterial biofilms, using gentamicin as reference. The selection of these compounds was primarily based on the obtained results in the MIC assay against *P. aeruginosa* (ATCC 27853). The activity was quantified as percentage (%) of biofilm (BF) inhibition and the results are presented in Table 6.

### 2.5. 2D-QSAR Studies

#### Free-Wilson 2D-QSAR Model

QSAR is one of the oldest and most used instruments to examine the correlation between the biological activity of a compound and its molecular structure. 2D-QSAR models are based on the molecular properties derived from the whole structure of compounds. The Free-Wilson 2D-QSAR model studies the detailed relationship between the biological activity and the small changes in the compounds’ substituents [40,41]. In this model, the contribution to the biological activity of the scaffold and the substituents is quantified using Equation (1):

Log E = C_s_ + x_ij_ + y_ij_ (1)

The obtained activity was expressed as log E, where E represents the inversed value of the activity, C_s_ represents the contribution of the scaffold to the activity, x_ij_ and y_ij_ represent the sums of contributions of the substituents. The equation is obtained after applying multiple linear regression on a matrix with a number of lines equal to the number of compounds and a number of columns equal to the number of substituents. The presence of a compound is quantified with “1” and its absence with “0” (Table S5 from the Supplementary Materials). Each compound is described by an equation (for example, the equation for compound **6a** would be: Log E = C_s_ + x_1_ + y_1_).

The obtained equations in the Free-Wilson 2D-QSAR model for the antimicrobial activities against all tested strains, following the multiple linear regression analysis are presented in Table 7. Compound **6a** was identified as an outlier in the equation for the activity against *E. coli* and was eliminated to increase the goodness of fit of the equation. This is because the activity of compound **6a** against *E. coli* was markedly lower compared to the other compounds. Similar behavior was observed in the other equations, except for those describing the activity against *S. aureus*, *C. albicans*, and *A. brasiliensis*. In those cases, the differences between compound **6a** and the remaining compounds were less pronounced and therefore did not significantly affect the goodness of fit of the corresponding equations.

For the activity against *S. enteritidis*, *S. typhimurium*, and *E. faecalis*, respectively for the activity against *S. typhimurium* (food isolate) and *S. derby* (food isolate), the same equations that correlate the structure–activity relationship were obtained. The codification of the substituents is presented in Section 4.

The mathematical models were validated through the leave-one-out cross-validation (LOOCV) statistical method. The cross-validation coefficients (ΔCV, Table 7) varied between 0.0003579 and 0.1191464, thus indicating the obtained models have a good predictability.

The calculated log E (lg E) values using the obtained equations are presented in Table 8. In all cases, the p values resulting from the comparison between the two observed/calculated log E values, using the two-sample assuming equal variances *t*-test, were between 0.923979 and 0.999758. Therefore, no statistically significant differences (*p* > 0.05) were observed between the two log E values in every case.

## 3. Discussion

### 3.1. Chemical Synthesis

Intermediate compounds **2a**–**e**, **3a**–**e**, **4a**, **4f**, and **6k** were previously reported in the literature [35,42,43,44,45,46,47,48,49,50,51,52,53]. According to the Reaxys database, intermediate compounds **4b**–**e** and the final products **6a**–**j** and **8a**–**j** were not previously reported.

The 4-thiazolylchloromethyl derivatives **4a**–**e** were confirmed by extended spectral analysis. Based on the IR spectra, the intermediates had an additional band for the C-Cl in the fingerprint region, between 702 cm^−1^ and 713 cm^−1^. The intermediates were further confirmed by the MS spectra, following the identification of the corresponding molecular peaks. The corresponding signals for the proton and carbon atoms were identified in the expected regions of the ^1^H- and ^13^C-NMR spectra.

The final products **6a**–**j** were confirmed by extended spectral analysis. According to the IR spectra, the same bands were observed as for the intermediates **3a**–**e**, except for the C-Cl band. The corresponding molecular peaks were identified in the MS spectra and the corresponding signals for the proton and carbon atoms were identified in the expected regions of the ^1^H- and ^13^C-NMR spectra. The presence of the deshielded N-H proton signal at chemical shifts ranging between 11.36 and 11.46 ppm confirmed that compounds **6a**–**j** were mainly obtained in the *E* configuration, according to the literature data, which is also the most energy-favorable configuration [35,54,55].

The obtained intermediates **4f** and **6k** were previously synthesized by our research group and reconfirmed this time through IR and MS analysis [45,56]. Regarding intermediate **6k**, the presence of wide νN-H stretching band at 3414 cm^−1^ in the IR spectrum confirmed the condensation successfully. The corresponding molecular peak was identified in the MS spectra.

The final compounds **8a**–**j** were confirmed through extended spectral analysis. Based on the IR spectra, two wide νC=O stretching bands corresponding to the amide bond could be observed for all compounds in the range between 1691 cm^−1^ and 1601 cm^−1^. The corresponding molecular peaks were identified in the MS spectra and the corresponding signals for the proton and carbon atoms were identified in the expected regions of the ^1^H- and ^13^C-NMR spectra.

### 3.2. In Silico Evaluation

#### 3.2.1. Druggability and ADMETox Predictions

The molecular weight for every compound was between 400.50 g/mol and 509.42 g/mol and the number of rotatable bonds between 6 and 8. Each compound had 4–7 HBAs and 1–2 HBDs, while the value of TPSA was between 149.55 Å^2^ and 210.30 Å^2^. The computed MLogP for every compound had a positive value between 1.37 and 3.76, while the ESOL was predicted to be between 1.13 µg/mL and 8.28 µg/mL. A total of 18 out of the 20 screened compounds respected the Lipinski’s rule of five, which means that they have a molecular weight lower than 500 g/mol, a number of HBA (O and N atoms) lower than 10, a number of HBD (NH and OH groups) lower than 5, and a value of MLogP lower than 4.15. Compounds **6g** and **6h** had one violation of Lipinski’s rule of five due to their molecular weight being higher than the threshold. Overall, the presented compounds had good druggability properties [19,20].

Based on the computed pharmacokinetic descriptors, all compounds were predicted to have low GI absorption, making them suitable for the treatment of enteric infections. In terms of CYP450 isoenzymes inhibition, all compounds were predicted to inhibit CYP3A4 and CYP2C9 isoenzymes, thus representing an increased potential of drug interactions. Compounds **6a**–**c**, **6e**–**g**, **6i**–**j**, and **8a**–**j** were predicted as potential CYP2C19 inhibitors, while compounds **8c**–**e**, **8g**, and **8i**–**j** as potential CYP1A2 inhibitors. None of the **6a**–**j** compounds were predicted as CYP2D6 inhibitors, suggesting that the coumarin moiety may not have affinity for this isoenzyme. The same observation may be valid also for the CYP1A2 inhibition. In terms of affinity for the P glycoprotein, only compounds **8f** (4-trifluorophenyl substituted) and **8h** (adamantanyl substituted) were predicted as substrates. However, the reduced predicted absorption rate could significantly limit the risk of pharmacokinetic drug–drug interactions.

Regarding the toxicological properties, on one hand, most of the compounds were predicted to produce no skin irritation, carcinogenicity, and nephrotoxicity. On the other hand, they were predicted to possess hepatotoxicity and acute oral toxicity. Only compound **8b** was predicted as potential carcinogenic, while **8h** was the only compound predicted to possess respiratory toxicity. Compound **8a** and **8h** were the only compounds predicted to produce reproductive toxicity. Compounds **8a**, **8e**, and **8h** were not predicted to be nephrotoxic agents. While low predicted GI absorption may reduce systemic exposure, it does not eliminate safety concerns, notably hepatotoxicity and high acute oral toxicity (class III). Therefore, the pharmaceutical potential of these compounds should be interpreted cautiously and supported by experimental toxicological profiling. Notably, many authorized antimicrobials have recognized safety liabilities, examples including the aminoglycoside-induced nephrotoxicity and ototoxicity, azole-induced hepatotoxicity, or vancomycin-induced red man syndrome, highlighting the need for experimental validation of the in silico alerts [57,58,59,60].

#### 3.2.2. DFT Calculations

Across compounds **6a**–**j**, a consistent solvent effect was observed for both methyl- and amine-substituted thiadiazole-derived compounds. Moving from vacuum to solvated environments, it led to a modest stabilization of the HOMO, between 0.05 and 0.15 eV. This stabilization increased slightly with the solvent polarity, but it was largely saturated between polar solvent and water.

In compounds **6a**–**j** the HOMO was localized over the hydrazone-thiazole region of the compounds, while the LUMO was localized over the coumarin heterocycle (Table S3 from the Supplementary Materials).

Within each pair, amino-thiadiazole compounds (**6b**, **6d**, **6f**, **6h**, and **6j**) generally exhibited slightly lower HOMO levels compared to their methyl analogues (**6a**, **6c**, **6e**, **6g**, and **6i**), particularly when solvated. This trend reflected the stronger interaction of the polar amine group with the solvent, but the amplitude of this effect remained moderate, because the HOMO was found across the hydrazone-thiazole region (Table S3 from the Supplementary Materials). The methyl or amino substitution occurs at a site distant from the HOMO region, and the thiazole and thiadiazole rings are connected through a sp^3^-hybridized carbon atom. This linkage substantially limited electronic communication, thereby reducing the ability of the thiadiazole moiety to influence the electronic properties of the hydrazone–thiazole fragment where the HOMO was localized. On the other hand, substitution of the coumarin core with various substituents did not significantly affect the HOMO energy level, because the oxygen atoms within the coumarin structure are located much closer to the HOMO region and have contributed predominantly to its electronic character. Consequently, the electronic effects of the variable substituents from coumarin became negligible.

The LUMO energies in compounds **6a**–**j** showed a more pronounced sensitivity to both substitution and solvent polarity. For most compounds, the transition from vacuum to solvents resulted in a stabilization of approximately 0.05–0.20 eV. This effect was particularly obvious for compounds **6g**–**j**, where the LUMO was significantly lowered in polar environments. Because LUMO was found on the coumarin moiety (Table S3 from the Supplementary Materials), the variable substitution of the coumarin influenced the LUMO levels. A significant influence was identified for the alkoxy electron donating substitution with methoxy and ethoxy groups in compounds **6c**–**f**, which had higher energy when compared to the brominated derivatives **6g** and **6h**. As expected, the amino or methyl substitution of the thiadiazole heterocycle had no significant effect on LUMO levels, because the respective substitution was found on the other end of the molecules, with no possible way to directly influence the electron density on the coumarin heterocycle.

In compounds **8a**–**j** the HOMO was localized on the phenyl-thiazole region, while the LUMO was localized either on the R-substituted phenyl ring directly linked to the amide or on the phenyl-thiazole region, overlapping the HOMO, depending on the electronic nature of the substituents grafted on the R-substituted phenyl ring (Table S3 from the Supplementary Materials).

In compounds **8a**–**j**, the HOMO energies had a broader range when compared to compounds **6a**–**j**, reflecting the diverse electronic effects due to the nature of the attached functional groups. The solvent effect on the HOMO was generally modest, with typical shifts below 0.2 eV from vacuum to water. In several cases (compounds **8a** and **8h**), the HOMO energy was insensitive to the solvent polarity, indicating a limited orbital–solvent interaction. The HOMO was predominantly localized on the phenyl-thiazole fragment, spatially distant from the variable substituents (Table S3 from the Supplementary Materials). Nevertheless, the conformational folding induced by the flexible thioether linker, which acts as a molecular hinge, allowed the substituents to indirectly affect the HOMO energy levels.

Regarding the distribution of the LUMO, an interesting observation was worth making. In compounds where the substitution of the amide was made with electron donating groups (**8a**, R = 4-methoxy), furanyl (**8d**), benzyl (**8e**), adamantanyl (**8h**), the LUMO was found on the phenyl-thiazole region (Table S3 from the Supplementary Materials), while in the compounds substituted with electron withdrawing groups (**8b** − R = 4-nitro, **8c** − R = 2-fluoro, **8f** − R = 4-trifluoromethyl, **8i** − R = 3-chloro, and **8j** − R = 3,4-difluoro), it was found on the R-substituted phenyl ring directly linked to the amide bond (Table S3 from the Supplementary Materials).

Compared to compounds **6a**–**j**, the compounds **8a**–**j** showed a larger dispersion of LUMO energies, indicating that functional groups changes were a dominant factor in influencing the electron distribution across the compounds.

Taken together, these results demonstrated that solvent polarity consistently stabilized both HOMO and LUMO energies, with a stronger effect on the LUMO. Substitution with the amine group in compounds **6a**–**j** enhanced solvent sensitivity relative to methyl substitution, particularly for unoccupied orbitals. In contrast, for compounds **8a**–**j** it was shown that the functional group was the dominant factor governing frontier orbital energies, while solvent effects acted as a secondary, but still significant factor.

#### 3.2.3. Molecular Docking Studies

All compounds registered binding affinities between −9.4 and −7.7 kcal/mol (Table 3). Overall, compounds **6a**–**j** had better affinities compared to compounds **8a**–**j**. In the first series of compounds, the highest binding affinity was registered by compounds **6i** and **6j** (BA = −9.4 kcal/mol). In the second series of compounds, the highest BA was registered by compounds **8c** and **8f** (BA = −8.9 kcal/mol).

According to the graphical depictions of compounds **6i**, **6j**, **8c**, and **8f** in the ATPase domain of GyrB (Figure 6 and Figure 7, Figures S105 and S106 from the Supplementary Materials), the sidechain of Ser108 is predicted to act as a HBD to one of the available nitrogen atoms in the compounds, either the thiadiazole heterocycle (**8c**, and **8f**, Figure 7 and Figure S106 from the Supplementary Materials), the 5-amino group of the thiadiazole heterocycle (**6i**, Figure S105 from the Supplementary Materials), or the hydrazone linker (**6j**, Figure 6). Parts of the compounds also fit in a hydrophobic binding pocket comprising various amino acids: the benzo[*f*]coumarin heterocycle of compounds **6i** and **6j** in a pocket comprising Val71, Val69, Val43, Phe169, Val167, and Val 120 (Figure 6 and Figure S105 from the Supplementary Materials), respectively, and the phenyl-thiazole region of compounds **8c** and **8f** in a pocket comprising Val97, Val93, Ile94, Phe104, and Ala100 (Figure 7 and Figure S106 from the Supplementary Materials). Moreover, it was observed that the positively charged Arg76 sidechain is predicted to be involved in a polar contact with one of the nitrogen atoms of the thiadiazole heterocycle in compound **6j** (Figure 6).

The predicted binding poses could be supplementarily explained by the DFT calculations. The regions in the compounds where the HOMO was localized (the hydrazone-thiazole and phenyl-thiazole regions) interacted primarily with the supposed regions of the protein where the LUMO was localized [61]. The electrostatic potential maps (Table S4 from the Supplementary Materials, Figure 6 and Figure 7, Figures S105 and S106 from the Supplementary Materials) provided further insight into predicting the binding of these compounds to the protein, based on their electronic densities. The white regions around the aromatic rings (for example, the coumarin and benzo[*f*]coumarin heterocycles in compounds **6a**–**j** or the 2-phenyl ring in compounds **8a**–**j**) confirmed their nonpolar and hydrophobic characteristics, which are important for the interaction with the protein through π-π stacking, van der Waals interactions, and other types of noncovalent interactions [62]. The red areas corresponded to the electron-rich regions, which can overlap the HOMO in some cases, and can interact with the protein through electron donation (for example, the 5-amino group in compound **6j**, Figure 6). The blue areas corresponded to electron-deficient regions, which can overlap the LUMO in some cases, for example the benzamide moiety substituted with electron-withdrawing groups in compounds **8a**–**j** (Table S3 from the Supplementary Materials, Figure 6 and Figure 7, Figures S105 and S106 from the Supplementary Materials) [63].

### 3.3. Antimicrobial Evaluation

All tested compounds **6a**–**j** and **8a**–**j** showed antibacterial activity in different potencies against the tested bacterial strains [64]. Overall, the antibacterial activity was better in the **8a**–**j** series than in the **6a**–**j** series.

All compounds **8a**–**j** were active on *E. coli* (MIC = 15.62 μg/mL) and *E. faecalis* (MICs = 31.25–62.50 μg/mL), the activity being similar or superior to ciprofloxacin (Table 4). The activity against *E. coli* also correlates with the results obtained in the molecular docking study on the ATPase domain of the GyrB 24 kDa from *E. coli* (Table 3). Nevertheless, this mechanism should be regarded as tentative, given that the study relied solely on molecular docking. Compounds **8e**–**j** also showed superior activity to ciprofloxacin against *S. derby* (MIC = 31.25 μg/mL). The activity against the other strains was inferior to ciprofloxacin (Table 4).

Compounds **6d**–**h** showed superior activity to ciprofloxacin against *S. derby* (MIC = 31.25 μg/mL) and *E. faecalis* (MIC = 62.50 μg/mL). Additionally, compounds **6b**, **6c**, and **6i** were more potent than ciprofloxacin against *E. faecalis*. The activity against the other strains was inferior to ciprofloxacin (Table 4).

The lowest antibacterial activity was registered for compounds **6a** and **6j** (except against *E. coli*). None of the tested compounds showed significant activity against *S. aureus* (Table 4).

The promising antibacterial activity against the tested Enterobacteriaceae strains (*E. coli* and *Salmonella spp.*) and *E. faecalis*, coupled with the low GI absorption predicted in silico, make compounds **6a**–**j** and **8a**–**j** good candidates for the development of novel antibacterials in enteric infections.

All tested compounds **6a**–**j** and **8a**–**j** showed antifungal activity in different potencies against the tested strains. The activity against *C. albicans* was similar to fluconazole (MIC = 15.62 μg/mL) in most cases, except for compounds **6c**, **6h**, **8a**, **8c**, and **8d** (MIC = 31.25 μg/mL) (Table 5). The antifungal activity was less potent against *A. brasiliensis* (MICs = 31.25–62.50 μg/mL) compared to *C. albicans* (Table 5).

Overall, the activity profile was not uniform across the tested strains. In contrast to the generally better activity observed for several Gram-negative strains and for *C. albicans*, the activity against *S. aureus* was consistently less favorable. This difference may reflect species-specific factors that influence effective exposure to the compounds [65,66].

### 3.4. Antibiofilm Activity

All tested compounds **6b**, **6c**, **6i**, **6j**, **8a**, **8b**, and **8e**–**j** showed antibiofilm activity with different potencies against the tested biofilms. Except for the inactive compounds **8b** and **8e**–**j** against *E. faecalis* BF, activity was registered in each of the other cases.

Gentamicin was used as a reference to compare the antibiofilm activity of the tested compounds primarily due to its ability to disrupt bacterial virulence factors, thus including biofilms [38,67].

Some concentration–response profiles were non-monotonic. This behavior may reflect the intrinsic biological complexity of biofilm formation and differential responses at sub-inhibitory concentrations [38,68,69,70].

The activity against *E. faecalis* BF was weak in all instances, including gentamicin (Table 6). The highest inhibition percentages were registered by compounds **6j** (27.75–23.05%) and **8a** (24.61–21.47%) at concentrations of 500–125 μg/mL, which were either superior or equal to gentamicin at the same concentrations (23.04–19.90%).

The most effective antibiofilm activity was observed against *P. aeruginosa* BF, where all tested compounds showed BF inhibitions over 50% at concentrations between 500 and 31.25 μg/mL. All compounds, except for compound **8b**, were still active at the lowest concentration, with superior BF inhibition (72.33–7.73%) compared to gentamicin (3.77%) (Table 6). The most potent compounds, with inhibition percentages over 50%, were **8e** (72.33%), **8h** (71.23%), **6i** (70.97%), **6b** (70.45%), **8j** (61.70%), and **6j** (52.57%).

The activity against *E. coli* BF was comparable to that of gentamicin at 500–62.50 μg/mL for all tested compounds, except for compounds **6i** (inactive at 62.50 μg/mL) and **8a** (38.01%—weak inhibition at 62.50 μg/mL). Compound **8b** showed additional weak BF inhibition percentages (12.08–4.30%) at 31.25–7.81 μg/mL (Table 6).

All tested compounds were regarded as weak inhibitors against *S. typhimurium* BF. None of the compounds (maximum inhibition percentage 39.37%) nor gentamicin (maximum inhibition percentage 45.09%) showed inhibition over 50% (Table 6). Compounds **6i**, **8a**, and **8g**–**j** kept their activity until 0.10 μg/mL (14.20–0.48%), although inferior to gentamicin (27.93%).

There was a more potent BF inhibition at 125 μg/mL than at higher concentrations against *E. coli* and *S. typhimurium* BFs. The activity of compound **8j** was superior to gentamicin against *E. coli* BF at this concentration (Table 6).

The antibiofilm activity against the tested BFs varied in the following order *P. aeruginosa* > *E. coli* > *S. typhimurium* > *E. faecalis*. A total of 10 out of 12 compounds were more active than gentamicin against *P. aeruginosa* BF at the lowest tested concentration. In the case of *E. coli* BF, no effective inhibitors were observed at concentrations under 62.50 μg/mL. Many compounds were active against *S. typhimurium* BF until the lowest concentration, but they were weak inhibitors at all tested concentrations. Finally, the activity against *E. faecalis* BF was the weakest, with compounds **8b** and **8e**–**j** completely inactive.

Based on the qualitative structure–activity relationship (SAR) studies in these compounds, it could be observed that compounds containing the coumarin heterocycle (**6b**, **6c**, **6i**, and **6j**) were more potent in many instances compared to the 2-phenylthiazolyl-substituted compounds (**8a**, **8b**, and **8e**–**j**).

The annulation of a supplementary ring to the existing coumarin heterocycle was favorable for the overall activity of the benzo[*f*]coumarin compounds **6i** and **6j**. A similar trend was observed in a previous antibiofilm study reported by our research group [38].

With respect to the 2-phenylthiazolyl compounds **8a**, **8b**, and **8e**–**j**, the most favorable substitutions for the overall antibiofilm activity were 4-methoxyphenyl (**8a**), adamantanyl (**8h**), and 3,4-difluorophenyl (**8j**). Additionally, the substitution with 4-nitrophenyl was responsible for maintaining compound **8b** active at 31.25–7.81 μg/mL against the *E. coli* BF.

**Figure 8:**
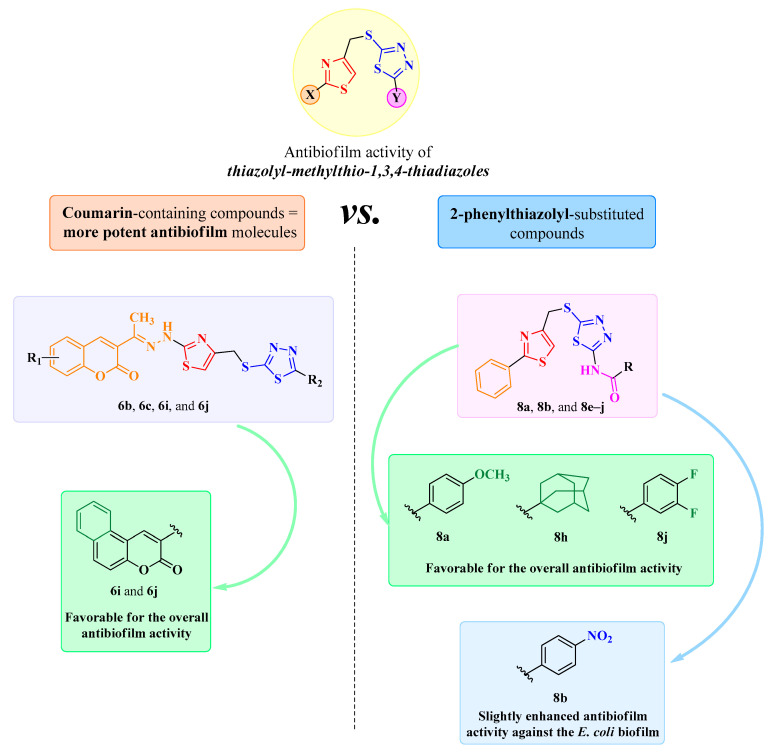
Qualitative SAR study of the antibiofilm activity of the novel thiazolyl-methylthio-1,3,4-thiadiazole hybrid compounds.

The qualitative SAR study of the antibiofilm activity for the compounds **6b**, **6c**, **6i**, **6j**, **8a**, **8b**, and **8e**–**j** is illustrated in Figure 8.

### 3.5. 2D-QSAR Studies

Based on the obtained equations from the Free-Wilson models of each antimicrobial activity against the tested strains, the contributions to the activity of the scaffold and each substituent are presented in Table 9. According to the obtained models, the contributions of x_2_ (8-ethoxycoumarinyl-hydrazonoethyl), x_6_ (phenyl), and y_1_ (methyl) substituents to the activity against the tested strains, excepting *E. coli*, were null. Similarly, the contribution of y_2_ (amino) substituent to the activity against *E. coli* was null. The codification of the substituents is presented in Section 4.

According to the ranking for the antibacterial activity against *E. coli*, the highest contribution to the activity was brought by the scaffold itself (C_s_ = 2.850). The similarity to halicin may be an explanation for this outcome, as this drug is considered potent against this strain (MICs = 8–32 µg/mL) [7,8].

The scaffold brought the most important contribution (C_s_ = 0.866) to the antibacterial activity against *S. enteritidis*, *S. typhimurium*, and *E. faecalis*, which was quantified by the same equation. The similarity to halicin may be an explanation for this outcome, as this drug is considered potent against these strains (MICs = 4–16 µg/mL), similarly to the previous case [7,71].

The substitution with 3,4-difluorobenzamide (**8j**, y_12_ = 0.289) and phenylacetamide (**8e**, y_7_ = 0.267) substituents in position 5 of the thiadiazole ring was the most favorable for the activity against *S. enteritidis*, *S. typhimurium*, and *E. faecalis*, both of which share a similar steric volume. Additionally, the most advantageous substituent (x_4_ = 0.044) in the position 2 of the thiazole ring was the 6-bromocoumarin-3-yl moiety (**6g**–**h**).

On the other hand, the benzamide derivatives para- or meta-substituted with large substituents like *p*-nitro (**8b**, y_4_ = −0.004), *m-*chloro (**8i**, y_11_ = −0.014) or *p*-methoxy (**8a**, y_3_ = −0.018) had lower activity. Ortho substitution (**8c**, y_5_ = −0.632) and furanyl bioisosteric substitution (**8d**, y_6_ = −0.662) were the most unfavorable for the activity against *S. enteritidis*, *S. typhimurium*, and *E. faecalis*. A large polycyclic substituent like benzo[*f*]coumarin-3-yl (**6i**–**j**) on the second position of the thiazole ring was also unfavorable for the activity (x_5_ = −0.283).

Additionally, the unsubstituted coumarin moiety in position 2 of the thiazole ring (**6a**–**b**, x_1_ = −0.330) and the unsubstituted benzamide moiety in position 5 of the thiazole ring (**8g**, y_9_ = −0.049) were unfavorable for the activity against *S. enteritidis*, *S. typhimurium*, and *E. faecalis*. Therefore, it was implied that the presence of a substituent on these aromatic moieties is necessary for the activity.

Similar to the previously mentioned model, the scaffold brought the highest contribution to the activity against *S. typhimurium* and *S. derby* food isolates (C_s_ = 0.987), while the ranking for the contribution of the substituents from position 2 of the thiazole ring was kept the same.

The most favorable substituents from position 5 of the thiadiazole ring for the activity against *S. typhimurium* and *S. derby* food isolates were the polyfluorinated ones (**8f** − y_8_ = 0.198 and **8j** − y_12_ = 0.168) and adamantanoylamide (**8h**, y_10_ = 0.189). The furanoylamide (**8d**, y_6_ = −0.782) and *o*-fluorobenzamide (**8c**, y_5_ = −0.753) substituents were again the most unfavorable for the activity.

In a similar manner as the previous cases, the scaffold brought the most important contribution to the activity against *P. aeruginosa* (C_s_ = 0.686), once again the explanation for this outcome being the similarity to the halicin, which showed similar potency against *P. aeruginosa* according to the literature [7].

Referring to substituents, the highest contribution to the activity was brought by the substituents from position 5 of the thiadiazole ring, namely 3,4-difluorobenzamide (**8j**, y_12_ = 0.469) and phenylacetamide (**8e**, y_7_ = 0.447), which are similar in terms of steric volume. The most favorable substituent grafted in position 2 of the thiazole ring was benzo[*f*]coumarin-3-yl (**6i**–**j**, x_5_ = 0.169), underlining the importance of a large polycyclic substituent for the activity against *P. aeruginosa*.

On the other hand, the activity was decreased when the compounds were substituted with 6-bromocoumarin-3-yl (**6g**–**h**, x_4_ = −0.107), 8-ethoxycoumarin-3-yl (**6e**–**f**, x_3_ = −0.137), coumarin-3-yl (**6a**–**b**, x_1_ = −0.180), 2-fluorobenzamide (**8c**, y_5_ = −0.452), and 2-furanoylamide (**8d**, y_6_ = −0.481).

According to the ranking, the highest contribution to the activity against *S. aureus* was brought by the substituents inserted into position 5 of the thiadiazole ring, particularly *p-*trifluoromethylbenzamide (**8f**, y_8_ = 0.620), *p*-methoxyphenylbenzamide (**8a**, y_3_ = 0.584), phenylacetamide (**8e**, y_7_ = 0.568), and benzamide (**8g**, y_9_ = 0.553).

In the case of this model, the contribution of the scaffold towards the activity was unfavorable (C_s_ = −0.037). This comes in contradiction with the data available regarding the antibacterial activity of halicin against *S. aureus*, which is a potent agent against this bacteria (MICs = 2–8 µg/mL) [7,8,72].

Based on the model, unfavorable contributions to the activity against *S. aureus* were brought by the substituents in compounds with inferior activity (MIC = 500 µg/mL) to ciprofloxacin (MIC = 125 µg/mL). Because of this difference in the activity, the contributions to the log E values are close to one another, from −0.004 to −0.060, and do not reflect properly the negative influence of these substituents towards the activity.

According to the ranking, the most important contribution to the activity against *C. albicans* was brought by the scaffold (C_s_ = 1.318). However, this time it cannot be compared to halicin because this drug was not tested on fungal strains.

All the coumarin substituents were favorable for the activity, while the benzamide substituents decreased the activity against *C. albicans*. The antifungal potential of coumarin-containing compounds is documented in the literature and, therefore, it explains the obtained results [73].

Similar to the previous model for the antifungal activity, the scaffold brought the most important contribution to the activity against *A. brasiliensis* (C_s_ = 1.047).

The importance of the coumarin moiety for designing antifungal compounds was observable again. However, the brominated substituent (**6g**–**h**, x_4_ = −0.107) decreased the activity. The most favorable substituents for the activity inserted in position 5 of the thiadiazole ring were *p*-trifluoromethylbenzamide (**8e**, y_8_ = 0.138), adamantanoylamide (**8h**, y_10_ = 0.129), and 3,4-difluorobenzamide (**8j**, y_12_ = 0.108). The most unfavorable substituents for the activity against *A. brasiliensis* were *o*-fluorobenzamide (**8c**, y_5_ = −0.211) and 2-furanoylamide (**8d**, y_6_ = −0.240).

As a general conclusion of the 2D-QSAR analysis, the scaffold brought the highest contribution to the activity against all tested strains, except on *S. aureus*, while the coumarin substituents were the most favorable for the antifungal activity, especially against *C. albicans*, and the benzamide substituents had mostly influenced the antibacterial activity.

**Figure 9:**
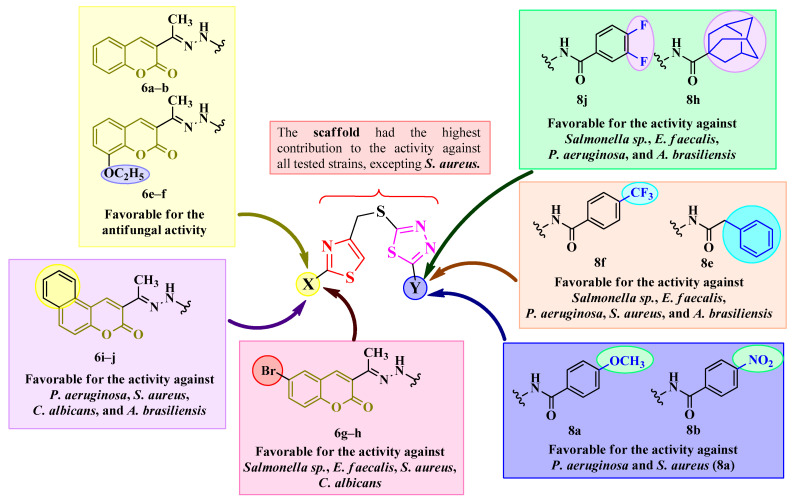
Summary of the quantitative structure–activity relationship in the antimicrobial compounds **6a**–**j** and **8a**–**j**.

The equations obtained in the Free-Wilson model can predict the compounds with the best biological activity by combining the substituents with the best contribution to the desired final effect. Based on the obtained Free-Wilson models, each substituent had a different contribution to the antimicrobial activity against a particular bacterial or fungal strain. A summary of the observed QSAR is illustrated in Figure 9.

One limitation of the present 2D-QSAR study is the relatively small dataset used for model development. In addition, the MIC values obtained for several tested strains are clustered into a limited number of repeated values. Consequently, while the QSAR analysis supports the structure–activity investigation, its predictive applicability is reduced.

## 4. Materials and Methods

### 4.1. Chemistry

The necessary reagents, solvents, and laboratory glassware were purchased from the local suppliers.

The reactions’ progress was verified using thin layer chromatography (TLC), employing silicagel 60 F_254_ as stationary phase and ethyl acetate:heptane 7:1 mixture as mobile phase.

The melting point (mp) was determined through the glass capillary method using an MPM-H1 (Schropp Gerätetechnik, Überlingen, Germany) melting point device.

All intermediate compounds were confirmed using infrared (IR) and mass spectral (MS) analysis, while the compounds **4a**–**e** and the final compounds were supplementarily confirmed by proton nuclear magnetic resonance (^1^H NMR) and carbon magnetic resonance (^13^C NMR) spectral analysis. The samples for the IR spectral analysis were prepared in KBr tablets under vacuum, while the analysis was performed on a FT/IR 61600 spectrometer (Jasco, Cremella, Italy). The samples for the MS analysis were dissolved in a mixture of acetonitrile and dimethylsulfoxide (DMSO) and the spectra were recorded using negative ionization modes on an Agilent 1100 series device, connected to an Agilent Ion Trap SL mass spectrometer (Agilent Technologies, Santa Clara, CA, USA). The samples for the NMR spectral analysis were dissolved in DMSO-*d*_6_ and the spectra were recorded using an Avance NMR spectrometer (Bruker, Karlsruhe, Germany), while tetramethylsilane (TMS) was used for calibration. The TMS peak was used as reference for the chemical shifts (ppm) calculation, which were reported in δ units. The identified signal multiplicity was presented using abbreviations for the peak patterns: app—apparent, s—singlet, d—doublet, dd—double doublet, t—triplet, q—quartet, and m—multiplet. To identify an atom in a specific region of the molecule, the following abbreviations were used: Adm—adamantane, Ar—phenyl, Cou—coumarin, Fur—furan, Tdz—thiadiazole, and Th—thiazole.

#### 4.1.1. Synthesis of Compounds **2a**–**e** and **3a**–**e**

The synthesis of compounds **2a**–**e** and **3a**–**e** was previously reported by other research groups [42,46,47,48,49,50,51,52,53]. The compounds were also previously resynthesized by our research group [35].

#### 4.1.2. Synthesis of Compounds **4a**–**f**

In order to obtain compounds **4a**–**e**, 15 mmol of compounds **3a**–**e** were mixed with 15 mL of anhydrous acetone and 5 mL of *N*,*N*-dimethylformamide (DMF), followed by the addition of 15 mmol of 1,3-dichloracetone. The mixture was stirred for approximately 30 min and left to sit for 3 days at room temperature. The progress of the reaction was frequently monitored through TLC. When the reaction was finished, the mixture was vacuum filtered and the obtained precipitate was abundantly washed with anhydrous acetone and left to dry. Finally, the precipitate was recrystallized in hot absolute methanol. This protocol represents a slightly adapted version of a previously reported method [74]. Compound **4a** was previously reported by another research group [43].

In order to obtain compound **4f**, the original protocol was employed in which 6 g (43.73 mmol) of thiobenzamide (**3f**) were dissolved in 30 mL of anhydrous acetone, followed by the dissolution of 5.56 g (43.79 mmol) of 1,3-dichloracetone. The mixture was stirred for 24 h at room temperature, followed by vacuum filtration and washing with diethyl ether. The obtained precipitate was slowly poured over 15 mL of concentrated sulfuric acid, with constant stirring and cooling in an ice bath. The mixture was left to sit for 2 h and then it was poured over ice with constant stirring. The obtained product was filtered and abundantly washed with water until it was free of acid. This intermediate was previously reported by another research group [44].

*(*E*)-1-(4-(Chloromethyl)thiazol-2-yl)-2-(1-(2-oxo-2*H*-chromen-3-yl)ethylidene)hydrazin-1-ium chloride* (**4a**): yellow solid; mp = 114–115 °C (no reported mp value in [43]); yield = 66%; FTIR (KBr) ν_max_ (cm^−1^): 3413 (N-H), 1708 (C=O), 1604 (C=C), 1568 (C=N), 1520 (C-N), 1234 (C-O), 759 (C-S), 705 (C-Cl); ESI^+^-MS: *m*/*z* 334.3 ([M+H]^+^); ^1^H-NMR (DMSO-*d*_6_, 500 MHz) δ (ppm): 8.66 (s, 1H, N-H), 8.21 (s, 1H, Cou), 7.85–7.84 (d, 1H, Cou, *J* = 8 Hz), 7.65–7.62 (app t, 1H, Cou, *J* ≈ 9.0 Hz), 7.44–7.42 (d, 1H, Cou, *J* = 8 Hz), 7.39–7.37 (app t, 1H, Cou, *J* ≈ 7.0 Hz), 7.02 (s, 1H, Th), 4.67 (s, 2H, -CH_2_-), 2.27 (s, 3H, -CH_3_); ^13^C-NMR (DMSO-*d*_6_, 125 MHz) δ (ppm): 169.3 (Th), 159.1 (C=O), 153.3 (Cou), 141.0 (Th), 132.3 (Cou), 129.1 (Cou), 126.2 (Cou), 124.7 (Cou), 118.8 (Cou), 116.1 (Cou), 115.9 (Cou), 109.1 (Cou), 47.1 (-CH_2_-), 16.3 (-CH_3_).
*(*E*)-1-(4-Chloromethyl)thiazol-2-yl)-2-(1-(8-methoxy-2-oxo-2*H*-chromen-3-yl)ethylidene)hydrazin-1-ium chloride* (**4b**): yellow-white solid; mp = carbonization over 159 °C; yield = 23%; FTIR (KBr) ν_max_ (cm^−1^): 3451 (N-H), 1707 (C=O), 1610 (C=C), 1578 (C=N), 1507 (C-N), 1285 (C-O ether), 1234 (C-O ester), 771 (C-S), 702 (C-Cl); ESI^+^-MS: *m*/*z* 364.4 ([M+H]^+^); ^1^H-NMR (DMSO-*d*_6_, 500 MHz) δ (ppm): 8.14 (s, 1H, Cou), 7.40–7.29 (m, 2H, Cou), 7.00 (s, 1H, Th), 4.60 (s, 2H, -CH_2_-), 3.92 (s, 3H, -OCH_3_), 2.26 (s, 3H, -CH_3_); ^13^C-NMR (DMSO-*d*_6_, 125 MHz) δ (ppm): 169.4 (Th), 158.8 (C=O), 146.2 (Cou), 142.6 (Cou), 141.0 (Th), 126.4 (Cou), 124.6 (Cou), 120.2 (Cou), 119.4 (Cou), 114.4 (Cou), 109.0 (Cou), 56.1 (-CH_3_), 41.4 (-CH_2_-), 16.2 (-CH_3_).
*(*E*)-1-(4-(Chloromethyl)thiazol-2-yl)-2-(1-(8-ethoxy-2-oxo-2*H*-chromen-3-yl)ethylidene)hydrazin-1-ium chloride* (**4c**): yellow crystals; mp = carbonization over 178 °C; yield = 73%; FTIR (KBr) ν_max_ (cm^−1^): 3411 (N-H), 1713 (C=O), 1608 (C=C), 1574 (C=N), 1515 (C-N), 1276 (C-O ether), 1234 (C-O ester), 772 (C-S), 703 (C-Cl); ESI^+^-MS: *m*/*z* 378.8 ([M+H]^+^); ^1^H-NMR (DMSO-*d*_6_, 500 MHz) δ (ppm): 8.13 (s, 1H, Cou), 7.38–7.27 (m, 3H, Cou), 7.00 (s, 1H, Th), 4.66 (s, 2H, -CH_2_-), 4.20–4.16 (q, 2H, -CH_2_-, *J* = 7 Hz), 2.26 (s, 3H, -CH_3_), 1.42–1.39 (t, 3H, -CH_3_, *J* = 7 Hz); ^13^C-NMR (DMSO-*d*_6_, 125 MHz) δ (ppm): 169.4 (Th), 158.9 (C=O), 145.4 (Cou), 142.7 (Cou), 141.0 (Th), 126.4 (Cou), 124.6 (Cou), 120.1 (Cou), 119.5 (Cou), 115.3 (Cou), 109.0 (Cou), 64.4 (-OCH_2_-), 41.4 (-CH_2_-), 16.2 (-CH_3_), 14.6 (-CH_3-_).
*(*E*)-2-(1-(6-Bromo-2-oxo-2*H*-chromen-3-yl)ethylidene-1-(4-(chloromethyl)thiazol-2-yl)hydrazin-1-ium chloride* (**4d**): yellow-white solid; mp = carbonization over 175 °C; yield = 60%; FTIR (KBr) ν_max_ (cm^−1^): 3442 (N-H), 1731 (C=O), 1611 (C=C), 1571 (C=N), 1507 (C-N), 1237 (C-O), 777 (C-S), 704 (C-Cl), 660 (C-Br); ESI^+^-MS: *m*/*z* 412.3 ([M+H]^+^); ^1^H-NMR (DMSO-*d*_6_, 500 MHz) δ (ppm): 8.14 (s, 1H, Cou), 8.13 (s, 1H, Cou), 7.79–7.76 (dd, 1H, Cou, *J* = 6.5, 2 Hz), 7.42–7.40 (d, 1H, Cou, *J* = 9 Hz), 7.00 (s, 1H, Th), 4.65 (s, 2H, -CH_2_-), 2.24 (s, 3H, -CH_3_); ^13^C-NMR (DMSO-*d*_6_, 125 MHz) δ (ppm): 169.4 (Th), 158.6 (C=O), 152.3 (Cou), 139.3 (Th), 134.4 (Cou), 131.0 (Cou), 127.4 (Cou), 120.8 (Cou), 118.2 (Cou), 116.2 (Cou), 109.0 (Cou), 41.5 (-CH_2_-), 16.1 (-CH_3_).
*(*E*)-1-(4-Chloromethyl)thiazol-2-yl)-2-(1-(3-oxo-3*H*-benzo[f]chromen-2-yl)ethylidene)hydrazin-1-ium chloride* (**4e**): yellow solid; mp = carbonization over 228 °C; yield = 30%; FTIR (KBr) ν_max_ (cm^−1^): 3445 (N-H), 1709 (C=O), 1628 (C=C), 1594 (C=N), 1507 (C-N), 1236 (C-O), 777 (C-S), 713 (C-Cl); ESI^+^-MS: *m*/*z* 406.2 ([M+H]^+^); ^1^H-NMR (DMSO-*d*_6_, 500 MHz) δ (ppm): 8.91 (s, 1H, Cou), 8.55–8.53 (d, 1H, Cou, *J* = 8.5 Hz), 8.21–8.19 (d, 1H, Cou, *J* = 9 Hz), 8.06–8.05 (d, 1H, Cou, *J* = 8 Hz), 7.76–7.73 (app t, 1H, Cou, *J* ≈ 7.8 Hz), 7.64–7.61 (t, 1H, Cou, *J* = 7 Hz), 7.59–7.57 (d, 1H, Cou, *J* = 9.5 Hz), 7.04 (s, 1H, Th), 4.69 (s, 2H, -CH_2_-), 2.35 (s, 3H, -CH_3_-); ^13^C-NMR (DMSO-*d*_6_, 125 MHz) δ (ppm): 169.4 (Th), 159.0 (C=O), 153.2 (Cou), 136.8 (Th), 133.6 (Cou), 129.9 (Cou), 128.9 (Cou), 128.8 (Cou), 128.5 (Cou), 126.2 (Cou), 125.2 (Cou), 122.1 (Cou), 116.4 (Cou), 112.8 (Cou), 109.3 (Cou), 41.0 (-CH_2_-), 16.4 (-CH_3_).
*4-(Chloromethyl)-2-phenylthiazole* (**4f**): light brown solid; mp = 48 °C (lit. mp = 51 °C [44]); yield = 87%; FTIR (KBr) ν_max_ (cm^−1^): 1601 (C=N), 1512 (C-N), 754 (C-S), 713 (C-Cl); ESI^+^-MS: *m*/*z* 209.9 ([M+H]^+^).

#### 4.1.3. Synthesis of Compounds **6a**–**k**

To obtain compounds **6a**–**j**, 2 mmol of 5-methyl-1,3,4-thiadiazole-2-thiol (**5a**) or 2 mmol of 5-amino-1,3,4-thiadiazole-2-thiol (5**b**) were dissolved in 10 mL of DMSO in a beaker, followed by the addition of 6 mmol of anhydrous potassium carbonate. The mixture was stirred for 30 min at room temperature and then 2 mmol of compounds **4a**–**e** were added to the reaction. The obtained mixture was energetically stirred for 3–4 h at room temperature until it changed its color to dark orange or red. The progress of the reaction was monitored through TLC. When the reaction was finished, the mixture was poured over ice under constant stirring. The suspension was neutralized with a hydrochloric acid 10% solution and some crystals of sodium chloride were added to accelerate the flocculation and precipitation of the final product. Finally, the suspension was vacuum filtered, washed with water, and dried. The final products were recrystallized in hot absolute methanol. This protocol represents a slightly adapted version of a previously reported method by our research group [75,76,77].

In order to obtain compound **6k**, the original method was employed in which 10 mmol of **5b** were dissolved in 25 mL of anhydrous acetone and 5 mL of DMF into a flask, followed by the addition of 20 mmol of potassium carbonate. The mixture was refluxed for 30 min using a water bath at 45 °C. Over the boiling mixture, 10 mmol of compound **4f** were added and the mixture was left to boil for one more hour, using TLC to monitor the progress. When the reaction was finished, the mixture was cooled on ice and then vacuum filtered. The obtained precipitate was constantly washed with cold water until it was free of any salts. The final product was recrystallized in hot absolute methanol. This intermediate was previously reported by our research group [45].

*(*E*)-3-(1-(2-(4-(((5-Methyl-1,3,4-thiadiazol-2-yl)thio)methyl)thiazol-2-yl)hydrazono)ethyl)-2*H*-chromen-2-one* (**6a**): yellow solid; mp = 207–208 °C; yield = 36%; FTIR (KBr) ν_max_ (cm^−1^): 3411 (N-H), 1719 (C=O), 1609 (C=C), 1567 (C=N), 1532 (C-N), 1236 (C-O), 753 (C-S); ESI^+^-MS: *m*/*z* 430.0 ([M+H]^+^), 452.0 ([M+Na]^+^); ^1^H-NMR (DMSO-*d*_6_, 500 MHz) δ (ppm): 11.36 (s, 1H, N-H), 8.13 (s, 1H, Cou), 7.85–7.83 (dd, 1H, Cou, *J* = 6.5, 1.5 Hz), 7.66–7.62 (m, 1H, Cou), 7.44–7.43 (d, 1H, Cou, *J* = 8 Hz), 7.40–7.37 (td, 1H, Cou, *J* = 6.5, 1 Hz), 6.84 (s, 1H, Th), 4.44 (s, 2H, -CH_2_-S-), 2.68 (s, 3H, -CH_3_), 2.24 (s, 3H, -CH_3_); ^13^C-NMR (DMSO-*d*_6_, 125 MHz) δ (ppm): 169.5 (Th), 159.1 (C=O), 153.3 (Tdz), 140.6 (Th), 132.1 (Cou), 129.0 (Cou), 126.5 (Cou), 124.7 (Cou), 118.8 (Cou), 115.9 (Cou), 34.1 (-CH_2_-), 16.1 (-CH_3_), 15.2 (-CH_3_).
*(*E*)-3-(1-(2-(4-(((5-Amino-1,3,4-thiadiazol-2-yl)thio)methyl)thiazol-2-yl)hydrazono)ethyl)-2*H*-chromen-2-one* (**6b**): yellow-green solid; mp = 199 °C; yield = 47%; FTIR (KBr) ν_max_ (cm^−1^): 3413 (N-H), 1723 (C=O), 1604 (C=C), 1569 (C=N), 1507 (C-N), 1233 (C-O), 755 (C-S); ESI^+^-MS: *m*/*z* 431.0 ([M-H]^+^); ^1^H-NMR (DMSO-*d*_6_, 500 MHz) δ (ppm): 11.37 (s, 1H, N-H), 8.13 (s, 1H, Cou), 7.85–7.83 (dd, 1H, Cou, *J* = 6.5, 1.5 Hz), 7.65–7.62 (m, 1H, Cou), 7.44–7.42 (d, 1H, Cou, *J* = 8 Hz), 7.39–7.36 (td, 1H, Cou, *J* = 6.5, 1 Hz), 6.72 (s, 1H, Th), 4.19 (s, 2H, -CH_2_-S-), 2.24 (s, 3H, -CH_3_); ^13^C-NMR (DMSO-*d*_6_, 125 MHz) δ (ppm): 170.1 (Tdz), 169.4 (Th), 159.1 (C=O), 153.2 (Tdz), 149.2 (Cou), 140.6 (Th), 132.1 (Cou), 129.0 (Cou), 126.5 (Cou), 124.7 (Cou), 118.8 (Cou), 115.9 (Cou), 35.1 (-CH_2_-), 16.1 (-CH_3_).
*(*E*)-8-Methoxy-3-(1-(2-(4-(((5-methyl-1,3,4-thiadiazol-2-yl)thio)methyl)thiazol-2-yl)hydrazono)ethyl)-2*H*-chromen-2-one* (**6c**): yellow solid; mp = 224 °C; yield = 43%; FTIR (KBr) ν_max_ (cm^−1^): 3449 (N-H), 1718 (C=O), 1627 (C=C), 1572 (C=N), 1533 (C-N), 1275 (C-O ether), 1236 (C-O ester), 782 (C-S); ESI^+^-MS: *m*/*z* 460.3 ([M+H]^+^); ^1^H-NMR (DMSO-*d*_6_, 500 MHz) δ (ppm): 11.36 (s, 1H, N-H), 8.09 (s, 1H, Cou), 7.38–7.36 (m, 1H, Cou), 7.33–7.30 (m, 2H, Cou), 6.83 (s, 1H, Th), 4.44 (s, 2H, -CH_2_-S-), 3.92 (s, 3H, -OCH_3_), 2.68 (s, 3H, -CH_3_), 2.24 (s, 3H, -CH_3_); ^13^C-NMR (DMSO-*d*_6_, 125 MHz) δ (ppm): 169.5 (Tdz), 165.6 (Th), 158.8 (C=O), 146.2 (Cou), 142.5 (Th), 140.8 (Cou), 126.6 (Cou), 124.6 (Cou), 120.1 (Cou), 119.4 (Cou), 114.4 (Th), 56.0 (-OCH_3_), 34.2 (-CH_2_-), 16.0 (-CH_3_), 15.2 (-CH_3_).
*(*E*)-3-(1-(2-(((5-Amino-1,3,4-thiadiazol-2-yl)thio)methyl)thiazol-2-yl)hydrazono)ethyl)-8-methoxy-2*H*-chromen-2-one* (**6d**): dark yellow solid; mp = carbonization over 215 °C, yield = 19%; FTIR (KBr) ν_max_ (cm^−1^): 3425 (N-H), 1709 (C=O), 1623 (C=C), 1570 (C=N), 1508 (C-N), 1274 (C-O ether), 1236 (C-O ester), 771 (C-S); ESI^+^-MS: *m*/*z* 461.2 ([M+H]^+^); ^1^H-NMR (DMSO-*d*_6_, 500 MHz) δ (ppm): 11.37 (s, 1H, N-H), 8.10 (s, 1H, Cou), 7.39–7.29 (m, 3H, Cou), 6.72 (s, 1H, Th), 4.19 (s, 2H, -CH_2_-S-), 3.92 (s, 3H, -OCH_3_), 2.24 (s, 3H, -CH_3_); ^13^C-NMR (DMSO-*d*_6_, 125 MHz) δ (ppm): 169.6 (Tdz), 163.4 (Th), 158.8 (C=O), 146.2 (Cou), 142.5 (Th), 140.8 (Cou), 124.6 (Cou), 120.1 (Cou), 119.4 (Cou), 114.4 (Cou), 113.3 (Cou), 103.0 (Th), 56.1 (-OCH_3_), 35.1 (-CH_2_-), 16.0 (-CH_3_).
*(*E*)-8-Ethoxy-3-(1-(2-(4-(((5-methyl-1,3,4-thiadiazol-2-yl)thio)methyl)thiazol-2-yl)hydrazono)ethyl)-2*H*-chromen-2-one* (**6e**): yellow solid; mp = 200–201 °C; yield = 69%; FTIR (KBr) ν_max_ (cm^−1^): 3413 (N-H), 1706 (C=O), 1609 (C=C), 1557 (C=N), 1505 (C-N), 1274 (C-O ether), 1236 (C-O ester), 769 (C-S); ESI^+^-MS: *m*/*z* 474.4 ([M+H]^+^); ^1^H-NMR (DMSO-*d*_6_, 500 MHz) δ (ppm): 11.36 (s, 1H, N-H), 8.08 (s, 1H, Cou), 7.37–7.35 (dd, 1H, Cou, *J* = 5, 2 Hz), 7.31–7.26 (m, 2H, Cou), 6.83 (s, 1H, Th), 4.44 (s, 2H, -CH_2_-S-), 4.20–4.16 (q, 2H, -CH_2_-, *J* = 7 Hz), 2.68 (s, 3H, -CH_3_), 2.24 (s, 3H, -CH_3_), 1.42–1.39 (t, 3H, -CH_3_, *J* = 7 Hz); ^13^C-NMR (DMSO-*d*_6_, 125 MHz) δ (ppm): 169.5 (Tdz), 165.6 (Th), 164.3 (Tdz), 158.9 (C=O), 145.4 (Cou), 142.6 (Th), 140.8 (Cou), 126.5 (Cou), 124.6 (Cou), 120.1 (Cou), 119.5 (Cou), 115.2 (Cou), 107.8 (Th), 64.3 (-OCH_2_-), 34.2 (-CH_2_-), 16.1 (-CH_3_), 15.2 (-CH_3_), 14.5 (-CH_3_).
*(*E*)-3-(1-(2-(4-(((5-Amino-1,3,4-thiadiazol-2-yl)thio)methyl)thiazol-2-yl)hydrazono)ethyl)-8-ethoxy-2*H*-chromen-2-one* (**6f**): yellow-green solid; mp = 208 °C; yield = 71%; FTIR (KBr) ν_max_ (cm^−1^): 3408 (N-H), 1702 (C=O), 1609 (C=C), 1568 (C=N), 1510 (C-N), 1277 (C-O ether), 1232 (C-O ester), 773 (C-S); ESI^+^-MS: *m*/*z* 475.3 ([M+H]^+^); ^1^H-NMR (DMSO-*d*_6_, 500 MHz) δ (ppm): 11.37 (s, 1H, N-H), 8.08 (s, 1H, Cou), 7.37–7.35 (m, 1H, Cou), 7.30–7.29 (m, 2H, Cou), 6.71 (s, 1H, Th), 4.20–4.16 (m, 4H, -O-CH_2_- and -CH_2_-S-), 2.24 (s, 3H, CH_3_), 1.42–1.39 (t, 3H, -CH_3_, *J* = 7 Hz); ^13^C-NMR (DMSO-*d*_6_, 125 MHz) δ (ppm): 170.1 (Tdz), 169.4 (Th), 158.9 (C=O), 158.7 (C=N), 149.2 (Tdz), 145.4 (Cou), 142.6 (Th), 140.8 (Cou), 126.6 (Cou), 124.6 (Cou), 120.3 (Cou), 120.1 (Cou), 119.5 (Cou), 115.2 (Cou), 64.4 (-OCH_2_-), 30.7 (-CH_2_-), 16.1 (-CH_3_), 14.6 (-CH_3_).
*(*E*)-6-Bromo-3-(1-(2-(4-(((5-methyl-1,3,4-thiadiazol-2-yl)thio)methyl)thiazol-2-yl)hydrazono)ethyl)-2*H*-chromen-2-one* (**6g**): yellow solid; mp = 207 °C; yield = 15%; FTIR (KBr) ν_max_ (cm^−1^): 3436 (N-H), 1733 (C=O), 1631 (C=C), 1573 (C=N), 1510 (C-N), 1233 (C-O), 778 (C-S), 622 (C-Br); ESI^+^-MS: *m*/*z* 508.2 ([M+H]^+^); ^1^H-NMR (DMSO-*d*_6_, 500 MHz) δ (ppm): 11.40 (s, 1H, N-H), 8.12–8.12 (d, 1H, Cou, *J* = 2.5 Hz), 8.09 (s, 1H, Cou), 7.78–7.76 (dd, 1H, Cou, *J* = 6, 2.5 Hz), 7.41–7.39 (d, 1H, Cou, *J* = 9 Hz), 6.85 (s, 1H, Th), 4.44 (s, 2H, -CH_2_-S-), 2.68 (s, 3H, -CH_3_), 2.23 (s, 3H, -CH_3_); ^13^C-NMR (DMSO-*d*_6_, 125 MHz) δ (ppm): 165.6 (Tdz), 164.3 (Th), 158.6 (C=O), 152.2 (Tdz), 139.2 (Th), 139.1 (Cou), 134.3 (Cou), 130.9 (Cou), 127.5 (Cou), 120.8 (Cou), 118.1 (Cou), 116.2 (Cou), 34.2 (-CH_2_-), 16.0 (-CH_3_), 15.2 (-CH_3_).
*(*E*)-3-(1-(2-(4-(((5-Amino-1,3,4-thiadiazol-2-yl)thio)methyl)thiazol-2-yl)hydrazono)ethyl)-6-bromo-2*H*-chromen-2-one* (**6h**): dark yellow solid; mp = 206 °C; yield = 46%; FTIR (KBr) ν_max_ (cm^−1^): 3421 (N-H), 1714 (C=O), 1622 (C=C), 1573 (C=N), 1507.10 (C-N), 1230 (C-O), 780 (C-S), 657 (C-Br); ESI^+^-MS: *m*/*z* 509.2 ([M+H]^+^); ^1^H-NMR (DMSO-*d*_6_, 500 MHz) δ (ppm): 11.41 (s, 1H, N-H), 8.11–8.11 (d, 1H, Cou, *J* = 2.5 Hz), 8.09 (s, 1H, Cou), 7.77–7.75 (dd, 1H, Cou, *J* = 6, 2.5 Hz), 7.40–7.38 (d, 1H, Cou, *J* = 9 Hz), 6.72 (s, 1H, Th), 4.19 (s, 2H, -CH_2_-S-), 2.23 (s, 3H, -CH_3_); ^13^C-NMR (DMSO-*d*_6_, 125 MHz) δ (ppm): 170.4 (Tdz), 170.1 (Th), 158.6 (C=O), 158.5 (C=N), 152.2 (Tdz), 149.2 (Cou), 139.7 (Th), 139.2 (Cou), 134.3 (Cou), 131.2 (Cou), 130.9 (Cou), 127.5 (Cou), 120.8 (Cou), 118.1 (Cou), 116.2 (Th), 30.6 (-CH_2_-), 16.0 (-CH_3_).
*(*E*)-2-(1-(2-(4-(((5-Methyl-1,3,4-thiadiazol-2-yl)thio)methyl)thiazol-2-yl)hydrazono)ethyl)-3*H*-benzo[f]chromen-3-one* (**6i**): yellow solid; mp = 235 °C; yield = 74%; FTIR (KBr) ν_max_ (cm^−1^): 3445 (N-H), 1719 (C=O), 1629 (C=C), 1576 (C=N), 1529 (C-N), 1235 (C-O), 783 (C-S); ESI^+^-MS: *m*/*z* 480.1 ([M+H]^+^), 502.1 ([M+Na]^+^); ^1^H-NMR (DMSO-*d*_6_, 500 MHz) δ (ppm): 11.45 (s, 1H, N-H), 8.89–8.88 (d, 1H, Cou, *J* = 6 Hz), 8.55–8.53 (d, 1H, Cou, *J* = 8.5 Hz), 8.24–8.22 (d, 1H, Cou, *J* = 9 Hz), 8.10–8.08 (d, 1H, Cou, *J* = 8.5 Hz), 7.79–7.76 (app t, 1H, Cou, *J* ≈ 7.2 Hz), 6.86 (s, 1H, Th), 4.46 (s, 2H, -CH_2_-S-), 2.69 (s, 3H, -CH_3_), 2.32 (s, 3H, -CH_3_); ^13^C-NMR (DMSO-*d*_6_, 125 MHz) δ (ppm): 173.8 (Tdz), 167.9 (Tdz), 164.8 (Th), 142.9 (Th), 139.3 (Cou), 136.5 (Cou), 133.5 (Cou), 129.9 (Cou), 129.4 (Cou), 128.9 (Cou), 128.5 (Cou), 126.2 (Cou), 125.2 (Cou), 123.2 (Cou), 122.5 (Cou), 122.0 (Cou), 116.4 (Cou), 112.9 (Cou), 107.3 (Th), 31.0 (-CH_2_-), 16.0 (-CH_3_), 15.2 (-CH_3_).
*(*E*)-2-(1-(2-(4-(((5-Amino-1,3,4-thiadiazol-2-yl)thio)methyl)thiazol-2-yl)hydrazono)ethyl)-3*H*-benzo[f]chromen-3-one* (**6j**): dark yellow solid; mp = 230 °C; yield = 55%; FTIR (KBr) ν_max_ (cm^−1^): 3430 (N-H), 1715 (C=O), 1626 (C=C), 1572 (C=N), 1507 (C-N), 1234 (C-O), 780 (C-S); ESI^+^-MS: *m*/*z* 481.0 ([M+H]^+^); ^1^H-NMR (DMSO-*d*_6_, 500 MHz) δ (ppm): 11.46 (s, 1H, N-H), 8.85 (s, 1H, Cou), 8.51–8.50 (d, 1H, Cou, *J* = 8.5 Hz), 8.21–8.19 (d, 1H, Cou, *J* = 9 Hz), 8.07–8.06 (d, 1H, Cou, *J* = 8.5 Hz), 7.77–7.74 (t, 1H, Cou, *J* = 8 Hz), 7.65–7.58 (m, 2H, Cou), 6.73 (s, 1H, Th), 4.21 (s, 2H, -CH_2_-S-), 2.32 (s, 3H, -CH_3_); ^13^C-NMR (DMSO-*d*_6_, 125 MHz) δ (ppm): 170.1 (Tdz), 159.0 (C=O), 153.1 (Cou), 149.2 (Th), 136.3 (Cou), 133.4 (Cou), 129.9 (Cou), 128.9 (Cou), 128.8 (Cou), 128.5 (Cou), 126.1 (Cou), 125.5 (Cou), 122.0 (Cou), 116.3 (Cou), 112.8 (Th), 30.6 (-CH_2_-), 16.0 (-CH_3_).
*5-(((2-Phenylthiazol-4-yl)methyl)thio)-1,3,4-thiadiazol-2-amine* (**6k**): white-green crystals; mp = 183 °C (lit. mp = 71–73 °C [45]); yield = 88%; FTIR (KBr) ν_max_ (cm^−1^): 3414 (N-H), 1626 (C=N), 1514 (C-N), 760 (C-S); ESI^+^-MS: *m*/*z* 307.0 ([M+H]^+^).

#### 4.1.4. Synthesis of Compounds **8a**–**j**

To obtain compounds **8a**–**j**, 2 mmol of **6k** were added in a beaker with 5 mL of tetrahydrofuran (THF), using 2.15 mmol of triethylamine as a catalyst. Then, 2 mmol of variously substituted acyl chlorides (**7a**–**j**) were added to the reaction. The mixture was kept cool and stirred for 5–6 h until the reaction was finished, which was confirmed via TLC. The excess solvent was left to evaporate and the precipitate was suspended in water and vacuum filtered. The obtained precipitate was washed with more water and left to dry. Finally, it was recrystallized in hot absolute methanol.

*4-Methoxy-*N*-(5-(((2-phenylthiazol-4-yl)methyl)thio-1,3,4-thiadiazol-2-yl)benzamide* (**8a**): white solid; mp = 248 °C; yield = 20%; FTIR (KBr) ν_max_ (cm^−1^): 3439 (N-H), 1658 (C=O amide I), 1604 (C=O amide II), 1578 (C=N), 1512 (C-N), 1265 (C-O), 780 (C-S); ESI^+^-MS: *m*/*z* 441.2 ([M+H]^+^); ^1^H-NMR (DMSO-*d*_6_, 500 MHz) δ (ppm): 12.94 (s, 1H, N-H), 8.13–8.11 (d, 2H, Ar, *J* = 8 Hz), 7.93 (m, 2H, Ar), 7.64 (s, 1H, Th), 7.49 (m, 3H, Ar), 7.10–7.08 (d, 2H, Ar, *J* = 8 Hz), 4.65 (s, 2H, -CH_2_-), 3.86 (s, 3H, -CH_3_); ^13^C-NMR (DMSO-*d*_6_, 125 MHz) δ (ppm): 167.3 (C=O), 163.0 (Tdz), 152.1 (Tdz), 132.7 (Th), 130.5 (Ar), 130.3 (Ar), 129.2 (Ar), 126.0 (Ar), 118.0 (Ar), 113.9 (Th), 55.5 (-CH_3_), 33.5 (-CH_2_-).
*4-Nitro-*N*-(5-(((2-phenylthiazol-4-yl)methyl)thio)-1,3,4-thiadiazol-2-yl)benzamide* (**8b**): pale yellow solid; mp = 258 °C; yield = 49%; FTIR (KBr) ν_max_ (cm^−1^): 3453 (N-H), 1655 (C=O amide I), 1604 (C=O amide II), 1574 (C=N), 1528 (N-O), 1513 (C-N), 1346 (N-O), 762 (C-S); ESI^+^-MS: *m*/*z* 456.0 ([M+H]^+^); ^1^H-NMR (DMSO-*d*_6_, 500 MHz) δ (ppm): 13.54 (s, 1H, N-H), 8.38–8.36 (d, 2H, Ar, *J* = 9 Hz), 8.31–8.30 (d, 2H, Ar, *J* = 9 Hz), 7.93–7.92 (m, 2H, Ar), 7.66 (s, 1H, Th), 7.50–7.48 (m, 3H, Ar), 4.67 (s, 2H, -CH_2_-); ^13^C-NMR (DMSO-*d*_6_, 125 MHz) δ (ppm): 167.3 (C=O), 152.0 (Tdz), 149.8 (Ar), 132.7 (Th), 130.3 (Ar), 129.9 (Ar), 129.2 (Ar), 126.0 (Ar), 123.6 (Ar), 118.1 (Th), 33.5 (-CH_2_-).
*2-Fluoro-*N*-(5-(((2-phenylthiazol-4-yl)methyl)thio)-1,3,4-thiadiazol-2-yl)benzamide* (**8c**): yellow crystals; mp = 174 °C; yield = 65%; FTIR (KBr) ν_max_ (cm^−1^): 3451 (N-H), 1679 (C=O amide I), 1614 (C=O amide II), 1543 (C=N), 1521 (C-N), 1307 (C-F), 752 (C-S); ESI^+^-MS: *m*/*z* 429.3 ([M+H]^+^); ^1^H-NMR (DMSO-*d*_6_, 500 MHz) δ (ppm): 13.16 (s, 1H, N-H), 7.94–7.92 (m, 2H, Ar), 7.78–7.75 (app t, 1H, Ar, *J* ≈ 7 Hz), 7.68–7.64 (m, 2H, Ar and Th), 7.51–7.49 (m, 3H, Ar), 7.41–7.34 (m, 2H, Ar), 4.66 (s, 2H, -CH_2_-); ^13^C-NMR (DMSO-*d*_6_, 125 MHz) δ (ppm): 167.3 (C=O), 160.4 (Tdz), 158.4 (Tdz), 152.1 (Ar), 134.1 (Th), 134.0 (Ar), 132.7 (Ar), 130.3 (Ar), 129.2 (Ar), 126.0 (Ar), 124.6 (Ar), 118.1 (Ar), 116.4 (Ar), 116.3 (Th), 33.6 (-CH_2_-).
N*-(5-(((2-Phenylthiazol-4-yl)methyl)thio)-1,3,4-thiadiazol-2-yl)furan-2-carboxamide* (**8d**): white crystals; mp = 197 °C; yield = 72%; FTIR (KBr) ν_max_ (cm^−1^): 3452 (N-H), 1673 (C=O amide I), 1604 (C=O amide II), 1543 (C=N), 1521 (C-N), 1310 (C-O), 754 (C-S); ESI^+^-MS: *m*/*z* 401.2 ([M+H]^+^); ^1^H-NMR (DMSO-*d*_6_, 500 MHz) δ (ppm): 13.11 (s, 1H, N-H), 8.05 (s, 1H, Fur), 7.93–7.91 (m, 2H, Ar), 7.71 (s, 1H, Fur), 7.64 (s, 1H, Th), 7.50–7.48 (m, 3H, Ar), 6.77 (s, 1H, Fur), 4.65 (s, 2H, -CH_2_-); ^13^C-NMR (DMSO-*d*_6_, 125 MHz) δ (ppm): 167.3 (C=O), 152.1 (Tdz), 147.8 (Fur), 132.7 (Th), 130.3 (Ar), 129.2 (Ar), 126.0 (Ar), 118.1 (Th), 117.5 (Ar), 112.4 (Fur), 33.5 (-CH_2_-).
*2-Phenyl-*N*-(5-(((2-phenylthiazol-4-yl)methyl)thio)-1,3,4-thiadiazol-2-yl)acetamide* (**8e**): white crystals; mp = 192–193 °C; yield = 66%; FTIR (KBr) ν_max_ (cm^−1^): 3450 (N-H), 1691 (C=O amide I), 1634 (C=O amide II), 1572 (C=N), 1510 (C-N), 764 (C-S); ESI^+^-MS: *m*/*z* 425.2 ([M+H]^+^); ^1^H-NMR (DMSO-*d*_6_, 500 MHz) δ (ppm): 12.88 (s, 1H, N-H), 7.90–7.88 (m, 2H, Ar), 7.61 (s, 1H, Th), 7.47–7.46 (m, 3H, Ar), 7.37–7.32 (m, 5H, Ar), 4.60 (s, 2H, -CH_2_-), 3.82 (s, 2H, -CH_2_-); ^13^C-NMR (DMSO-*d*_6_, 125 MHz) δ (ppm): 169.5 (C=O), 167.2 (Th), 159.2 (Tdz), 157.8 (Tdz), 134.4 (Th), 132.7 (Ar), 130.3 (Ar), 129.2 (Ar), 129.1 (Ar), 128.4 (Ar), 126.9 (Ar), 126.0 (Ar), 118.0 (Th), 41.3 (CH_2_), 33.5 (CH_2_).
N*-(5-(((2-Phenylthiazol-4-yl)methyl)thio)-1,3,4-thiadiazol-2-yl)-4-(trifluoromethyl)benzamide* (**8f**): white crystals; mp = 220 °C; yield = 85%; FTIR (KBr) ν_max_ (cm^−1^): 3448 (N-H), 1657 (C=O amide I), 1604 (C=O amide II), 1543 (C=N), 1512 (C-N), 1323 (C-F), 759 (C-S); ESI^+^-MS: *m*/*z* 479.2 ([M+H]^+^); ^1^H-NMR (DMSO-*d*_6_, 500 MHz) δ (ppm): 13.40 (s, 1H, N-H), 8.28–8.27 (d, 2H, Ar, *J* = 8 Hz), 7.92–7.91 (d, 2H, Ar, *J* = 8 Hz), 7.64 (s, 1H, Th), 7.50–7.47 (m, 3H, Ar), 4.66 (s, 2H, -CH_2_-); ^13^C-NMR (DMSO-*d*_6_, 125 MHz) δ (ppm): 167.3 (C=O), 158.5 (Tdz), 152.1 (Tdz), 135.2 (Th), 132.7 (Ar), 132.5 (Ar), 132.3 (Ar), 132.0 (Ar), 130.3 (Ar), 129.3 (Ar), 129.1 (Ar), 126.9 (Ar), 126.0 (Ar), 125.5 (Ar), 124.7 (Ar), 122.6 (-CF_3_), 120.4 (Ar), 118.0 (Th), 33.5 (-CH_2_-).
N*-(5-(((2-Phenylthiazol-4-yl)methyl)thio)-1,3,4-thiadiazol-2-yl)benzamide* (**8g**): white solid; mp = 157–158 °C; yield = 91%; FTIR (KBr) ν_max_ (cm^−1^): 3446 (N-H), 1676 (C=O amide I), 1636 (C=O amide II), 1558 (C=N), 1507 (C-N), 765 (C-S); ESI^+^-MS: *m*/*z* 411.1 ([M+H]^+^); ^1^H-NMR (DMSO-*d*_6_, 500 MHz) δ (ppm): 13.13 (s, 1H, N-H), 8.12–8.10 (d, 2H, Ar, *J* = 7.5 Hz), 7.94–7.92 (m, 2H, Ar), 7.69–7.65 (m, 2H, Th + Ar), 7.58–7.55 (m, 2H, Ar), 7.52–7.49 (m, 3H, Ar), 4.66 (s, 2H, -CH_2_-); ^13^C-NMR (DMSO-*d*_6_, 125 MHz) δ (ppm): 167.3 (C=O), 152.1 (Tdz), 133.0 (Th), 132.7 (Ar), 130.3 (Ar), 129.2 (Ar), 128.6 (Ar), 128.3 (Ar), 126.0 (Ar), 118.1 (Th), 33.5 (CH_2_).
*(3r,5r,7r)-*N*-(5-(((2-Phenylthiazol-4-yl)methyl)thio-1,3,4-thiadiazol-2-yl)adamantane-1-carboxamide* (**8h**): white solid; mp = 202 °C; yield = 26%; FTIR (KBr) ν_max_ (cm^−1^): 3445 (N-H), 1662 (C=O amide I), 1601 (C=O amide II), 1581 (C=N), 1530 (C-N), 765 (C-S); ESI^+^-MS: *m*/*z* 469.2 ([M+H]^+^); ^1^H-NMR (DMSO-*d*_6_, 500 MHz) δ (ppm): 12.24 (s, 1H, N-H), 7.92–7.90 (m, 2H, Ar), 7.62 (s, 1H, Th), 7.49–7.48 (m, 3H, Ar), 4.60 (s, 2H, -CH_2_-), 2.00–1.92 (m, 10H, Adm), 1.68 (m, 5H, Adm); ^13^C-NMR (DMSO-*d*_6_, 125 MHz) δ (ppm): 176.0 (C=O), 167.2 (Th), 160.0 (Tdz), 157.5 (Tdz), 152.2 (Th), 132.7 (Ar), 130.3 (Ar), 129.1 (Ar), 126.0 (Ar), 117.9 (Th), 45.3 (-CH_2_-), 40.5 (-CH_2_-), 38.4 (-CH_2_-), 37.4 (-CH_2_-), 36.0 (-CH_2_-), 35.6 (-CH_2_-), 33.5 (-CH_2_-), 27.3 (CH).
*3-Chloro-*N*-(5-(((2-phenylthiazol-4-yl)methyl)thio)-1,3,4-thiadiazol-2-yl)benzamide* (**8i**): white solid; mp = 219 °C; yield = 94%; FTIR (KBr) ν_max_ (cm^−1^): 3449 (N-H), 1654 (C=O amide I), 1602 (C=O amide II), 1573 (C=N), 1534 (C-N), 756 (C-S), 683 (C-Cl); ESI^+^-MS: *m*/*z* 445.2 ([M+H]^+^); ^1^H-NMR (DMSO-*d*_6_, 500 MHz) δ (ppm): 13.24 (s, 1H, N-H), 8.17–8.16 (m, 1H, Ar), 8.05–8.04 (d, 1H, Ar, *J* = 8 Hz), 7.94–7.92 (m, 2H, Ar), 7.75–7.73 (d, 1H, Ar, *J* = 10 Hz), 7.65 (s, 1H, Th), 7.61–7.58 (m, 1H, Ar), 7.51–7.49 (m, 3H, Ar), 4.66 (s, 2H, -CH_2_-); ^13^C-NMR (DMSO-*d*_6_, 125 MHz) δ (ppm): 167.3 (C=O), 152.1 (Tdz), 133.4 (Th), 132.7 (Ar), 130.3 (Ar), 129.2 (Ar), 128.1 (Ar), 127.1 (Ar), 126.0 (Ar), 118.1 (Th), 33.5 (-CH_2_-).
*3,4-Difluoro-*N*-(5-(((2-phenylthiazol-4-yl)methyl)thio)-1,3,4-thiadiazol-2-yl)benzamide* (**8j**): white solid; mp = 221 °C; yield = 35%; FTIR (KBr) ν_max_ (cm^−1^): 3441 (N-H), 1650 (C=O amide I), 1602 (C=O amide II), 1558 (C=N), 1530 (C-N), 1302 (C-F), 765 (C-S); ESI^+^-MS: *m*/*z* 446.9 ([M+H]^+^); ^1^H-NMR (DMSO-*d*_6_, 500 MHz) δ (ppm): 13.24 (s, 1H, N-H), 8.20–8.16 (m, 1H, Ar), 8.02–7.99 (m, 1H, Ar), 7.93–7.91 (m, 2H, Ar), 7.67–7.62 (m, 2H, Th + Ar), 7.50–7.48 (m, 3H, Ar), 4.66 (s, 2H, -CH_2_-); ^13^C-NMR (DMSO-*d*_6_, 125 MHz) δ (ppm): 167.3 (C=O), 153.4 (Tdz), 152.1 (Th), 151.4 (Tdz), 150.2 (Ar), 148.2 (Ar), 132.7 (Th), 130.3 (Ar), 129.2 (Ar), 126.0 (Ar), 118.1 (Th), 33.5 (-CH_2_-).

### 4.2. In Silico Evaluation

#### 4.2.1. ADMETox in Silico Studies

The in silico prediction of the pharmacokinetic and toxicologic profiles of the compounds **6a**–**j** and **8a**–**j** was performed using SwissADME and admetSAR 2.0 web tools, and Toxtree 3.1.0 open-source software [22,30,31,78]. Toxtree 3.1.0 software was used to predict the acute oral toxicity, using the revised Cramer decision tree method [31].

#### 4.2.2. DFT Calculations

The calculations for the studied compounds **6a**–**j** and **8a**–**j** were performed using Spartan24 (Wavefunction, Irvine, CA, USA) at the M06-2X level of theory with the 6-311++G(D,P) basis set on an AMD Ryzen9 7900 (Advanced Micro Devices, Inc., Santa Clara, CA, USA).

To evaluate how the polarity of the solvent affects the structural features of the compounds influencing their electronic and molecular properties, the calculations were conducted with vacuum, nonpolar solvent (ε = 7.43), polar solvent (ε = 37.22), and water as environments.

#### 4.2.3. Molecular Docking

The binding affinity of compounds **6a**–**j** and **8a**–**j** to the ATPase domain of DNA Gyrase B was evaluated using AutoDock Vina 1.1.2 [79,80]. The target macromolecule was obtained from Protein Data Bank (PDB ID: 5MMN) [39,81]. The search space was set as a cube with sides equal to 20 for each site, with Cartesian coordinates of the center of the searching space as x = 0.912, y = 5.895, z = −10.141, centered on the previously crystallized ligand, and the binding site confirmed by performing a supplementary BLAST (Basic Local Alignment Search Tool) using BLAST+ 2.17.0 (National Institutes of Health, Bethesda, MD, USA), which indicated the amino acids involved in ATP binding [82]. The preparation of the ligands and target was made using AutoDockTools 4.2.6 according to the previously reported protocol [80,83,84,85]. The target protein was preliminarily modeled to complete missing residues using SWISS-MODEL [86,87]. The visualization of the results of the molecular docking study was performed using Chimera 1.10.2 [88].

### 4.3. Antimicrobial Evaluation

The determination of the antimicrobial activity of compounds **6a**–**j** and **8a**–**j** was performed using the MIC method, according to a previously reported protocol by our research group [38].

### 4.4. Antibiofilm Evaluation

The determination of the antibiofilm activity of compounds **6b**, **6c**, **6i**, **6j**, **8a**, **8b**, and **8e**–**j** was performed according to a previously reported protocol by our research group [38].

### 4.5. 2D-QSAR Studies

#### Free-Wilson 2D-QSAR Model

The matrix creation and the two-sample assuming equal variances *t*-test were performed using Microsoft Excel 2021 software (Microsoft, Redmond, WA, USA). The multiple linear regression was performed using IBM SPSS Statistics 29.0.2.0 software (IBM, Armonk, NY, USA).

A 21x19 matrix was built after each compound was coded as “xnyn”, where “x” represents the substituents on the second position of the thiazole ring and “y” represents the substituents on the second position of the thiadiazole ring. In total, there were six distinctive “x” substituents and 12 “y” substituents. For each compound, the line in the matrix was completed with “1” if a specific substituent was present in the structure of the compound or “0” if it was absent (Table 10 and Table S5 from the Supplementary Materials). Additionally, a final column was added to the matrix containing the obtained antimicrobial activity expressed as log E, where E represents the inversed MIC (µM/mL). Each compound was defined by an equation, based on Equation (1). LOOCV analysis was performed using RStudio version 2023.12.1 build 402 (Posit PBC, Boston, MA, USA) [89].

Once the matrix was completed, it was imported into IBM SPSS Statistics software and multiple linear regression analysis was performed. Following this analysis, the obtained coefficients were extracted and used to calculate a new log E. The two log E values were compared using the two-sample assuming equal variances *t*-test [41,90].

## 5. Conclusions

The current study focused on the chemical design and synthesis of 20 novel thiazolyl-methyltio-1,3,4-thiadiazole hybrid compounds, followed by in silico and in vitro studies to evaluate their antimicrobial and antibiofilm activities. Further insight into the antimicrobial activity of these compounds was acquired through a 2D-QSAR study, using the Free-Wilson model.

The chemical design of compounds **6a**–**j** and **8a**–**j** stemmed from the structural analogy of the scaffold of these compounds with halicin, a potent promising antibacterial compound repurposed through AI from an antidiabetic compound, which was also demonstrated to possess antibiofilm potential. The synthesis of these compounds consisted of multiple condensation steps. All intermediate and final compounds were confirmed through spectral analysis.

The in silico studies aimed to evaluate the predictions regarding the druggability and ADMETox properties of these compounds. A molecular docking study was performed on the ATPase domain of the GyrB subunit from *E. coli* to benefit the assayed in vitro antibacterial activity. DFT calculations were performed to explore the electronic properties of compounds **6a**–**j** and **8a**–**j** and to gain insight into the predicted binding poses to the selected target. Based on the predicted druggability properties, all compounds had overall good potential to be further evaluated into drug development, with only two compounds (**6g** and **6h**) having one violation of Lipinski’s rule of five. According to the ADMETox predicted properties, the compounds had reduced GI absorption and BBB permeation capacity, which could limit the risk of pharmacokinetic drug–drug interactions and possible adverse effects. However, the predicted hepatotoxicity and high acute oral toxicity represent important liabilities that warrant further experimental validation before considering pharmaceutical development. DFT calculations revealed the localization of the frontier molecular orbitals across the two series of compounds and helped identifying the electronic densities in these compounds. Finally, the molecular docking study showed that compounds **6i**, **6j**, **8c**, and **8f** had the best binding affinities.

Based on the results obtained in the antimicrobial in vitro assay, all compounds showed both antibacterial and antifungal activities with different potencies. The overall antibacterial activity was better in compounds **8a**–**j** than **6a**–**j**. All compounds **8a**–**j** showed similar or superior activity to ciprofloxacin against *E. coli* and *E. faecalis*, with the in silico results from the molecular docking being correlated to the in vitro results. Compounds **6d**–**h** showed superior activity to ciprofloxacin against *S. derby* and *E. faecalis*. The antifungal activity was similar to fluconazole in most cases, while the activity against *A. brasiliensis* was less potent than that against *C. albicans*.

All tested compounds showed antibiofilm activity against the tested biofilms. The best activity was registered against the *P. aeruginosa* BF, where all tested compounds showed BF inhibitions over 50% at concentrations between 500 and 31.25 μg/mL and almost all tested compounds were more potent than gentamycin at 0.1 μg/mL.

The 2D-QSAR study conducted for the antimicrobial activity revealed that the thiazolyl-methylthio-thiadiazole scaffold provided the highest contribution to activity in almost all cases, except for the activity against *S. aureus*, where the highest contribution was provided by the *p-*trifluoromethylbenzamide substituent.

The investigated compounds are important for current research due to their analogue structure to halicin, the impact of the structural modulations of the scaffold and the substituents on the biological activity, compared to halicin.

## Figures and Tables

**Table 1:** The computed in silico physicochemical descriptors of compounds **6a**–**j** and **8a**–**j** with high significance for their pharmacokinetics. The prediction was done using SwissADME web tool [22].

Compound	MW (g/mol)	No. RB	No. HBA	No. HBD	TPSA (Å^2^)	MLogP	ESOL (µg/mL)	No. Lipinski Violations
**6a**	429.54	6	6	1	175.05	1.99	1.37	0
**6b**	430.53	6	6	2	201.07	1.64	4.02	0
**6c**	459.56	7	7	1	184.28	1.71	1.26	0
**6d**	460.55	7	7	2	210.30	1.37	3.70	0
**6e**	473.59	8	7	1	184.28	1.93	7.50	0
**6f**	474.58	8	7	2	210.30	1.60	2.20	0
**6g**	508.44	6	6	1	175.05	2.61	1.13	1
**6h**	509.42	6	6	2	201.07	2.27	5.91	1
**6i**	479.60	6	6	1	175.05	2.72	1.15	0
**6j**	480.59	6	6	2	201.07	2.39	3.37	0
**8a**	440.56	8	5	1	159.78	2.34	1.45	0
**8b**	455.53	8	6	1	195.37	1.74	1.54	0
**8c**	428.53	7	5	1	149.55	3.04	1.13	0
**8d**	400.50	7	5	1	162.69	1.68	4.21	0
**8e**	424.56	8	4	1	149.55	2.88	1.75	0
**8f**	478.53	8	7	1	149.55	3.48	2.61	0
**8g**	410.54	7	4	1	149.55	2.92	1.55	0
**8h**	468.66	7	4	1	149.55	3.76	3.37	0
**8i**	444.98	7	4	1	149.55	3.15	4.32	0
**8j**	446.52	7	6	1	149.55	3.42	8.28	0

**Table 2:** The energy levels of HOMO and LUMO for compounds **6a**–**j** and **8a**–**j** (eV).

Compound	Vacuum		Nonpolar Solvent (ε = 7.43)		Polar Solvent (ε = 37.22)		Water	
	HOMO	LUMO	HOMO	LUMO	HOMO	LUMO	HOMO	LUMO
**6a**	−7.02	−1.57	−7.08	−1.49	−7.10	−1.48	−7.10	−1.48
**6b**	−7.10	−1.59	−7.16	−1.51	−7.15	−1.49	−7.13	−1.48
**6c**	−6.96	−1.47	−7.06	−1.46	−7.10	−1.46	−7.10	−1.46
**6d**	−6.96	−1.47	−7.14	−1.48	−7.12	−1.46	−7.12	−1.46
**6e**	−6.96	−1.44	−7.07	−1.44	−7.10	−1.45	−7.10	−1.45
**6f**	−6.91	−1.43	−7.05	−1.45	−7.08	−1.45	−7.09	−1.46
**6g**	−7.10	−1.81	−7.10	−1.66	−7.10	−1.64	−7.10	−1.64
**6h**	−7.10	−1.81	−7.18	−1.67	−7.15	−1.63	−7.15	−1.62
**6i**	−6.95	−1.68	−7.01	−1.62	−7.02	−1.61	−7.03	−1.61
**6j**	−6.94	−1.70	−7.07	−1.66	−7.05	−1.64	−7.04	−1.63
**8a**	−7.66	−1.04	−7.66	−1.01	−7.66	−1.01	−7.66	−1.01
**8b**	−7.91	−1.99	−7.70	−2.07	−7.67	−2.10	−7.66	−2.10
**8c**	−7.64	−1.20	−7.61	−1.24	−7.61	−1.26	−7.61	−1.26
**8d**	−7.70	−1.13	−7.64	−1.10	−7.63	−1.12	−7.63	−1.13
**8e**	−7.64	−1.02	−7.61	−1.00	−7.61	−0.99	−7.61	−0.99
**8f**	−7.88	−1.45	−7.72	−1.33	−7.70	−1.32	−7.69	−1.32
**8g**	−7.73	−1.12	−7.70	−1.06	−7.69	−1.07	−7.69	−1.07
**8h**	−7.71	−1.00	−7.71	−0.97	−7.71	−0.97	−7.71	−0.97
**8i**	−7.77	−1.20	−7.71	−1.17	−7.70	−1.18	−7.70	−1.18
**8j**	−8.11	−1.13	−7.95	−1.15	−7.92	−1.16	−7.91	−1.17

**Table 3:** The results of the molecular docking of compounds **6a**–**j** and **8a**–**j** to the ATPase domain of GyrB expressed as the variation in Gibbs free energy (ΔG kcal/mol).

Compound	Binding Affinity	Compound	Binding Affinity
**6a**	−8.4	**8a**	−8.0
**6b**	−8.3	**8b**	−8.3
**6c**	−8.7	**8c**	−8.9
**6d**	−8.5	**8d**	−8.7
**6e**	−8.8	**8e**	−8.7
**6f**	−8.6	**8f**	−8.9
**6g**	−8.2	**8g**	−8.6
**6h**	−8.2	**8h**	−8.6
**6i**	−9.4	**8i**	−7.7
**6j**	−9.4	**8j**	−8.8

**Table 4:** The MIC (μg/mL) values of compounds **6a**–**j** and **8a**–**j** against the tested bacterial strains.

Compound	*E. coli* (ATCC 25922)	*S. enteritidis* (ATCC 13076)	*S.* *typhimurium* (ATCC 14028)	*S.* *typhimurium* (Food Isolate)	*S. derby* (Food Isolate)	*P. aeruginosa* (ATCC 27853)	*E. faecalis* (ATCC 29212)	*S. aureus* (ATCC 6538P)
**6a**	250	250	250	250	250	250	250	500
**6b**	15.62	62.50	62.50	62.50	62.50	62.50	**62.50**	500
**6c**	15.62	62.50	62.50	62.50	62.50	62.50	**62.50**	500
**6d**	31.25	62.50	62.50	31.25	**31.25**	125	**62.50**	500
**6e**	31.25	62.50	62.50	31.25	**31.25**	125	**62.50**	500
**6f**	31.25	62.50	62.50	31.25	**31.25**	125	**62.50**	500
**6g**	31.25	62.50	62.50	31.25	**31.25**	125	**62.50**	500
**6h**	31.25	62.50	62.50	31.25	**31.25**	125	**62.50**	500
**6i**	15.62	62.50	62.50	62.50	62.50	62.50	**62.50**	500
**6j**	31.25	250	250	250	250	62.50	250	500
**8a**	15.62	62.50	62.50	62.50	62.50	62.50	**62.50**	125
**8b**	15.62	62.50	62.50	62.50	62.50	62.50	**62.50**	500
**8c**	15.62	250	250	250	250	250	**62.50**	500
**8d**	15.62	250	250	250	250	250	**62.50**	500
**8e**	15.62	31.25	31.25	31.25	**31.25**	31.25	**62.50**	125
**8f**	15.62	62.50	62.50	31.25	**31.25**	62.50	**62.50**	125
**8g**	15.62	62.50	62.50	31.25	**31.25**	62.50	**62.50**	125
**8h**	15.62	62.50	62.50	31.25	**31.25**	62.50	**62.50**	500
**8i**	15.62	62.50	62.50	31.25	**31.25**	62.50	**62.50**	500
**8j**	15.62	62.50	62.50	31.25	**31.25**	31.25	**31.25**	500
**DMSO**	Bacterial growth in all wells							
**Ciprofloxacin**	15.62	15.62	15.62	15.62	62.50	31.25	125	15.62

**Table 5:** The MIC (μg/mL) values of compounds **6a**–**j** and **8a**–**j** against the tested fungal strains.

Compound	MIC (µg/mL)	
	*C. albicans* (ATCC 10231)	*A. brasiliensis* (ATCC 16404)
**6a**	15.62	31.25
**6b**	15.62	31.25
**6c**	31.25	31.25
**6d**	15.62	62.50
**6e**	15.62	31.25
**6f**	15.62	31.25
**6g**	15.62	62.50
**6h**	31.25	62.50
**6i**	15.62	31.25
**6j**	15.62	31.25
**8a**	31.25	62.50
**8b**	15.62	62.50
**8c**	31.25	62.50
**8d**	31.25	62.50
**8e**	15.62	31.25
**8f**	15.62	31.25
**8g**	15.62	31.25
**8h**	15.62	31.25
**8i**	15.62	31.25
**8j**	15.62	31.25
**DMSO**	Fungal growth in all wells	
**Fluconazole**	15.62	>250

**Table 6:** Percentage (%) of BF inhibition of compounds **6b**, **6c**, **6i**, **6j**, **8a**, **8b**, and **8e**–**j** against the tested bacterial biofilms.

Concentration (μg/mL)	C_1_ = 500	C_2_ = 250	C_3_ = 125	C_4_ = 62.50	C_5_ = 31.25	C_6_ = 15.62	C_7_ = 7.81	C_8_ = 2.60	C_9_ = 1.30	C_10_ = 0.60	C_11_ = 0.20	C_12_ = 0.10
Compound	BF inhibition (%)—*E. faecalis* ATCC 29212											
**6b**	15.18	19.90	19.90	–	–	–	–	–	–	–	–	–
**6c**	4.19	4.19	13.61	–	–	–	–	–	–	–	–	–
**6i**	16.75	21.47	18.32	–	–	–	–	–	–	–	–	–
**6j**	**27.75**	**26.18**	**23.04**	–	–	–	–	–	–	–	–	–
**8a**	**24.61**	21.47	**21.47**	–	–	–	–	–	–	–	–	–
**8b**	–	–	–	–	–	–	–	–	–	–	–	–
**8e**	–	–	–	–	–	–	–	–	–	–	–	–
**8f**	–	–	–	–	–	–	–	–	–	–	–	–
**8g**	–	–	–	–	–	–	–	–	–	–	–	–
**8h**	–	–	–	–	–	–	–	–	–	–	–	–
**8i**	2.62	–	–	–	–	–	–	–	–	–	–	–
**8j**	5.76	–	–	–	–	–	–	–	–	–	–	–
**Gentamicin**	23.04	21.47	19.90	15.18	–	–	–	–	–	–	–	–
	**BF inhibition (%)—*P. aeruginosa* ATCC 27583**											
**6b**	**96.63**	96.37	96.50	90.41	80.24	62.09	72.14	65.72	70.97	76.48	**77.13**	**70.45**
**6c**	95.79	96.18	96.24	90.02	79.72	6.69	79.72	73.69	71.42	72.59	12.20	**7.73**
**6i**	95.98	96.50	96.50	92.55	79.52	61.70	77.71	46.15	40.64	24.06	**73.89**	**70.97**
**6j**	96.44	96.70	96.57	96.37	88.47	65.53	60.28	30.86	65.59	74.21	**64.62**	**52.57**
**8a**	94.17	96.63	**96.76**	96.31	83.28	42.85	64.68	64.36	33.45	61.90	**75.57**	**36.43**
**8b**	94.69	95.20	96.05	95.46	81.79	69.48	–	–	–	–	–	–
**8e**	95.79	95.85	96.44	93.52	83.93	68.70	55.68	–	–	–	–	–
**8f**	95.40	95.85	96.24	90.09	72.59	60.34	31.64	–	67.28	40.84	**68.83**	**72.33**
**8g**	95.01	95.27	96.18	82.89	63.00	64.43	56.78	53.99	36.69	66.24	**60.86**	**36.04**
**8h**	95.01	95.72	96.37	86.26	54.64	54.25	–	64.23	59.70	55.55	37.47	**71.23**
**8i**	96.05	95.66	96.05	80.56	76.87	67.47	52.63	52.18	67.21	52.24	**71.10**	**44.66**
**8j**	96.44	95.66	96.37	94.49	63.06	66.30	46.87	48.16	61.25	52.05	**65.53**	**61.70**
**Gentamicin**	96.50	96.70	96.70	96.57	88.73	94.95	95.79	95.20	94.95	96.11	50.82	3.77
	**BF inhibition (%)—*E. coli* ATCC 25922**											
**6b**	84.95	85.21	86.25	77.95	–	–	–	–	–	–	–	–
**6c**	82.10	82.88	83.66	69.65	–	–	–	–	–	–	–	–
**6i**	85.73	85.73	85.21	–	–	–	–	–	–	–	–	–
**6j**	85.47	86.77	86.25	83.40	–	–	–	–	–	–	–	–
**8a**	**86.25**	85.99	84.69	38.01	–	–	–	–	–	–	–	–
**8b**	81.32	83.14	84.17	84.95	12.08	4.30	–	–	–	–	–	–
**8e**	83.66	85.99	85.47	70.17	–	–	–	–	–	–	–	–
**8f**	82.36	84.17	85.99	78.99	–	–	–	–	–	–	–	–
**8g**	81.58	83.40	86.77	68.61	–	–	–	–	–	–	–	–
**8h**	80.54	81.58	86.77	71.47	–	–	–	–	–	–	–	–
**8i**	83.40	83.40	85.73	77.69	–	–	–	–	–	–	–	–
**8j**	84.43	85.47	**87.03**	56.17	–	–	–	–	–	–	–	–
**Gentamicin**	85.99	87.03	86.77	87.03	82.88	85.21	83.40	83.40	84.43	79.25	80.28	15.71
	**BF inhibition (%)—*S. typhimurium* ATCC 14028**											
**6b**	30.22	32.51	32.51	22.21	8.48	19.92	15.35	14.20	14.20	23.36	–	–
**6c**	21.07	18.78	30.22	21.07	3.91	6.20	13.06	13.06	15.35	26.79	14.20	–
**6i**	26.79	30.22	33.65	27.93	22.21	17.64	19.92	22.21	19.92	38.23	11.92	3.91
**6j**	31.36	35.94	35.94	35.94	7.34	8.48	16.49	14.20	22.21	22.21	16.49	–
**8a**	37.08	37.08	39.37	32.51	8.48	17.64	24.50	15.35	18.78	34.80	22.21	3.91
**8b**	–	16.49	25.64	25.64	0.48	8.48	15.35	10.77	–	–	–	–
**8e**	19.92	27.93	26.79	26.79	18.78	10.77	15.35	13.06	15.35	22.21	8.48	–
**8f**	8.48	26.79	27.93	18.78	1.62	17.64	3.91	22.21	7.34	19.92	–	–
**8g**	1.62	23.36	31.36	32.51	2.76	16.49	25.64	17.64	10.77	25.64	3.91	0.48
**8h**	–	27.93	32.51	33.65	15.35	13.06	21.07	19.92	18.78	22.21	2.76	6.20
**8i**	9.63	18.78	23.36	26.79	11.92	18.78	31.36	23.36	18.78	25.64	17.64	14.20
**8j**	25.64	17.64	21.07	27.93	16.49	17.64	25.64	27.93	18.78	29.08	19.92	13.06
**Gentamicin**	43.95	38.23	45.09	40.51	43.95	35.94	41.66	38.23	39.37	39.37	29.08	27.93

**Table 7:** The Free-Wilson 2D-QSAR model.

Strain	Equation	R^2^	ΔCV
*E. coli*	log E = 2.850 − 1.410 × x1 − 1.600 × x2 − 1.743 × x3 − 1.710 × x4 − 1.580 × x5 − 0.550 × x6 + 0.150 × y1 − 0.850 × y3 − 0.830 × y4 − 0.860 × y5 − 0.890 × y6 − 0.860 × y7 − 0.810 × y8 − 0.880 × y9 − 0.820 × y10 − 0.840 × y11 − 0.840 × y12	0.840	0.0003579
*S. enteritidis* *S. typhimurium* *E. faecalis*	log E = 0.866 − 0.330 × x1 + 0.013 × x3 + 0.044 × x4 − 0.283 × x5 + 0.001 × y2 − 0.018 × y3 − 0.004 × y4 − 0.632 × y5 − 0.662 × y6 + 0.267 × y7 + 0.018 × y8 − 0.049 × y9 + 0.009 × y10 − 0.014 × y11 + 0.289 × y12	0.767	0.1191464
*S. typhimurium* (food isolate) *S. derby* (food isolate)	log E = 0.987 − 0.481 × x1 + 0.164 × x3 + 0.194 × x4 − 0.433 × x5 + 0.061 × y2 − 0.139 × y3 − 0.124 × y4 − 0.753 × y5 − 0.782 × y6 + 0.146 × y7 + 0.198 × y8 + 0.132 × y9 + 0.189 × y10 + 0.167 × y11 + 0.168 × y12	0.845	0.0966689
*P. aeruginosa*	log E = 0.686 − 0.180 × x1 − 0.137 × x3 − 0.107 × x4 + 0.169 × x5 + 0.061 × y2 + 0.162 × y3 + 0.177 × y4 − 0.452 × y5 − 0.481 × y6 + 0.447 × y7 + 0.198 × y8 + 0.132 × y9 + 0.189 × y10 + 0.167 × y11 + 0.469 × y12	0.843	0.1179366
*S. aureus*	log E = −0.037 − 0.029 × x1 + 0.013 × x3 + 0.044 × x4 + 0.019 × x5 + 0.001 × y2 + 0.584 × y3 − 0.004 × y4 − 0.030 × y5 − 0.060 × y6 + 0.568 × y7 + 0.620 × y8 + 0.553 × y9 + 0.009 × y10 − 0.014 × y11 − 0.013 × y12	1.000	0.0709040
*C. albicans*	log E = 1.318 + 0.121 × x1 + 0.164 × x3 + 0.044 × x4 + 0.169 × x5 + 0.001 × y2 − 1.072 × y3 − 0.455 × y4 − 0.482 × y5 − 0.511 × y6 − 0.486 × y7 − 0.434 × y8 − 0.501 × y9 − 0.443 × y10 − 0.466 × y11 − 0.163 × y12	0.962	0.0844221
*A. brasiliensis*	log E = 1.047 + 0.121 × x1 + 0.164 × x3 − 0.107 × x4 + 0.169 × x5 − 0.059 × y2 − 0.199 × y3 − 0.184 × y4 − 0.211 × y5 − 0.240 × y6 + 0.086 × y7 + 0.138 × y8 + 0.071 × y9 + 0.129 × y10 + 0.106 × y11 + 0.108 × y12	0.914	0.0202790

Legend: R^2^—coefficient of determination; ΔCV—coefficient of cross-validation. Color legend: Negative contributions to the activity were represented in the red color, while positive contributions in the green color.

**Table 8:** The observed and calculated lg E values for the antimicrobial activity of compounds **6a**–**j** and **8a**–**j**.

Comp.	*E. coli* (ATCC 25922)		*S.* *enteritidis* (ATCC 13076)		*S.* *typhimurium* (ATCC 14028)		*S.* *typhimurium* (Food Source)		*S. derby* (Food Source)		*P.* *aeruginosa* (ATCC 27853)		*E. faecalis* (ATCC 29212)		*S. aureus* (ATCC 6358P)		*C. albicans* (ATCC 10231)		*A.* *brasiliensis* (ATCC 16404)	
	lg E obs/calc		lg E obs/calc		lg E obs/calc		lg E obs/calc		lg E obs/calc		lg E obs/calc		lg E obs/calc		lg E obs/calc		lg E obs/calc		lg E obs/calc	
**6a**	0.235	–	0.235	0.536	0.235	0.536	0.235	0.506	0.235	0.506	0.235	0.506	0.235	0.536	−0.066	−0.066	1.439	1.439	1.138	1.168
**6b**	1.440	1.440	0.838	0.537	0.838	0.537	0.838	0.567	0.838	0.567	0.838	0.567	0.838	0.537	−0.065	−0.065	1.440	1.440	1.139	1.109
**6c**	1.469	1.400	0.866	0.866	0.866	0.866	0.866	0.987	0.866	0.987	0.866	0.686	0.866	0.866	−0.037	−0.037	1.167	1.318	1.167	1.047
**6d**	1.168	1.250	0.867	0.867	0.867	0.867	1.168	1.048	1.168	1.048	0.566	0.747	0.867	0.867	−0.036	−0.036	1.470	1.319	0.867	0.988
**6e**	1.181	1.260	0.880	0.879	0.880	0.879	1.181	1.151	1.181	1.151	0.578	0.549	0.880	0.879	−0.024	−0.024	1.482	1.482	1.181	1.211
**6f**	1.181	1.110	0.880	0.880	0.880	0.880	1.181	1.212	1.181	1.212	0.579	0.610	0.880	0.880	−0.023	−0.023	1.483	1.483	1.181	1.152
**6g**	1.211	1.290	0.910	0.910	0.910	0.910	1.211	1.181	1.211	1.181	0.609	0.579	0.910	0.910	0.007	0.007	1.513	1.362	0.910	0.940
**6h**	1.212	1.140	0.911	0.911	0.911	0.911	1.212	1.242	1.212	1.242	0.610	0.640	0.911	0.911	0.008	0.008	1.212	1.363	0.911	0.881
**6i**	1.487	1.420	0.885	0.583	0.885	0.583	0.885	0.554	0.885	0.554	0.885	0.855	0.885	0.583	−0.018	−0.018	1.487	1.487	1.186	1.216
**6j**	1.187	1.270	0.284	0.584	0.284	0.584	0.284	0.615	0.284	0.615	0.886	0.916	0.284	0.584	−0.017	−0.017	1.488	1.488	1.187	1.157
**8a**	1.450	1.450	0.848	0.848	0.848	0.848	0.848	0.848	0.848	0.848	0.848	0.848	0.848	0.848	0.547	0.547	0.246	0.246	0.848	0.848
**8b**	1,465	1.470	0.863	0.862	0.863	0.862	0.863	0.863	0.863	0.863	0.863	0.863	0.863	0.862	−0.040	−0.041	0.863	0.863	0.863	0.863
**8c**	1.438	1.440	0.234	0.234	0.234	0.234	0.234	0.234	0.234	0.234	0.234	0.234	0.836	0.836	−0.067	−0.067	0.836	0.836	0.836	0.836
**8d**	1.409	1.410	0.205	0.204	0.205	0.204	0.205	0.205	0.205	0.205	0.205	0.205	0.807	0.806	−0.096	−0.097	0.807	0.807	0.807	0.807
**8e**	1.434	1.440	1.133	1.133	1.133	1.133	1.133	1.133	1.133	1.133	1.133	1.133	0.832	0.832	0.531	0.531	0.832	0.832	1.133	1.133
**8f**	1.486	1.490	0.884	0.884	0.884	0.884	1.185	1.185	1.185	1.185	0.884	0.884	0.884	0.884	0.583	0.583	0.884	0.884	1.185	1.185
**8g**	1.420	1.420	0.817	0.817	0.817	0.817	1.119	1.119	1.119	1.119	0.817	0.826	0.817	0.817	0.516	0.516	0.817	0.817	1.119	1.118
**8h**	1.477	1.480	0.875	0.875	0.875	0.875	1.176	1.176	1.176	1.176	0.875	0.884	0.875	0.875	−0.028	−0.028	0.875	0.875	1.176	1.176
**8i**	1.455	1.460	0.852	0.852	0.852	0.852	1.153	1.154	1.153	1.154	0.852	0.861	0.852	0.852	−0.051	−0.051	0.852	0.852	1.153	1.153
**8j**	1.456	1.460	1.155	1.155	1.155	1.155	1.155	1.155	1.155	1.155	1.155	1.155	1.155	1.155	−0.049	−0.050	1.155	1.155	1.155	1.155

Legend: “–” signifies that compound **6a** was an outlier in the equation for the activity against *E. coli*.

**Table 9:** Contribution of the scaffold and of each substituent to the antimicrobial activity against the tested microbial strains.

Substituent	Contribution									
	*E. coli* (ATCC 25922)	*S.* *enteritidis* (ATCC 13076)	*S.* *typhimurium* (ATCC 14028)	*S.* *typhimurium* (Food Source)	*S. derby* (Food Source)	*P.* *aeruginosa* (ATCC 27853)	*E. faecalis* (ATCC 29212)	*S. aureus* (ATCC 6358P)	*C. albicans* (ATCC 10231)	*A.* *brasiliensis* (ATCC 16404)
**C_S_**	2.850	0.866	0.866	0.987	0.987	0.686	0.866	−0.037	1.318	1.047
**x_1_**	−1.410	−0.330	−0.330	−0.481	−0.481	−0.180	−0.330	−0.029	0.121	0.121
**x_2_**	−1.600	–	–	–	–	–	–	–	–	–
**x_3_**	−1.740	0.013	0.013	0.164	0.164	−0.137	0.013	0.013	0.164	0.164
**x_4_**	−1.710	0.044	0.044	0.194	0.194	−0.107	0.044	0.044	0.044	−0.107
**x_5_**	−1.580	−0.283	−0.283	−0.433	−0.433	0.169	−0.283	0.019	0.169	0.169
**x_6_**	−0.550	–	–	–	–	–	–	–	–	–
**y_1_**	0.150	–	–	–	–	–	–	–	–	–
**y_2_**	–	0.001	0.001	0.061	0.061	0.061	0.001	0.001	0.001	−0.059
**y_3_**	−0.850	−0.018	−0.018	−0.139	−0.139	0.162	−0.018	0.584	−1.072	−0.199
**y_4_**	−0.830	−0.004	−0.004	−0.124	−0.124	0.177	−0.004	−0.004	−0.455	−0.184
**y_5_**	−0.860	−0.632	−0.632	−0.753	−0.753	−0.452	−0.632	−0.030	−0.482	−0.211
**y_6_**	−0.890	−0.662	−0.662	−0.782	−0.782	−0.481	−0.662	−0.060	−0.511	−0.240
**y_7_**	−0.860	0.267	0.267	0.146	0.146	0.447	0.267	0.568	−0.486	0.086
**y_8_**	−0.810	0.018	0.018	0.198	0.198	0.198	0.018	0.620	−0.434	0.138
**y_9_**	−0.880	−0.049	−0.049	0.132	0.132	0.132	−0.049	0.553	−0.501	0.071
**y_10_**	−0.820	0.009	0.009	0.189	0.189	0.189	0.009	0.009	−0.443	0.129
**y_11_**	−0.840	−0.014	−0.014	0.167	0.167	0.167	−0.014	−0.014	−0.466	0.106
**y_12_**	−0.840	0.289	0.289	0.168	0.168	0.469	0.289	−0.013	−0.163	0.108

Legend: “–” signifies no contribution in the 2D-QSAR model.

**Table 10:** The general structure of the thiazolyl-methylthio-1,3,4-thiadiazoles and the substituents available in the tested compounds.

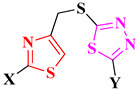			
Code	Substituent	Code	Substituent
**x1**	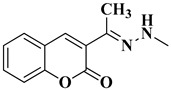	**y4**	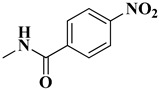
**x2**	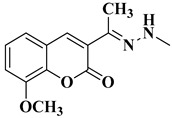	**y5**	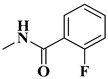
**x3**	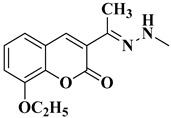	**y6**	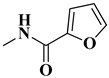
**x4**	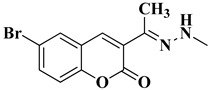	**y7**	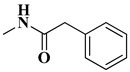
**x5**	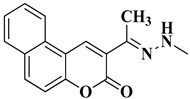	**y8**	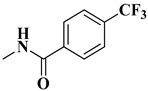
**x6**	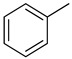	**y9**	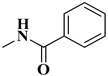
**y1**	**CH_3_^−^**	**y10**	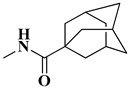
**y2**	**NH_2_^−^**	**y11**	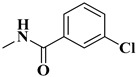
**y3**	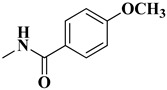	**y12**	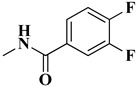

## Data Availability

The data presented in this study are available in this article.

## Supplementary Materials

The following supporting information can be downloaded at: https://www.mdpi.com/article/10.3390/antibiotics15050448/s1, Figures S1–S27: The IR spectra for compounds **4a**–**f**, **6a**–**k**, and **8a**–**j**; Figures S28–S54: The MS spectra for compounds **4a**–**f**, **6a**–**k**, and **8a**–**j**; Figures S55–S79: The ^1^H-NMR spectra for compounds **4a**–**e**, **6a**–**j**, and **8a**–**j**; Figures S80–S104: The ^13^C-NMR spectra for compounds **4a**–**e**, **6a**–**j**, and **8a**–**j**; Figures S105 and S106: The graphical depictions of the interaction between compounds **6i** and **8f** and the ATPase domain of the GyrB subunit; Figures S107–S110: The graphical depictions of compounds **6i**, **6j**, **8c**, and **8f** in the active site of the ATPase domain of the GyrB subunit displayed as surfaces; Tables S1 and S2: The computed in silico pharmacokinetic and toxicologic descriptors of compounds **6a**–**j** and **8a**–**j**; Table S3: The graphical depictions of the localization of the HOMO and LUMO frontier molecular orbitals in compounds **6a**–**j** and **8a**–**j**; Table S4: The graphical depictions of the electrostatic potential maps for compounds **6a**–**j** and **8a**–**j**. Table S5: The matrix of the thiazolyl-methylthio-thiadiazole compounds.
