# Supplementary material for: Thiazolyl-Methylthio-1,3,4-Thiadiazole Hybrids as Halicin Analogues with Antimicrobial and Antibiofilm Activities: Chemical Development, Biological Assessment, and 2D-QSAR Study
Source: Antibiotics (Basel). 2026 Apr 29;15(5):448. doi: 10.3390/antibiotics15050448 (PMC13203862; doi:10.3390/antibiotics15050448)
Supplement: Supplementary file 1 [file antibiotics-15-00448-s001.zip › antibiotics-4279701-supplementary.pdf]

antibiotics

MDPI

Article

Thiazolyl-Methylthio-1,3,4-Thiadiazole Hybrids as Halicin Analogues with Antimicrobial and Antibiofilm Activities: Chemical Development, Biological Assessment, and 2D-QSAR Study

Daniel Ungureanu [1], Gabriel Marc [2,*], Mihaela Niculina Duma [3], Dan Cristian Vodnar [4,5], Gheorghe-Adrian Martău [4,5], Laurian Vlase [6], Adrian Pîrnău [7], Brîndușa Tiperciuc [1], Cristina Moldovan [1], Ioana Ionuț [1], Anca Stana [1], Ilioara Oniga [8] and Ovidiu Oniga [1]

S1. Figures

S1.1. IR Spectra

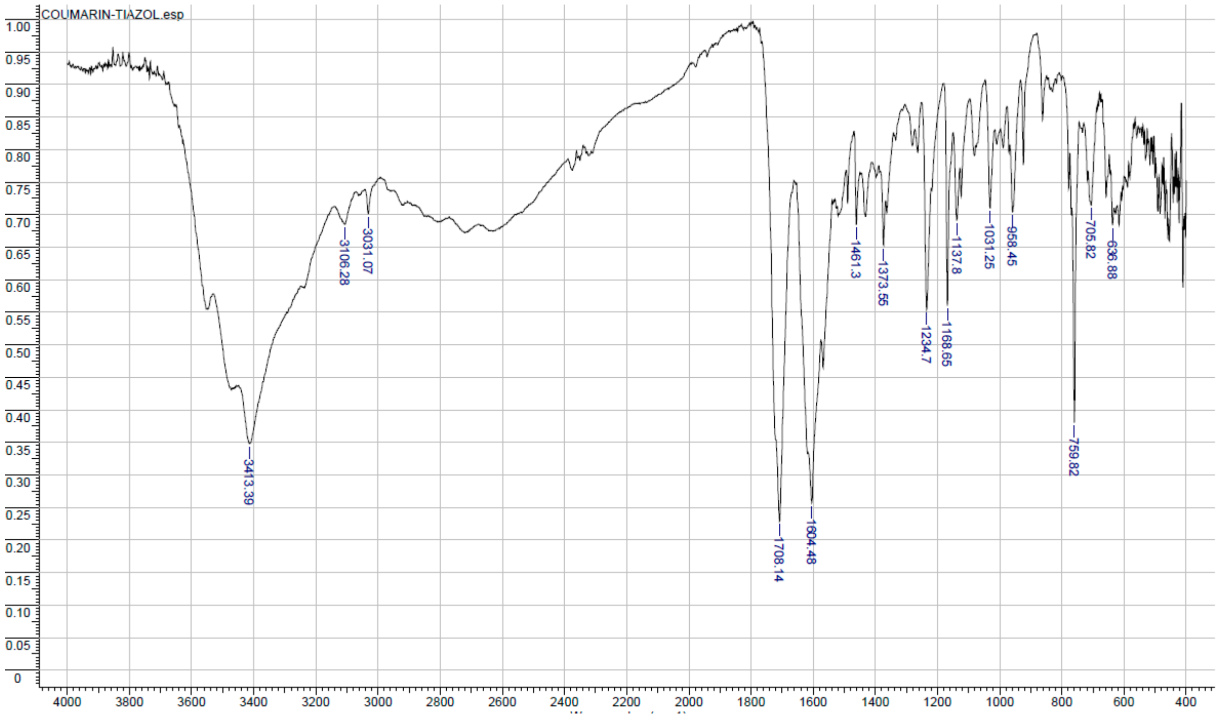

Figure S1. The IR spectrum for the compound **4a**.

 https://doi.org/10.3390/antibiotics15050448

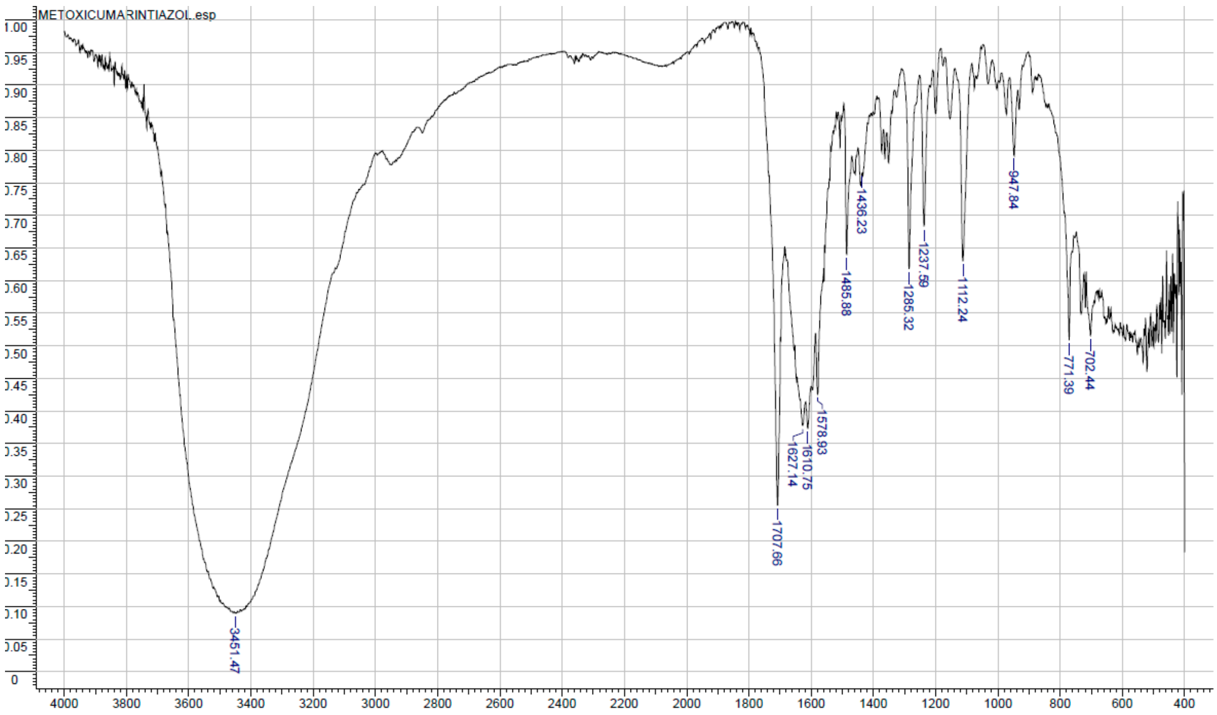

Figure S2. The IR spectrum for the compound **4b**.

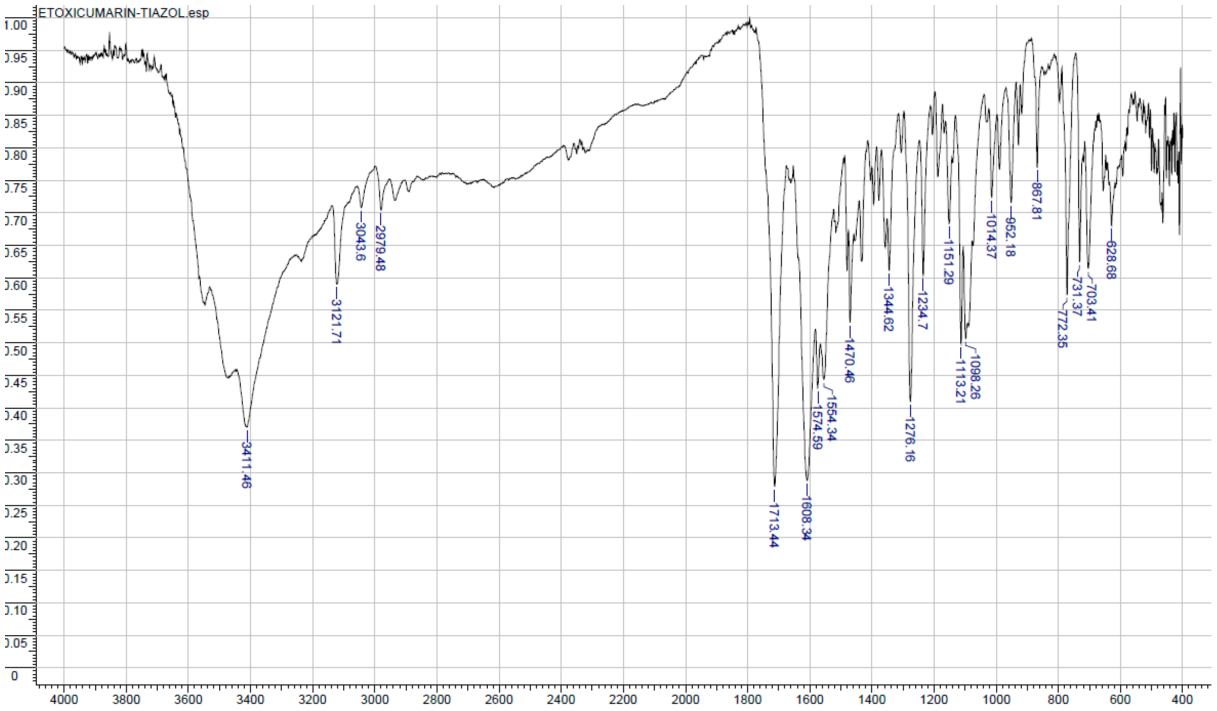

Figure S3. The IR spectrum for the compound **4c**.

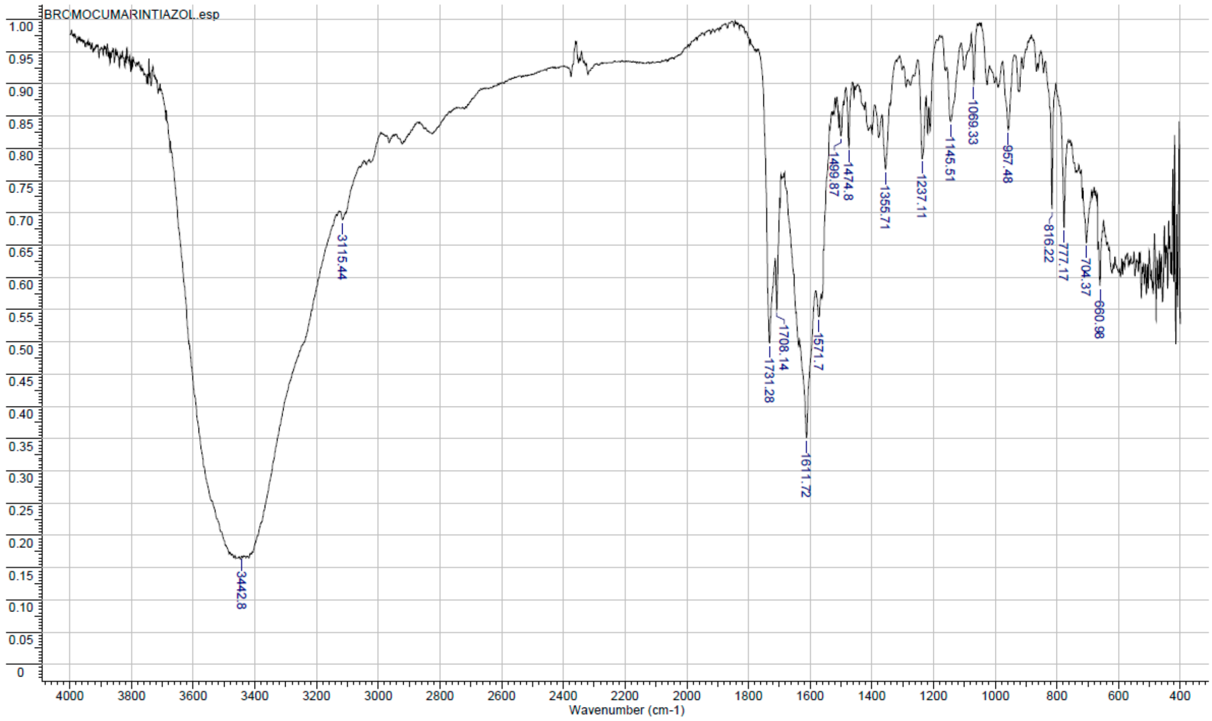

Figure S4. The IR spectrum for the compound **4d**.

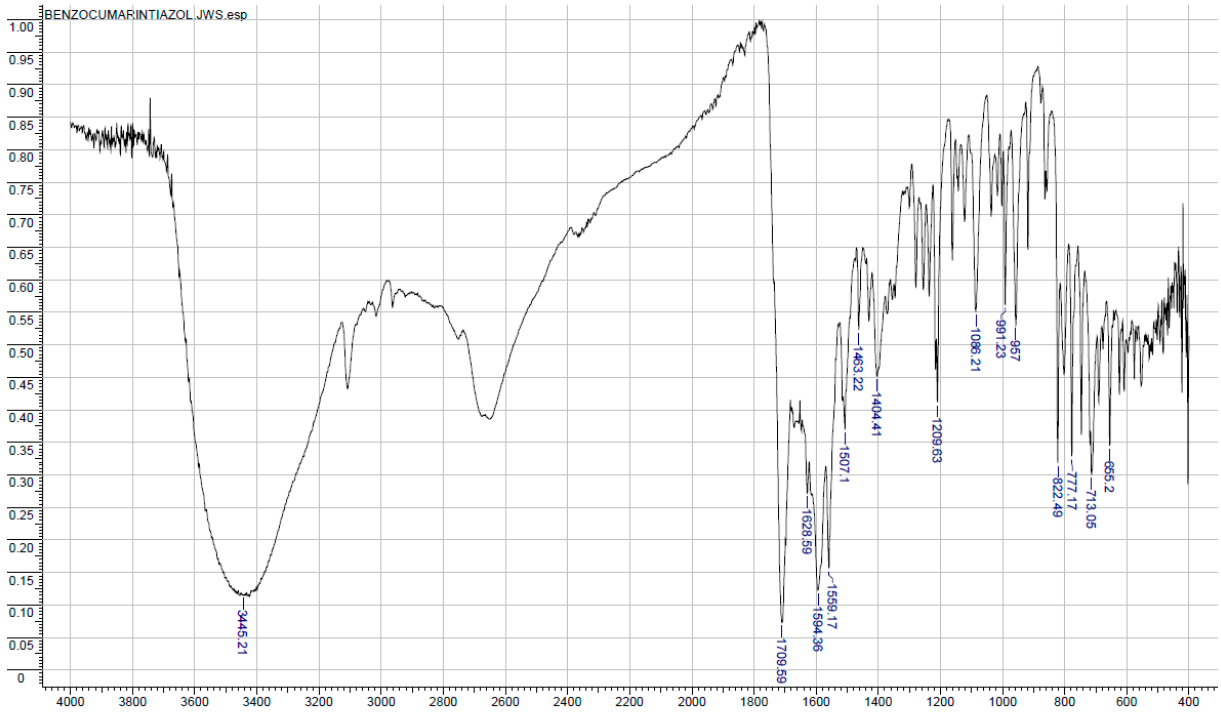

Figure S5. The IR spectrum for the compound **4e**.

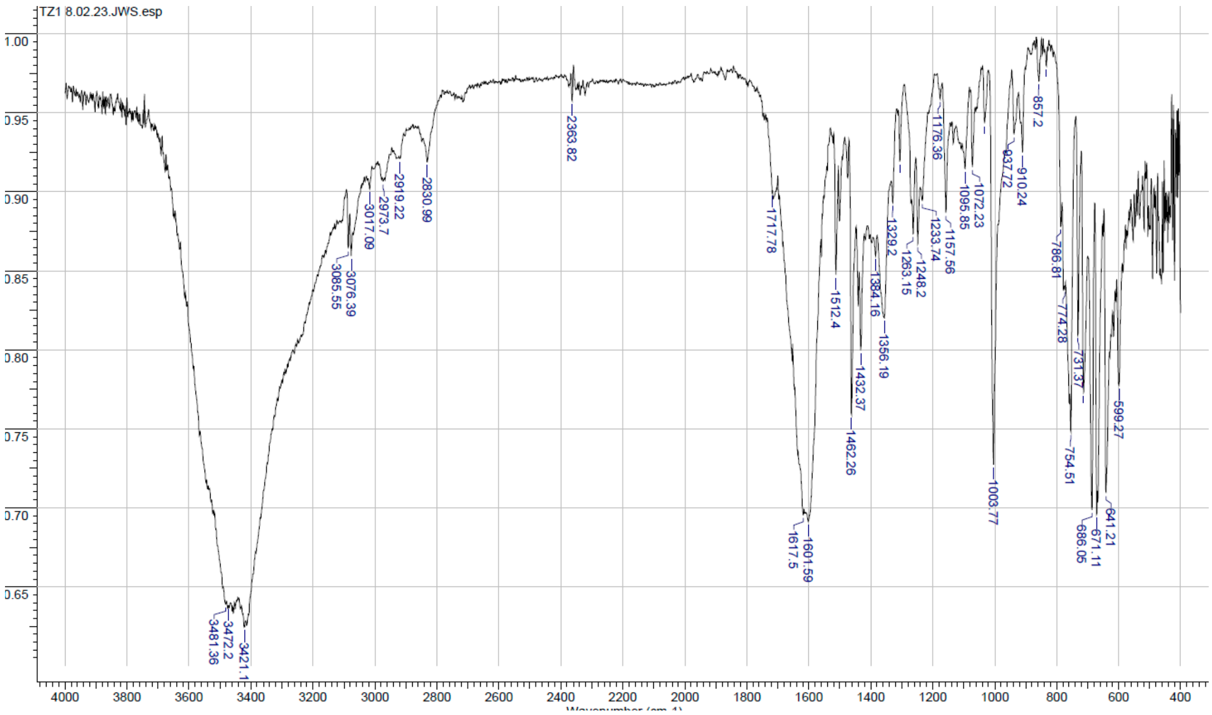

Figure S6. The IR spectrum for the compound **4f**.

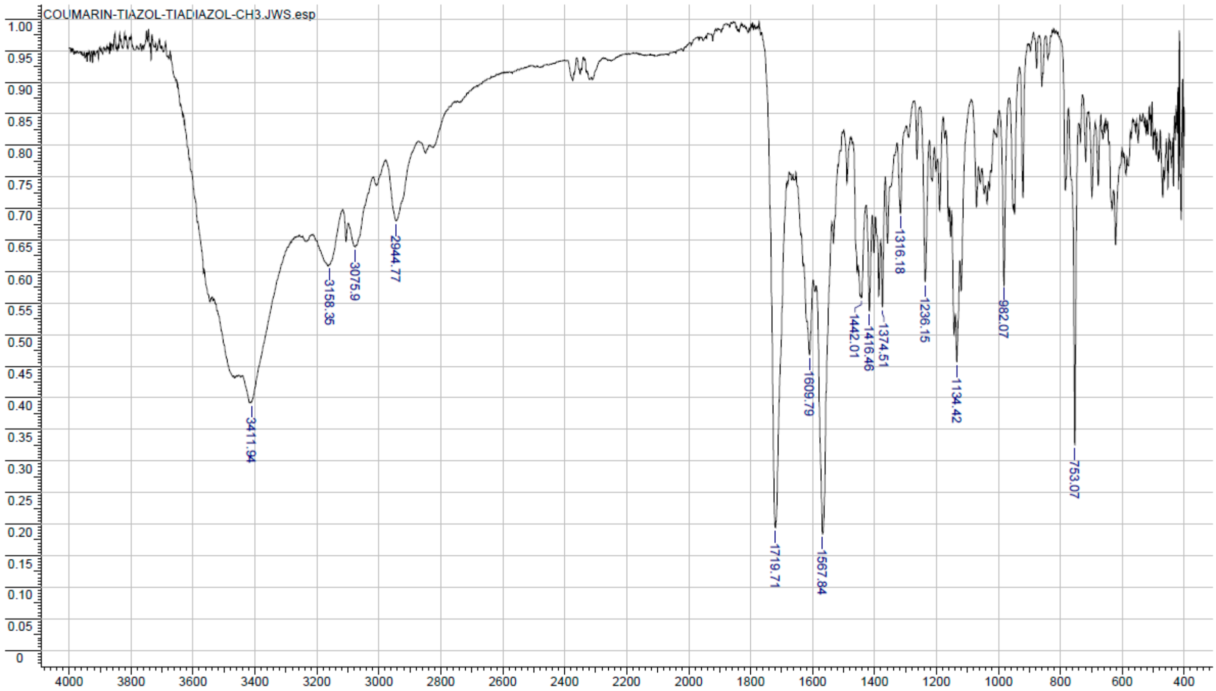

Figure S7. The IR spectrum for the compound **6a**.

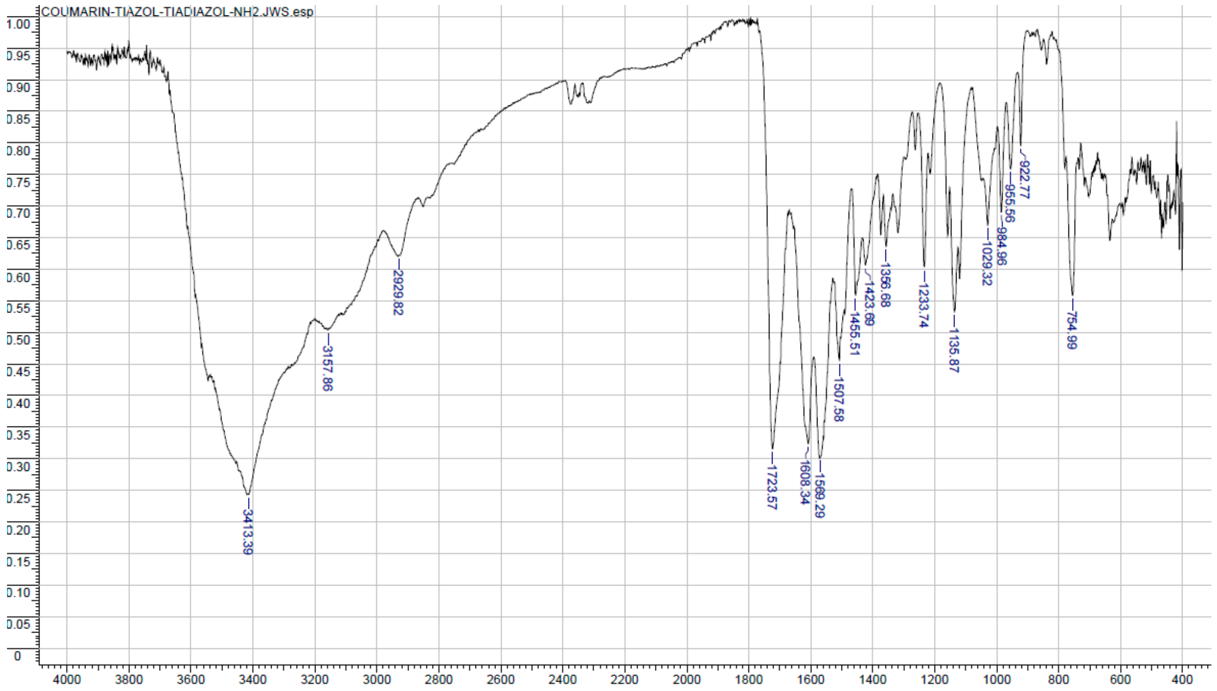

Figure S8. The IR spectrum for the compound **6b**.

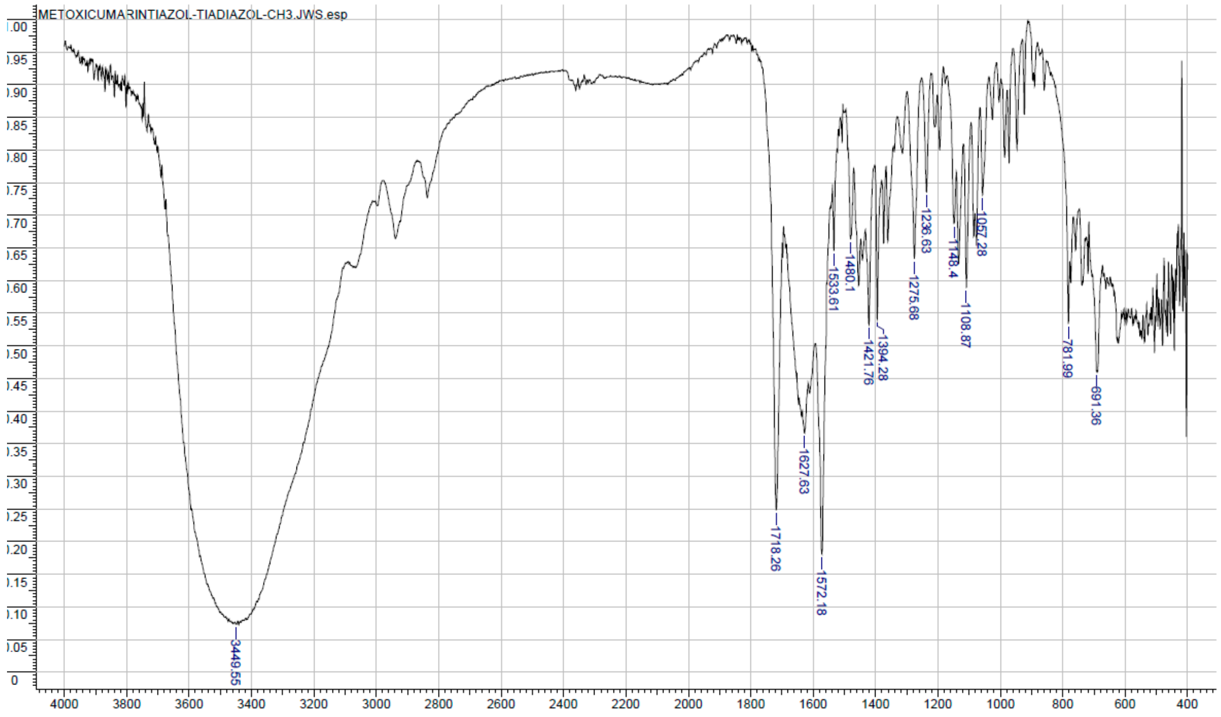

Figure S9. The IR spectrum for the compound **6c**.

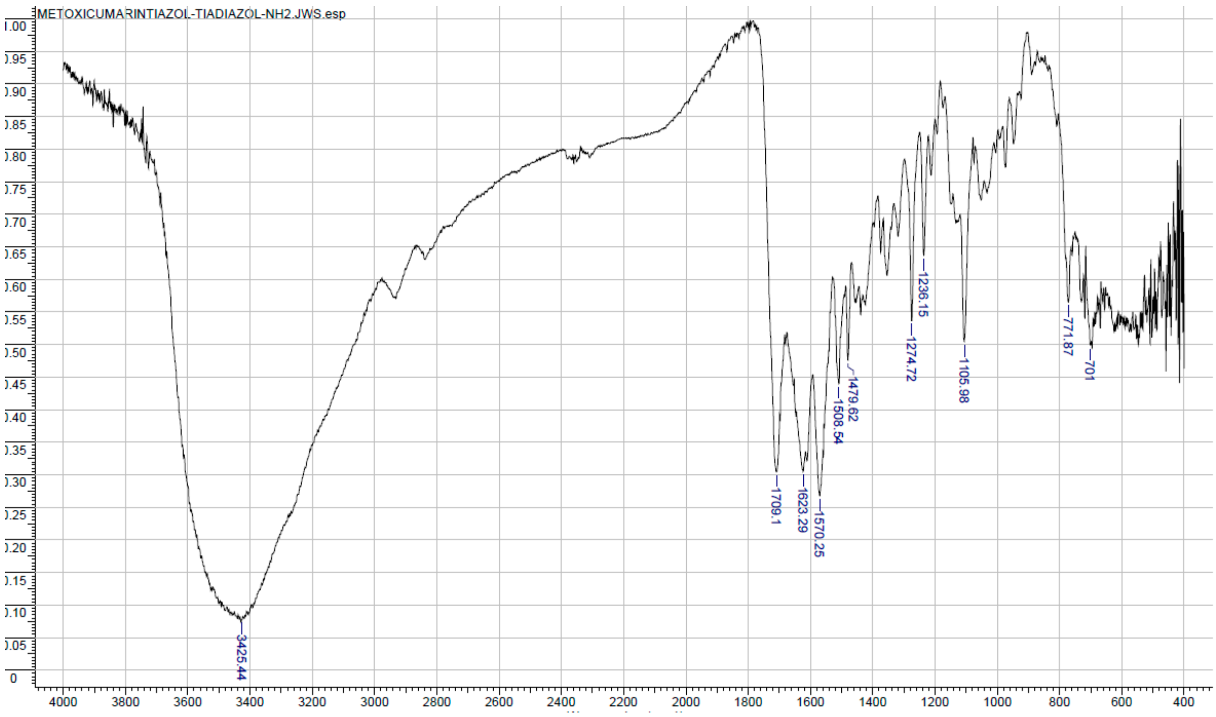

Figure S10. The IR spectrum for the compound **6d**.

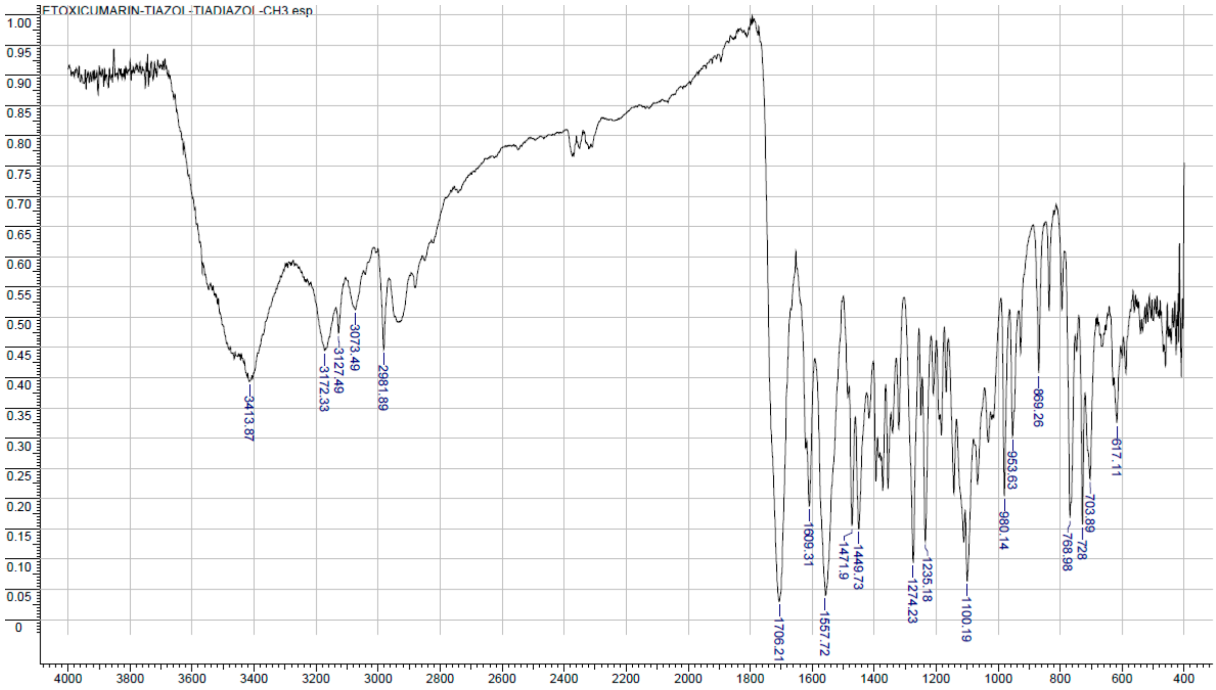

Figure S11. The IR spectrum for the compound **6e**.

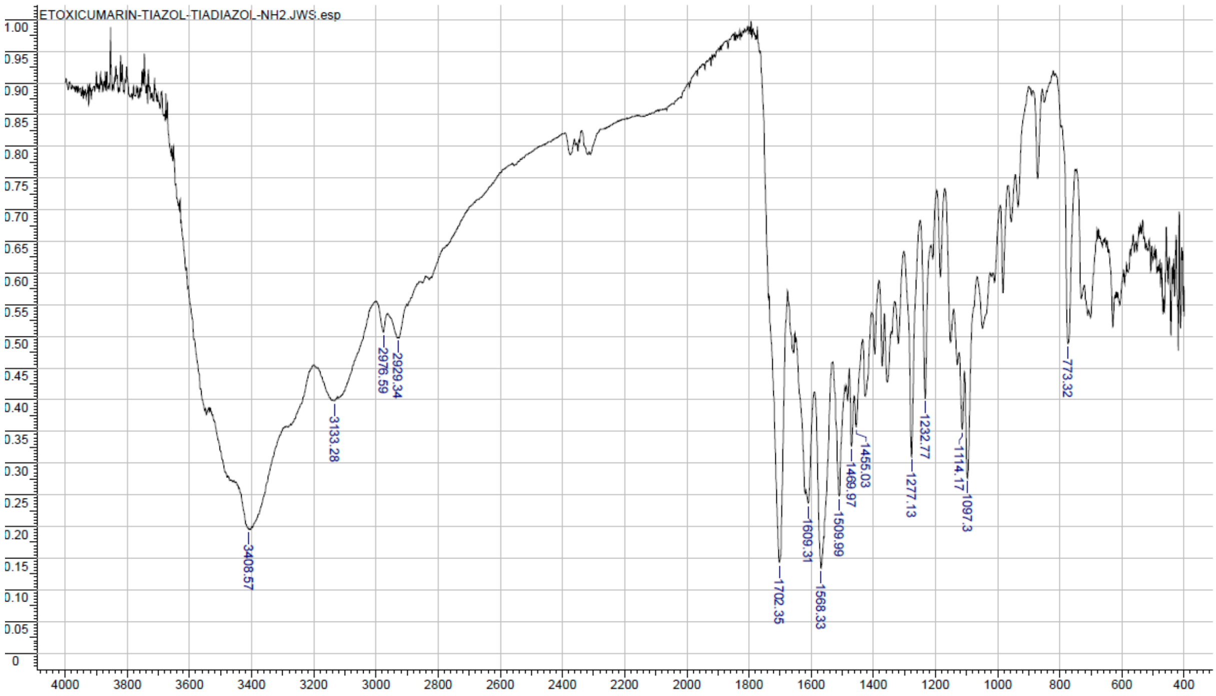

Figure S12. The IR spectrum for the compound **6f**.

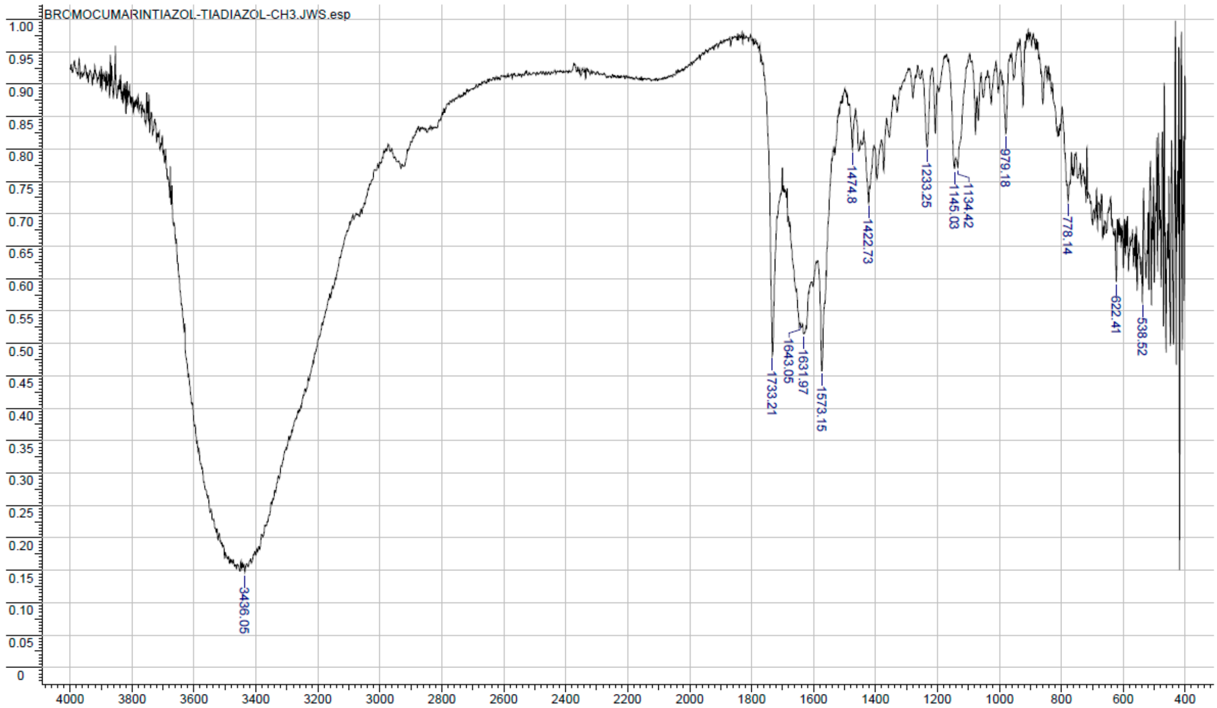

Figure S13. The IR spectrum for the compound **6g**.

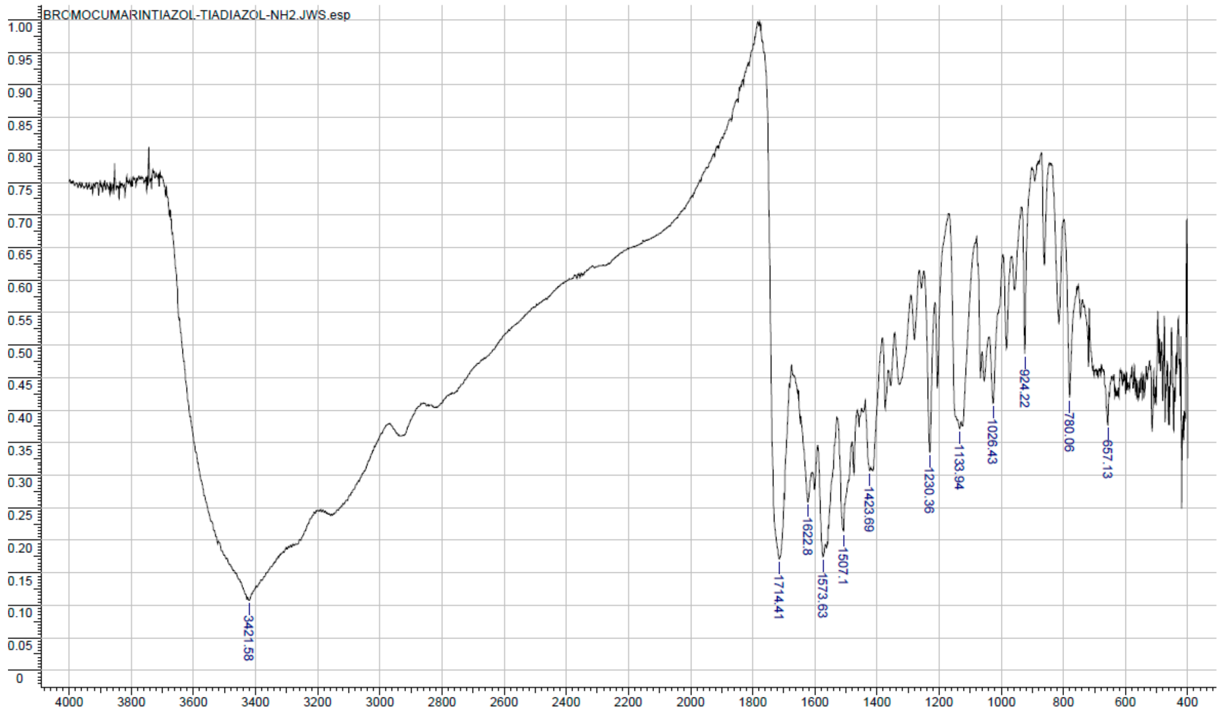

Figure S14. The IR spectrum for the compound **6h**.

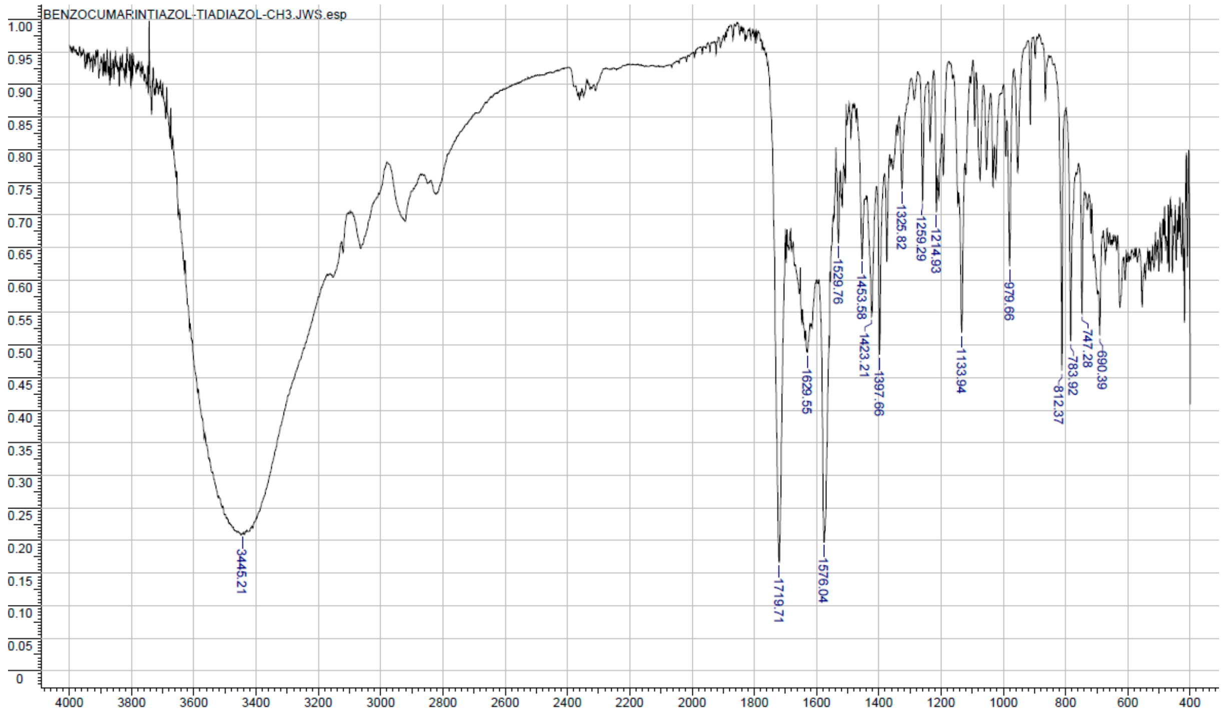

Figure S15. The IR spectrum for the compound **6i**.

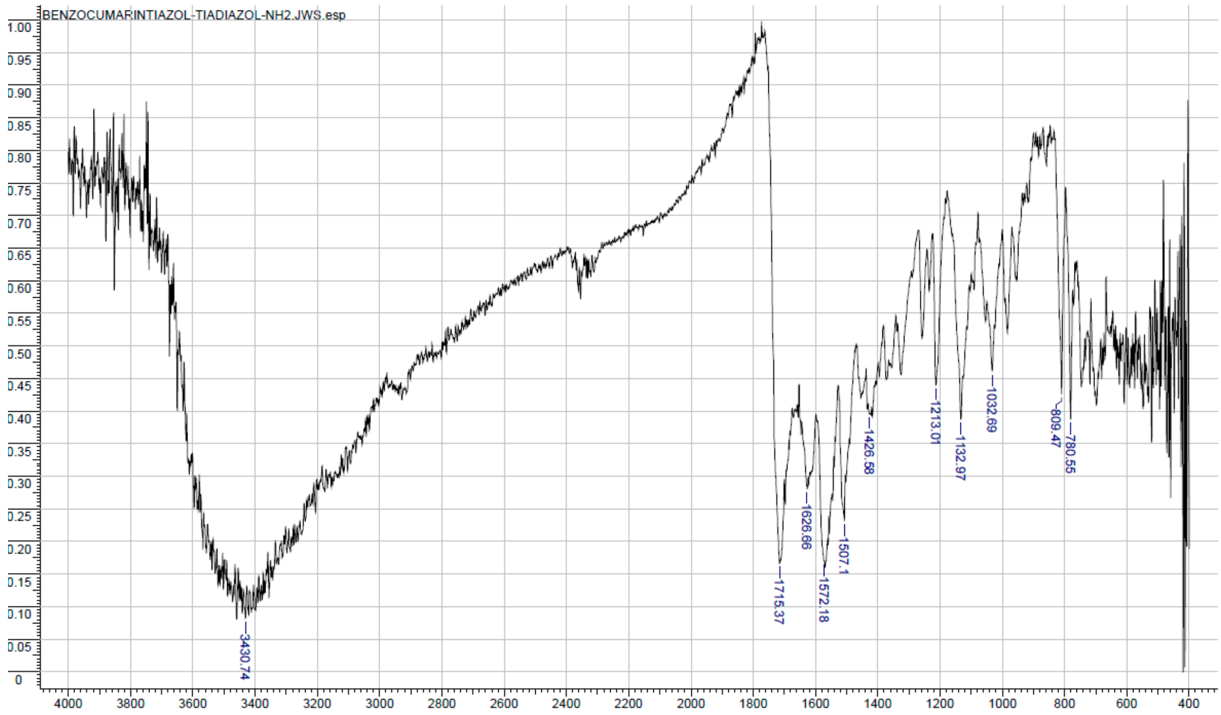

Figure S16. The IR spectrum for the compound **6j**.

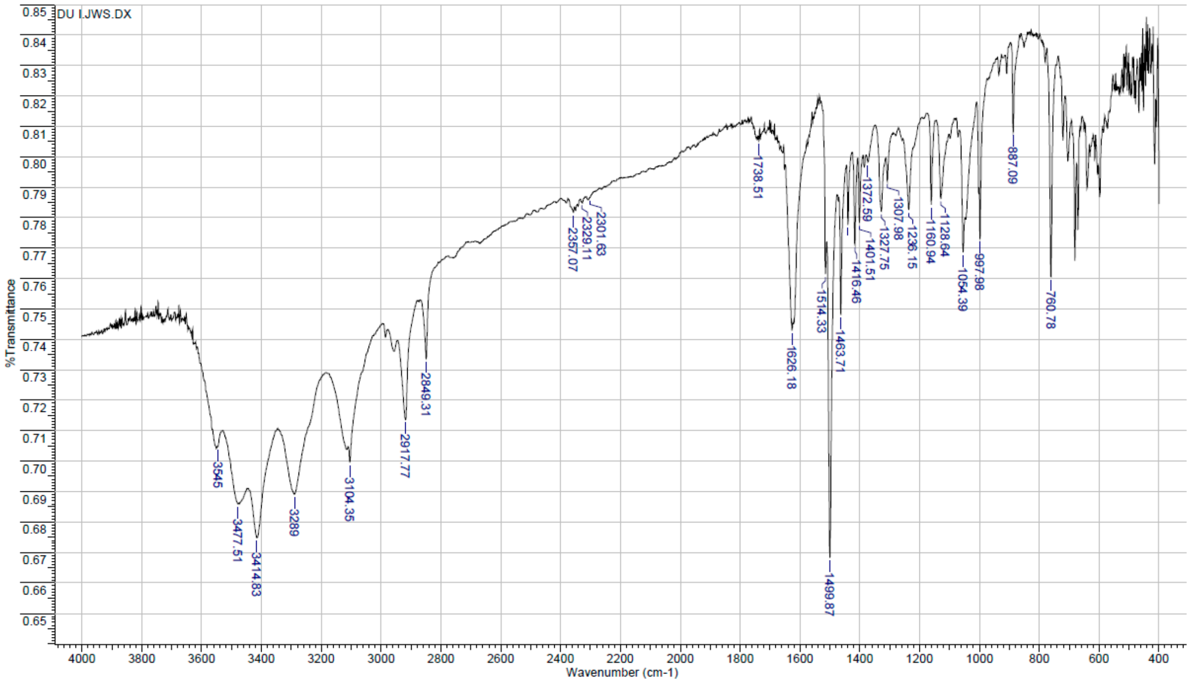

Figure S17. The IR spectrum for the compound **6k**.

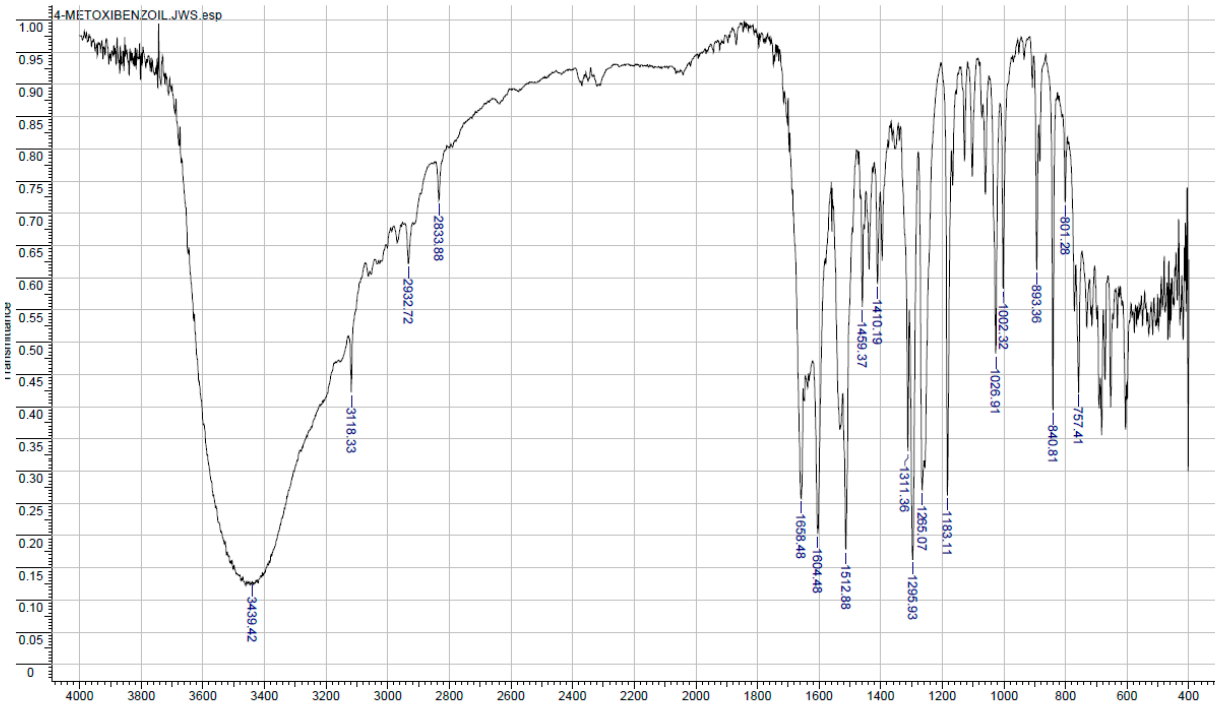

Figure S18. The IR spectrum for the compound **8a**.

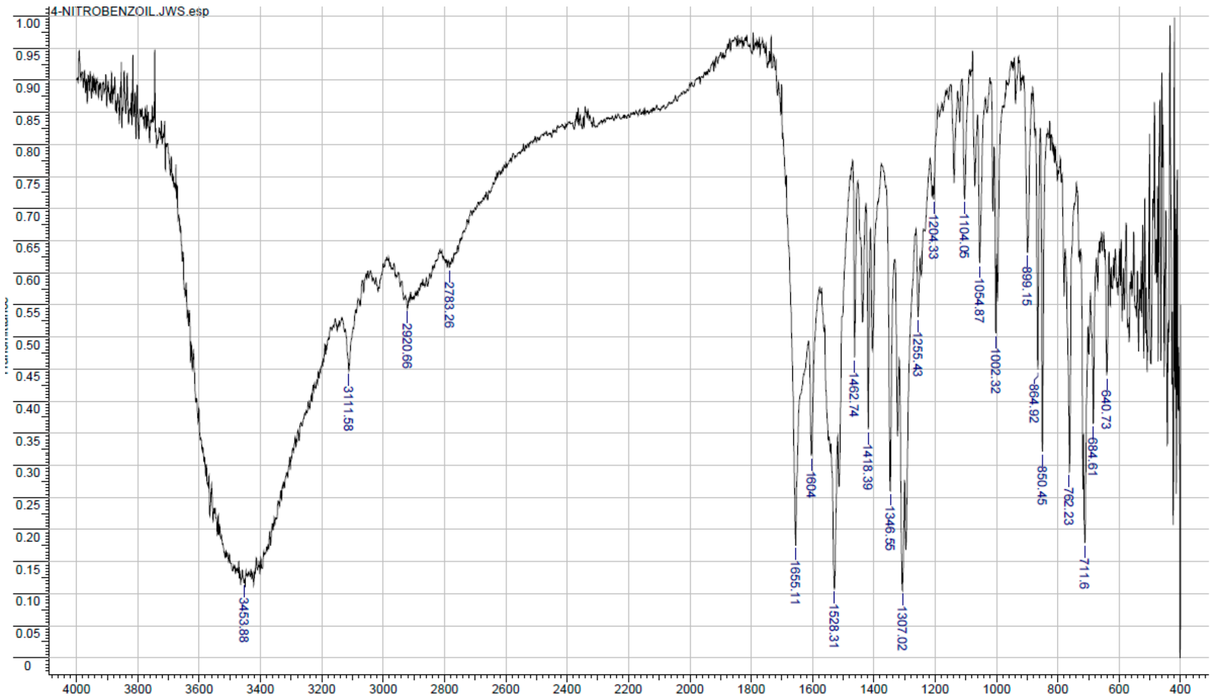

Figure S19. The IR spectrum for the compound **8b**.

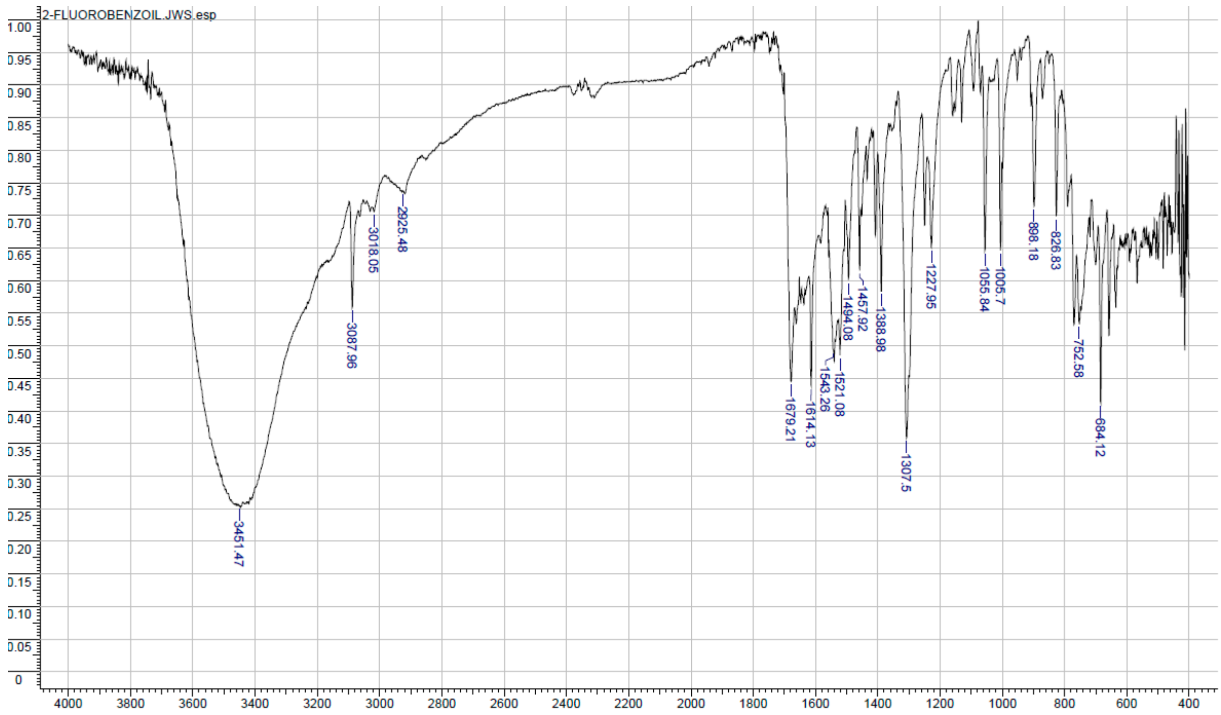

Figure S20. The IR spectrum for the compound **8c**.

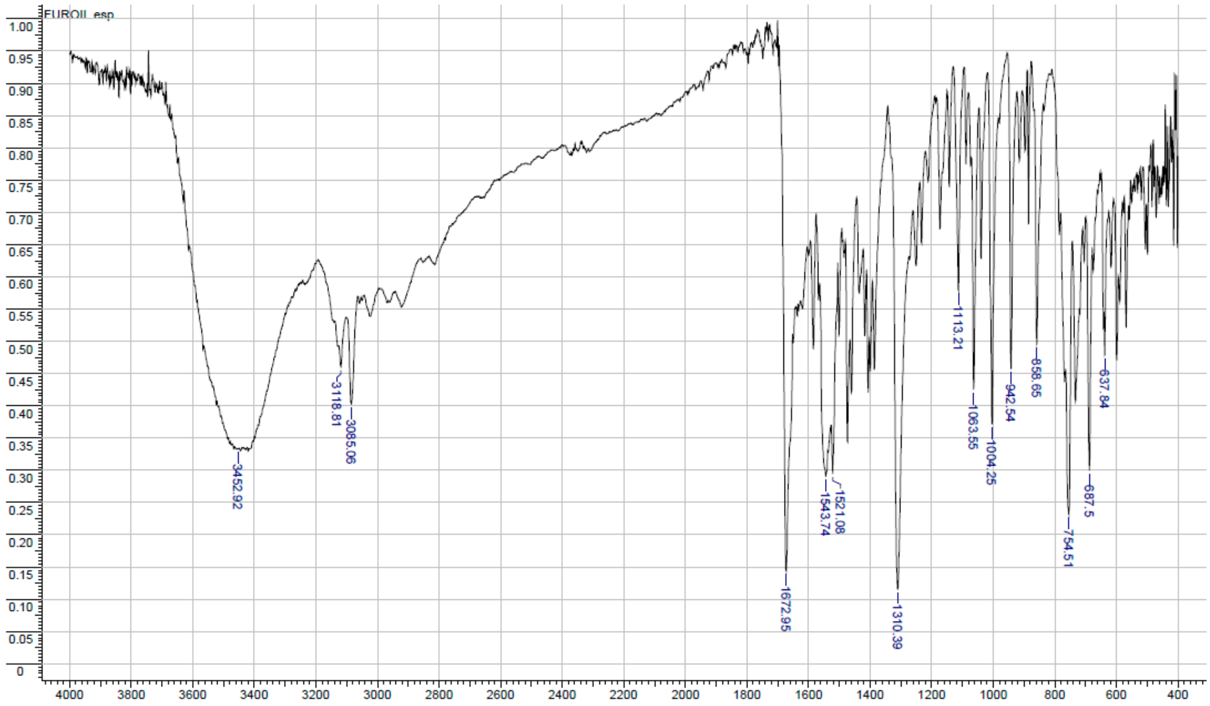

Figure S21. The IR spectrum for the compound **8d**.

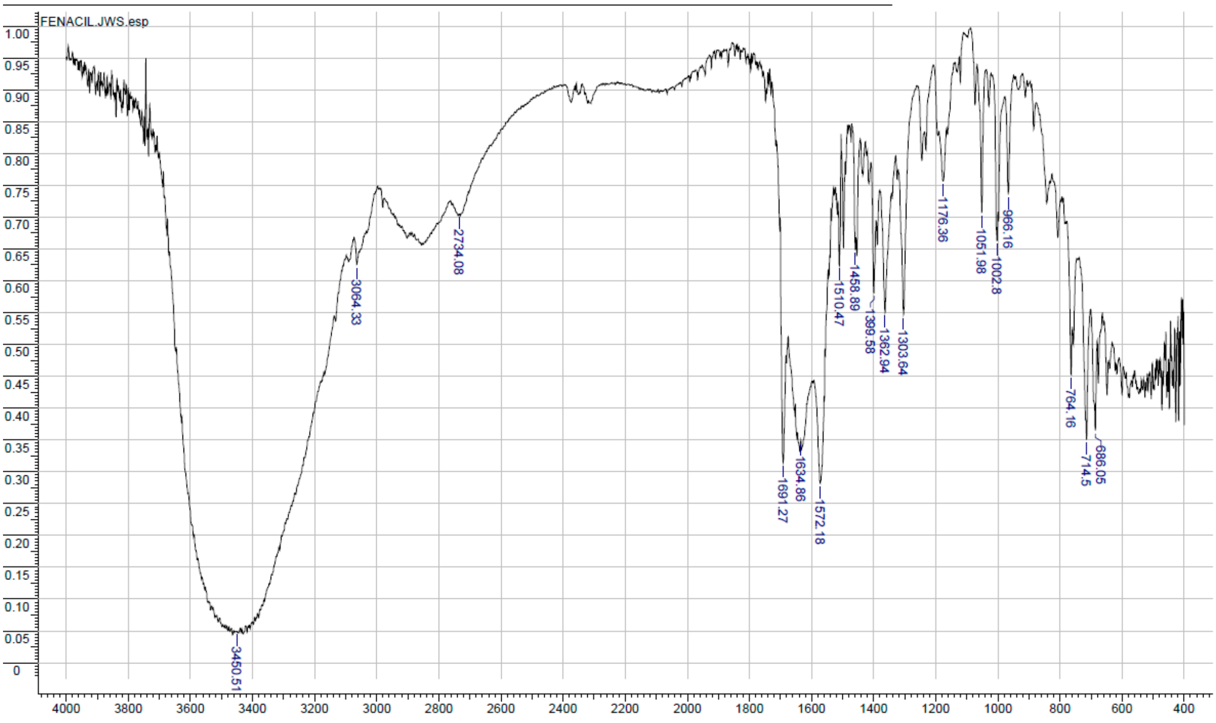

Figure S22. The IR spectrum for the compound **8e**.

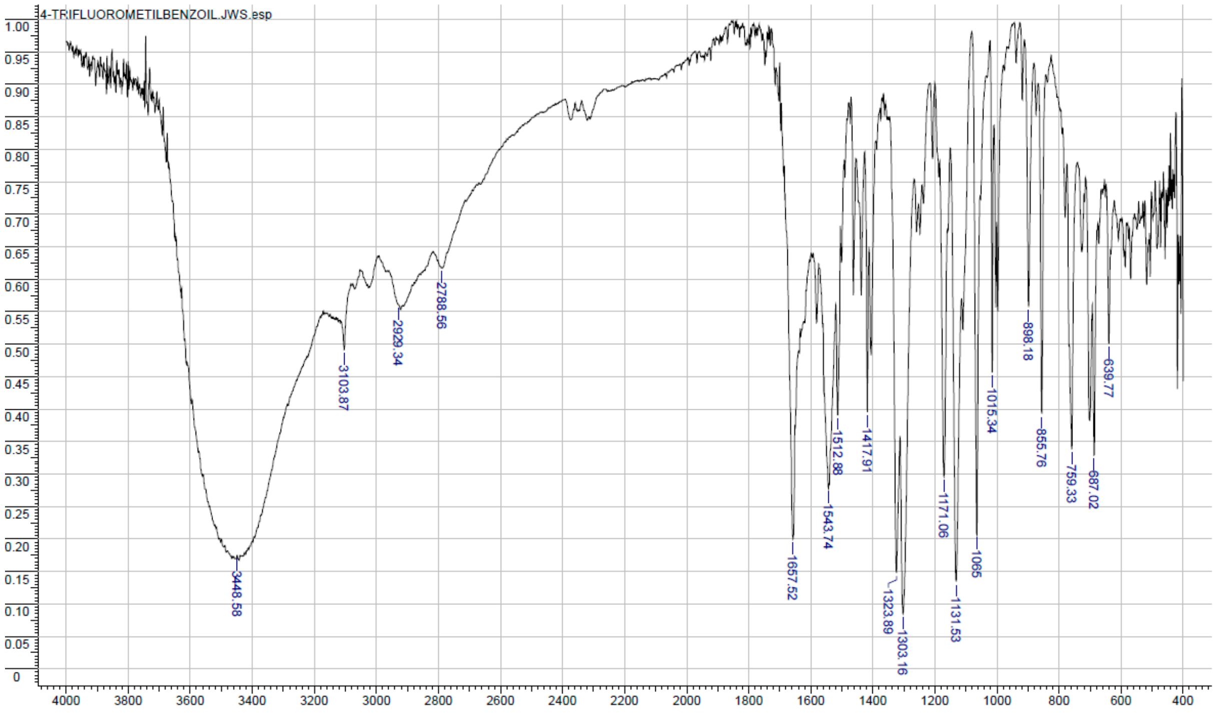

Figure S23. The IR spectrum for the compound **8f**.

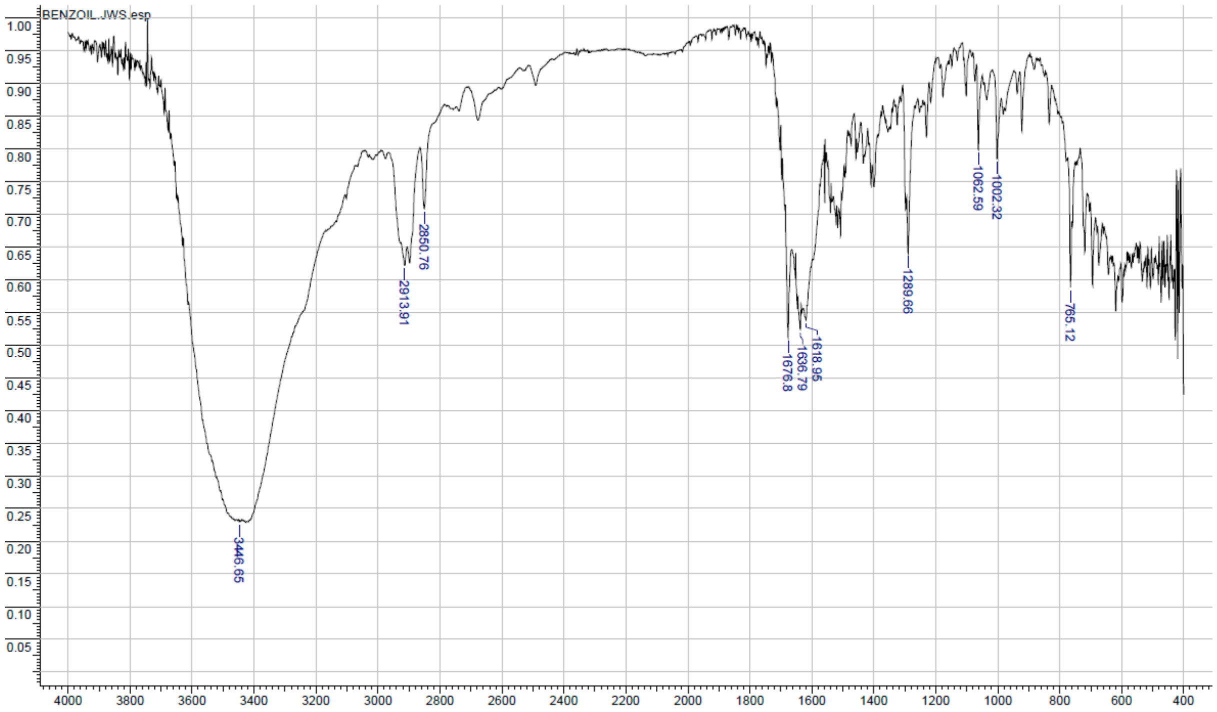

Figure S24. The IR spectrum for the compound **8g**.

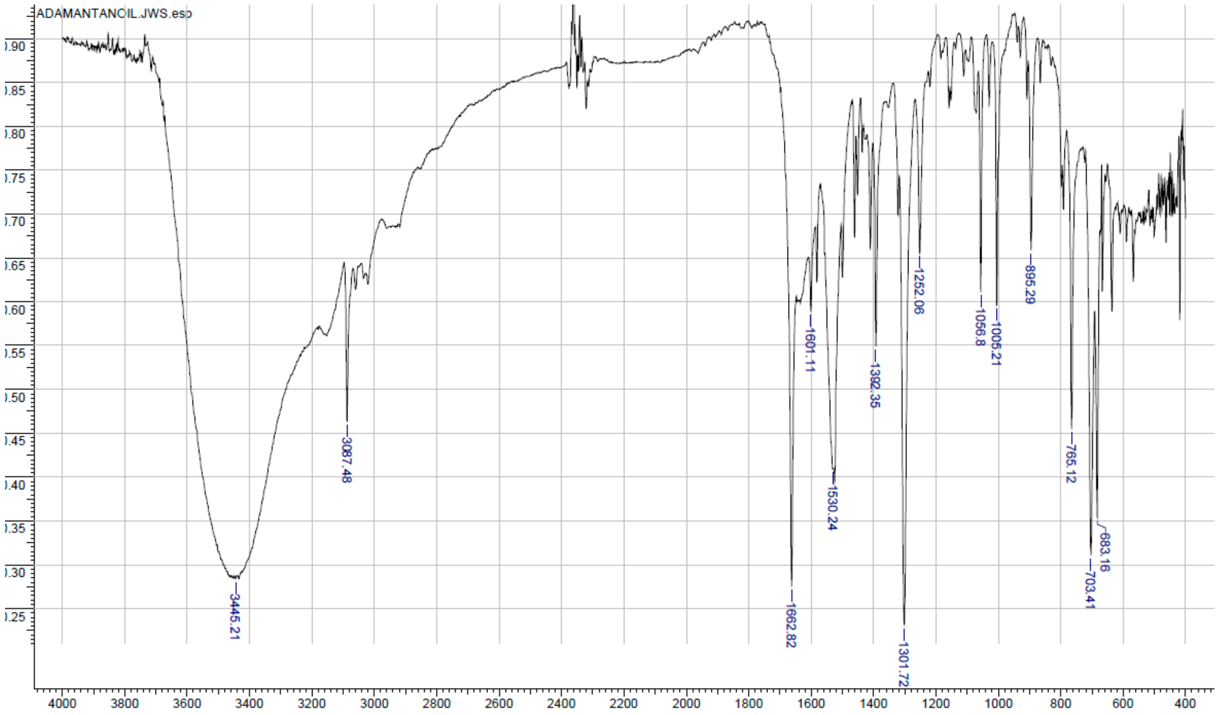

Figure S25. The IR spectrum for the compound **8h**.

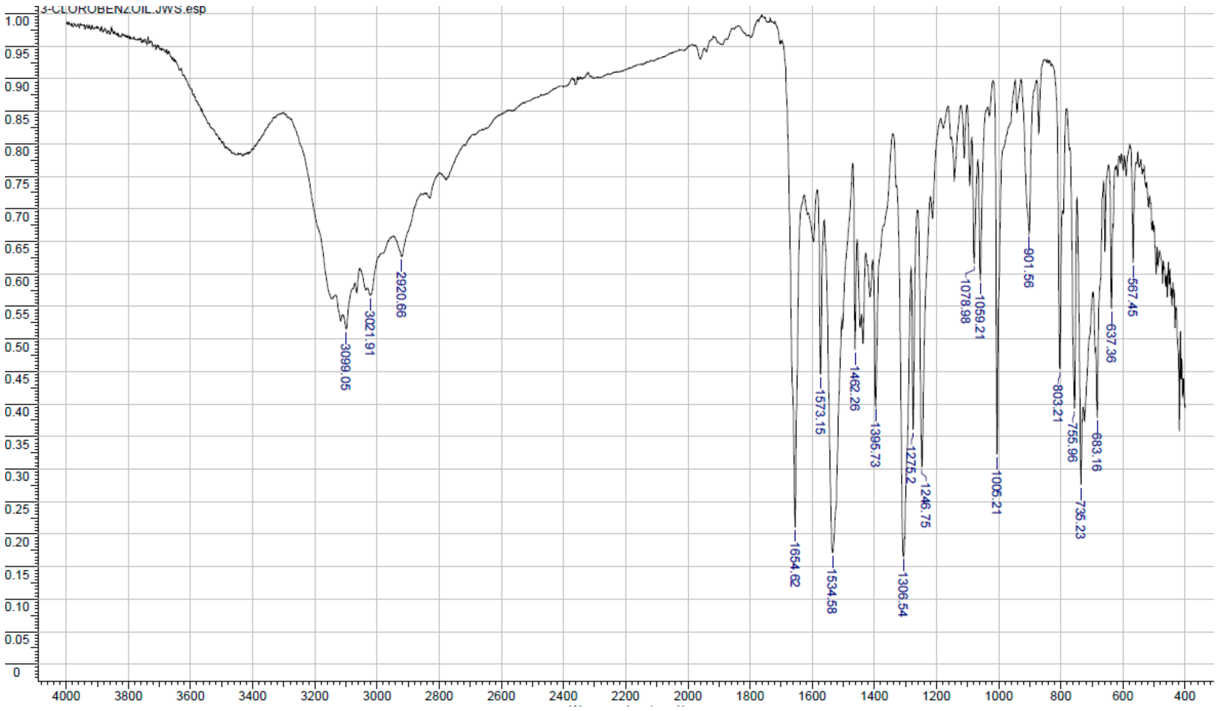

Figure S26. The IR spectrum for the compound **8i**.

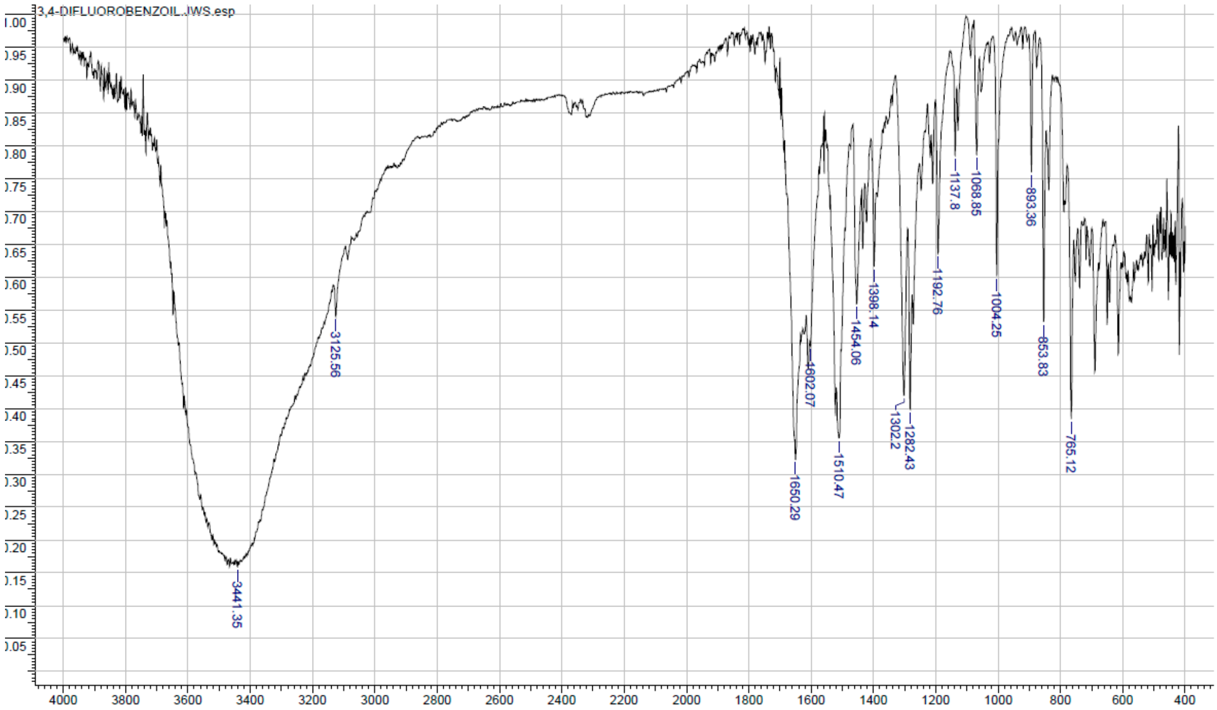

Figure S27. The IR spectrum for the compound **8j**.

S1.2. MS Spectra

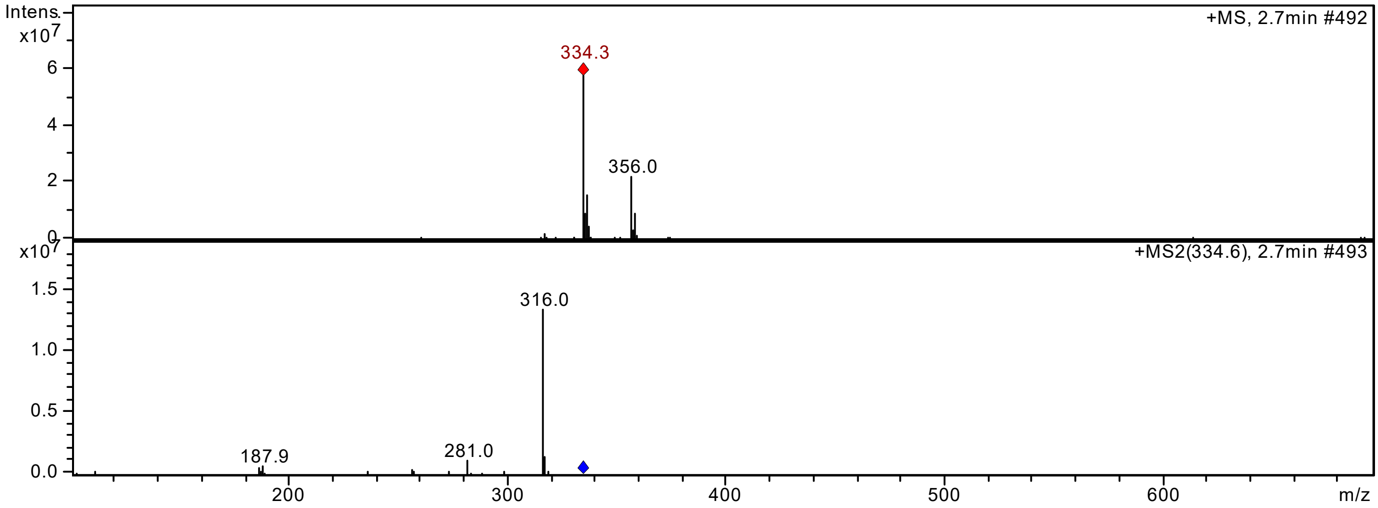

Figure S28. The MS spectrum for the compound **4a**.

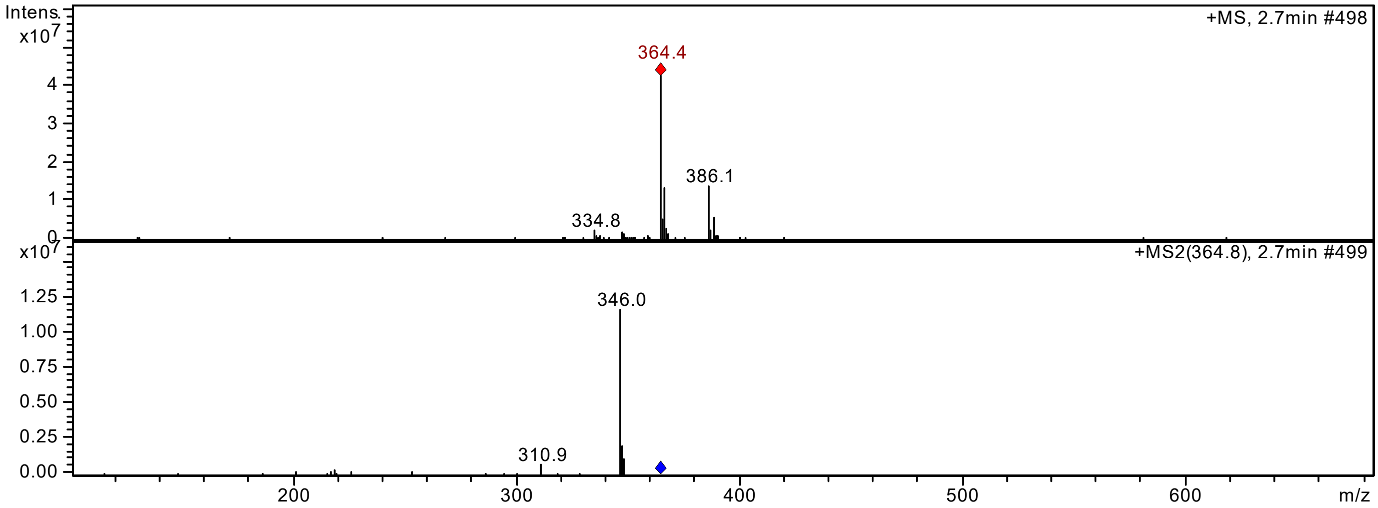

Figure S29. The MS spectrum for the compound **4b**.

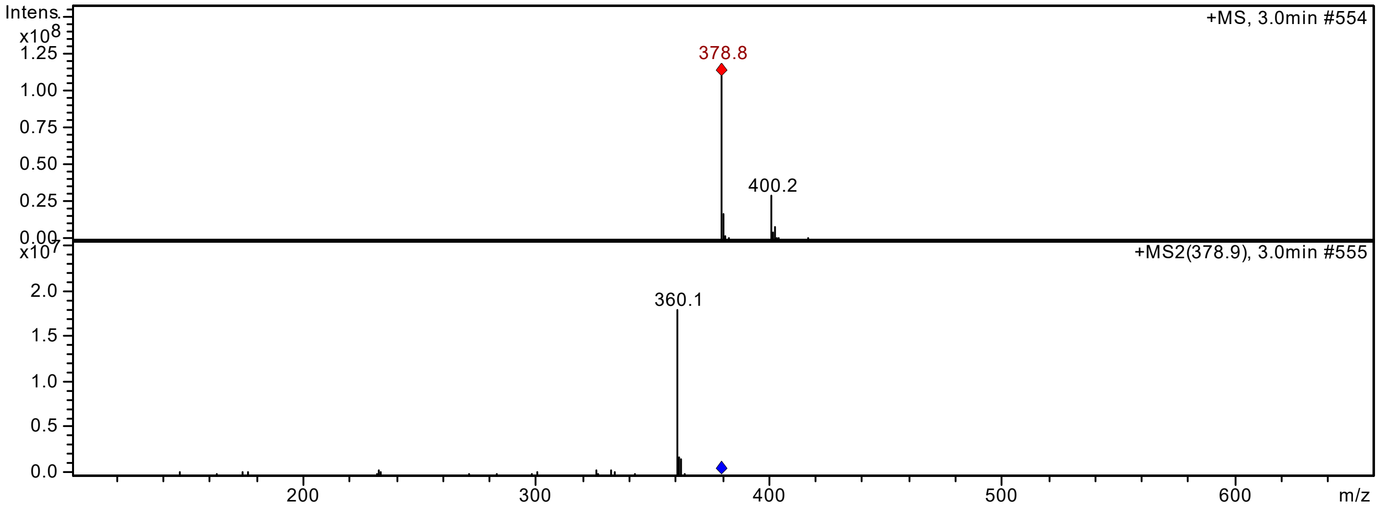

Figure S30. The MS spectrum for the compound **4c**.

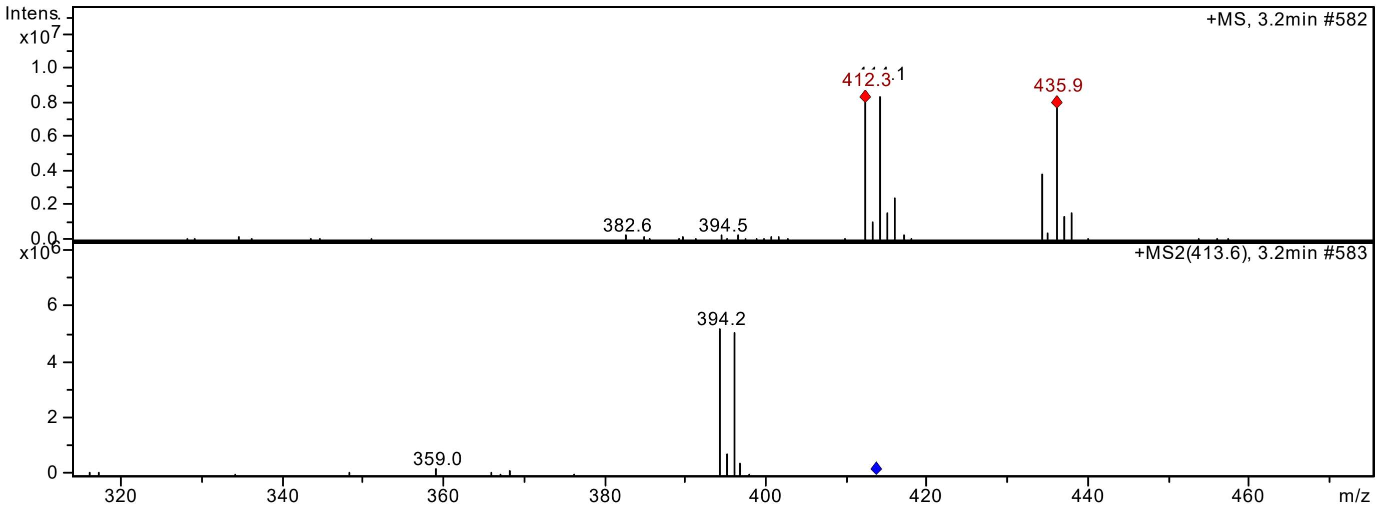

Figure S31. The MS spectrum for the compound **4d**.

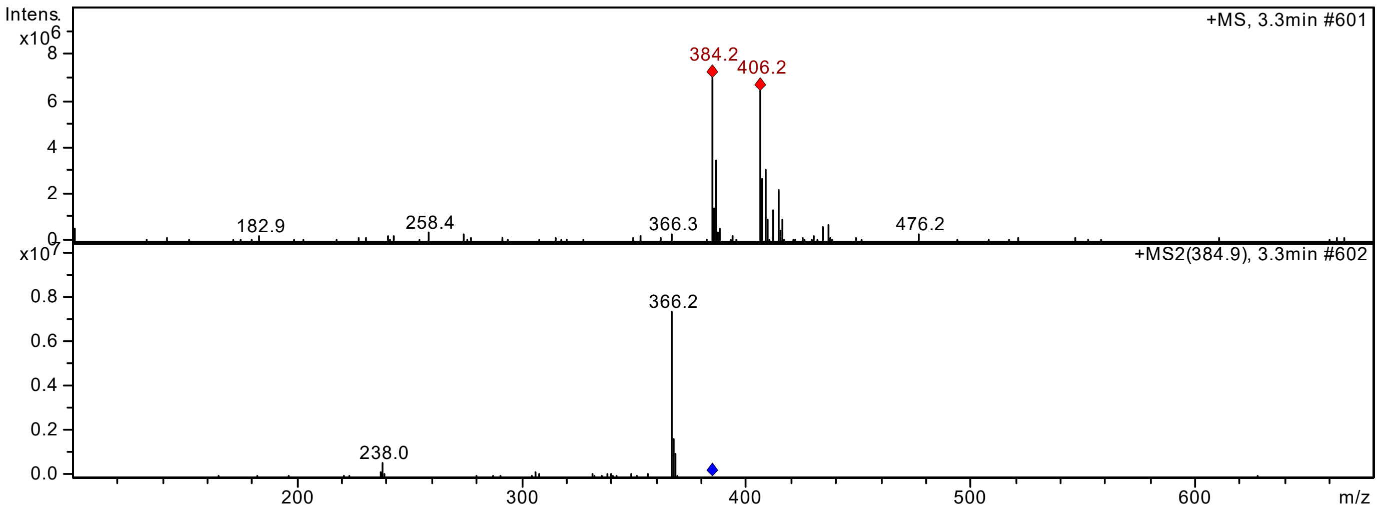

Figure S32. The MS spectrum for the compound **4e**.

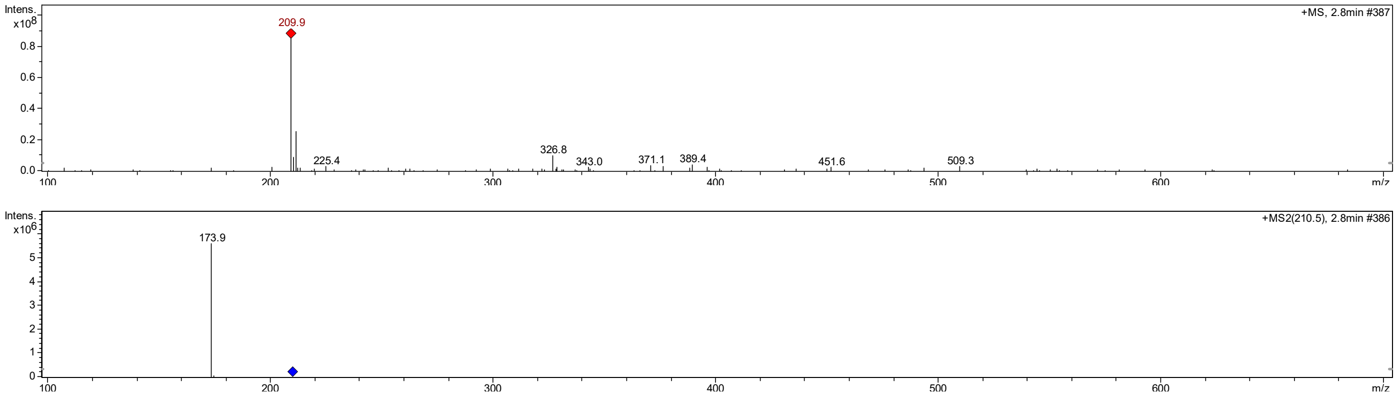

Figure S33. The MS spectrum for the compound **4f**.

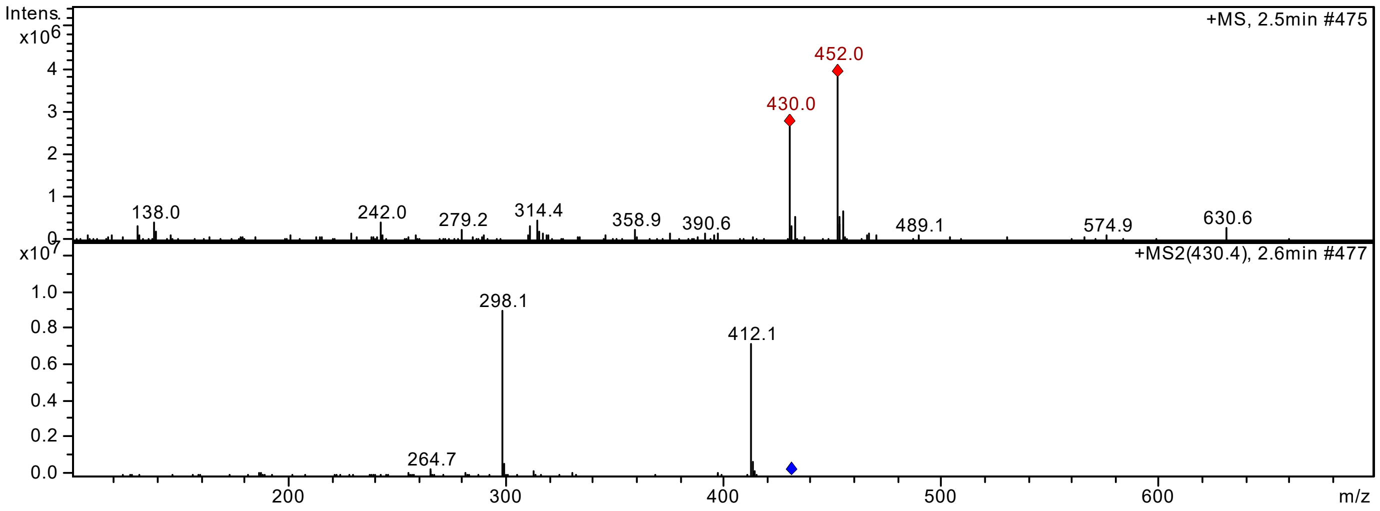

Figure S34. The MS spectrum for the compound **6a**.

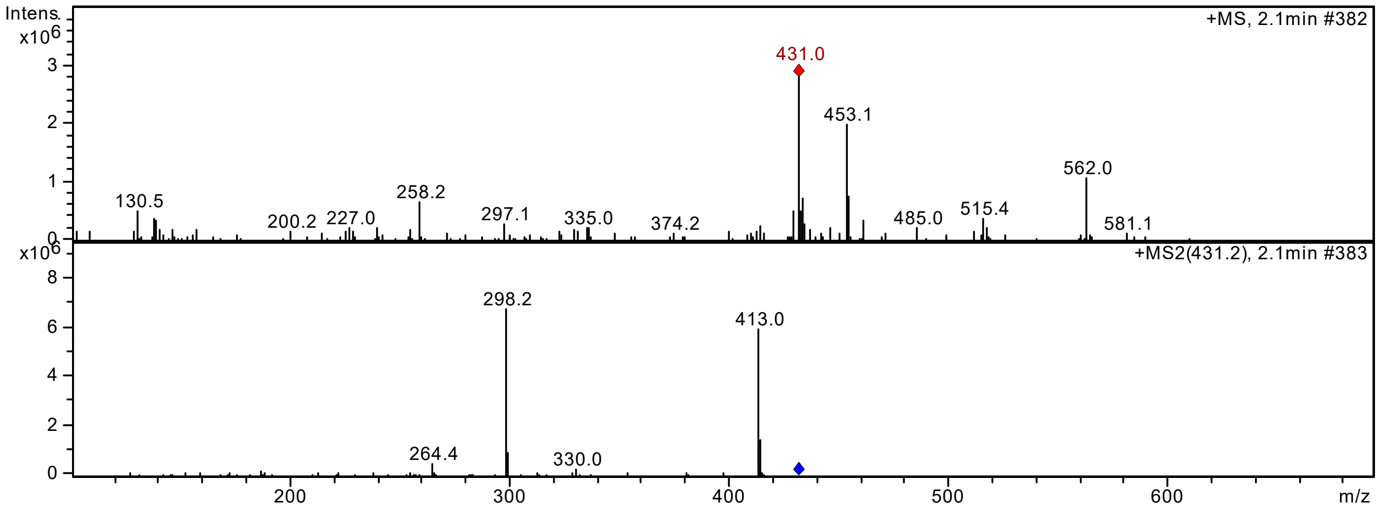

Figure S35. The MS spectrum for the compound **6b**.

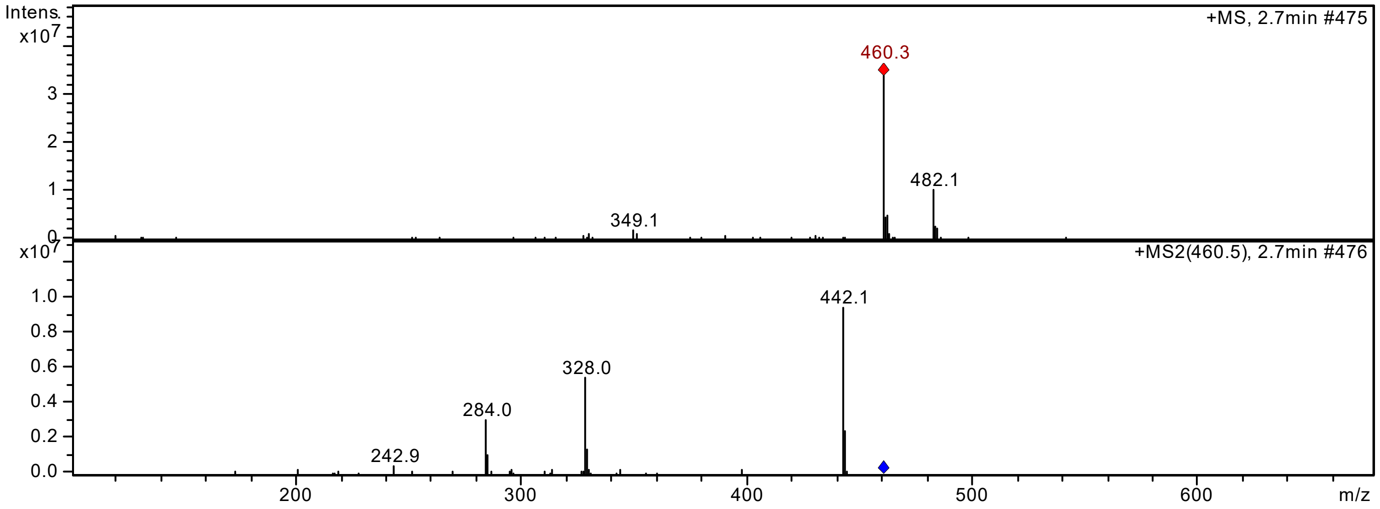

Figure S36. The MS spectrum for the compound **6c**.

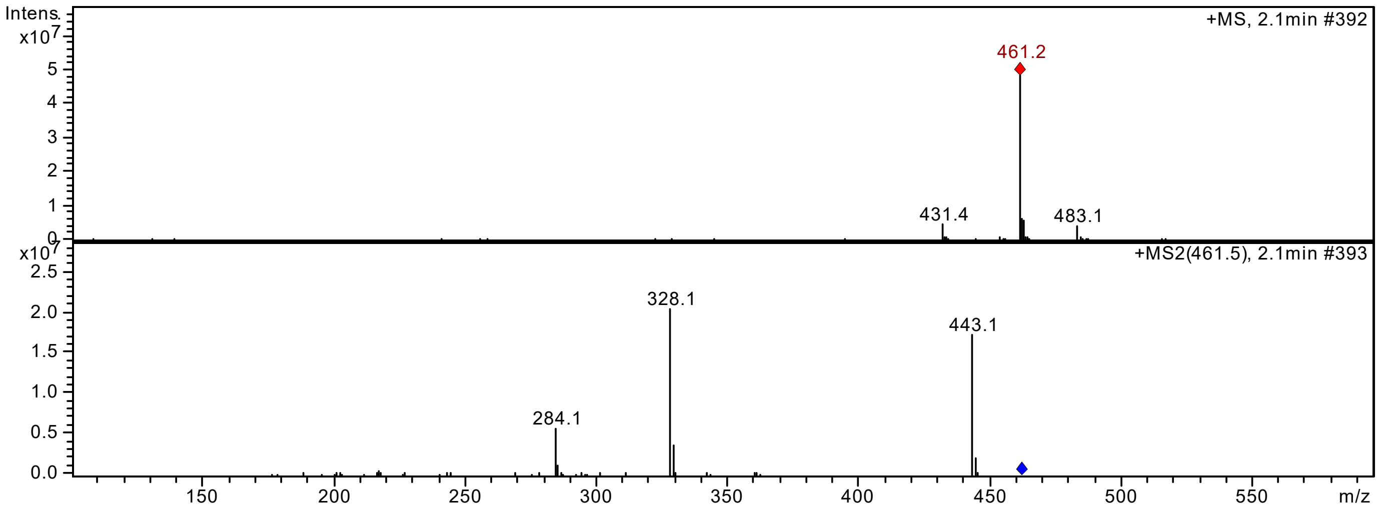

Figure S37. The MS spectrum for the compound **6d**.

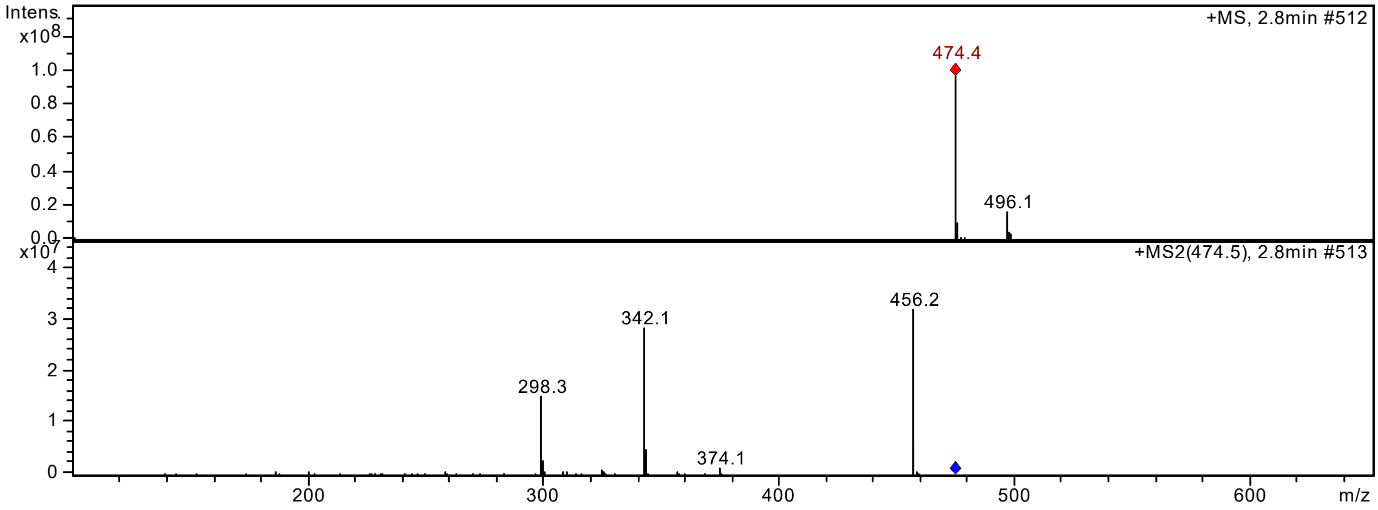

Figure S38. The MS spectrum for the compound **6e**.

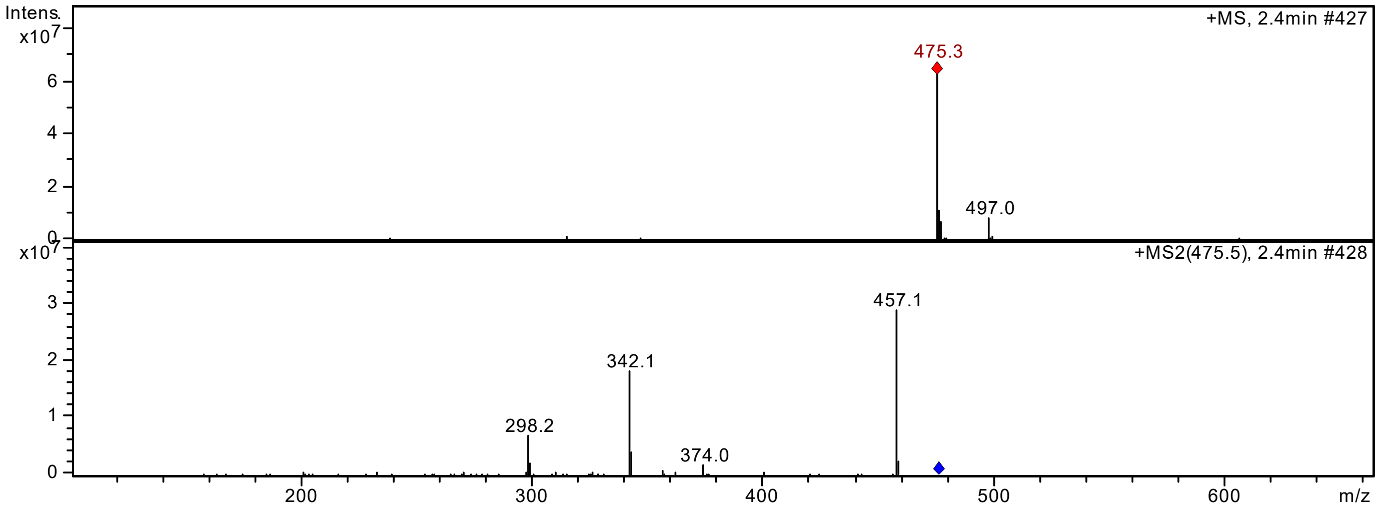

Figure S39. The MS spectrum for the compound **6f**.

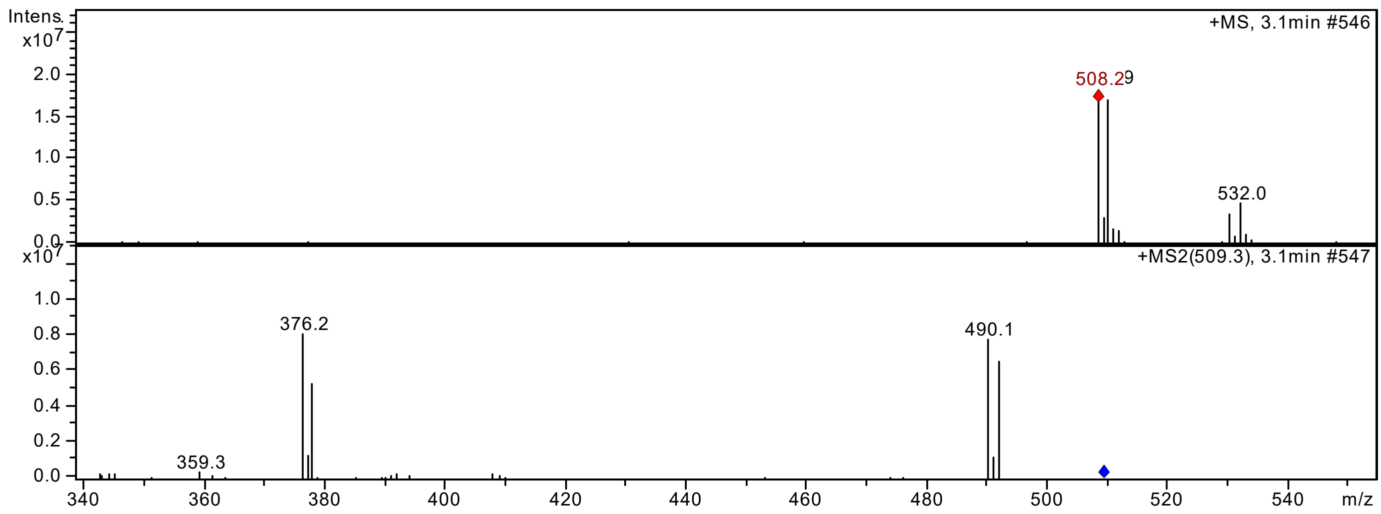

Figure S40. The MS spectrum for the compound **6g**.

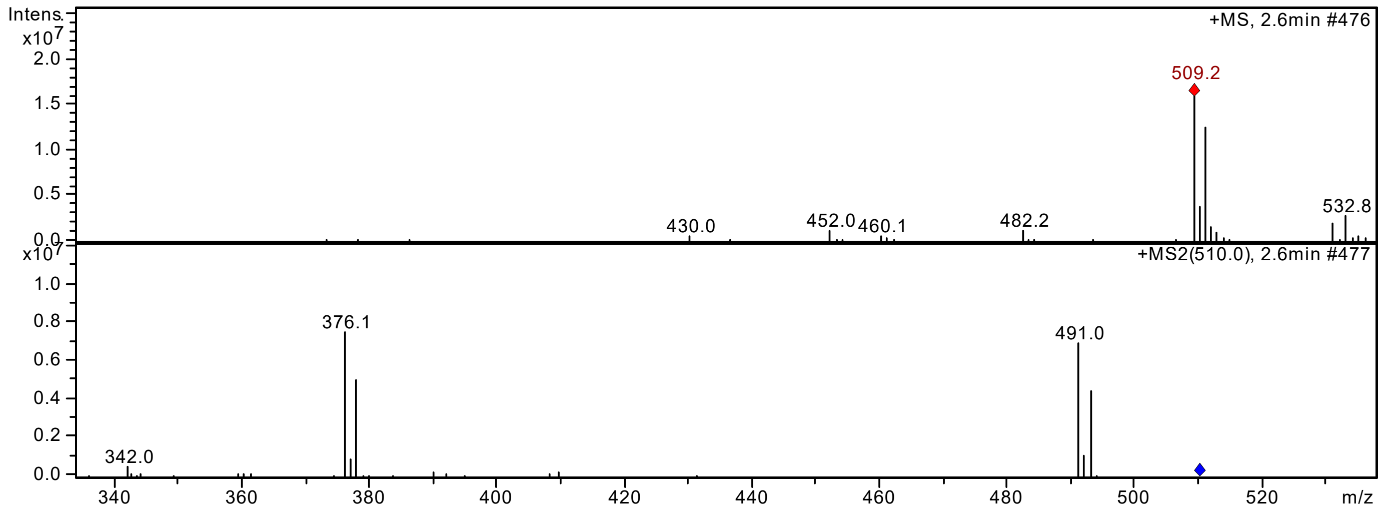

Figure S41. The MS spectrum for the compound **6h**.

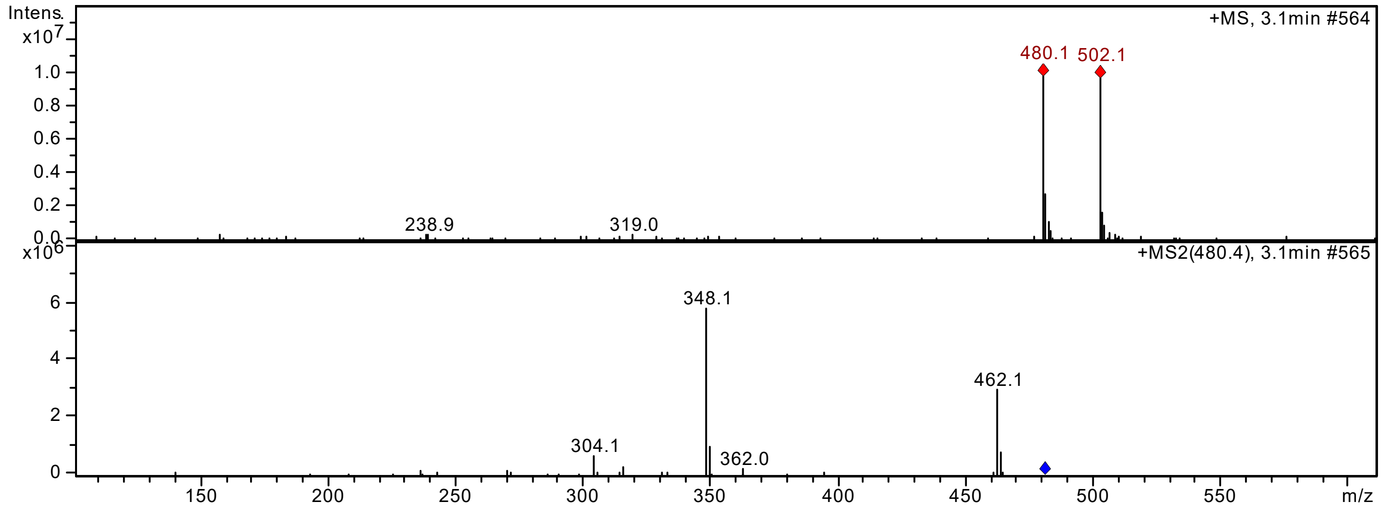

Figure S42. The MS spectrum for the compound **6i**.

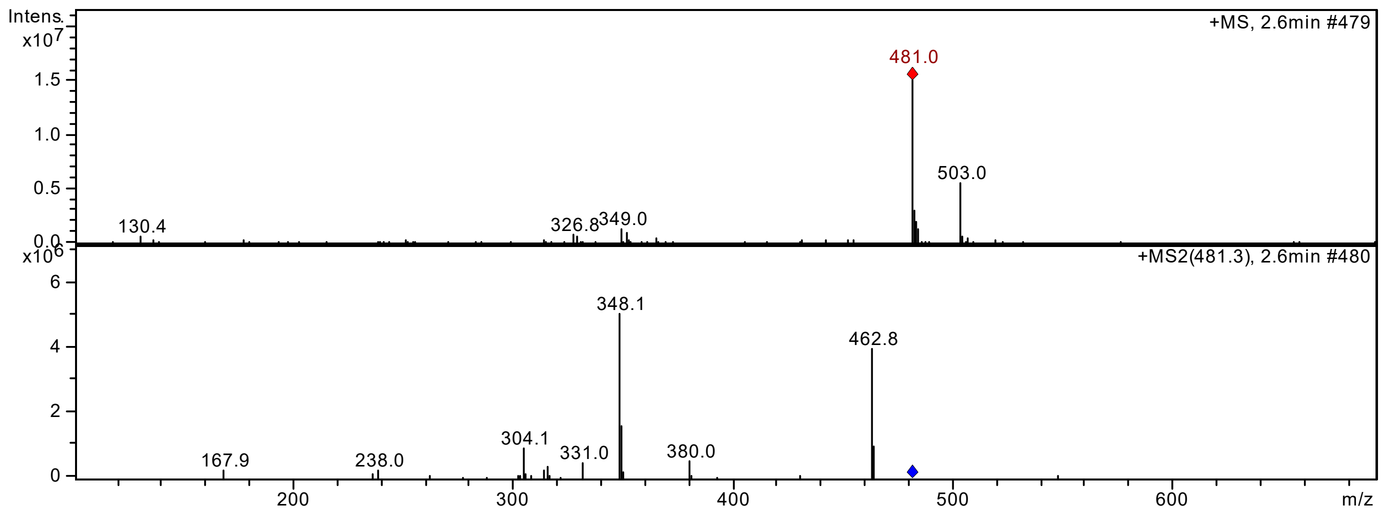

Figure S43. The MS spectrum for the compound **6j**.

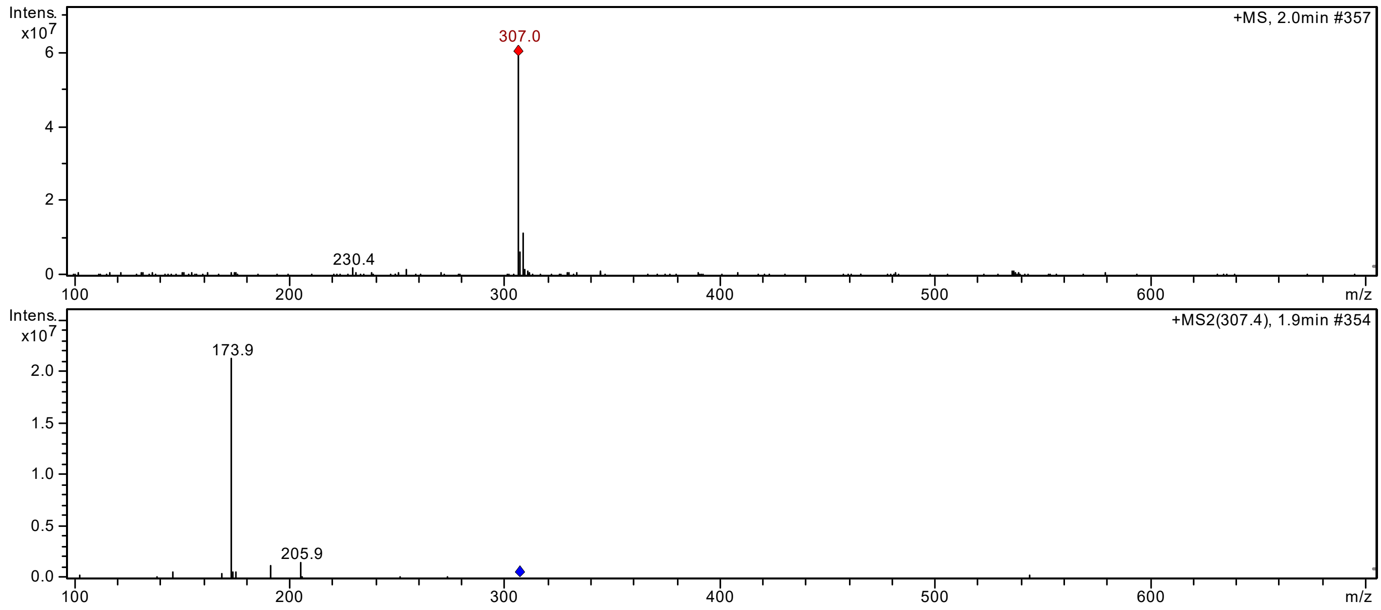

Figure S44. The MS spectrum for the compound **6k**.

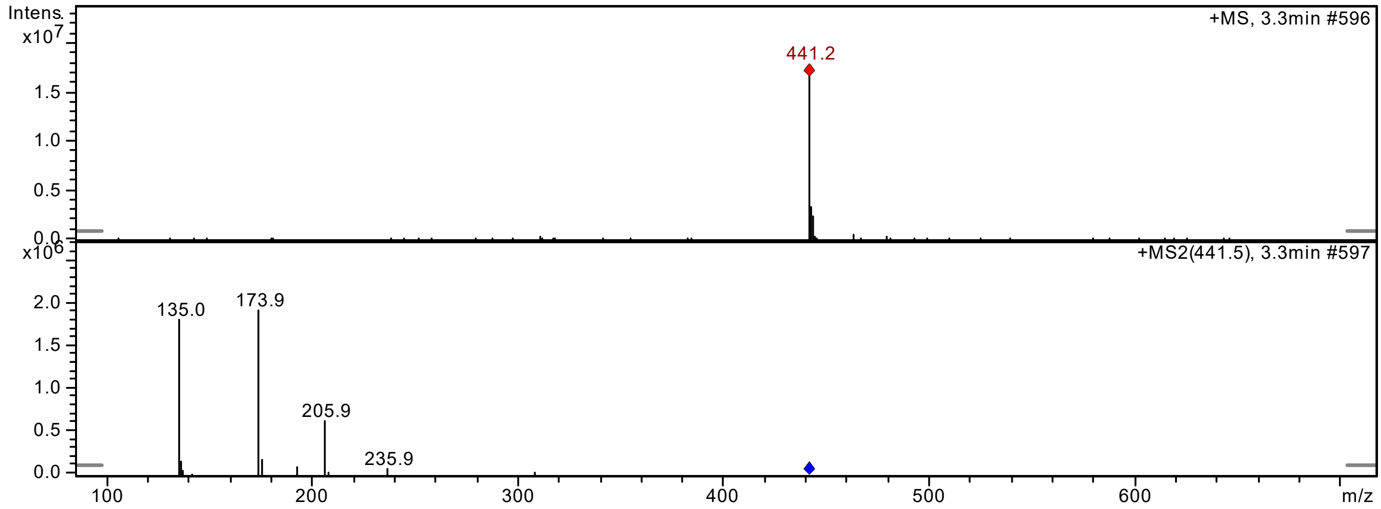

Figure S45. The MS spectrum for the compound **8a**.

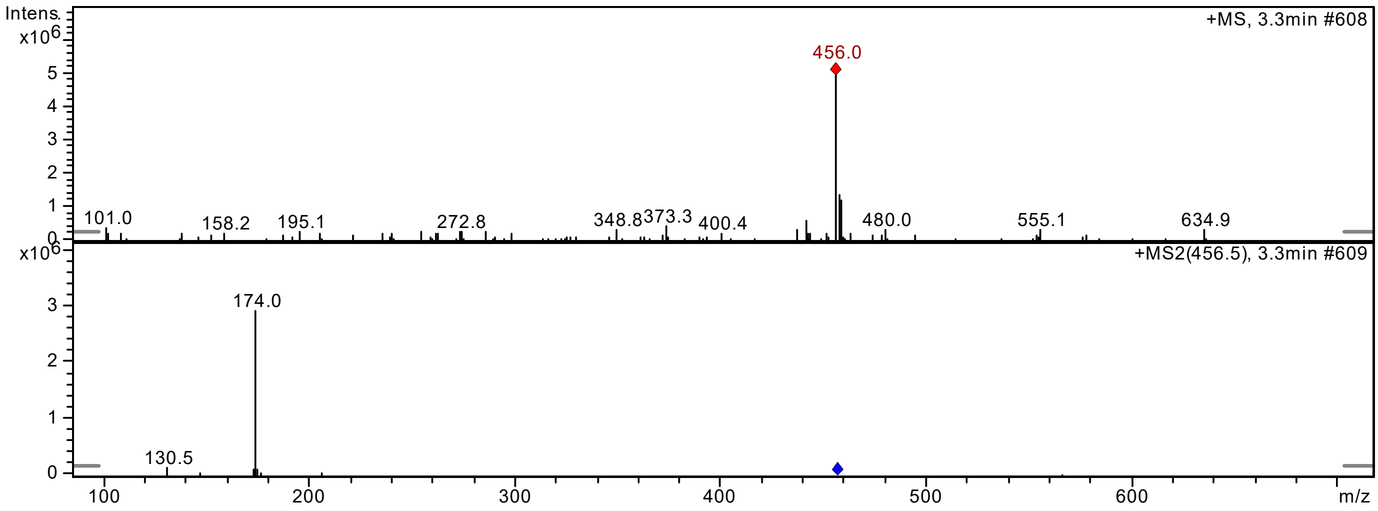

Figure S46. The MS spectrum for the compound **8b**.

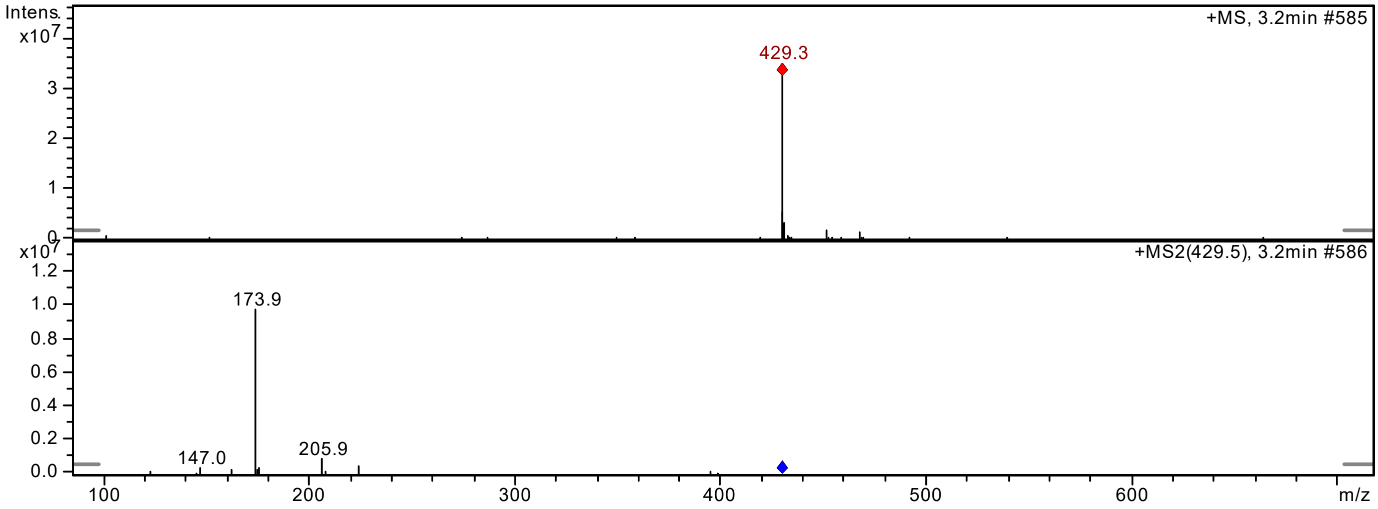

Figure S47. The MS spectrum for the compound **8c**.

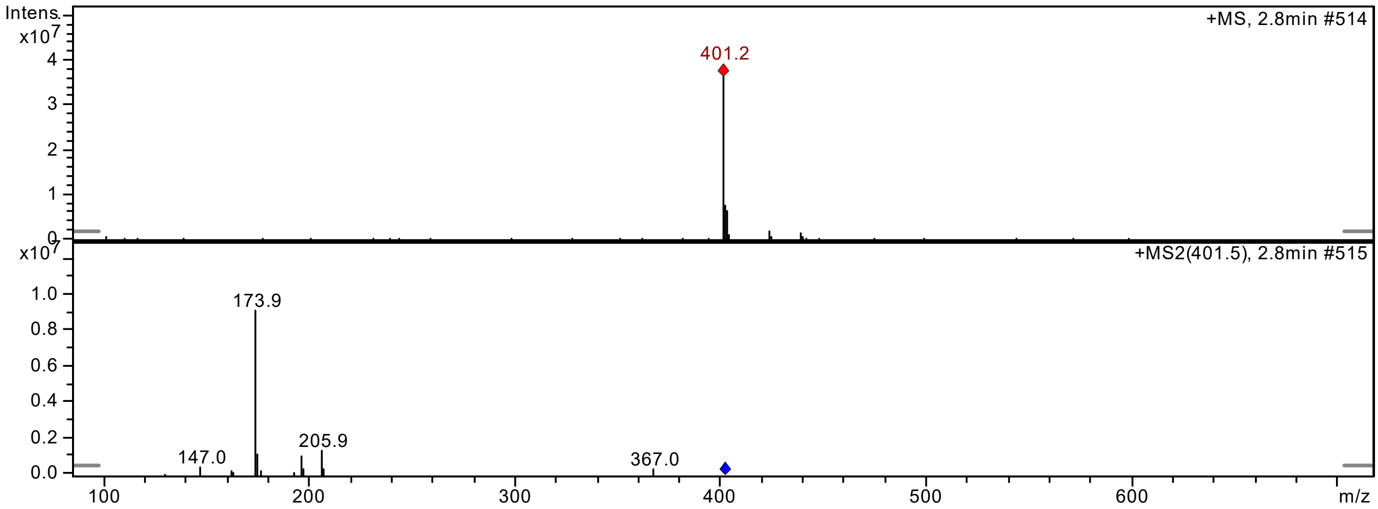

Figure S48. The MS spectrum for the compound **8d**.

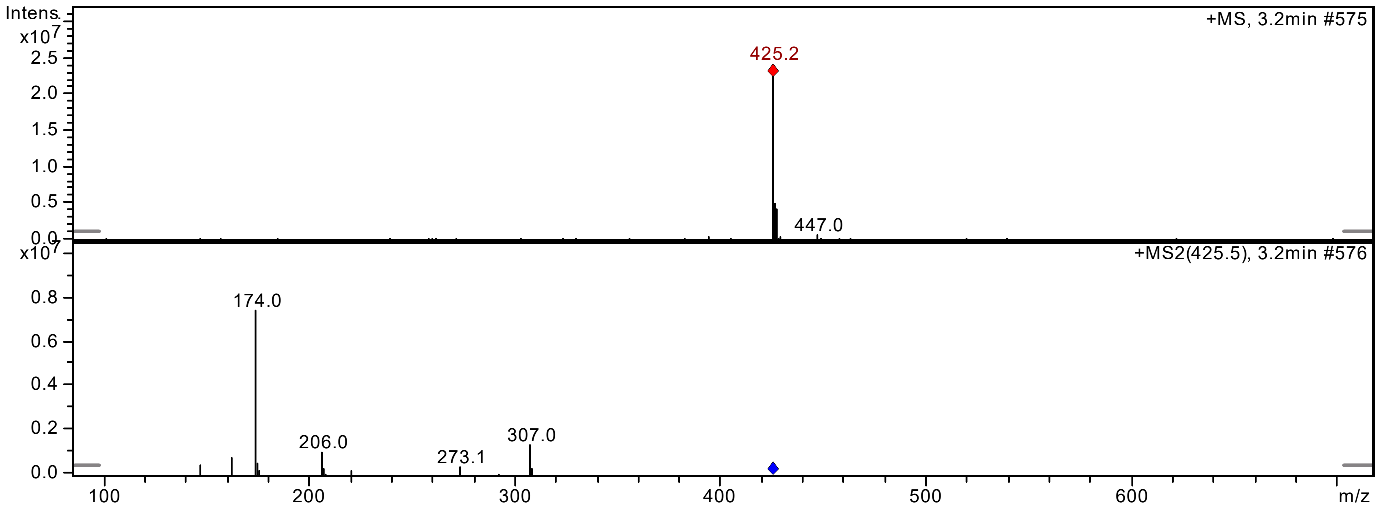

Figure S49. The MS spectrum for the compound **8e**.

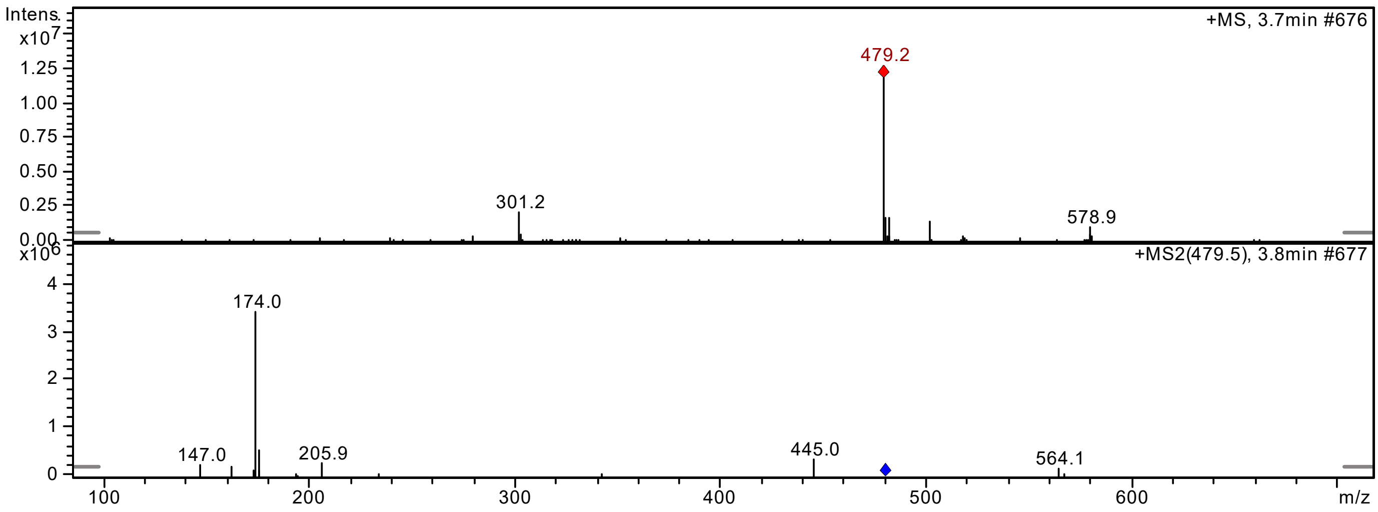

Figure S50. The MS spectrum for the compound **8f**.

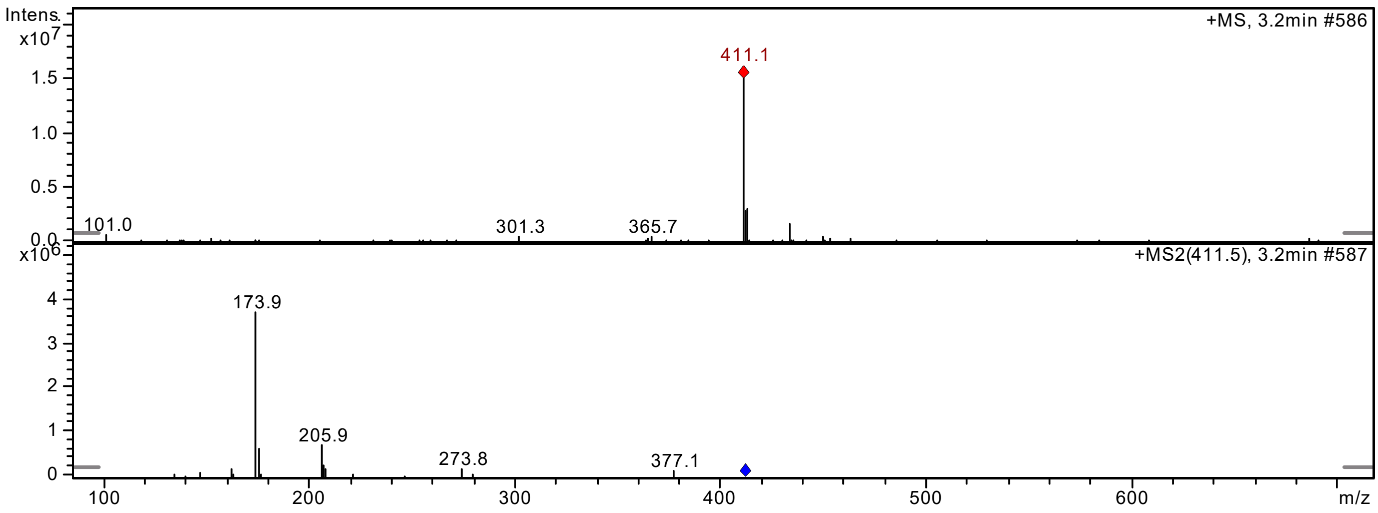

Figure S51. The MS spectrum for the compound **8g**.

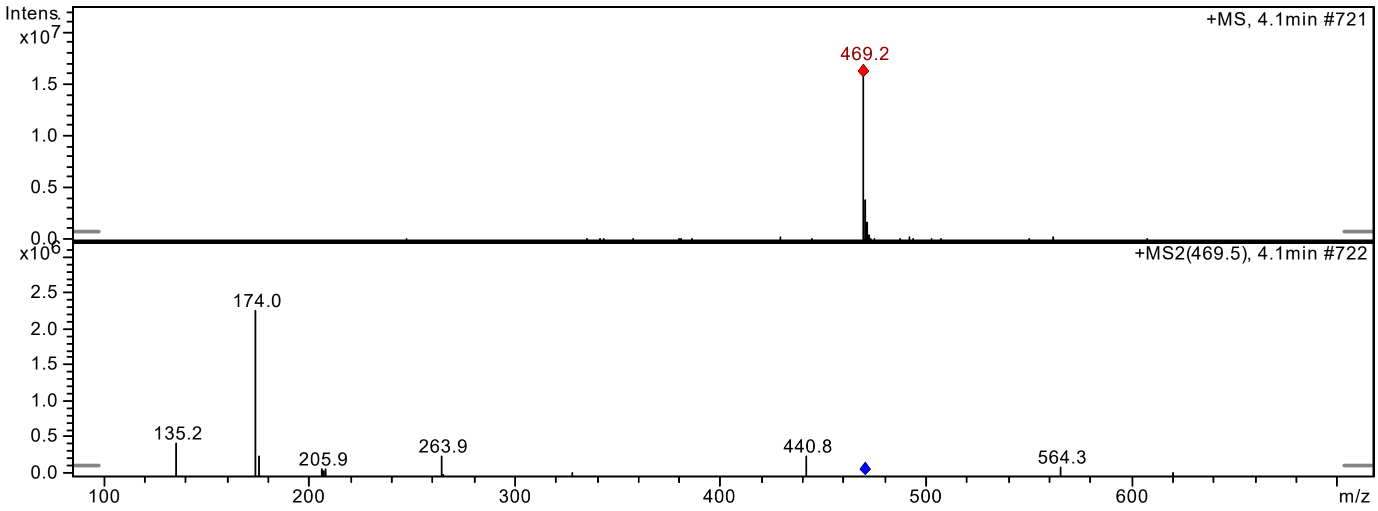

Figure S52. The MS spectrum for the compound **8h**.

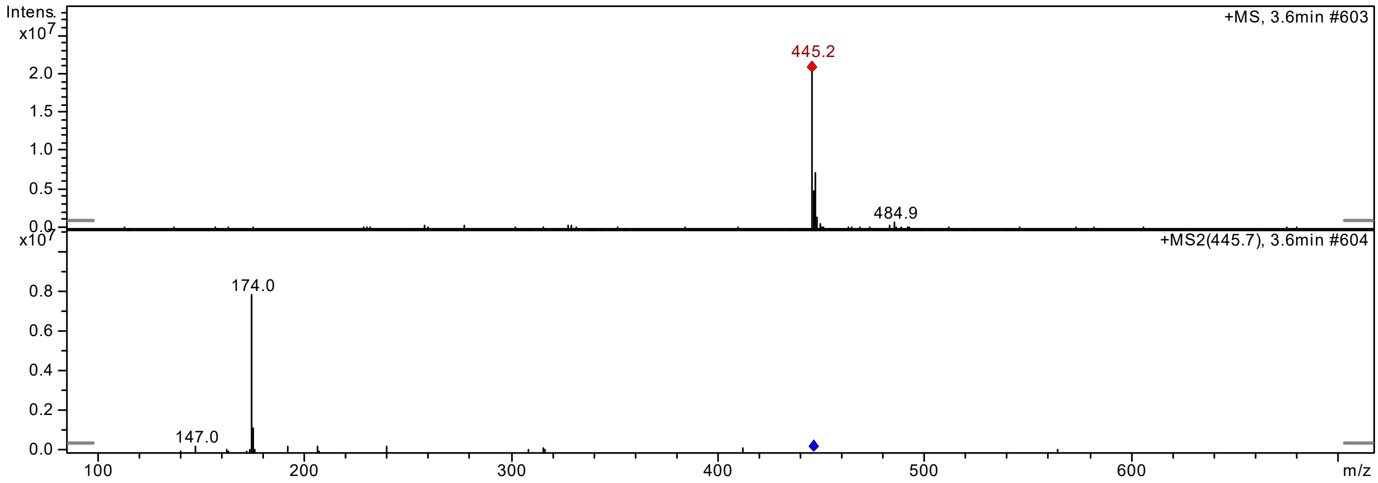

Figure S53. The MS spectrum for the compound **8i**.

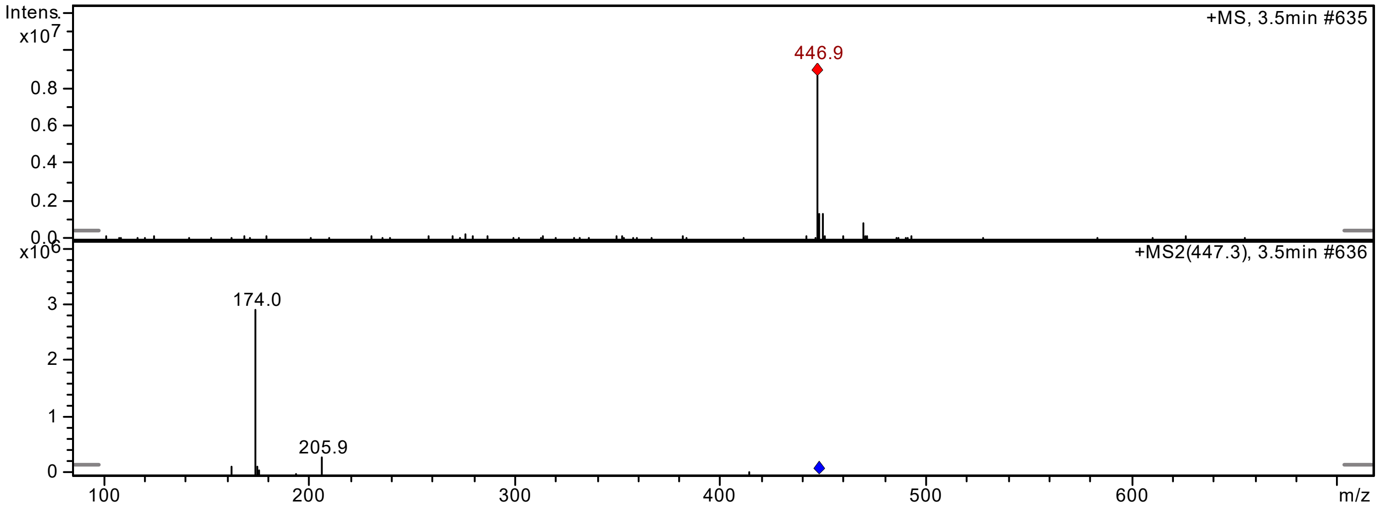

Figure S54. The MS spectrum for the compound **8j**.

S1.3. ^{1}H-NMR Spectra

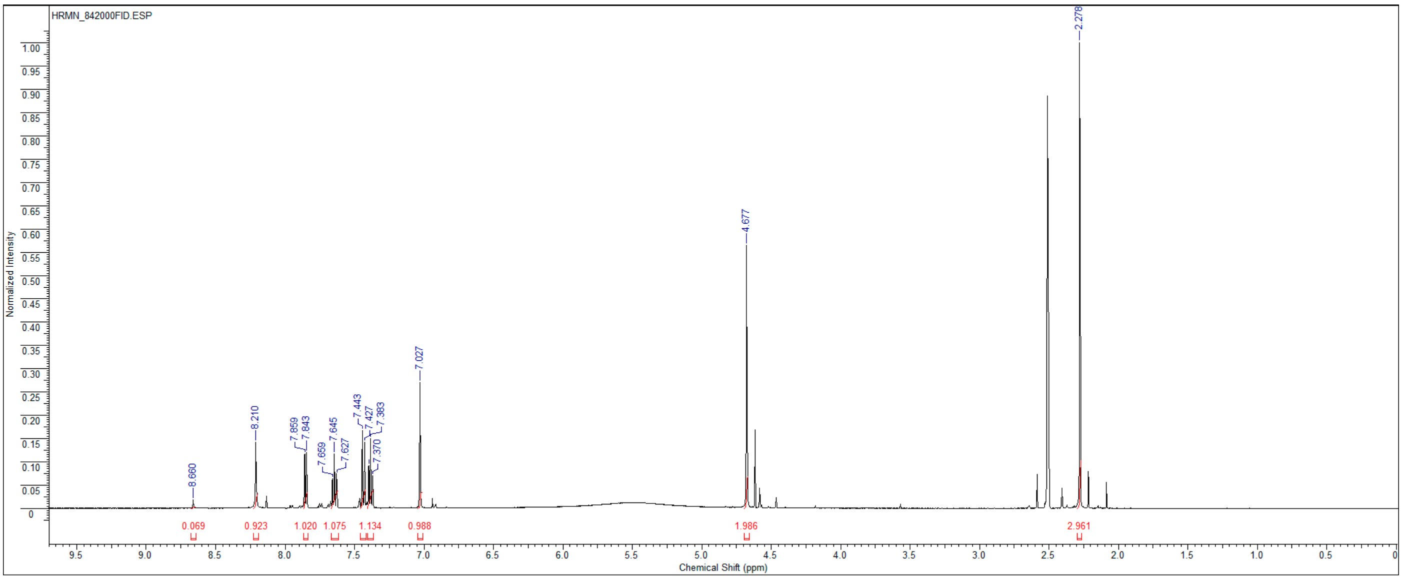

Figure S55. The ^{1}H-NMR spectrum for the compound **4a**.

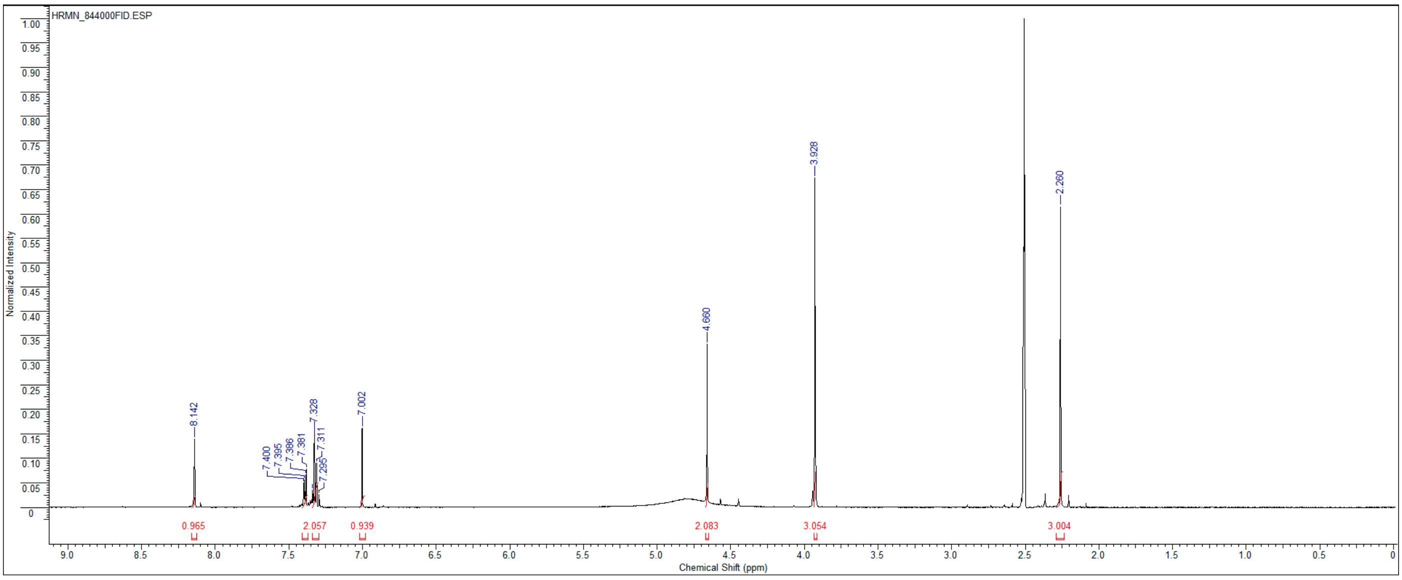

Figure S56. The ^{1}H-NMR spectrum for the compound **4b**.

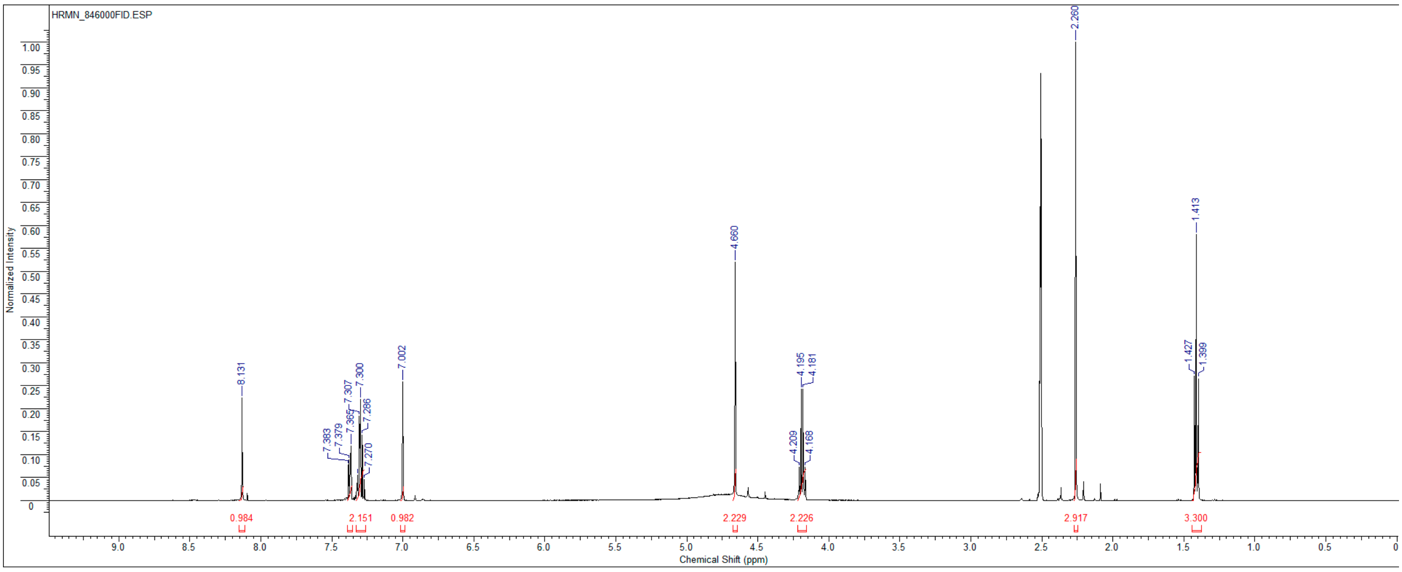

Figure S57. The ^{1}H-NMR spectrum for the compound **4c**.

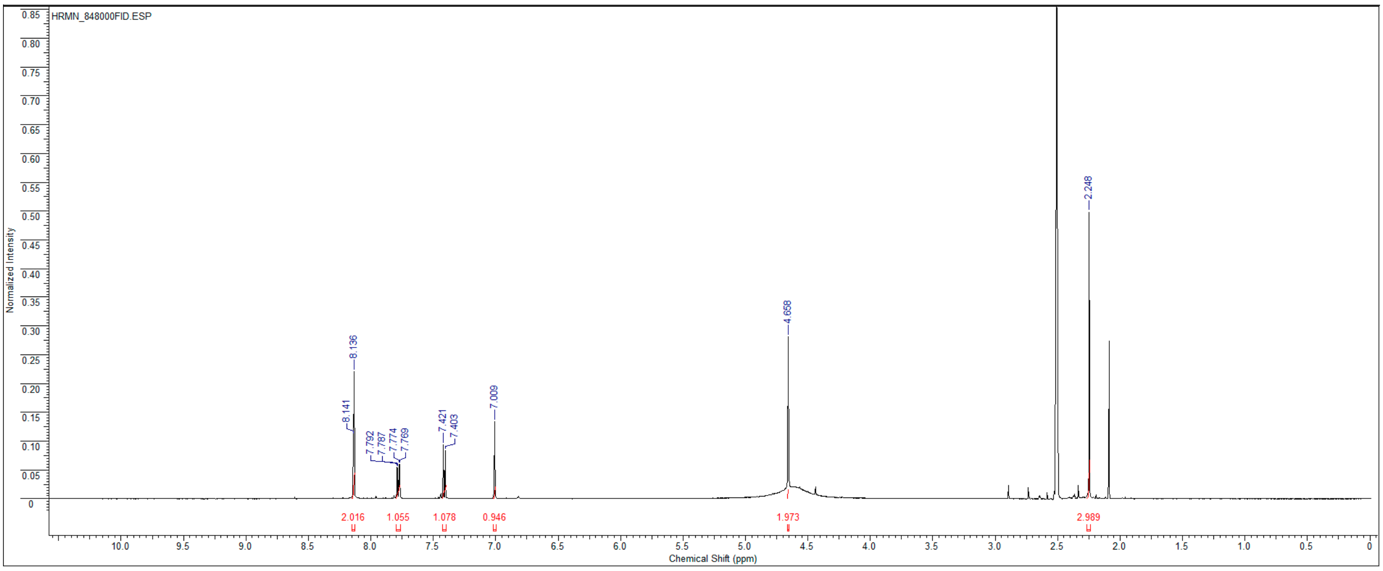

Figure S58. The ^{1}H-NMR spectrum for the compound **4d**.

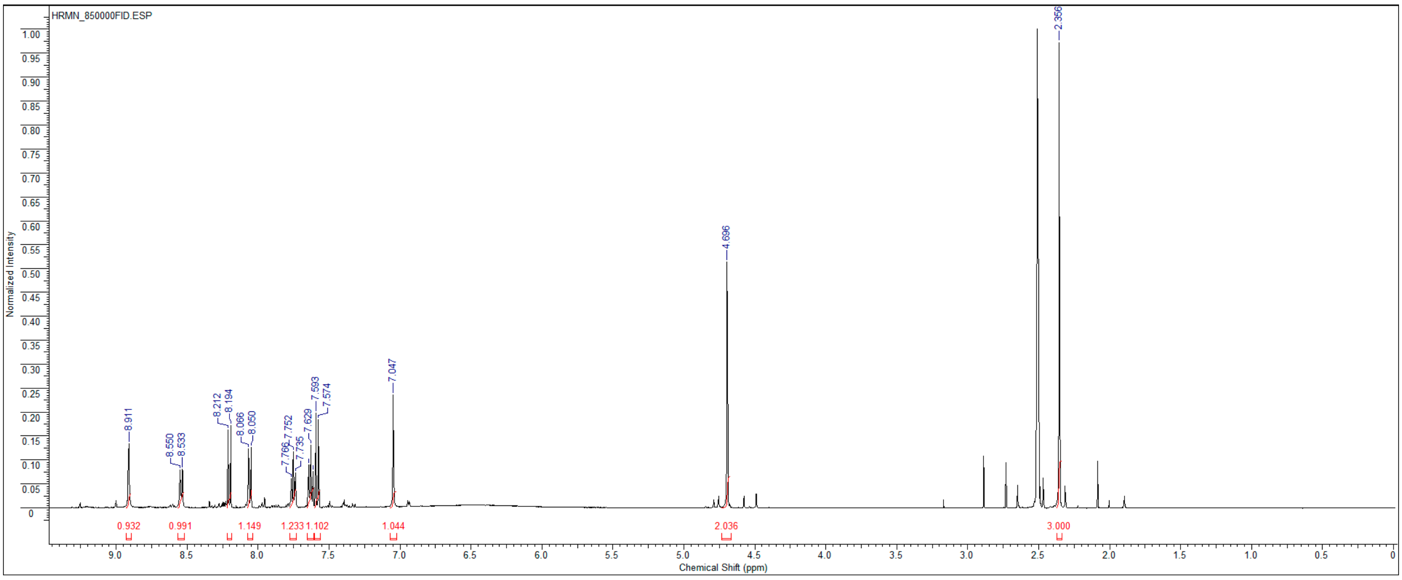

Figure S59. The ^{1}H-NMR spectrum for the compound **4e**.

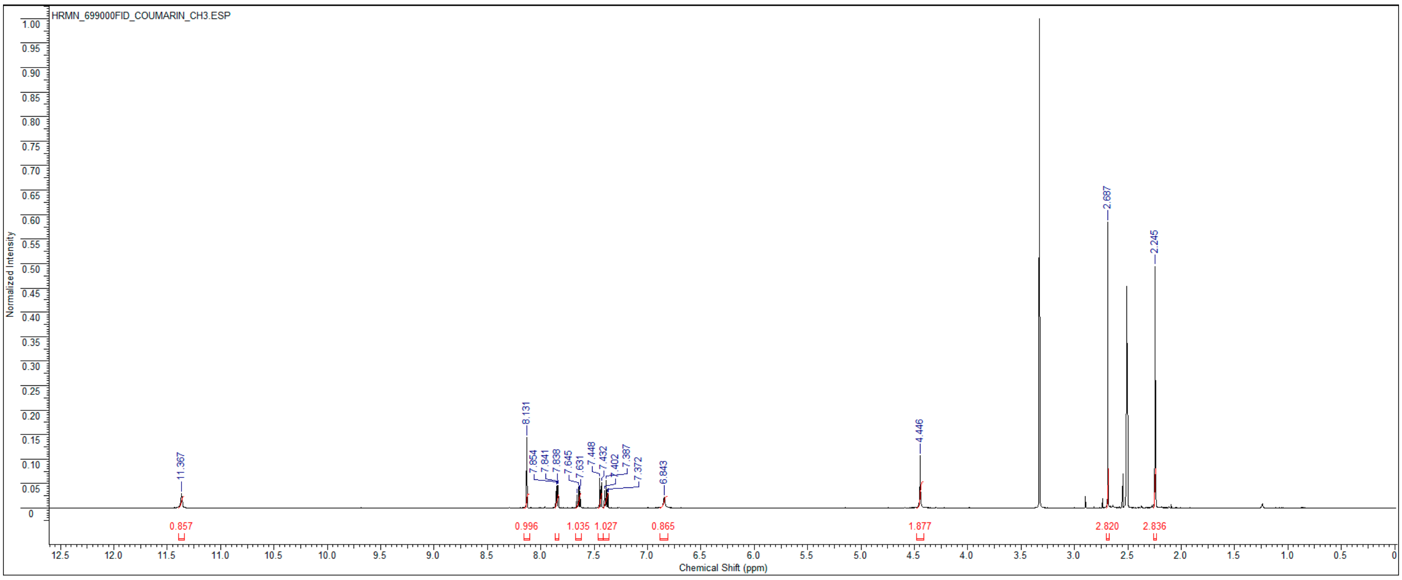

Figure S60. The ^{1}H-NMR spectrum for the compound **6a**.

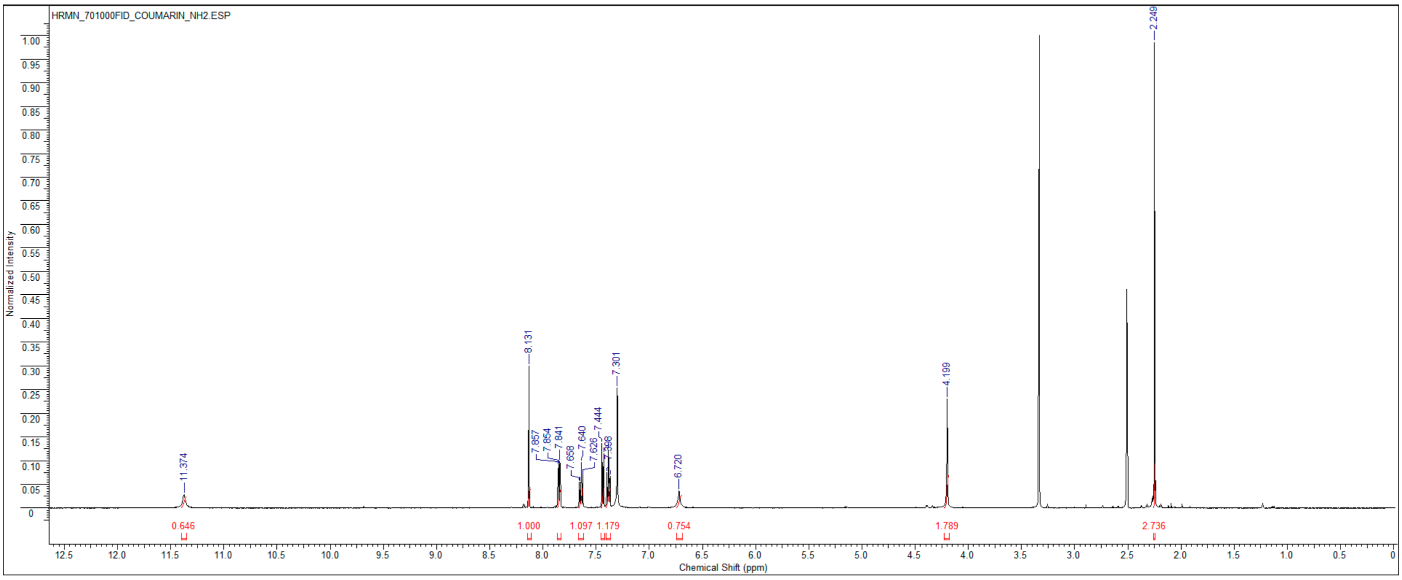

Figure S61. The ^{1}H-NMR spectrum for the compound **6b**.

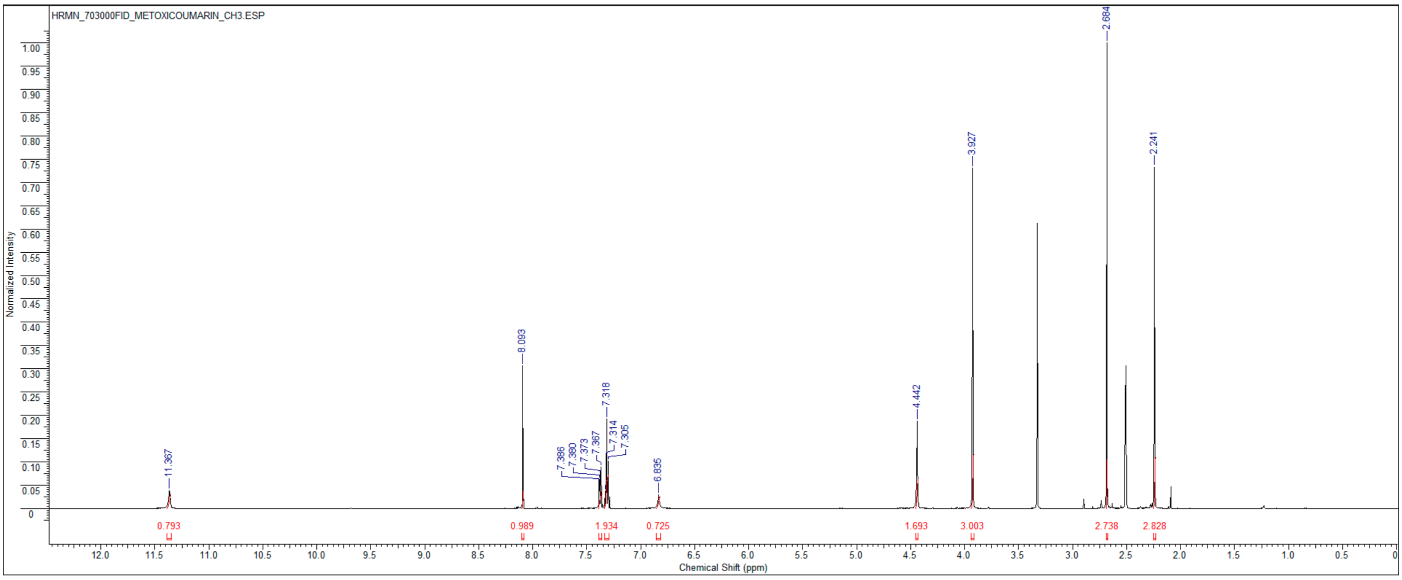

Figure S62. The ^{1}H-NMR spectrum for the compound **6c**.

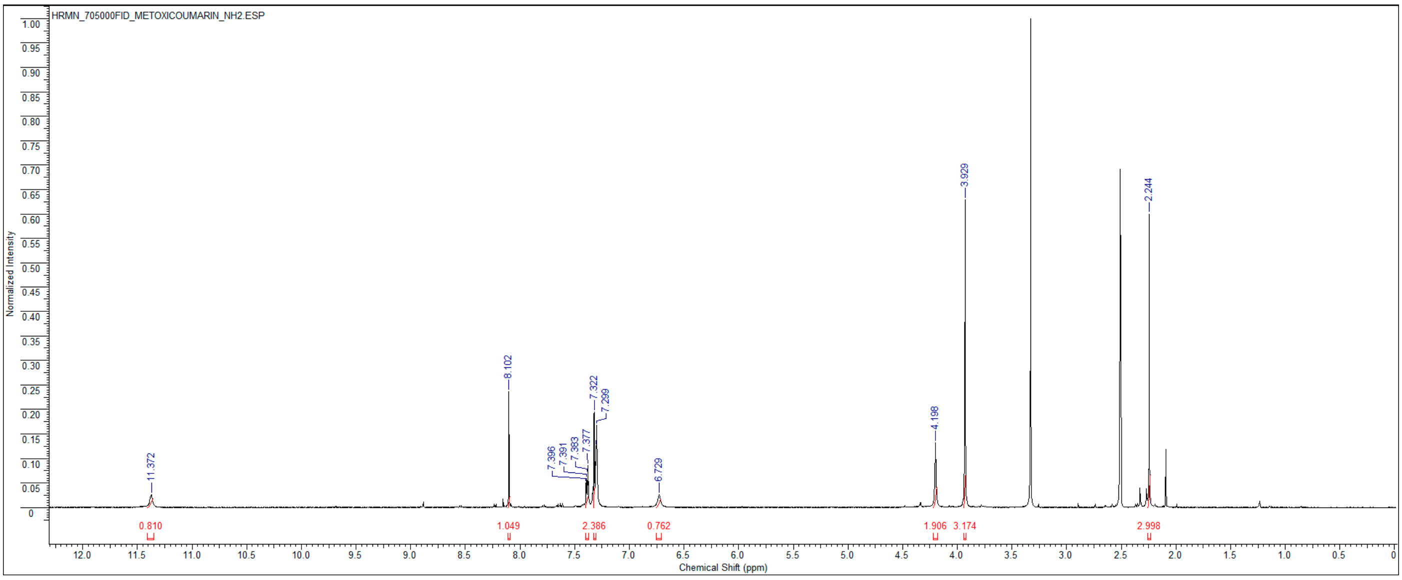

Figure S63. The ^{1}H-NMR spectrum for the compound **6d**.

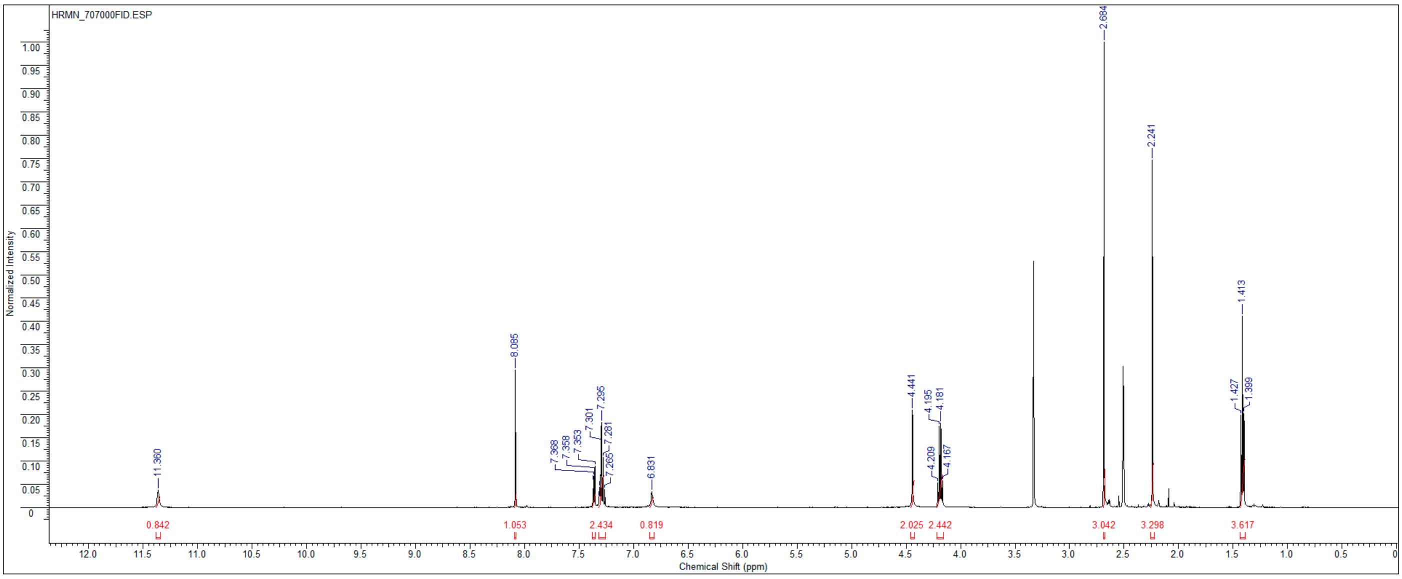

Figure S64. The ^{1}H-NMR spectrum for the compound **6e**.

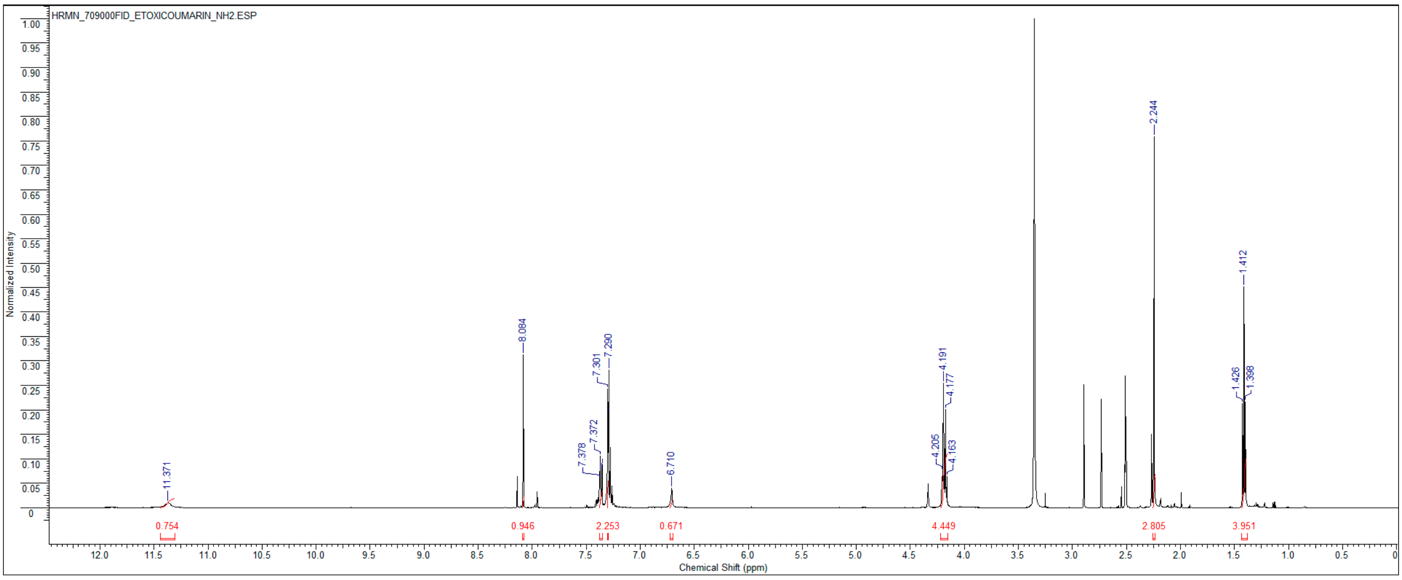

Figure S65. The ^{1}H-NMR spectrum for the compound **6f**.

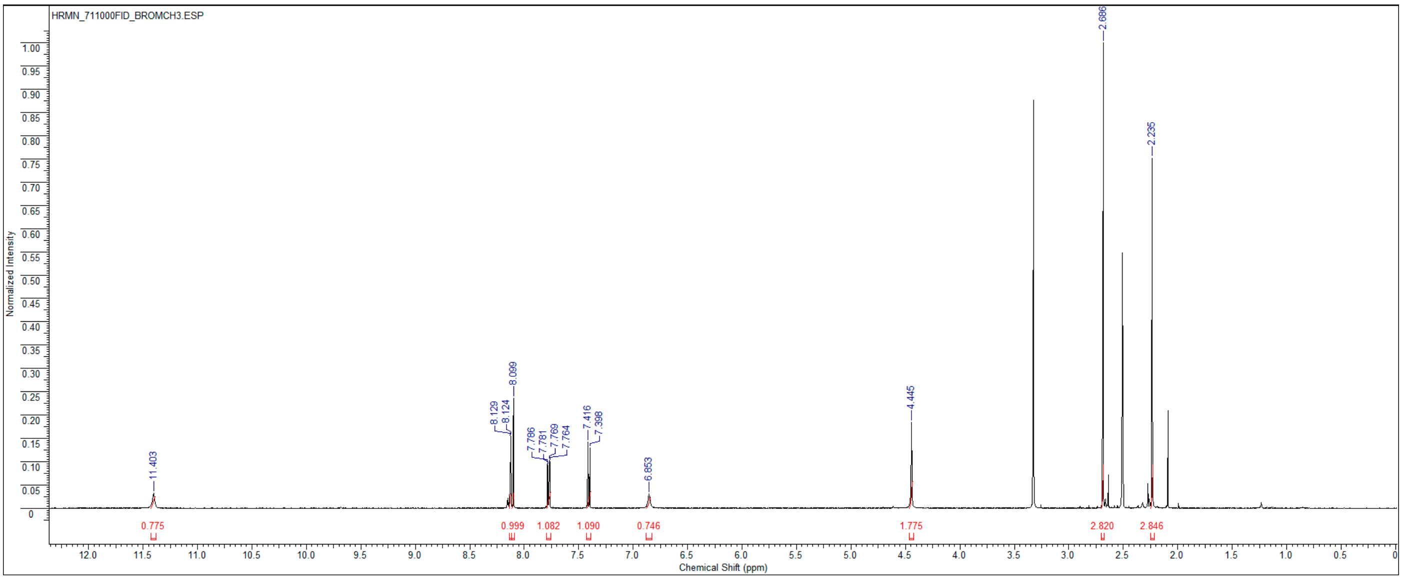

Figure S66. The ^{1}H-NMR spectrum for the compound **6g**.

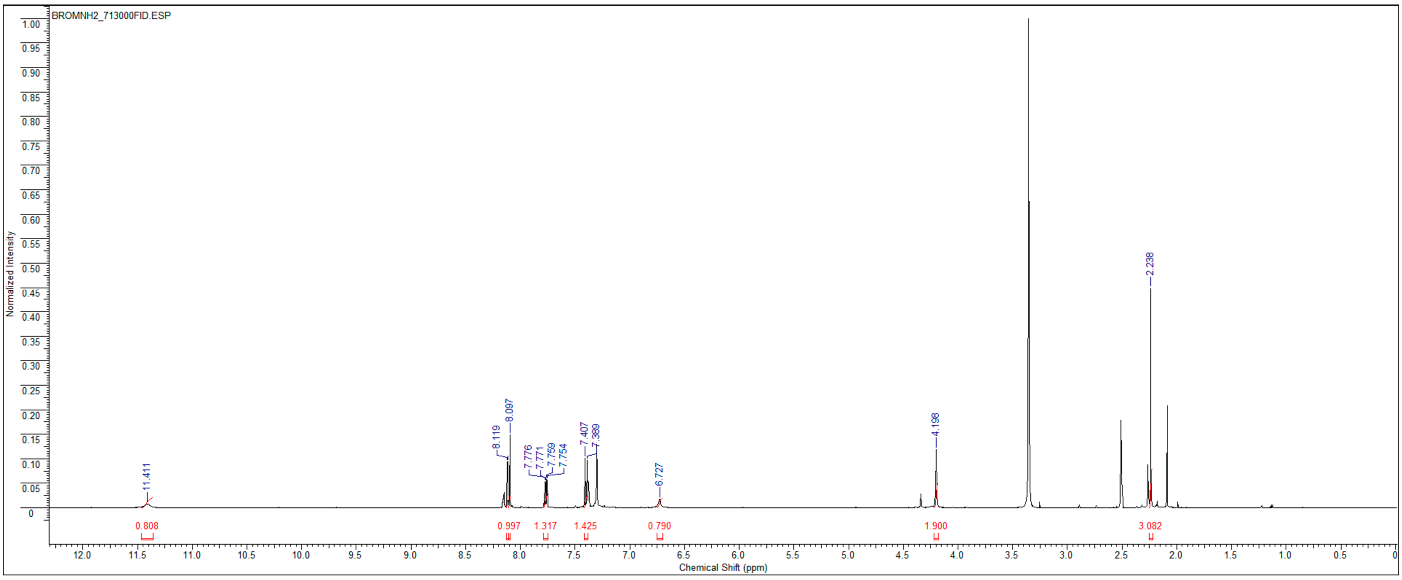

Figure S67. The ^{1}H-NMR spectrum for the compound **6h**.

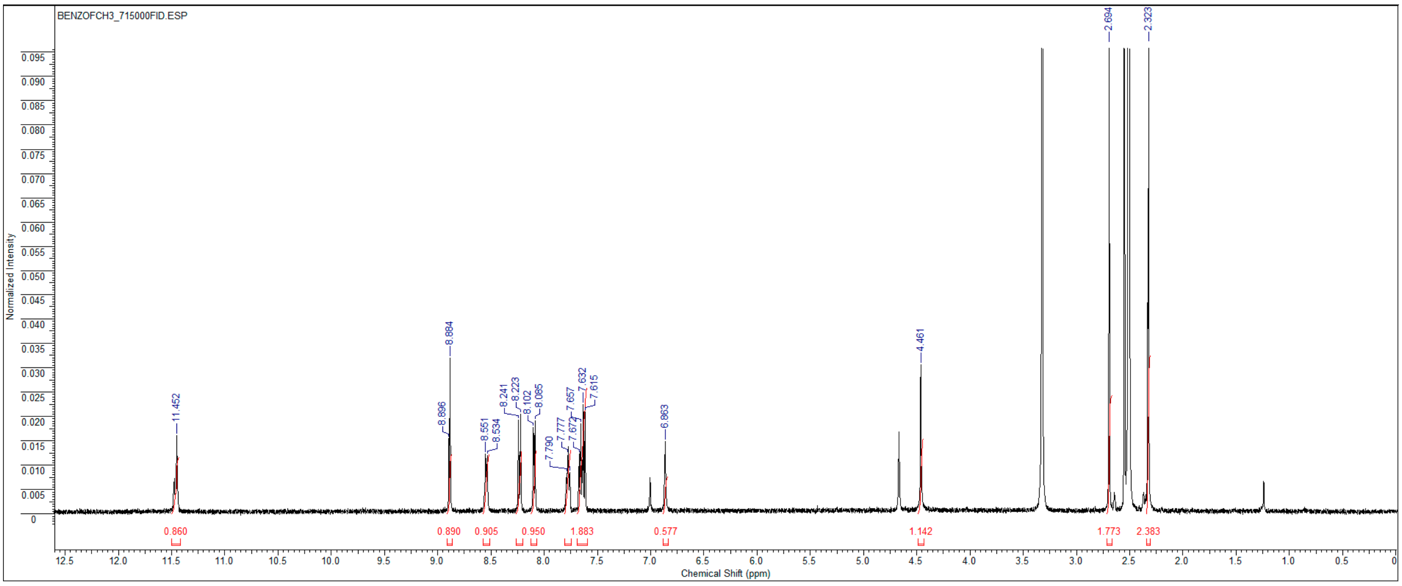

Figure S68. The ^{1}H-NMR spectrum for the compound **6i**.

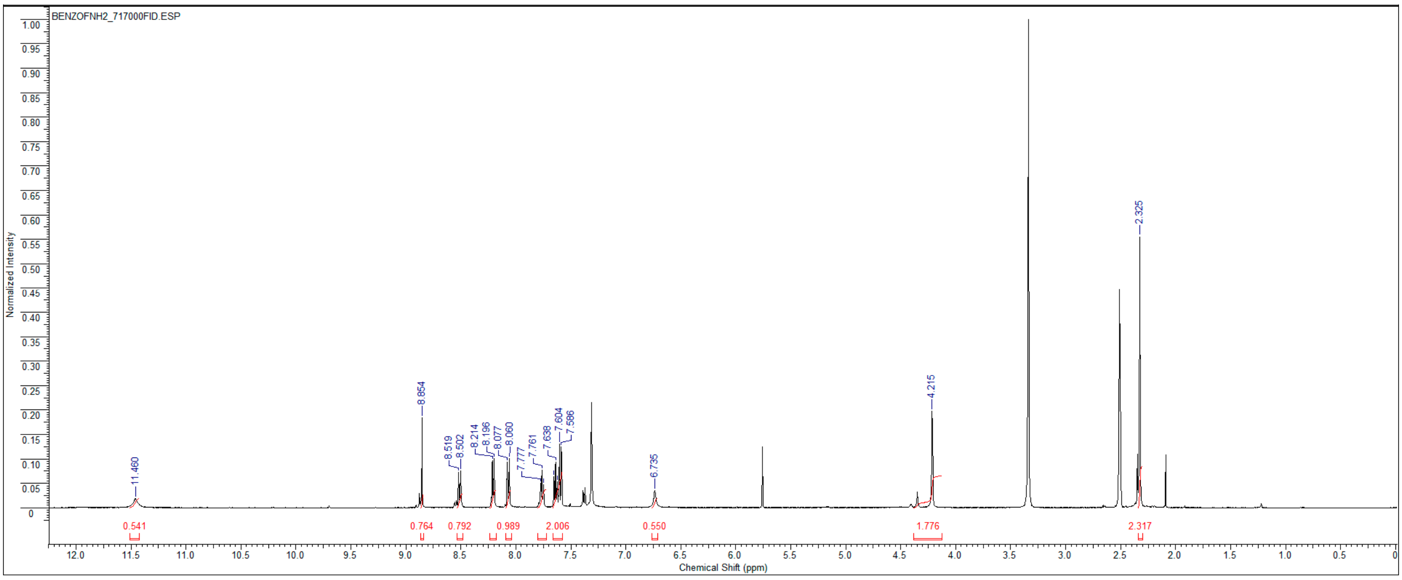

Figure S69. The ^{1}H-NMR spectrum for the compound **6j**.

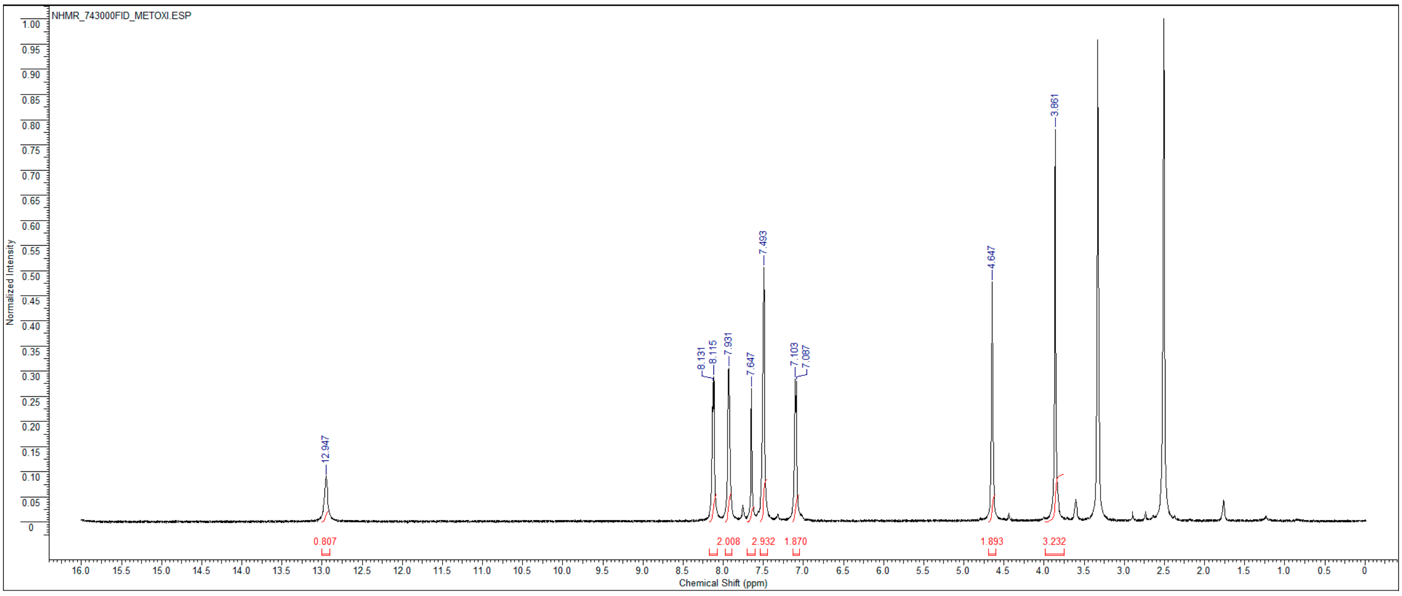

Figure S70. The ^{1}H-NMR spectrum for the compound **8a**.

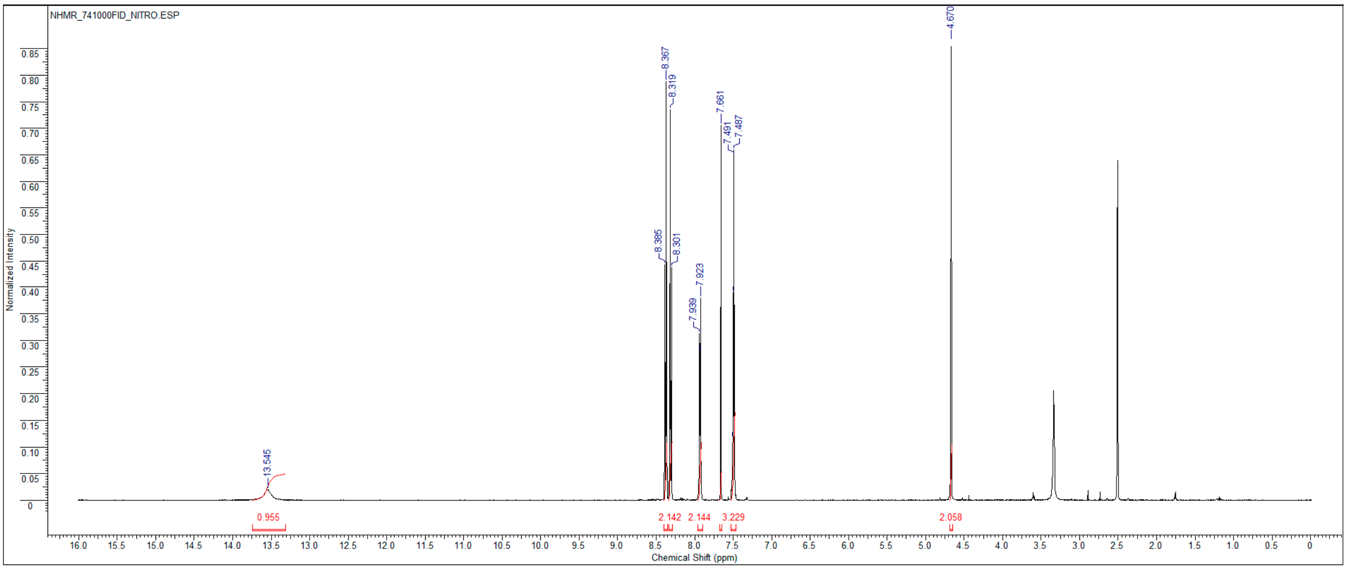

Figure S71. The ^{1}H-NMR spectrum for the compound **8b**.

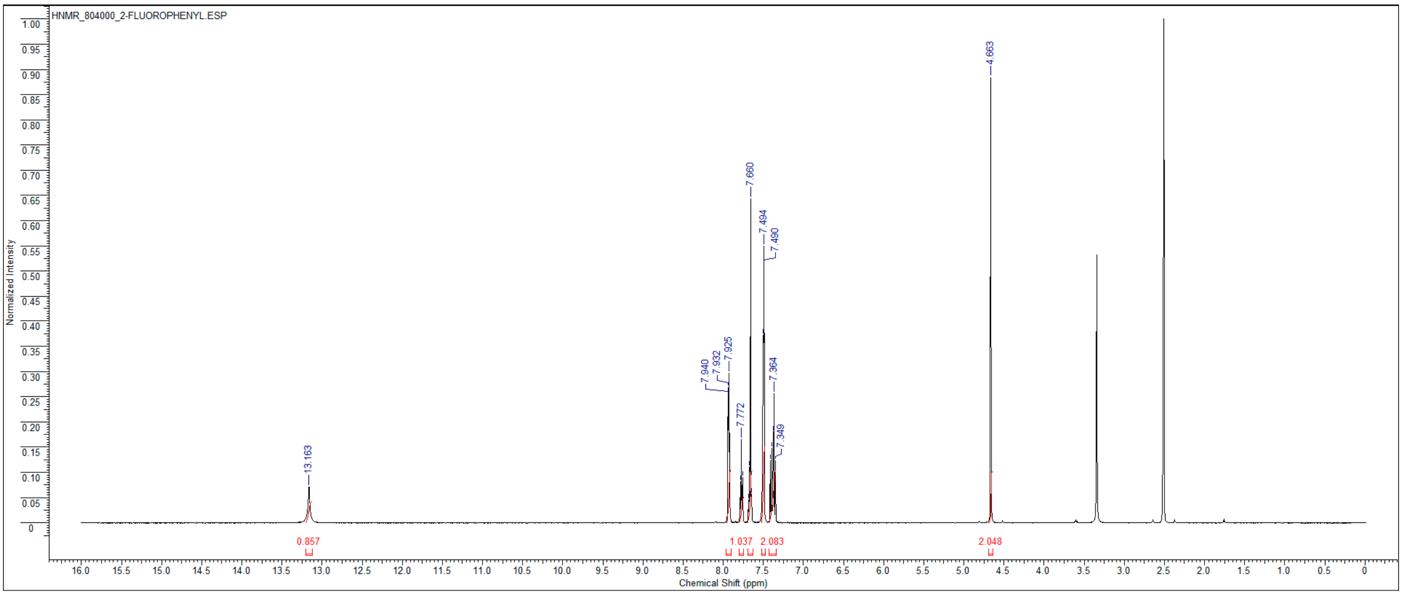

Figure S72. The ^{1}H-NMR spectrum for the compound **8c**.

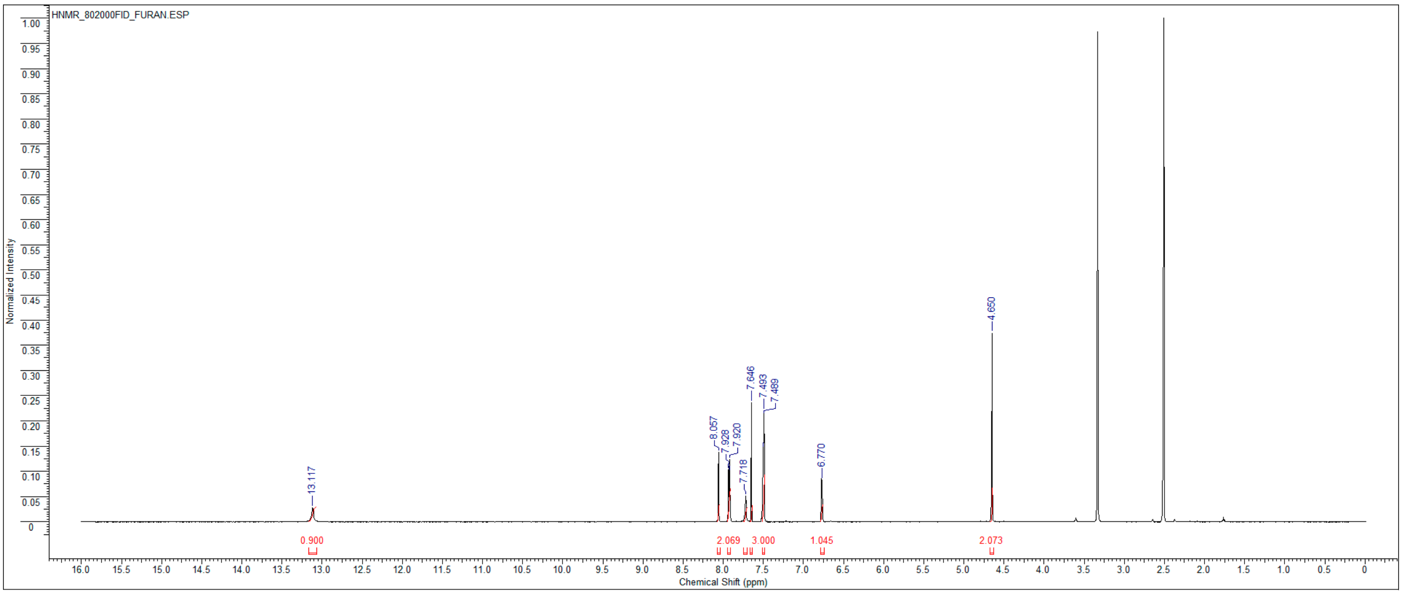

Figure S73. The ^{1}H-NMR spectrum for the compound **8d**.

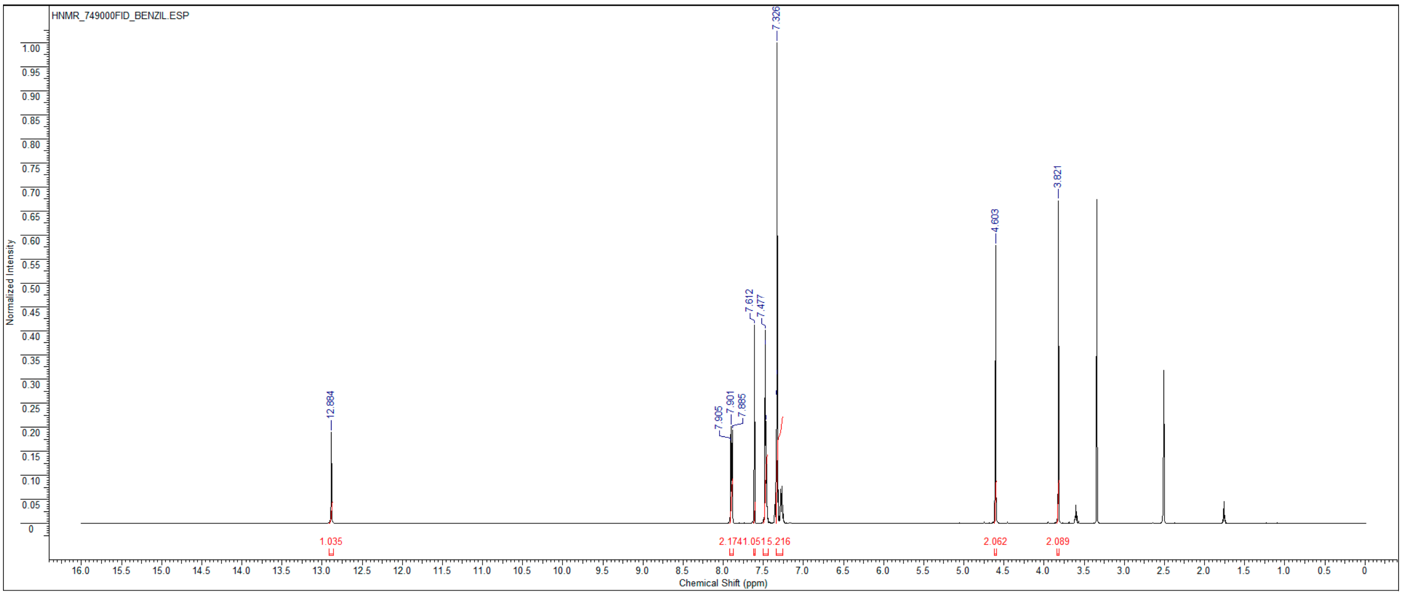

Figure S74. The ^{1}H-NMR spectrum for the compound **8e**.

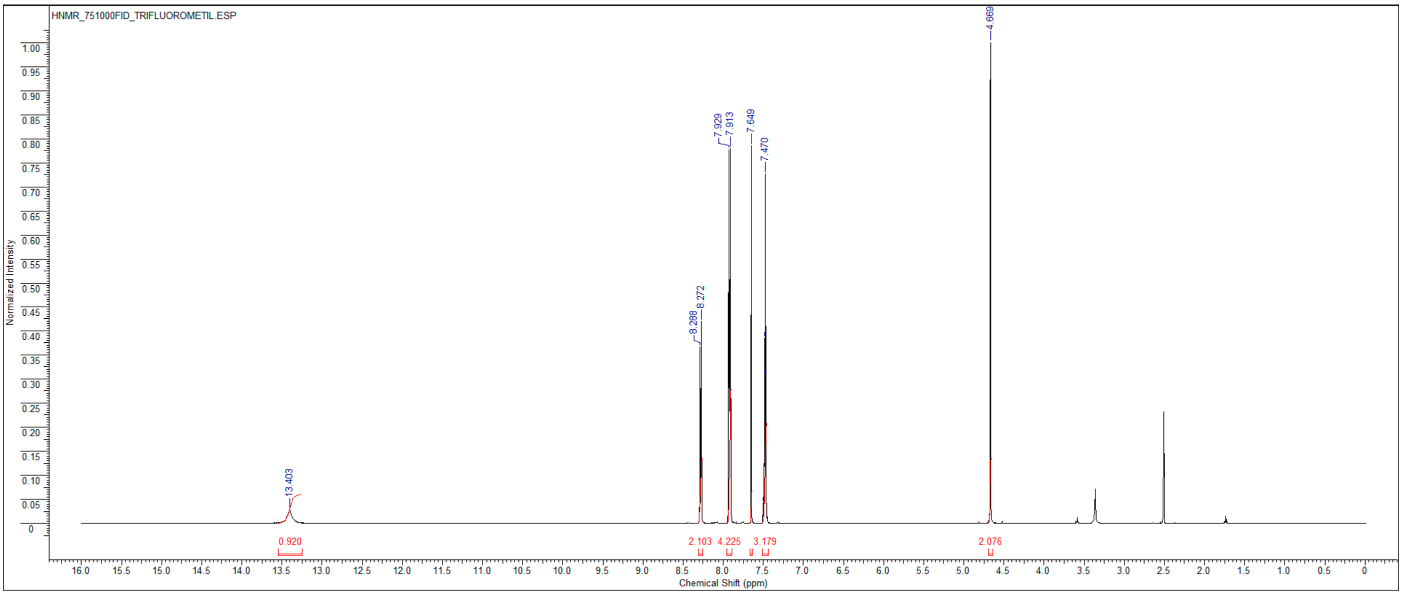

Figure S75. The ^{1}H-NMR spectrum for the compound **8f**.

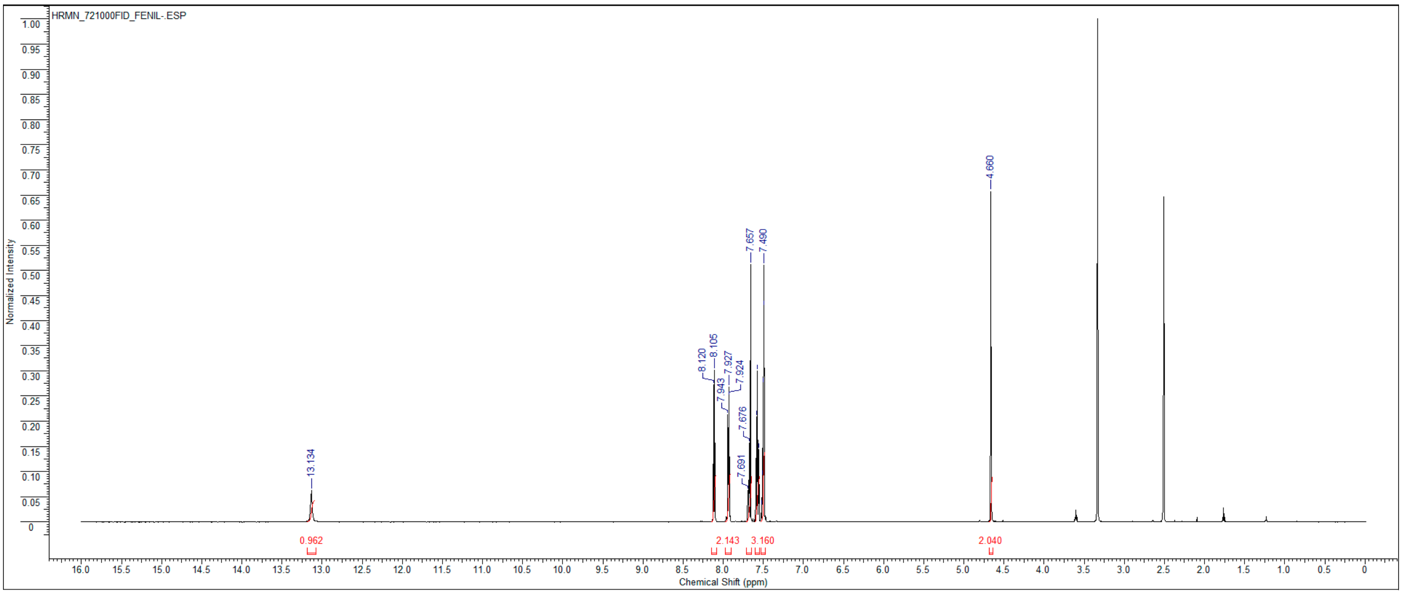

Figure S76. The ^{1}H-NMR spectrum for the compound **8g**.

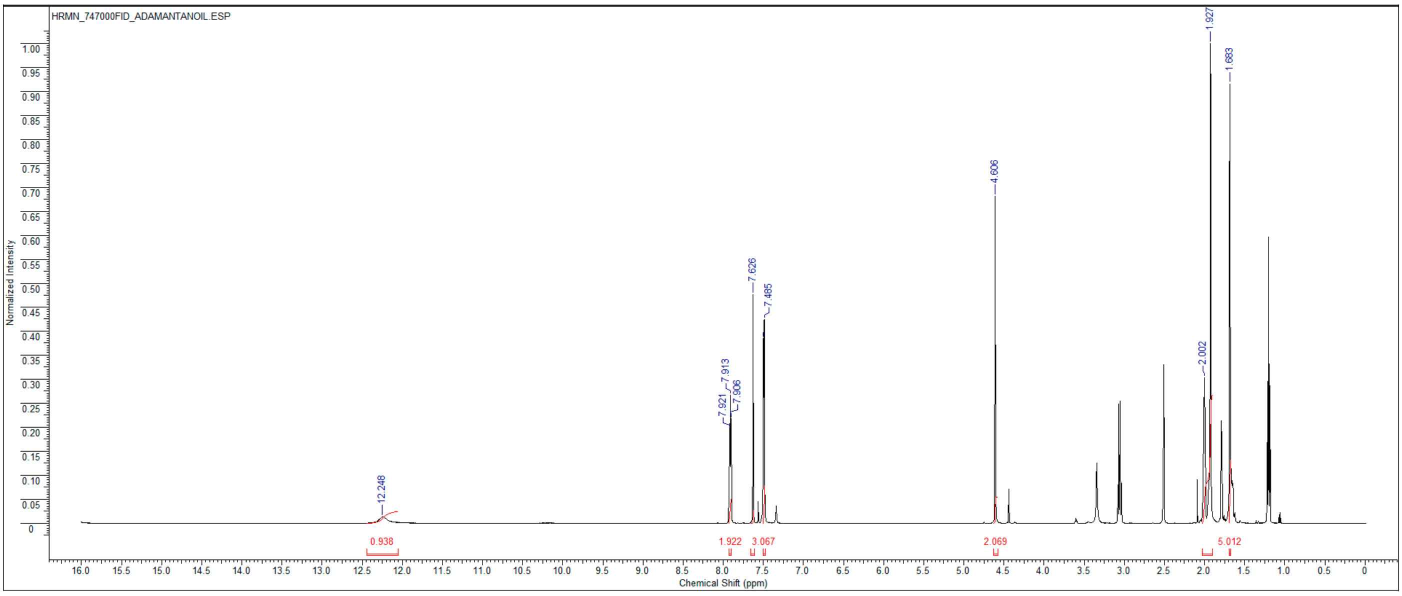

Figure S77. The ^{1}H-NMR spectrum for the compound **8h**.

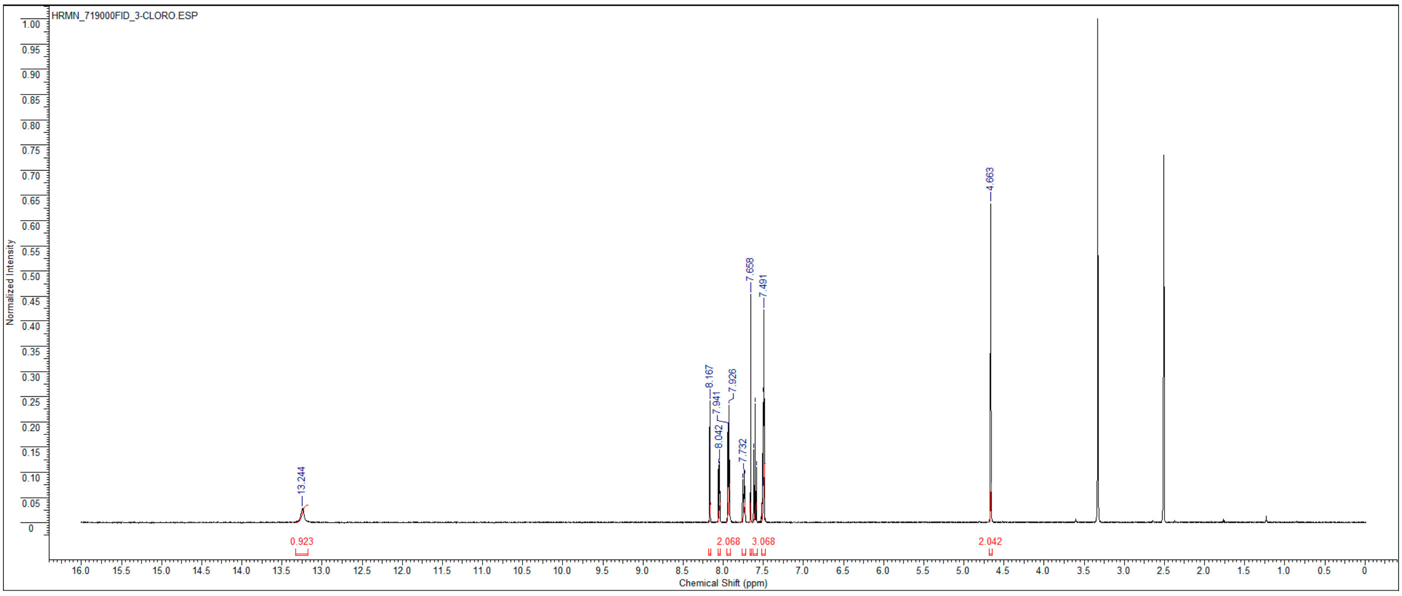

Figure S78. The ^{1}H-NMR spectrum for the compound **8i**.

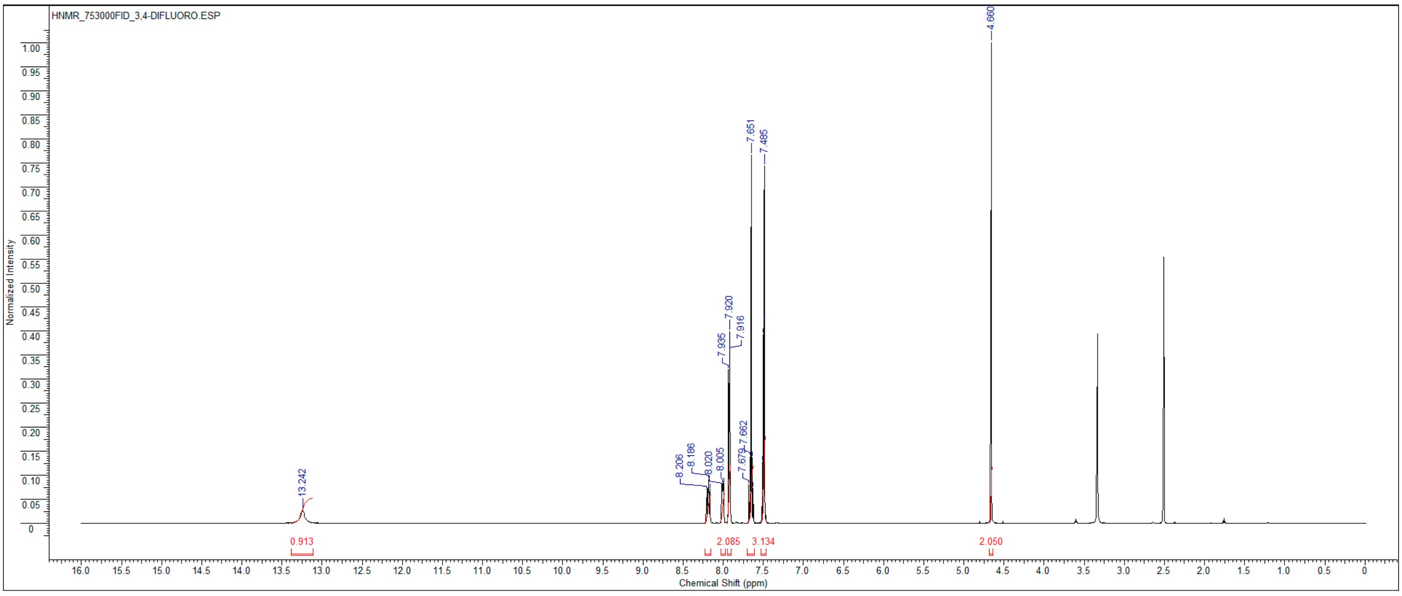

Figure S79. The ^{1}H-NMR spectrum for the compound **8j**.

S1.4. ^{13}C-NMR Spectra

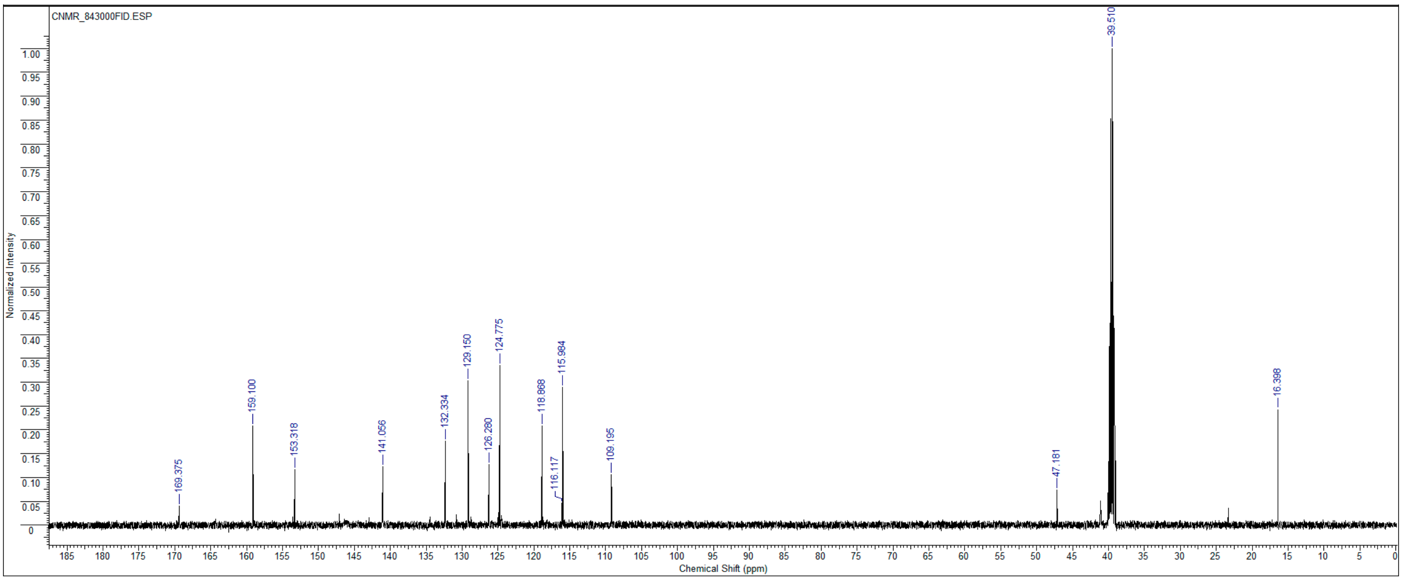

Figure S80. The ^{13}C-NMR spectrum for the compound **4a**.

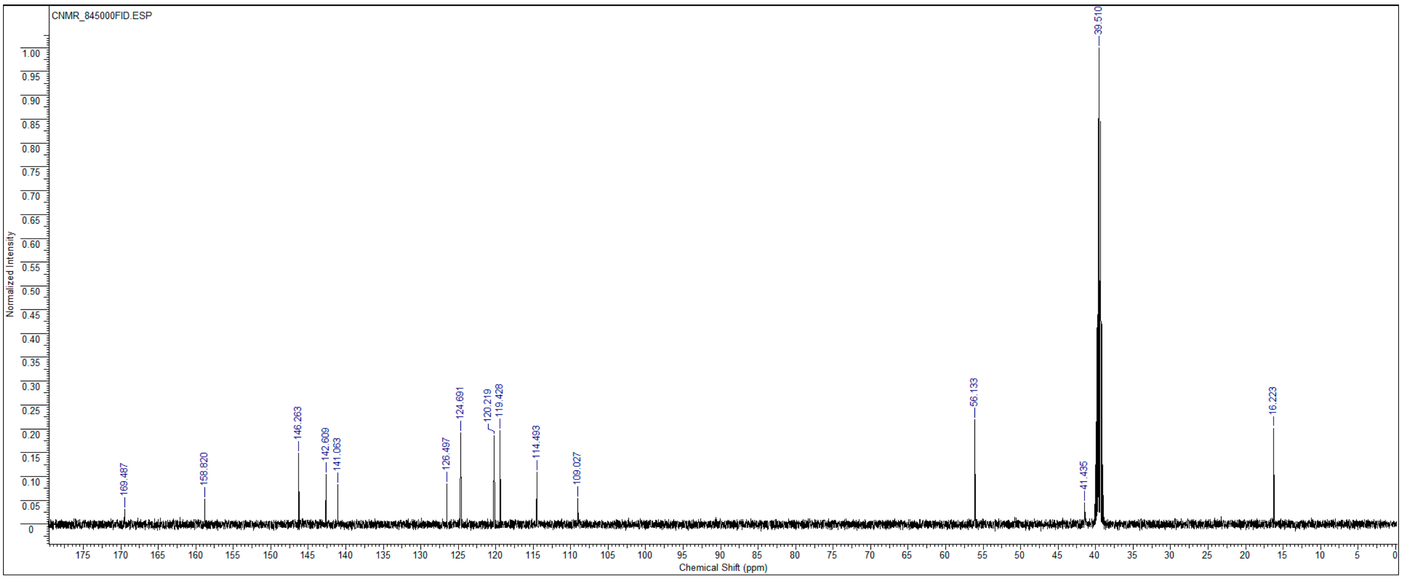

Figure S81. The ^{13}C-NMR spectrum for the compound **4b**.

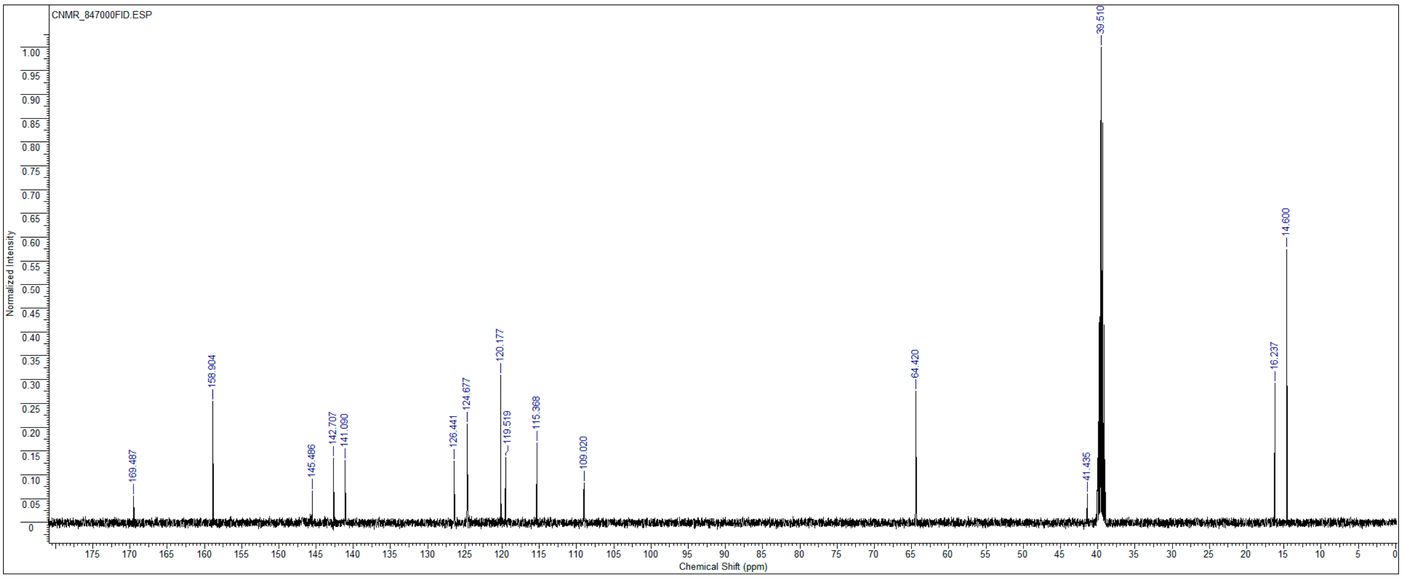

Figure S82. The ^{13}C-NMR spectrum for the compound **4c**.

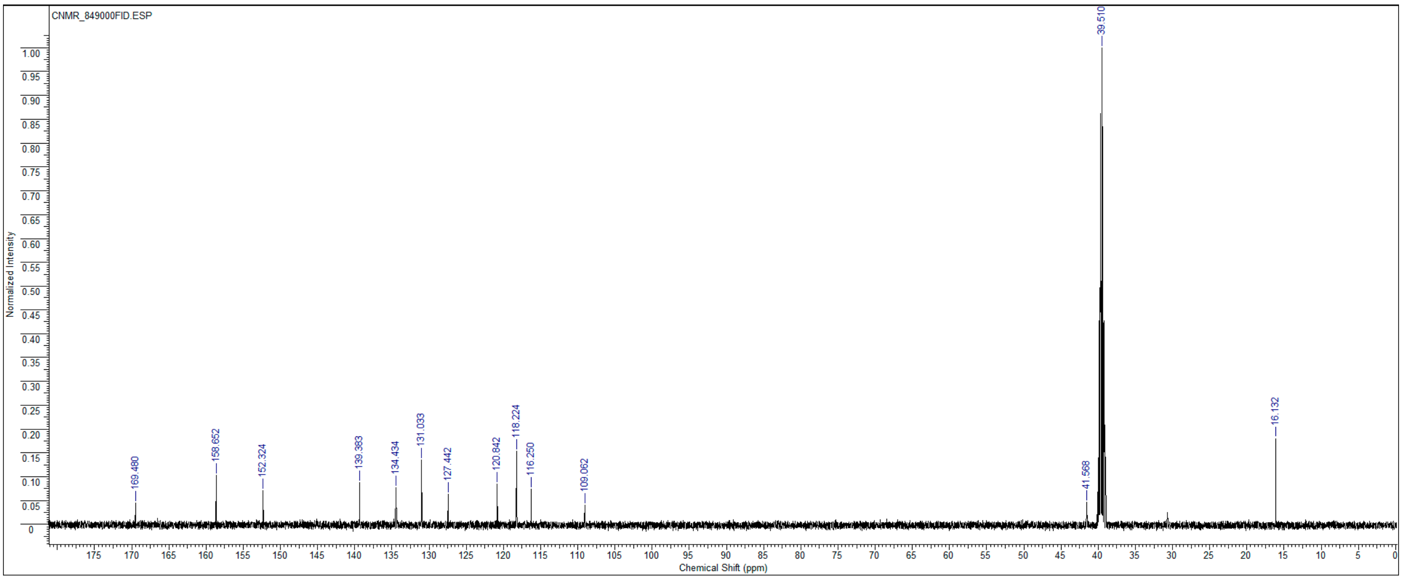

Figure S83. The ^{13}C-NMR spectrum for the compound **4d**.

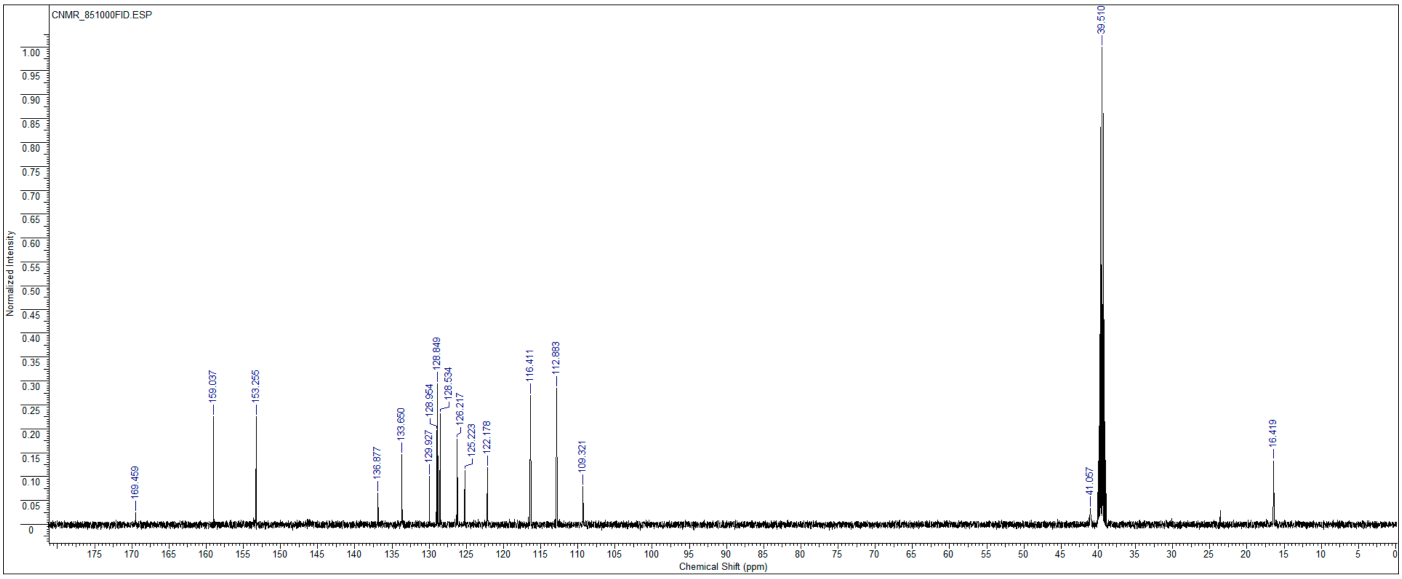

Figure S84. The ^{13}C-NMR spectrum for the compound **4e**.

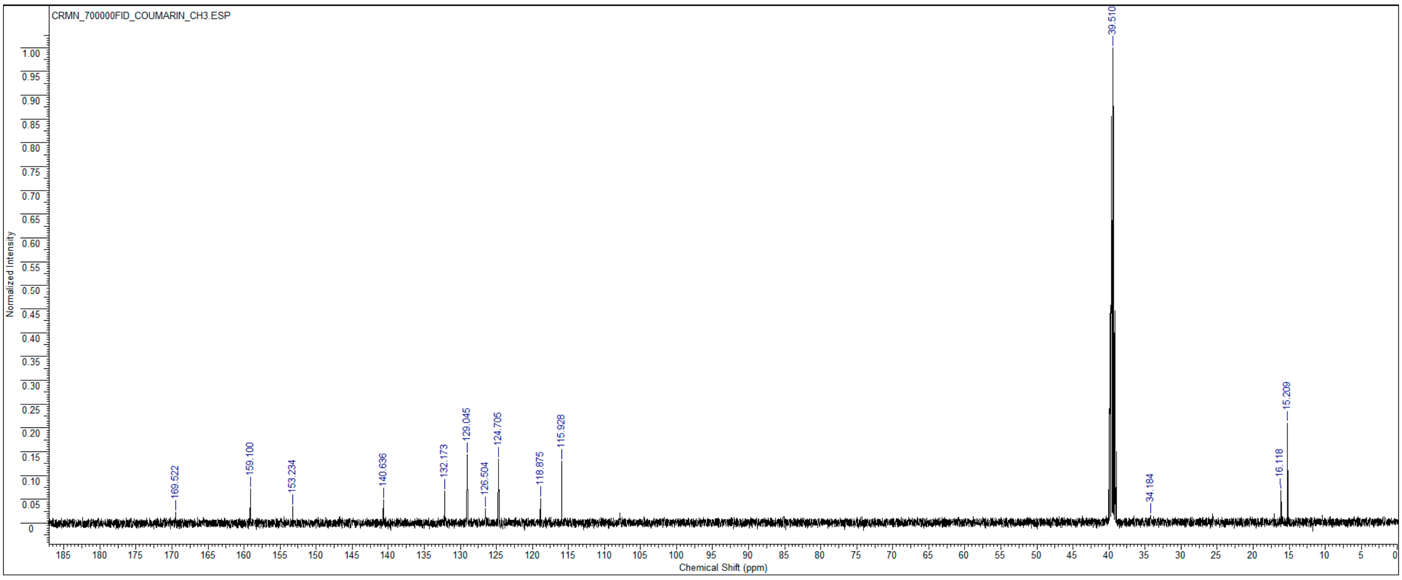

Figure S85. The ^{13}C-NMR spectrum for the compound **6a**.

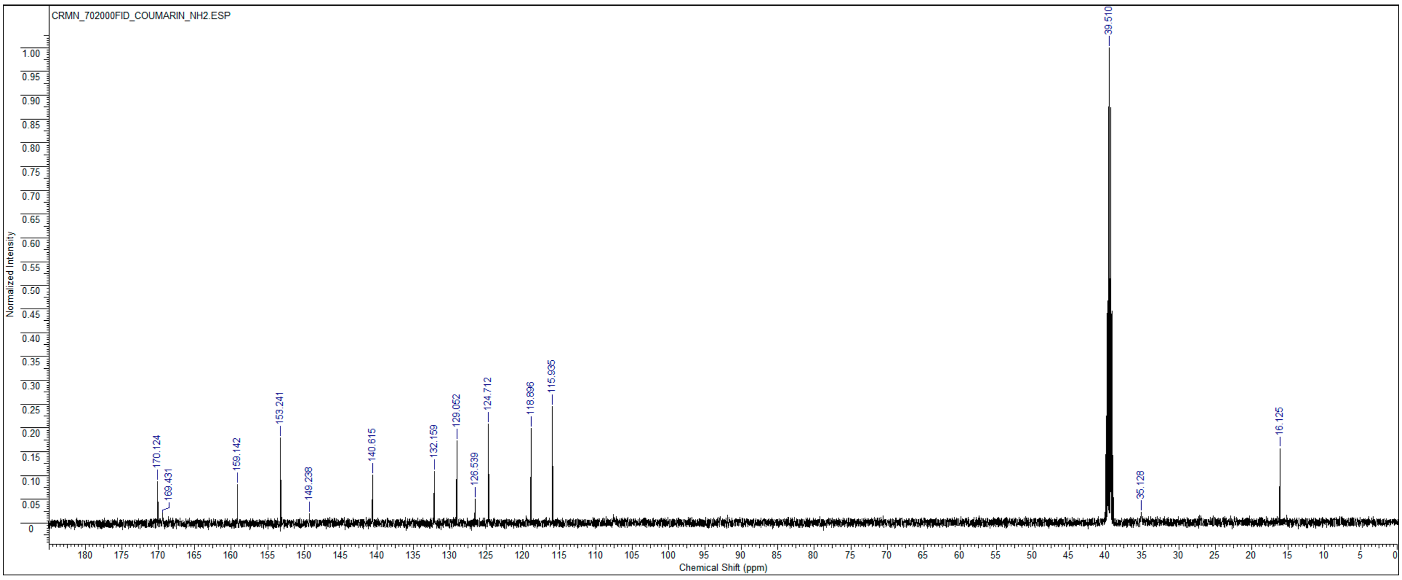

Figure S86. The ^{13}C-NMR spectrum for the compound **6b**.

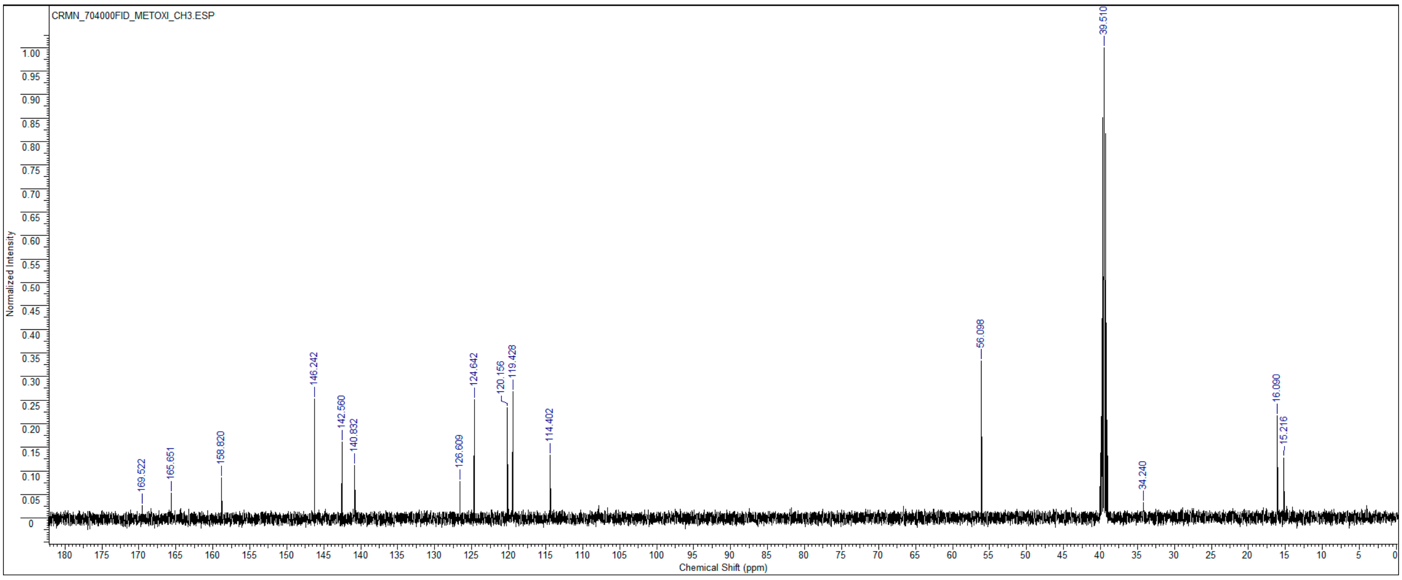

Figure S87. The ^{13}C-NMR spectrum for the compound **6c**.

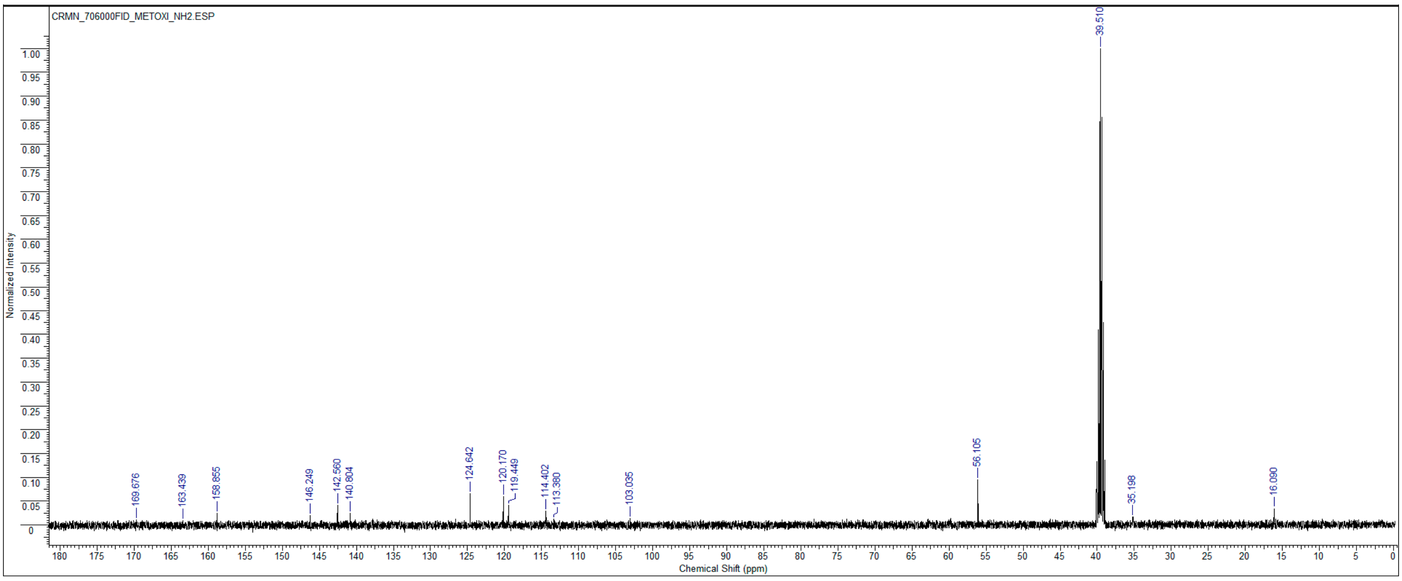

Figure S88. The ^{13}C-NMR spectrum for the compound **6d**.

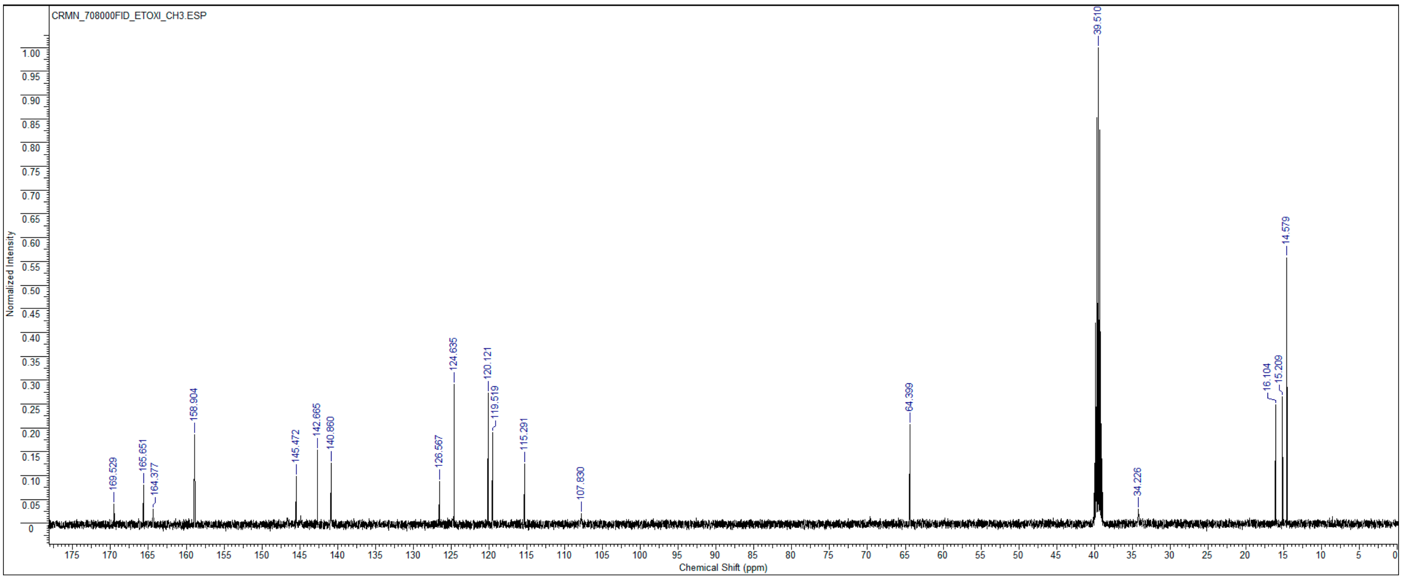

Figure S89. The ^{13}C-NMR spectrum for the compound **6e**.

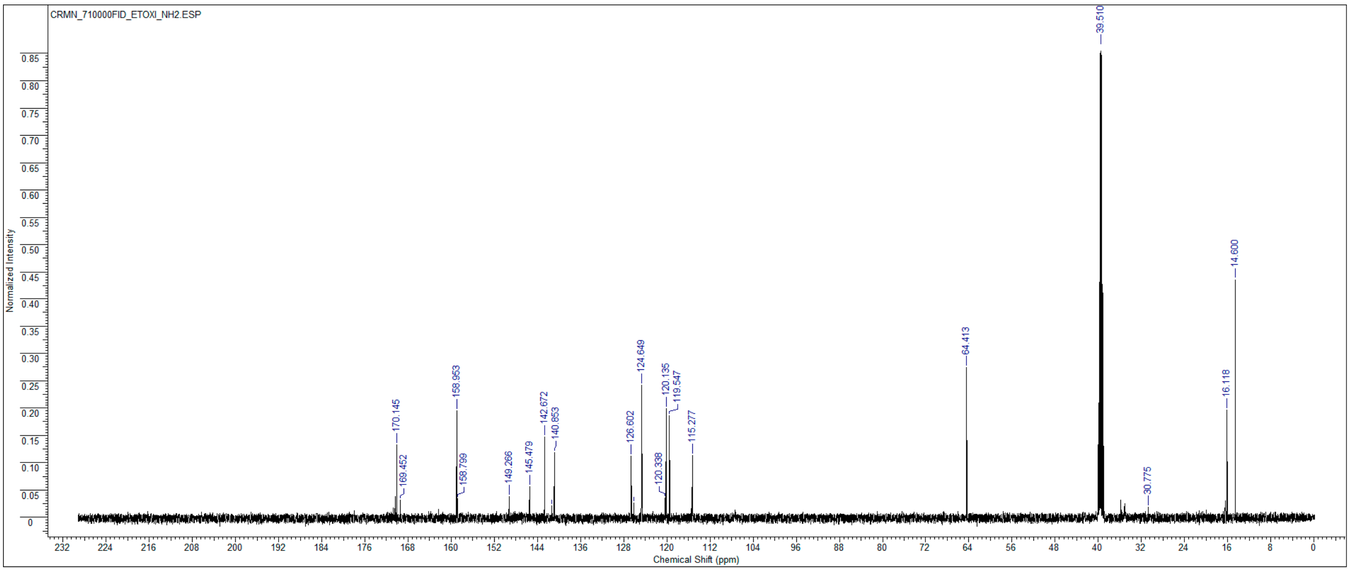

Figure S90. The ^{13}C-NMR spectrum for the compound **6f**.

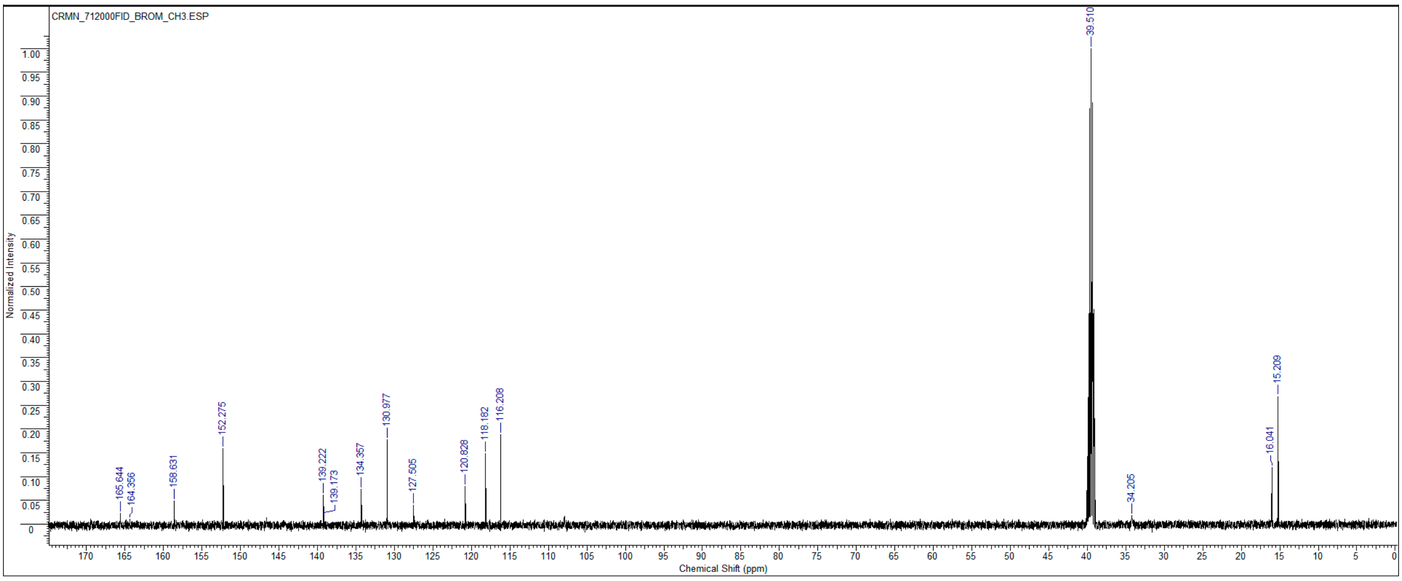

Figure S91. The ^{13}C-NMR spectrum for the compound **6g**.

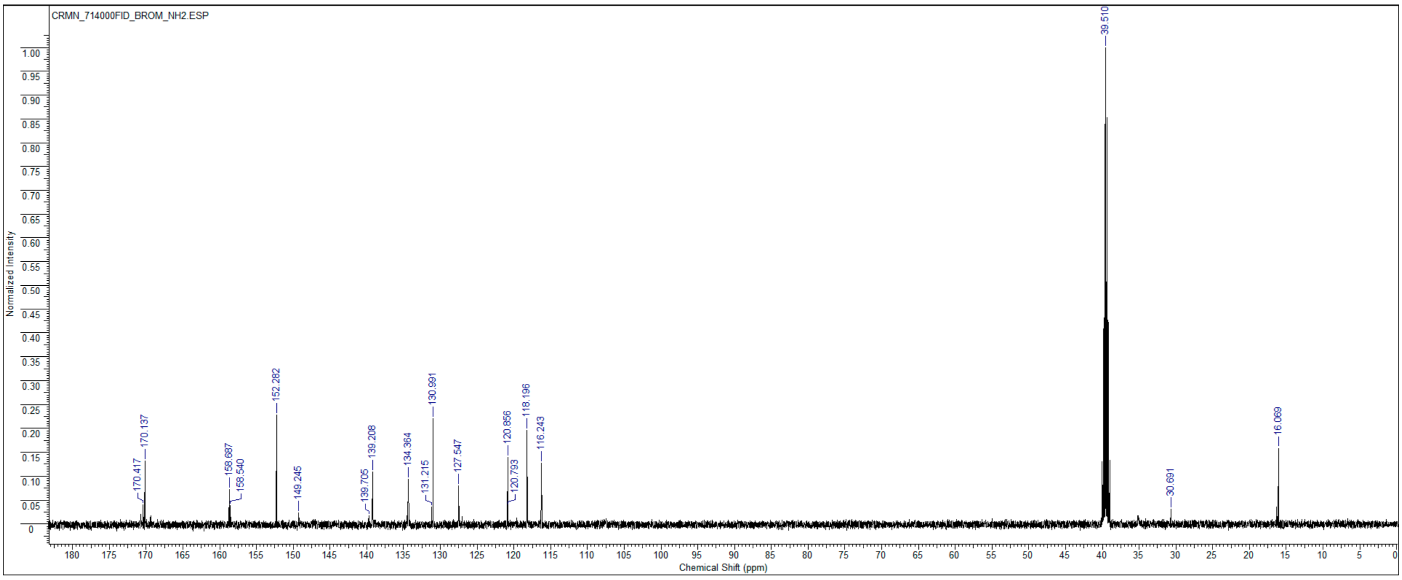

Figure S92. The ^{13}C-NMR spectrum for the compound **6h**.

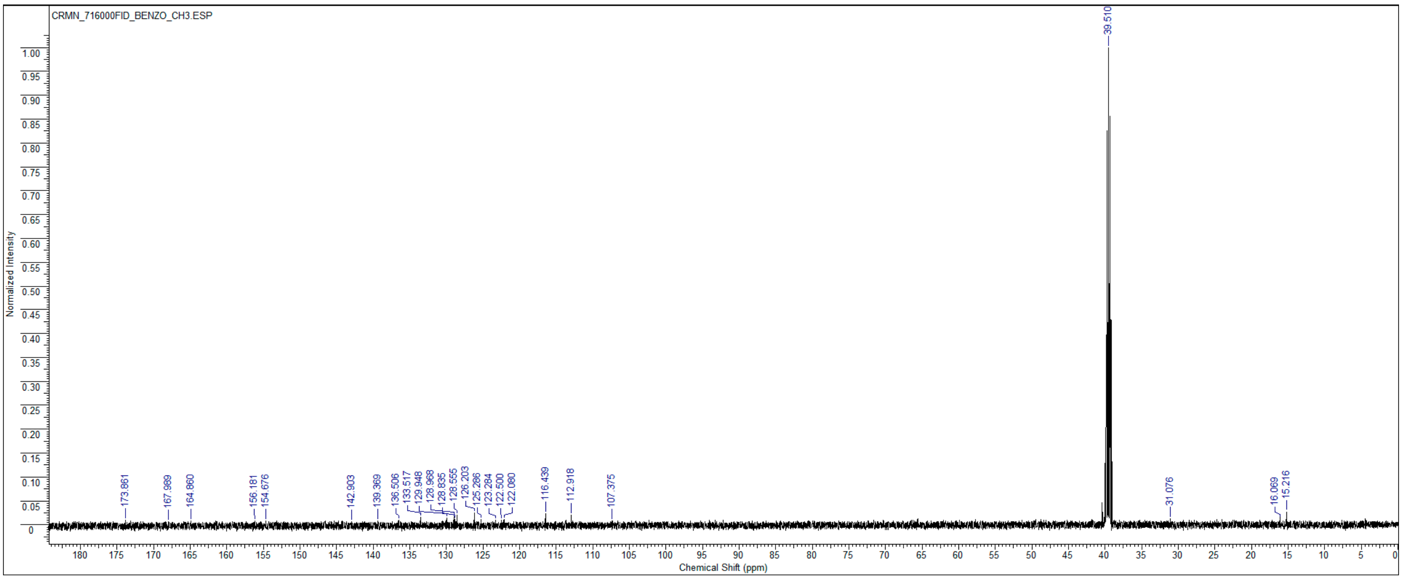

Figure S93. The ^{13}C-NMR spectrum for the compound **6i**.

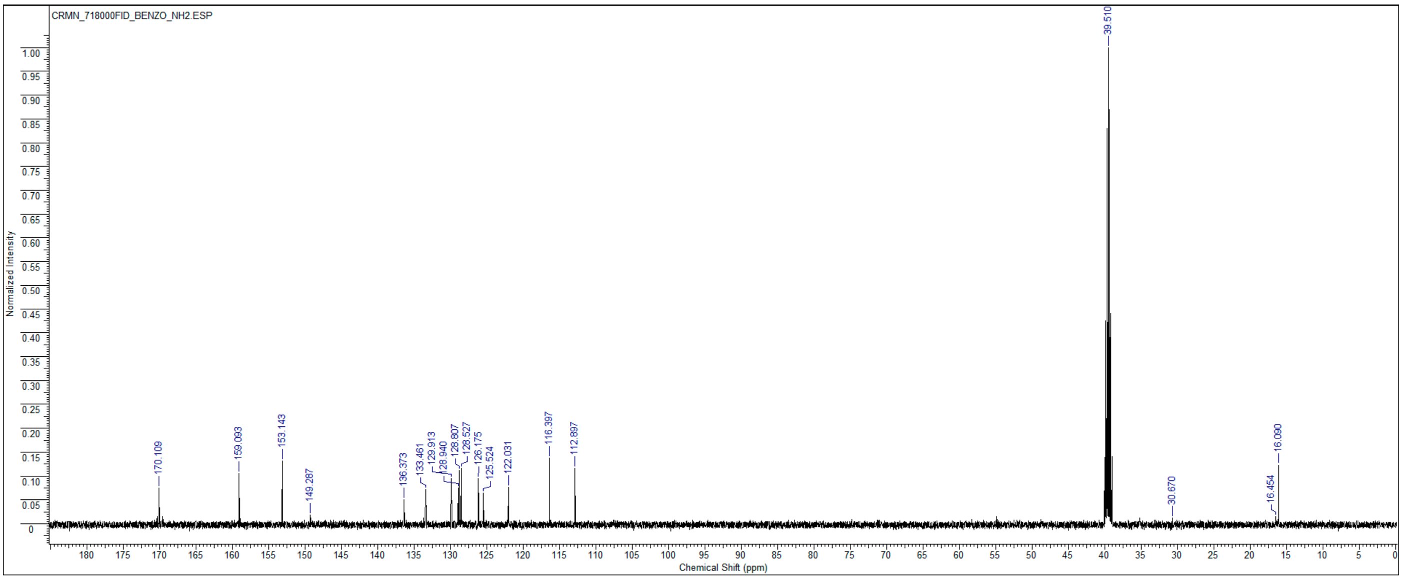

Figure S94. The ^{13}C-NMR spectrum for the compound **6j**.

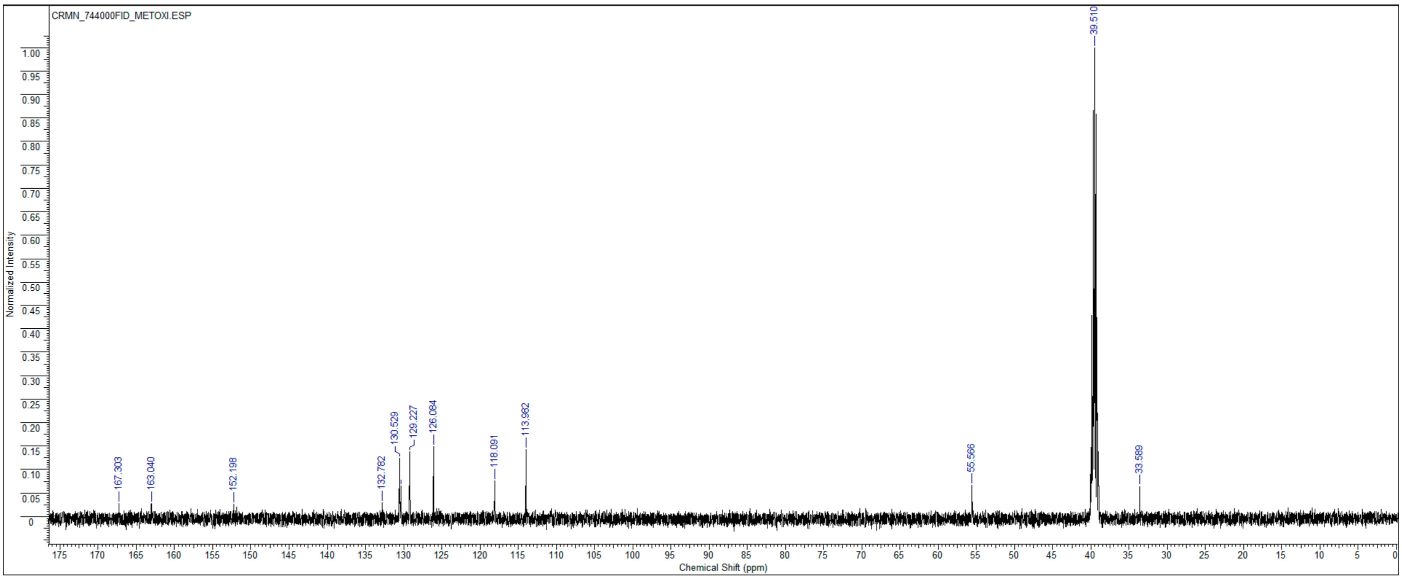

Figure S95. The ^{13}C-NMR spectrum for the compound **8a**.

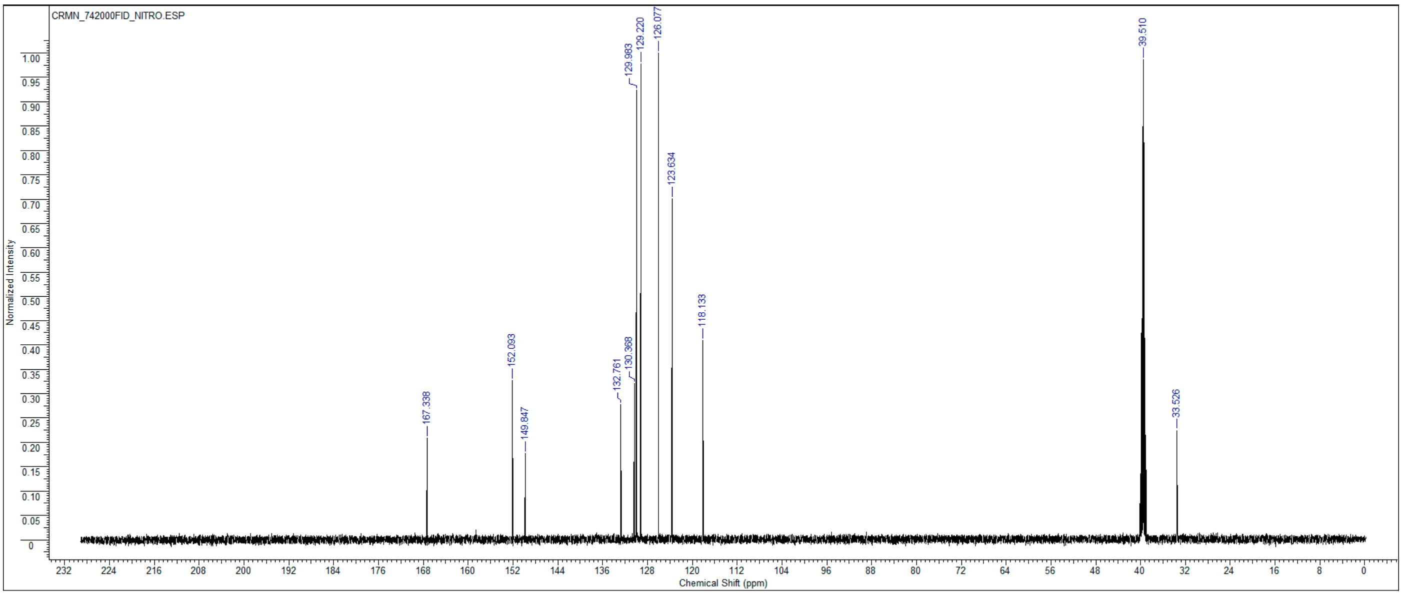

Figure S96. The ^{13}C-NMR spectrum for the compound **8b**.

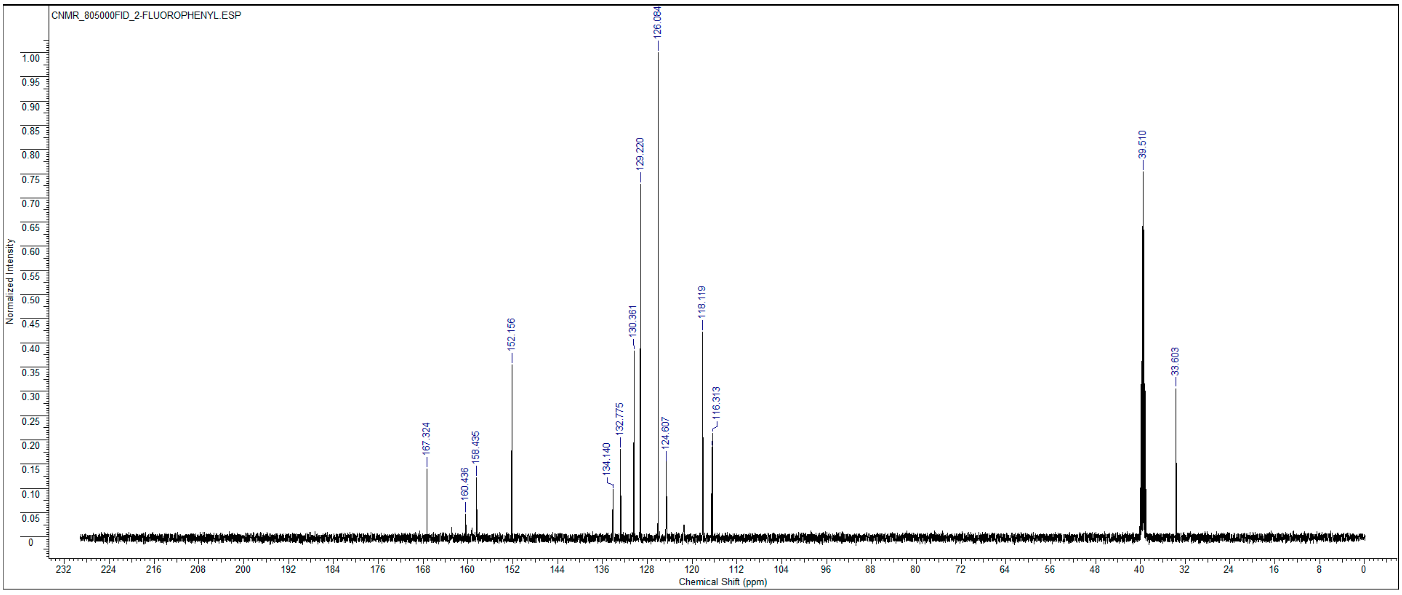

Figure S97. The ^{13}C-NMR spectrum for the compound **8c**.

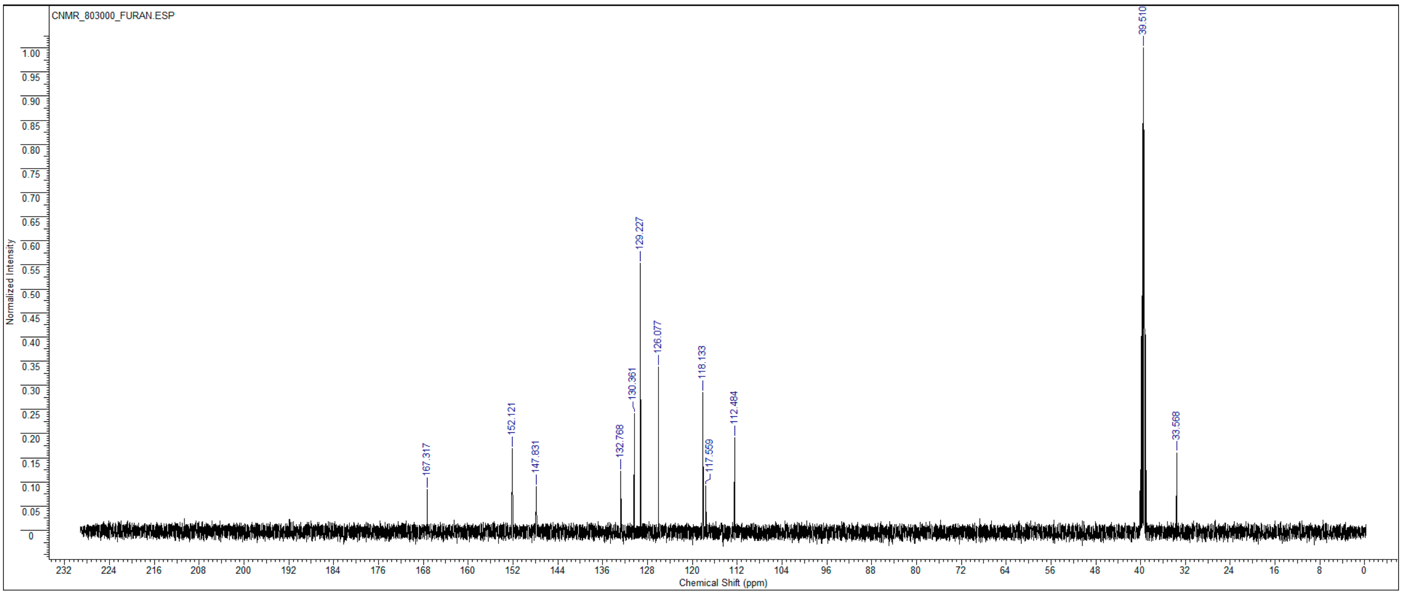

Figure S98. The ^{13}C-NMR spectrum for the compound **8d**.

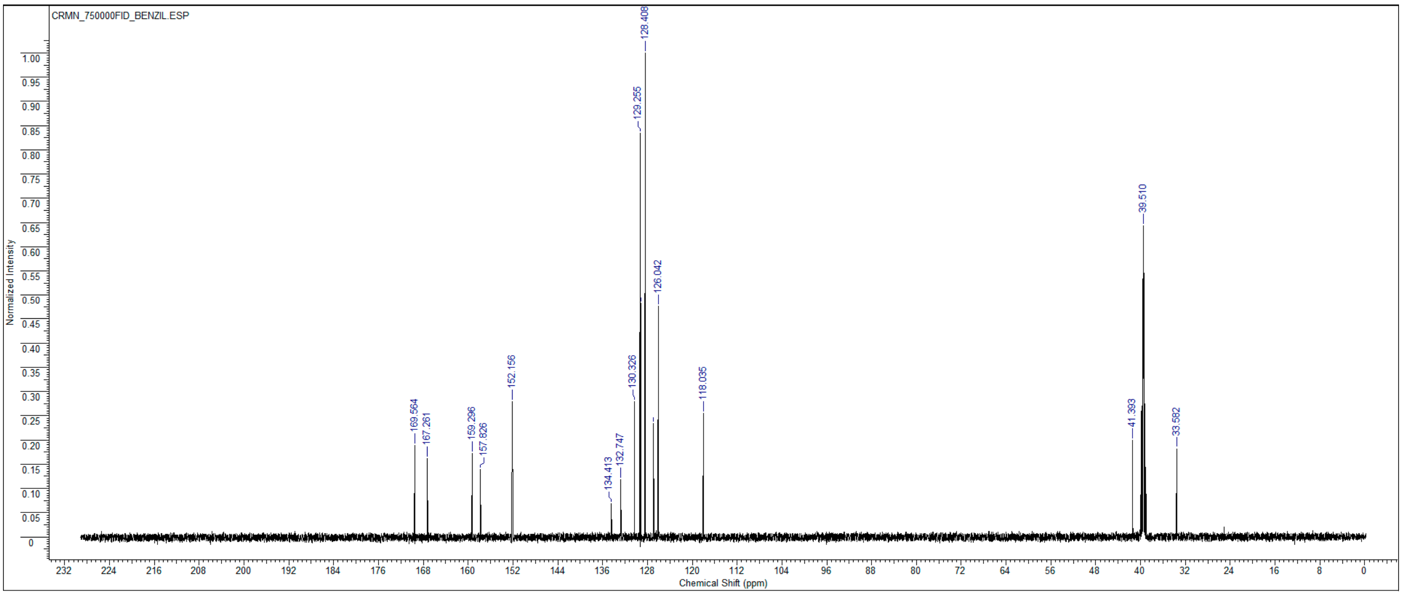

Figure S99. The ^{13}C-NMR spectrum for the compound **8e**.

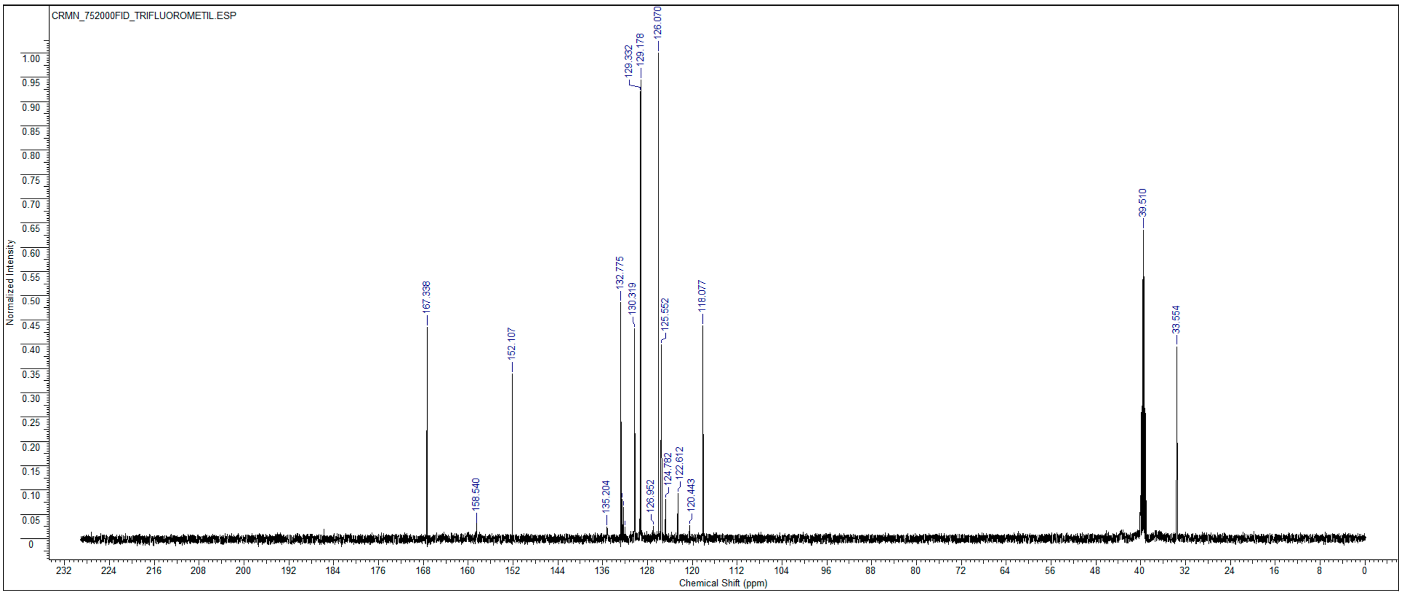

Figure S100. The ^{13}C-NMR spectrum for the compound **8f**.

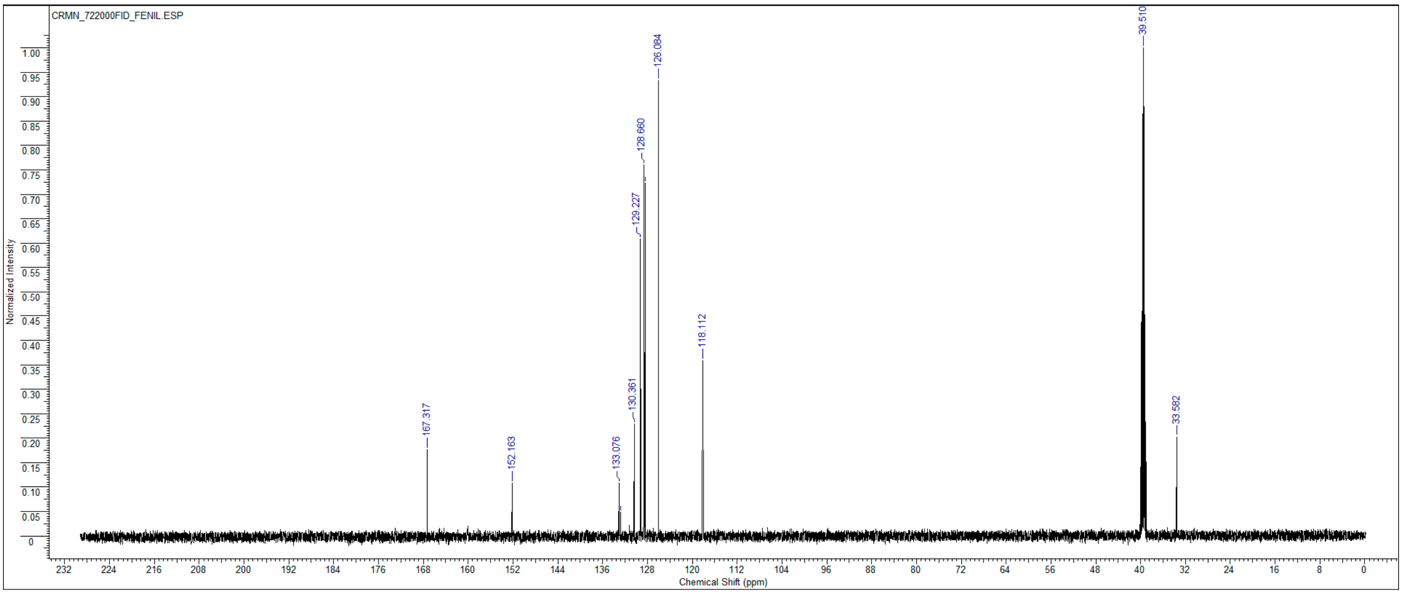

Figure S101. The ^{13}C-NMR spectrum for the compound **8g**.

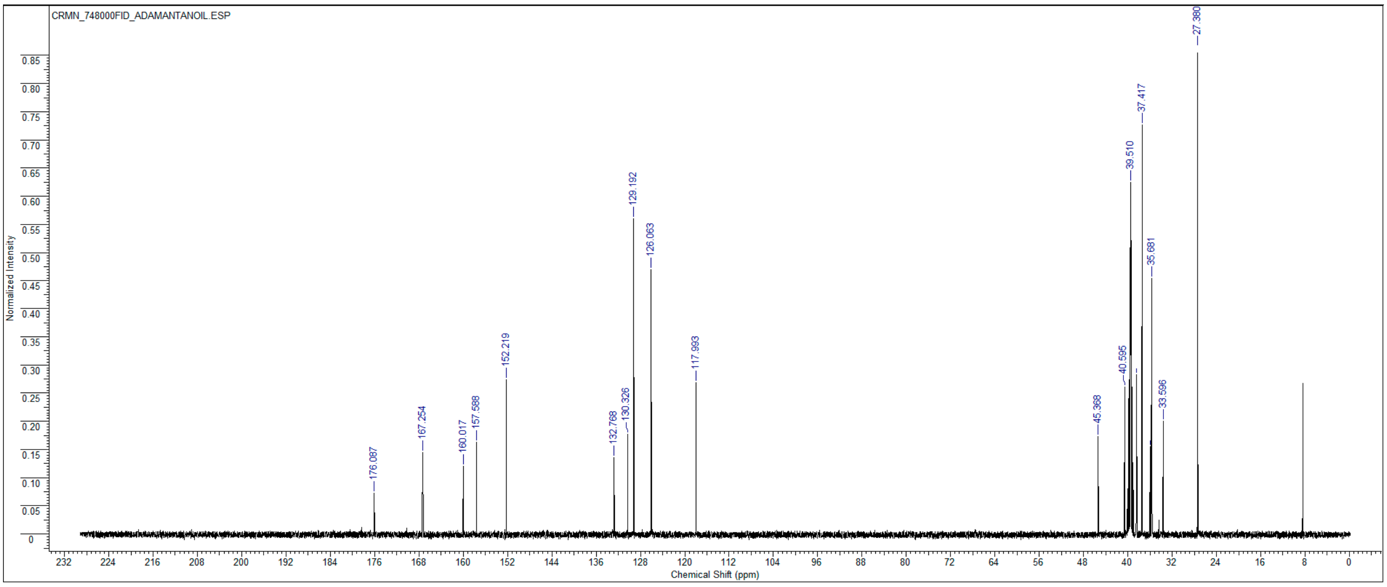

Figure S102. The ^{13}C-NMR spectrum for the compound **8h**.

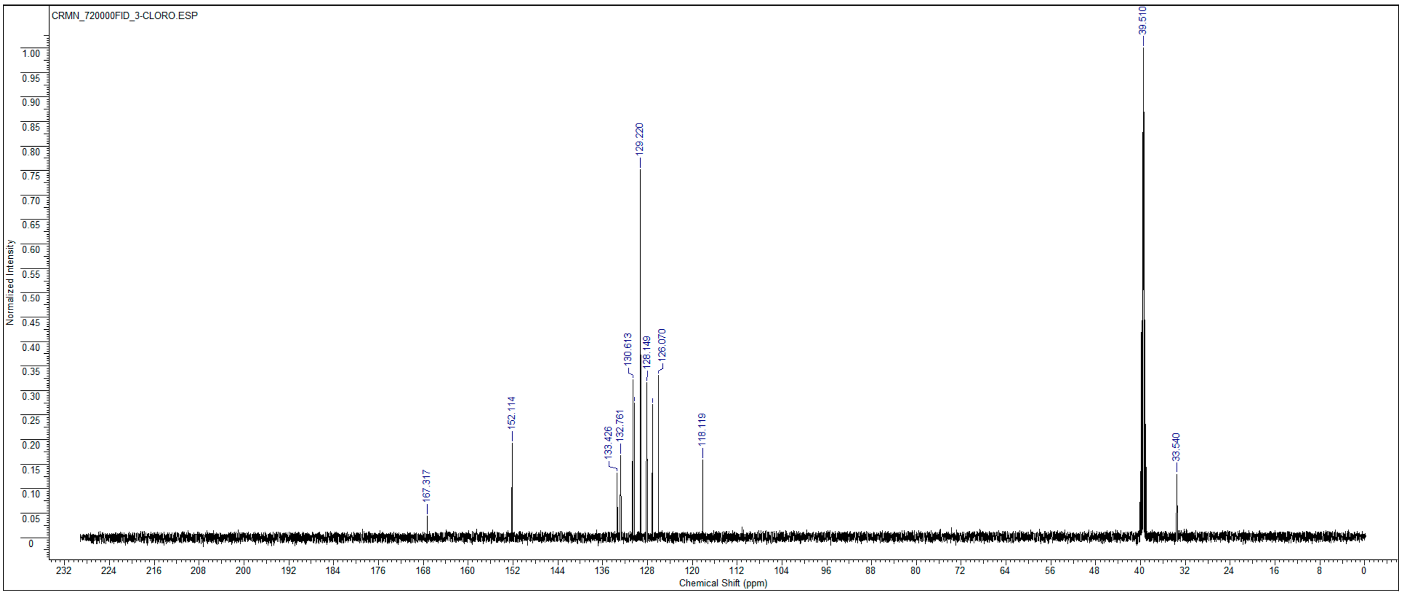

Figure S103. The ^{13}C-NMR spectrum for the compound **8i**.

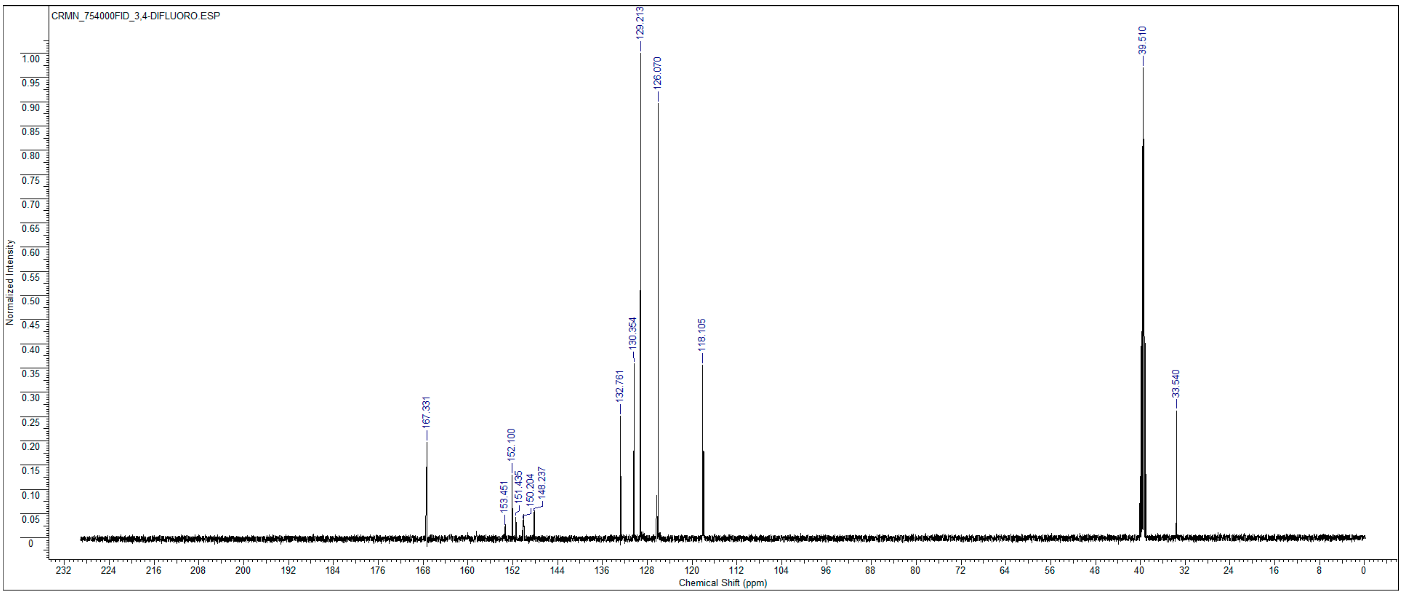

Figure S104. The ^{13}C-NMR spectrum for the compound **8j**.

1.5. Molecular Docking

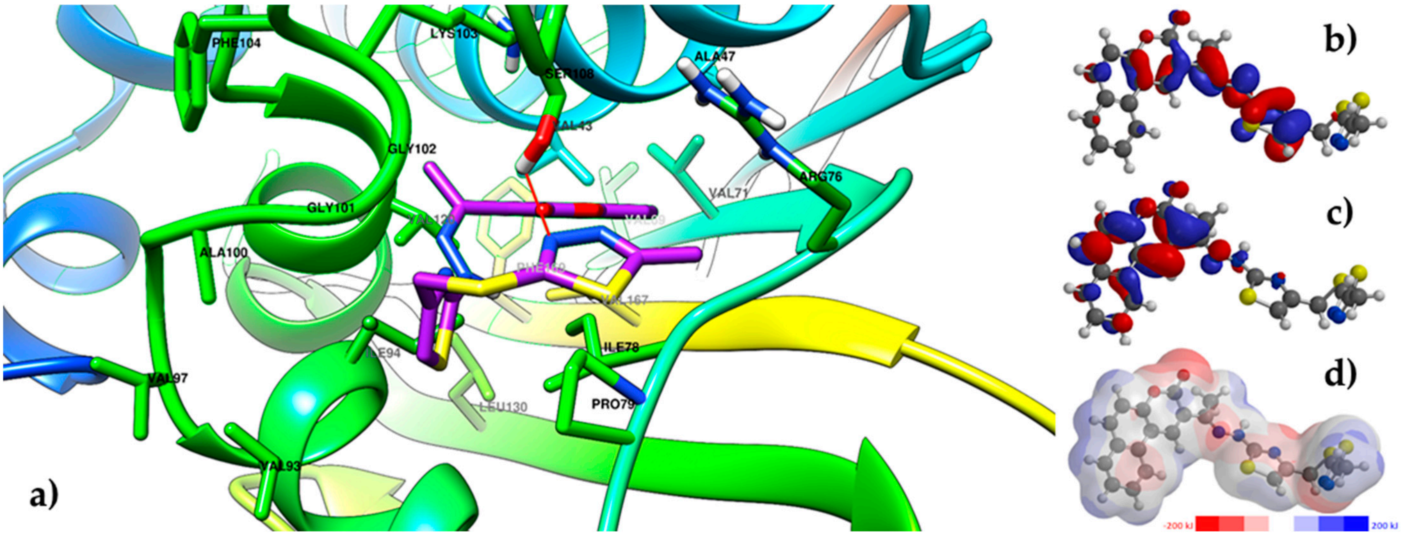

Figure S105. a) The predicted binding pose of compound **6i** in the ATPase domain of GyrB. The sidechain of Ser108 is predicted to act as a HBD to one of the nitrogen atoms of the thiadiazole heterocycle (red line), while the benzo[*f*]coumarin fits in a hydrophobic binding pocket comprised of Val71, Val69, Val43, Phe169, Val167, and Val120. The following coloring scheme was used: purple for carbon atoms, red for oxygen atoms, blue for nitrogen atoms, white for hydrogen atoms, and yellow for sulfur atoms; **b)** Graphical depiction of the localization of the HOMO frontier orbital in compound **6i**; **c)** Graphical depiction of the localization of the LUMO frontier orbital in compound **6i**; **d)** Graphical depiction of the electrostatic potential map of compound **6i**. Red represents the electron-rich regions, while blue represents the electron-depleted regions.

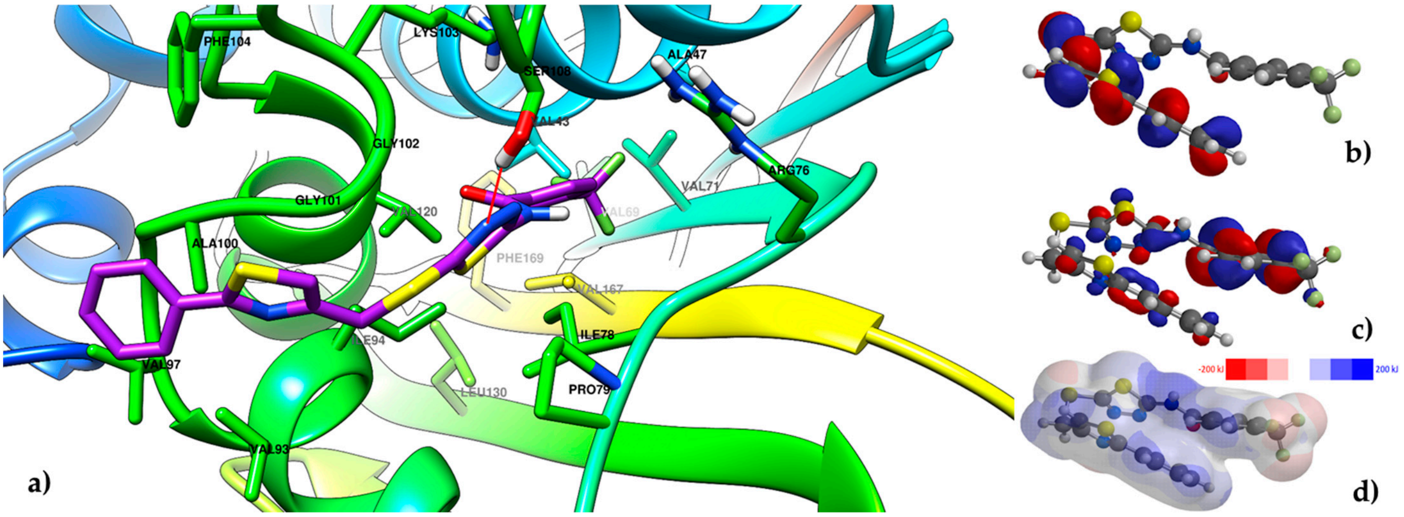

Figure S106. The predicted binding pose of compound **8f** in the ATPase domain of GyrB. The sidechain of Ser108 is predicted to act as a HBD (red line) to one of the nitrogen atoms of the thiadiazole heterocycle. The phenyl-thiazole (left side) is located in a hydrophobic region comprised of Val97, Val93, Ile94, Phe104, and Ala100. The following coloring scheme was used: purple for carbon atoms, red for oxygen atoms, blue for nitrogen atoms, white for hydrogen atoms, and yellow for sulfur atoms; **b)** Graphical depiction of the localization of the HOMO frontier orbital in compound **8f**; **c)** Graphical depiction of the localization of the LUMO frontier orbital in compound **8f**; **d)** Graphical depiction of the electrostatic potential map of compound **8f**. Red represents the electron-rich regions, while blue represents the electron-depleted regions.

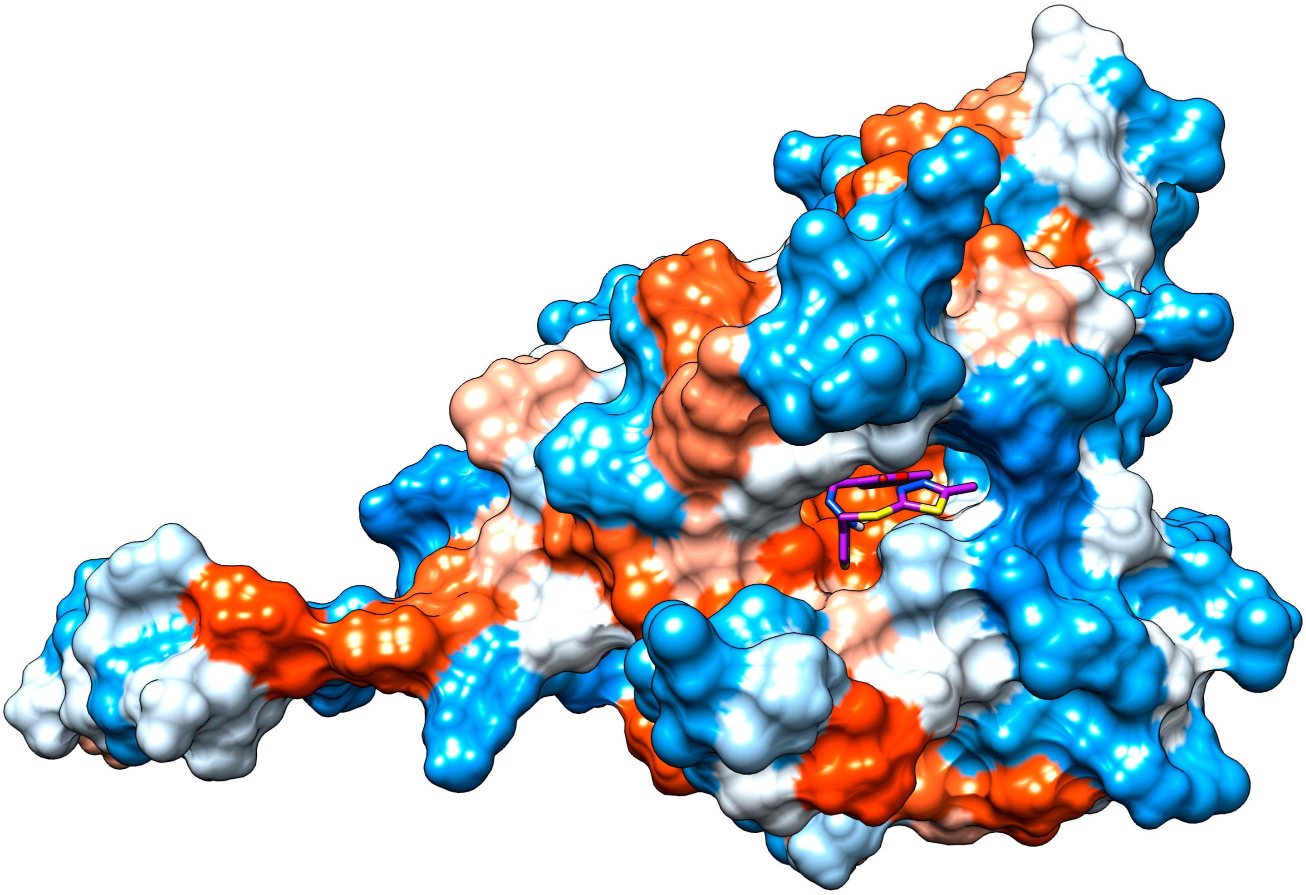

Figure S107. The graphical depiction of compound **6i** in the active site of the ATPase domain of GyrB displayed as surfaces.

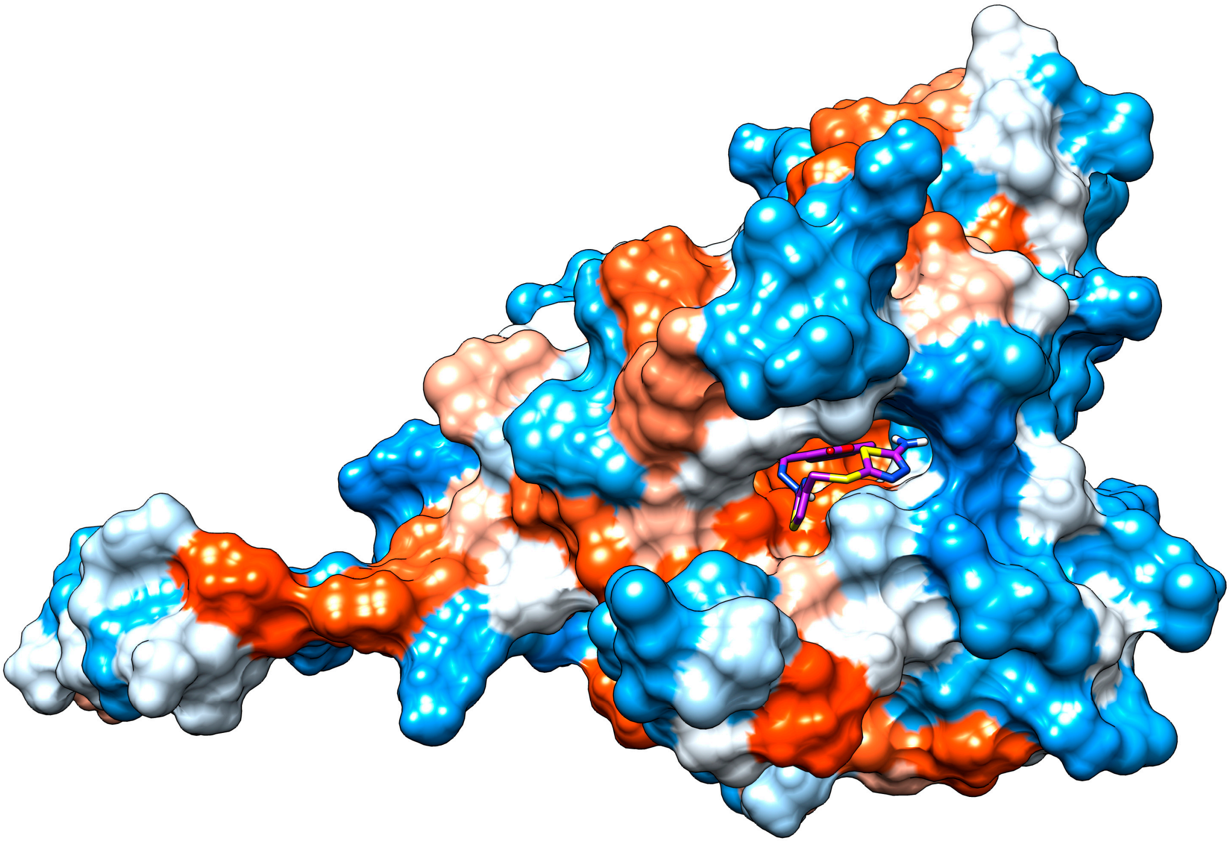

Figure S108. The graphical depiction of compound **6j** in the active site of the ATPase domain of GyrB displayed as surfaces.

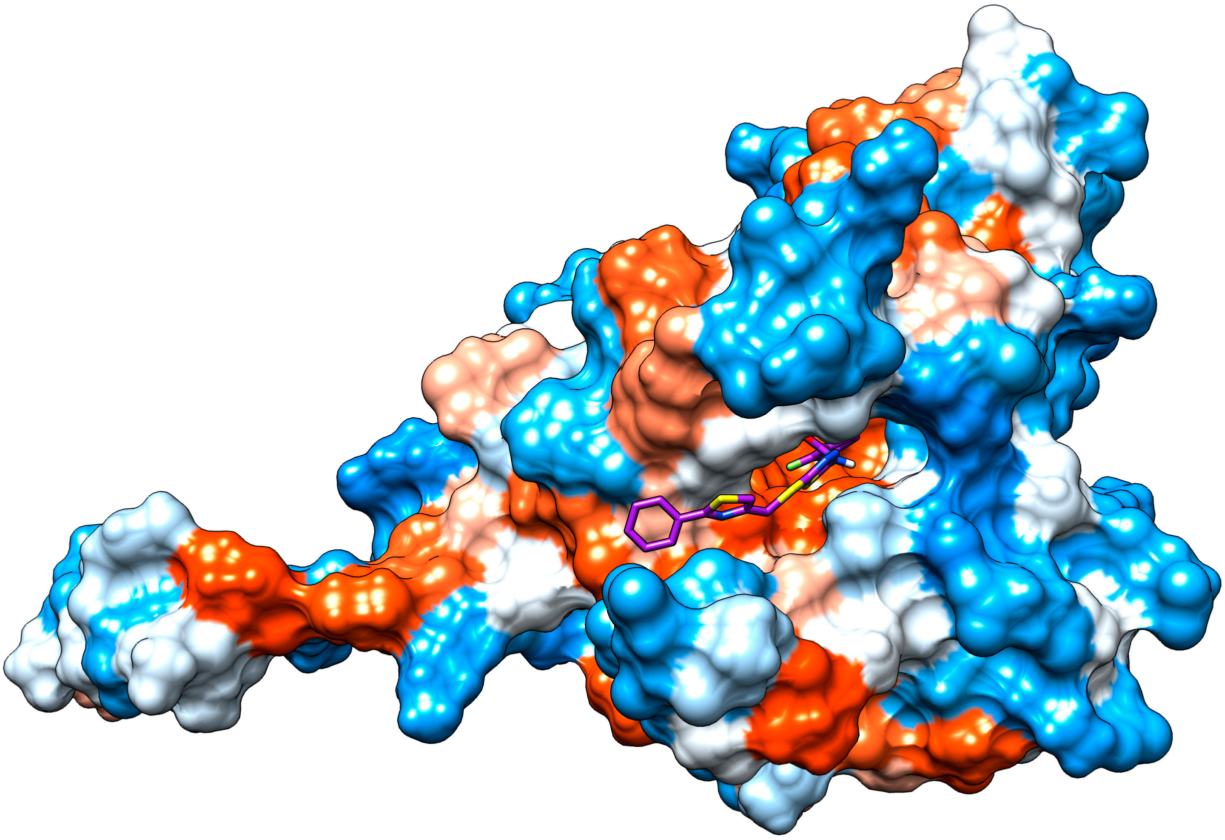

Figure S109. The graphical depiction of compound **8c** in the active site of the ATPase domain of GyrB displayed as surfaces.

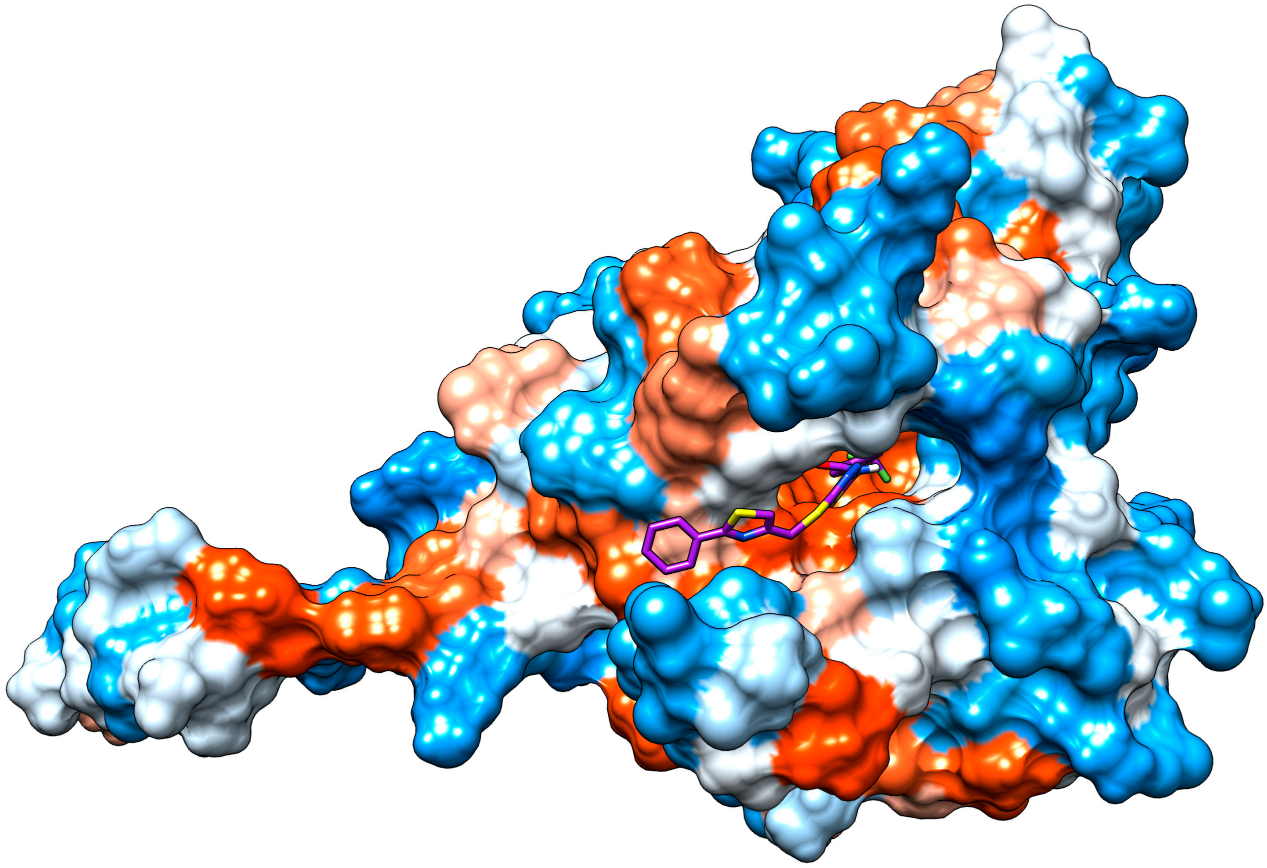

Figure S110. The graphical depiction of compound **8f** in the active site of the ATPase domain of GyrB displayed as surfaces.

S2. Tables

S2.1. ADMETox Predictions

Table S1. The computed in silico pharmacokinetic descriptors of compounds **6a-j** and **8a-j**. The prediction was done using SwissADME web tool.

Compound	GI absorption	BBB permeation	P-gp substrate	CYP1A2 inhibitor	CYP2C19 inhibitor	CYP2C9 inhibitor	CYP2D6 inhibitor	CYP3A4 inhibitor
**6a**	Low	No	No	No	Yes	Yes	No	Yes
**6b**	Low	No	No	No	Yes	Yes	No	Yes
**6c**	Low	No	No	No	Yes	Yes	No	Yes
**6d**	Low	No	No	No	No	Yes	No	Yes
**6e**	Low	No	No	No	Yes	Yes	No	Yes
**6f**	Low	No	No	No	Yes	Yes	No	Yes
**6g**	Low	No	No	No	Yes	Yes	No	Yes
**6h**	Low	No	No	No	No	Yes	No	Yes
**6i**	Low	No	No	No	Yes	Yes	No	Yes
**6j**	Low	No	No	No	Yes	Yes	No	Yes
**8a**	Low	No	No	No	Yes	Yes	Yes	Yes
**8b**	Low	No	No	No	Yes	Yes	No	Yes
**8c**	Low	No	No	Yes	Yes	Yes	Yes	Yes

**8d**	Low	No	No	Yes	Yes	Yes	Yes	Yes
**8e**	Low	No	No	Yes	Yes	Yes	Yes	Yes
**8f**	Low	No	Yes	No	Yes	Yes	Yes	Yes
**8g**	Low	No	No	Yes	Yes	Yes	Yes	Yes
**8h**	Low	No	Yes	No	Yes	Yes	Yes	Yes
**8i**	Low	No	No	Yes	Yes	Yes	Yes	Yes
**8j**	Low	No	No	Yes	Yes	Yes	Yes	Yes

Table S2. The computed in silico toxicologic descriptors of compounds **6a-j** and **8a-j**. The prediction was done using admetSAR 3.0 web tool and Toxtree 3.1.0 software.

**Compound**	**Carcinogenicity**	**Eye irritation**	**Skin irritation**	**Hepatotoxicity**	**Respiratory toxicity**	**Reproductive toxicity**	**Nephrotoxicity**	**Acute oral toxicity**
**6a**	No	Yes	No	Yes	No	No	Yes	Class III
**6b**	No	Yes	No	Yes	No	No	Yes	Class III
**6c**	No	Yes	No	Yes	No	No	Yes	Class III
**6d**	No	Yes	No	Yes	No	No	Yes	Class III
**6e**	No	Yes	No	Yes	No	No	Yes	Class III
**6f**	No	Yes	No	Yes	No	No	Yes	Class III
**6g**	No	Yes	No	Yes	No	No	Yes	Class III
**6h**	No	Yes	No	Yes	No	No	Yes	Class III
**6i**	No	Yes	No	Yes	No	No	Yes	Class III
**6j**	No	Yes	No	Yes	No	No	Yes	Class III
**8a**	No	No	No	Yes	No	Yes	No	Class III
**8b**	Yes	No	No	Yes	No	No	Yes	Class III
**8c**	No	No	No	Yes	No	No	Yes	Class III
**8d**	No	Yes	No	Yes	No	No	Yes	Class III
**8e**	No	No	No	Yes	No	No	No	Class III
**8f**	No	No	No	Yes	No	No	Yes	Class III
**8g**	No	Yes	No	Yes	No	No	Yes	Class III
**8h**	No	No	No	Yes	Yes	Yes	No	Class III
**8i**	No	Yes	No	Yes	No	No	Yes	Class III
**8j**	No	Yes	No	Yes	No	No	Yes	Class III

S2.2. Density Functional Theory Calculations

Table S3. Graphical depictions of the localization of the HOMO and LUMO frontier molecular orbitals in compounds **6a-j** and **8a-j**.

**Comp.**	**HOMO**	**LUMO**
6a	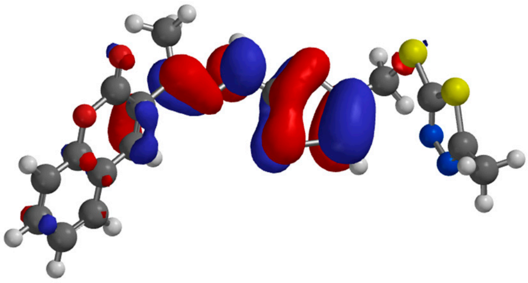	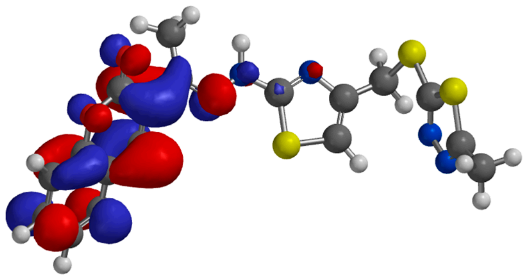

**6b**	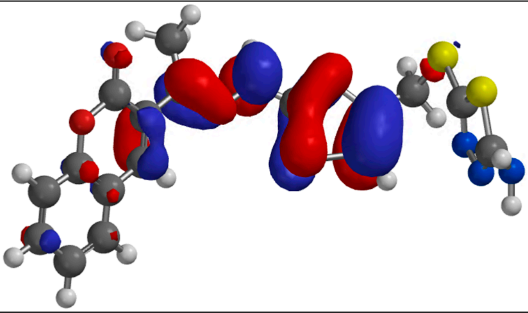	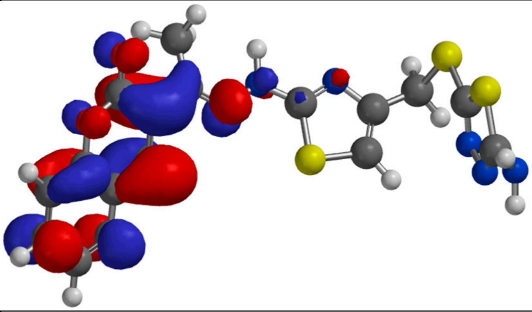
**6c**	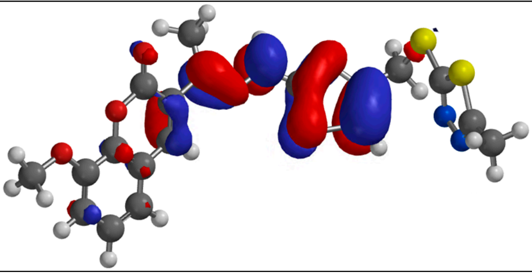	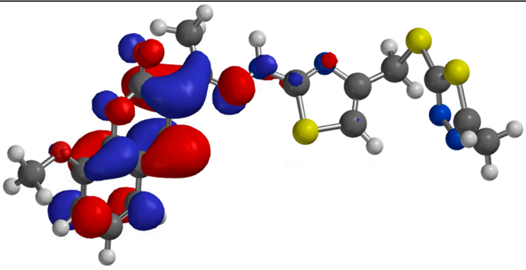
**6d**	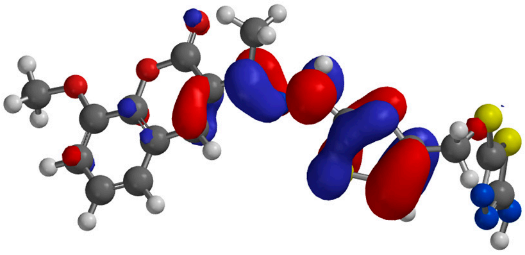	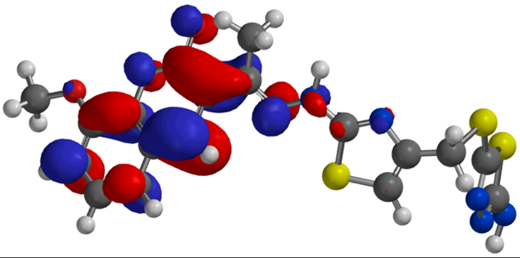
**6e**	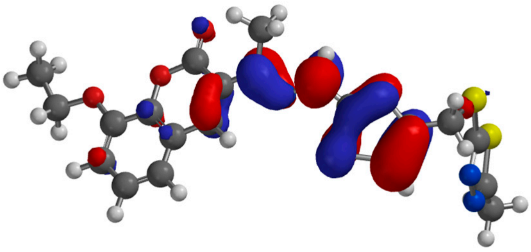	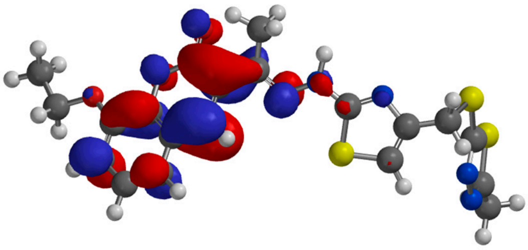
**6f**	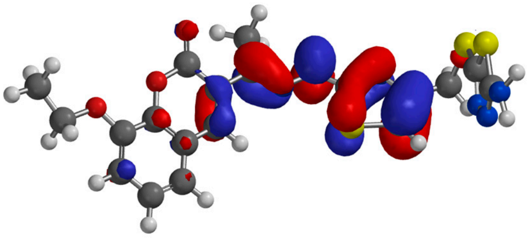	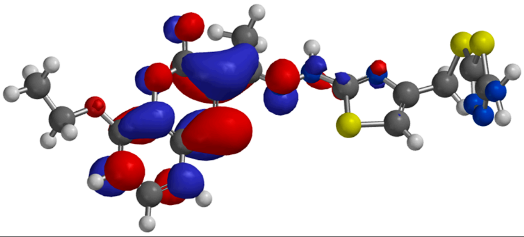
**6g**	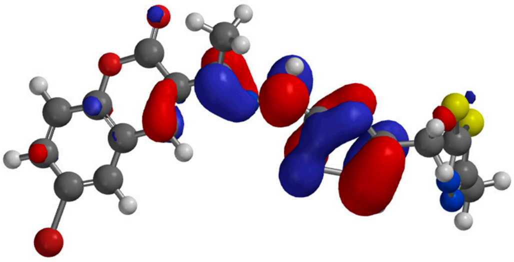	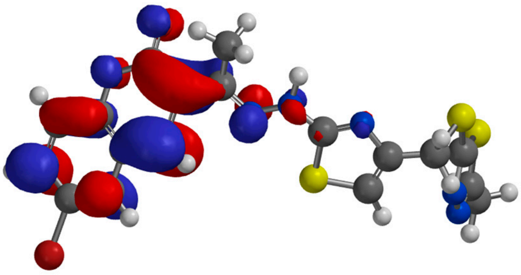

**6h**	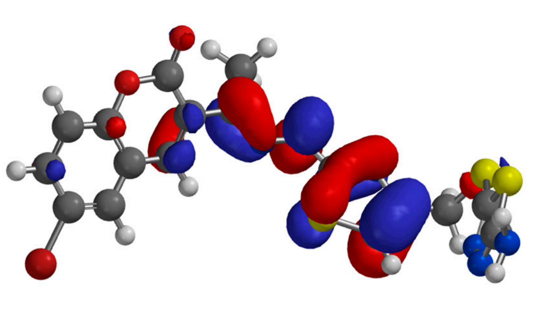	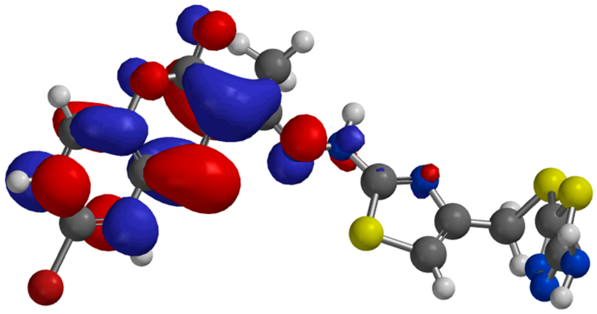
**6i**	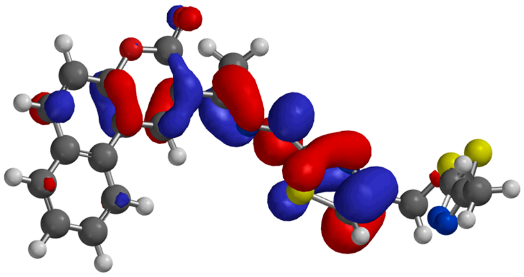	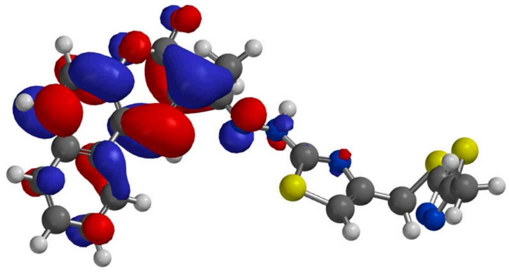
**6j**	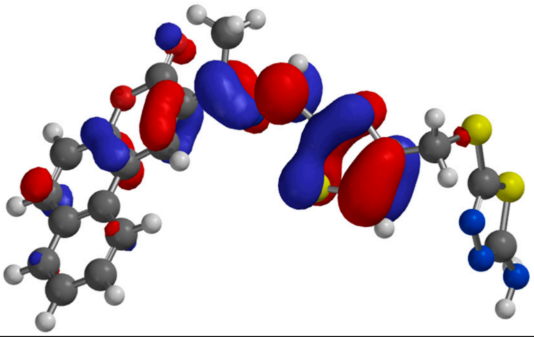	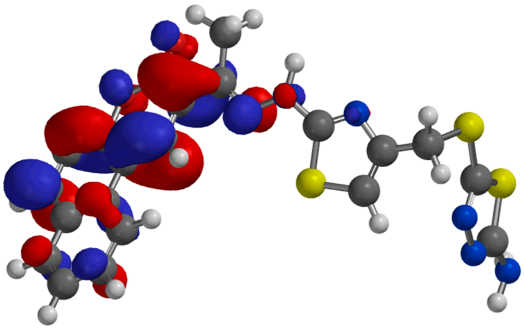
**8a**	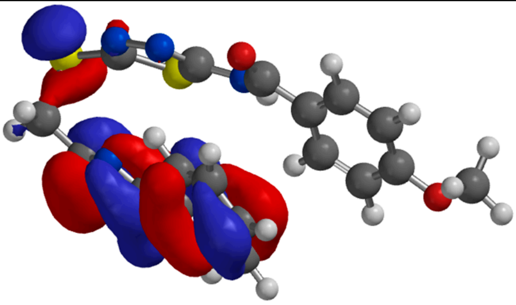	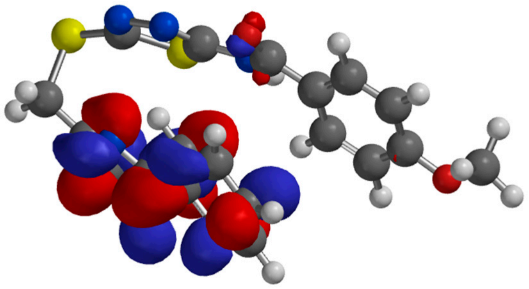
**8b**	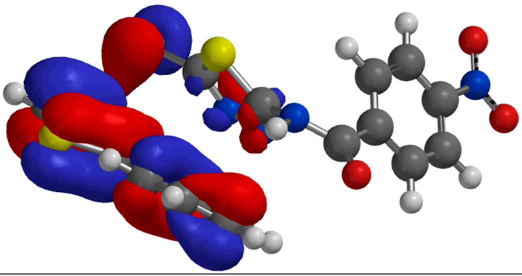	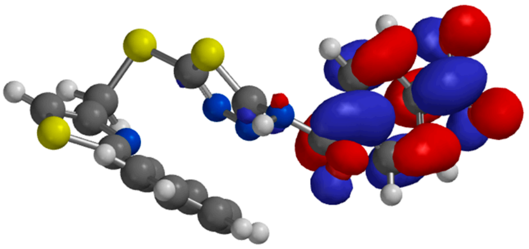

**8c**	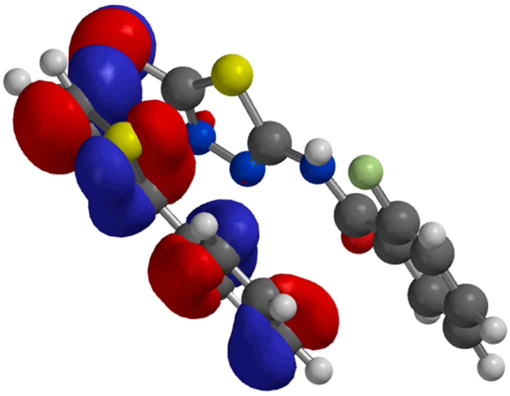	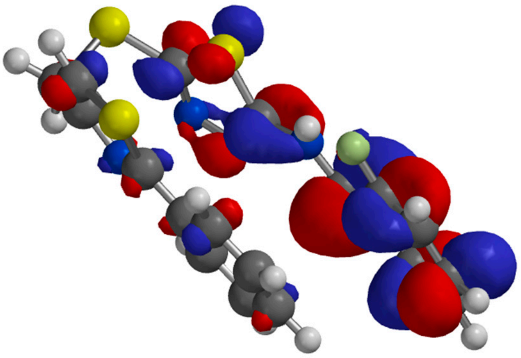
**8d**	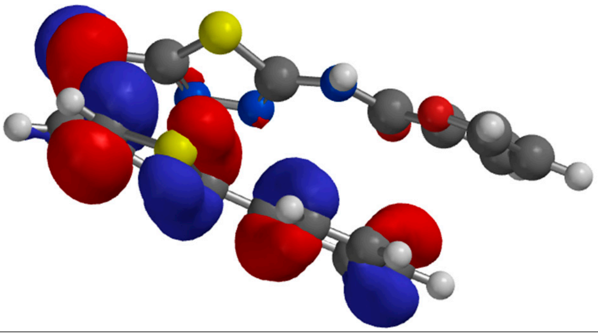	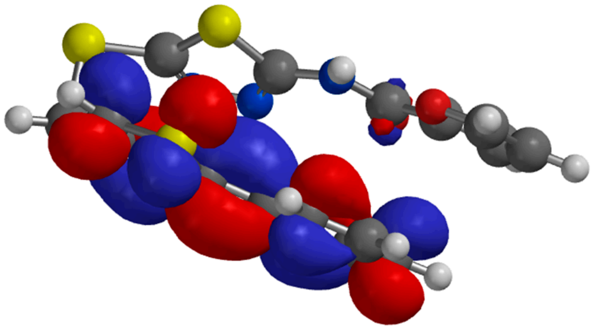
**8e**	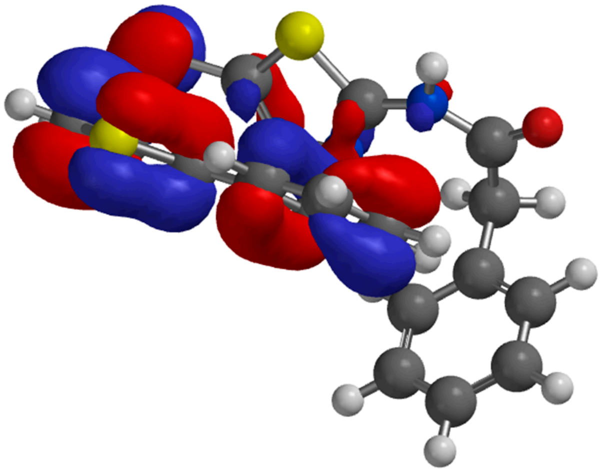	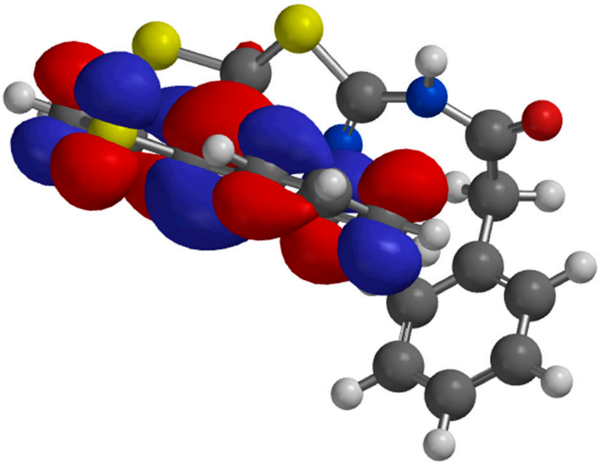
**8f**	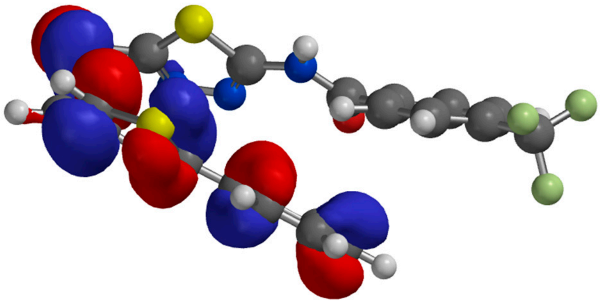	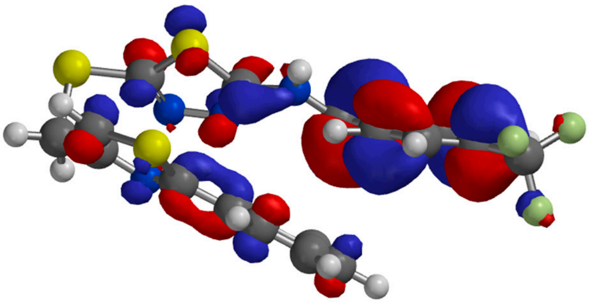

**8g**	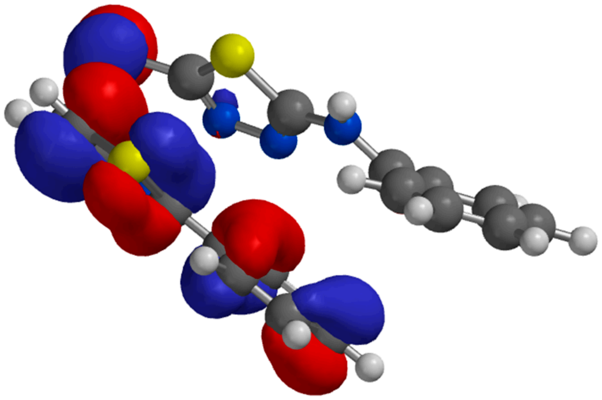	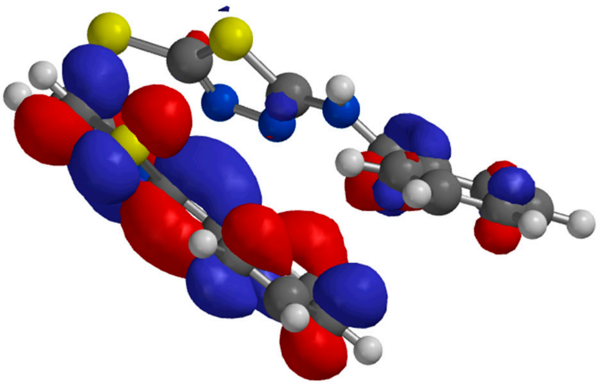
**8h**	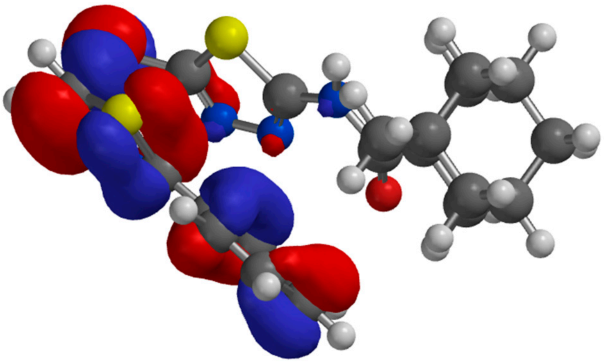	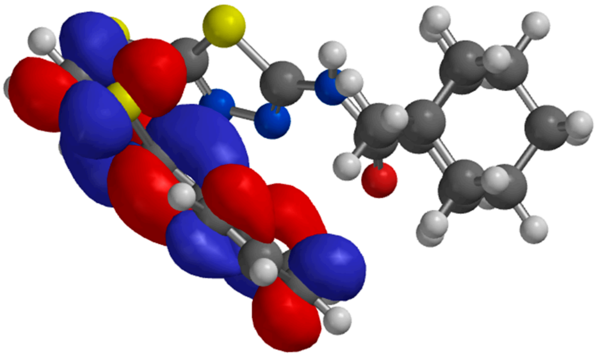
**8i**	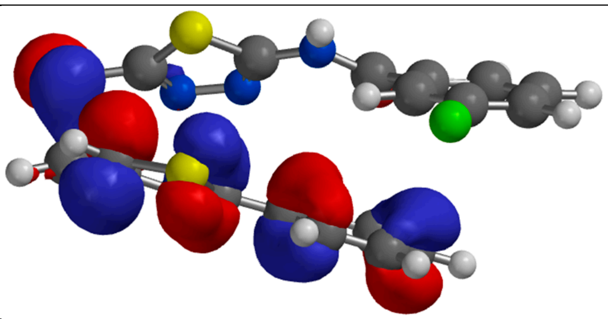	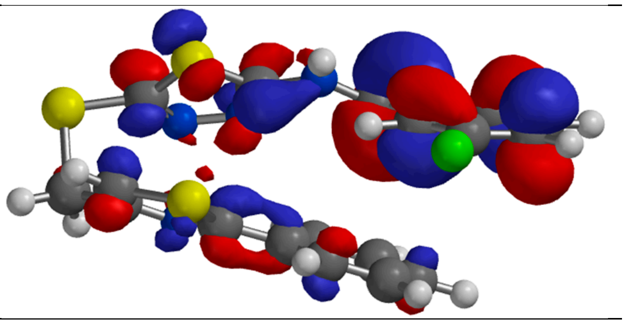
**8j**	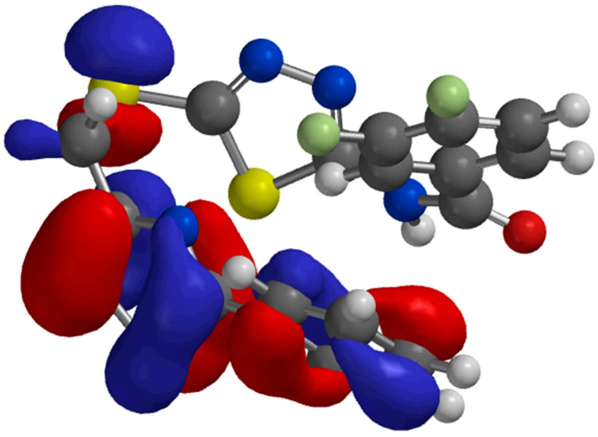	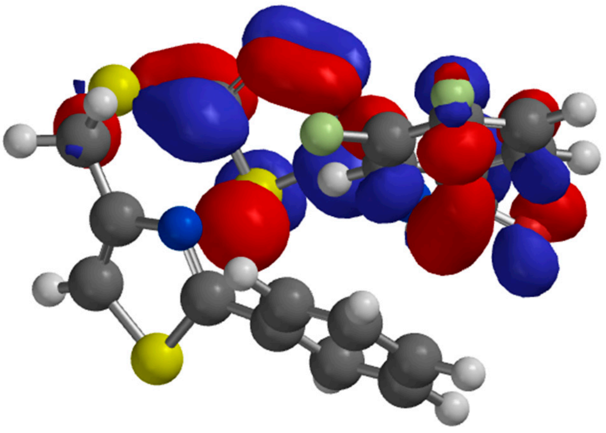

Table S4. Graphical depictions of the electrostatic potential maps of compounds **6a-j** and **8a-j**. Red represents the electron-rich regions, while blue represents the electron-depleted regions.

**Comp.**	**Electrostatic potential map**
**6a**	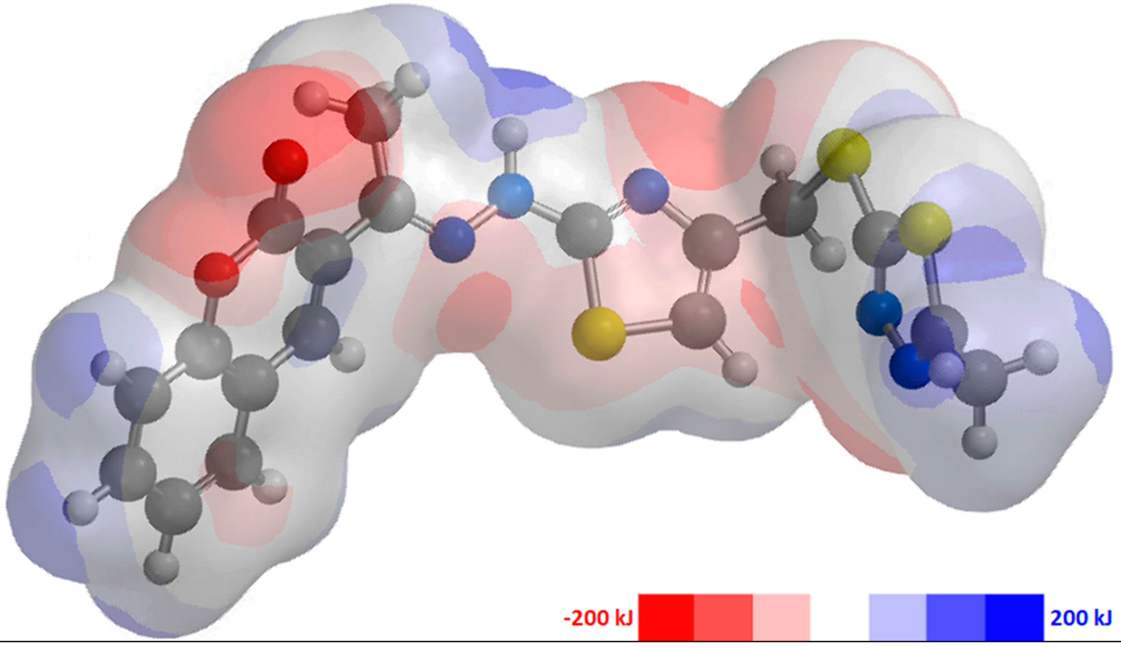
**6b**	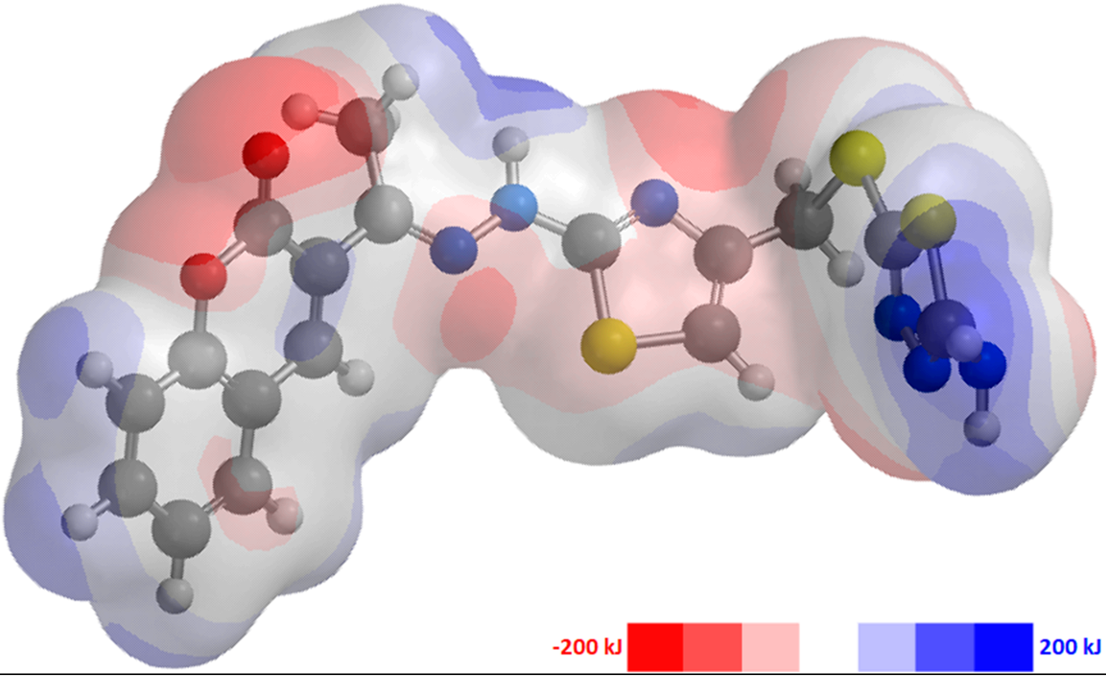

**6c**	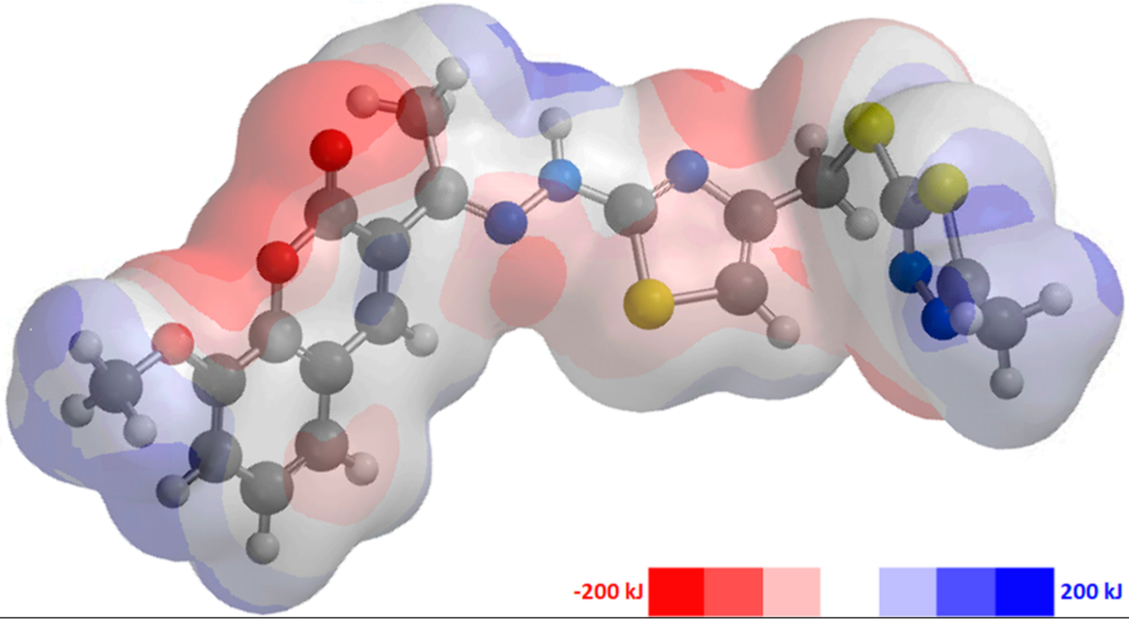
**6d**	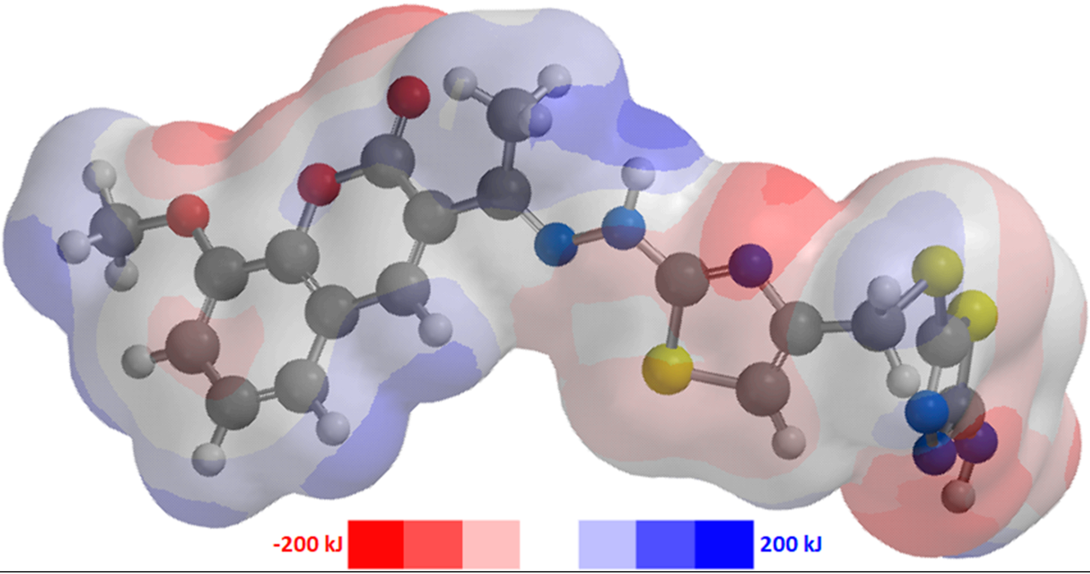
**6e**	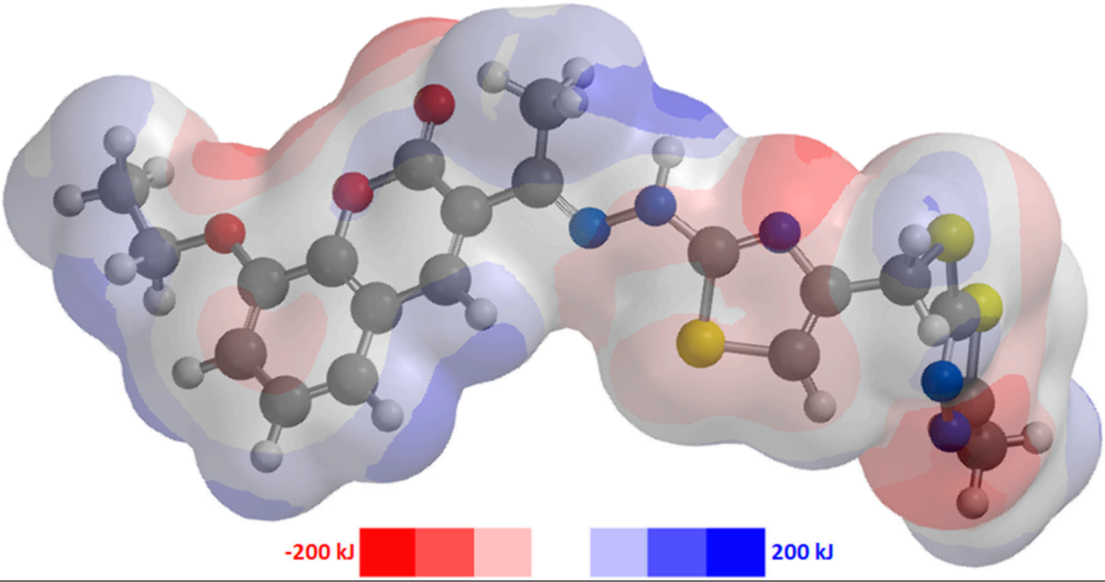

6f

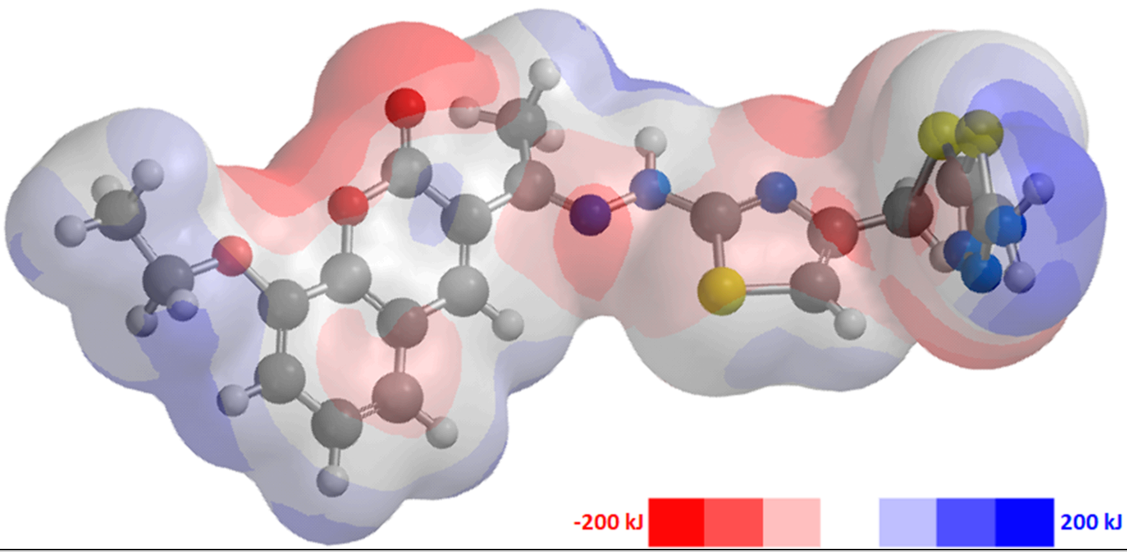

6g

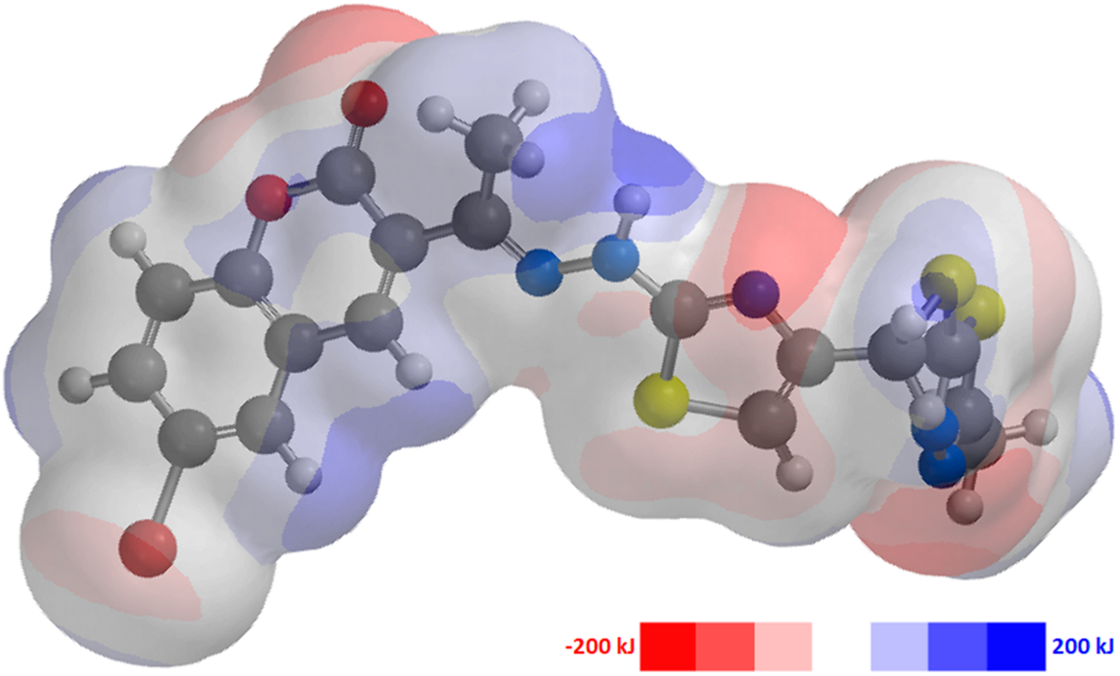

6h

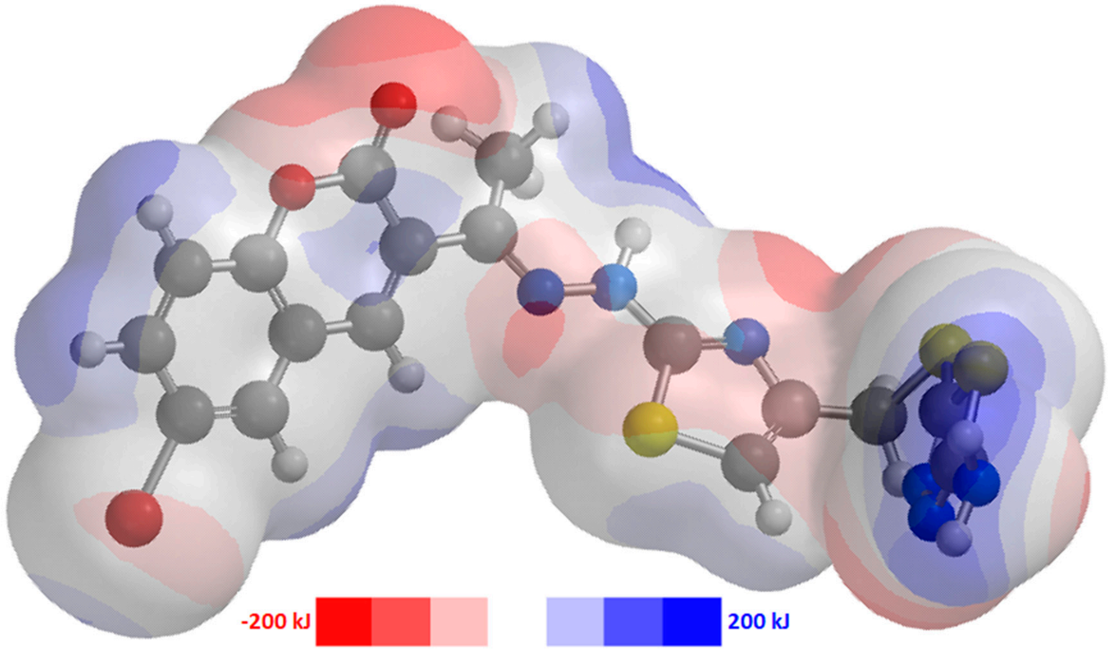

6i

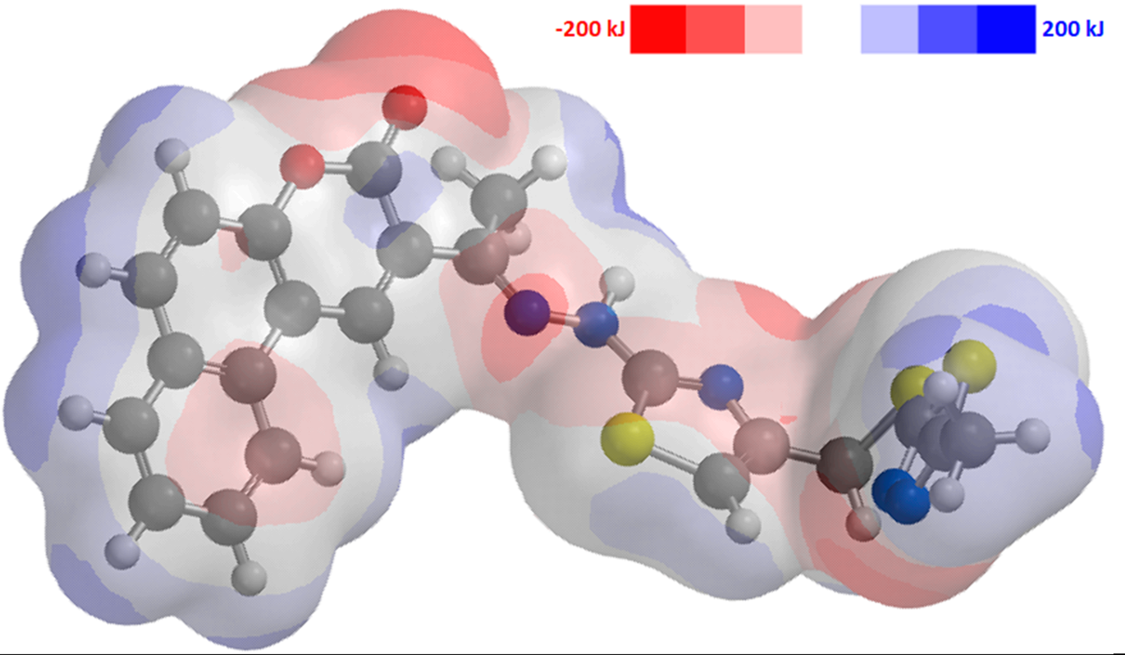

6j

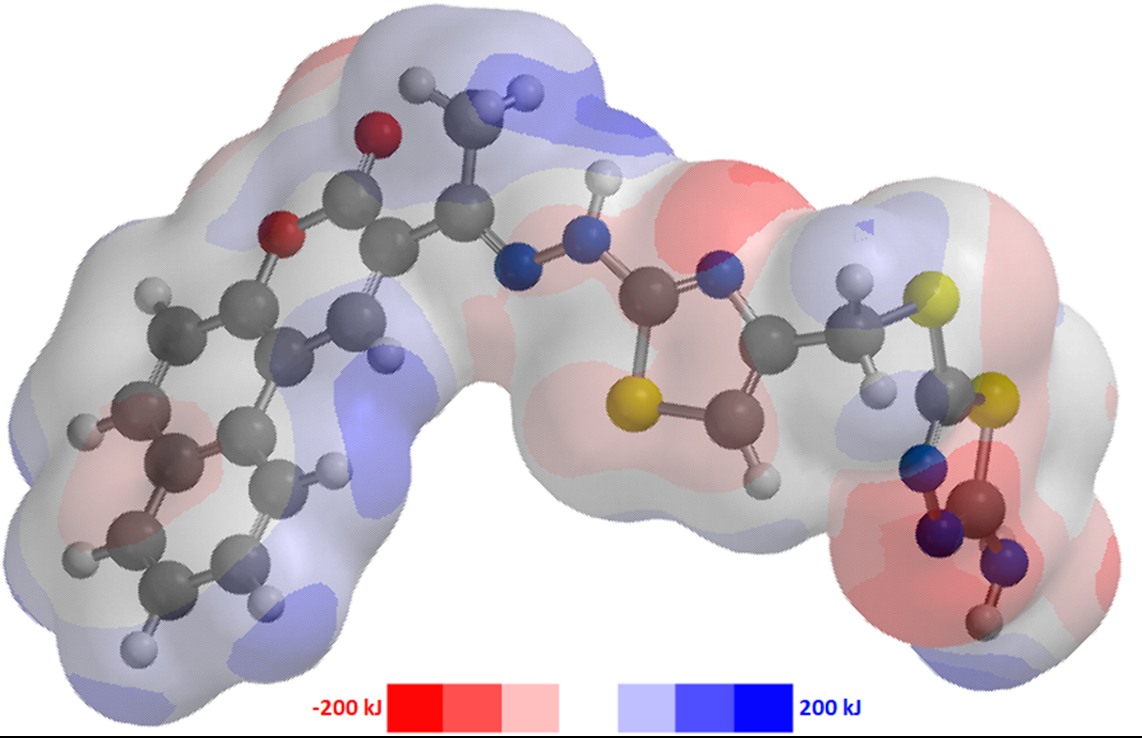

8a

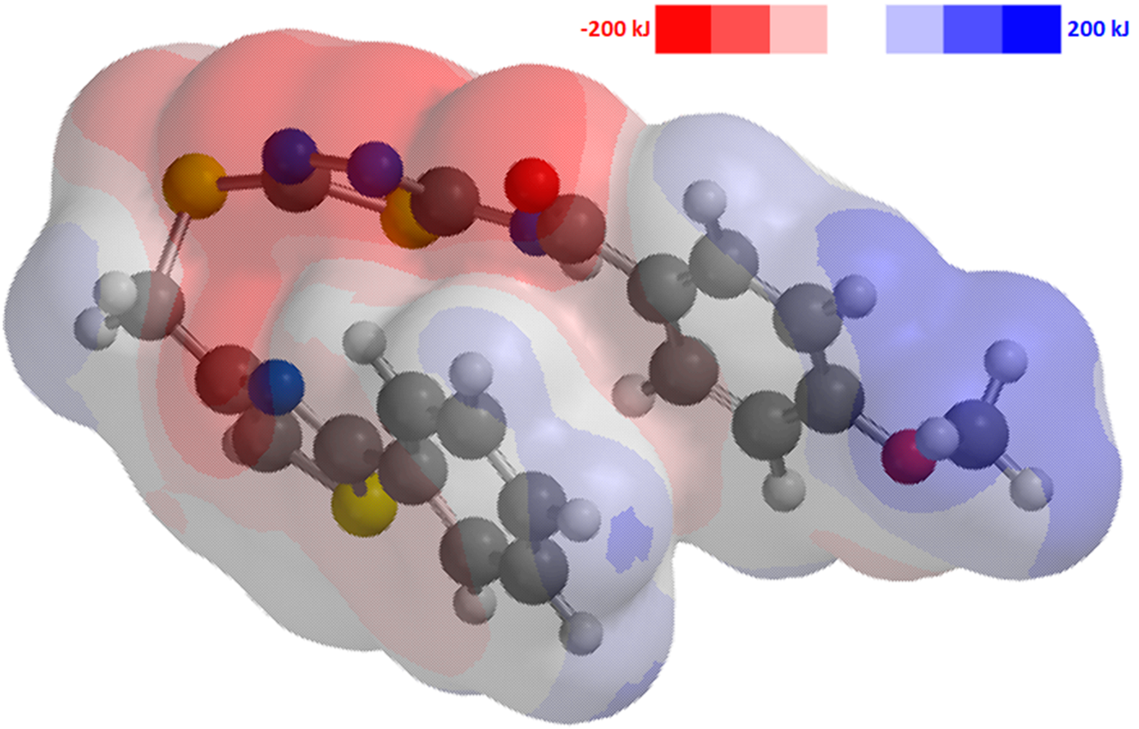

8b

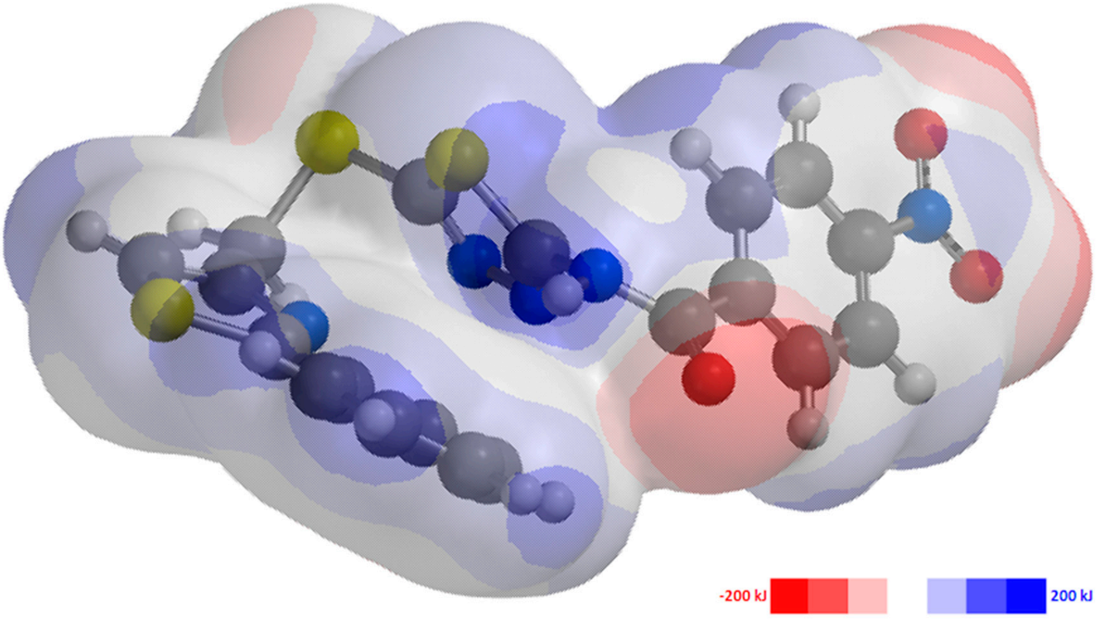

8c

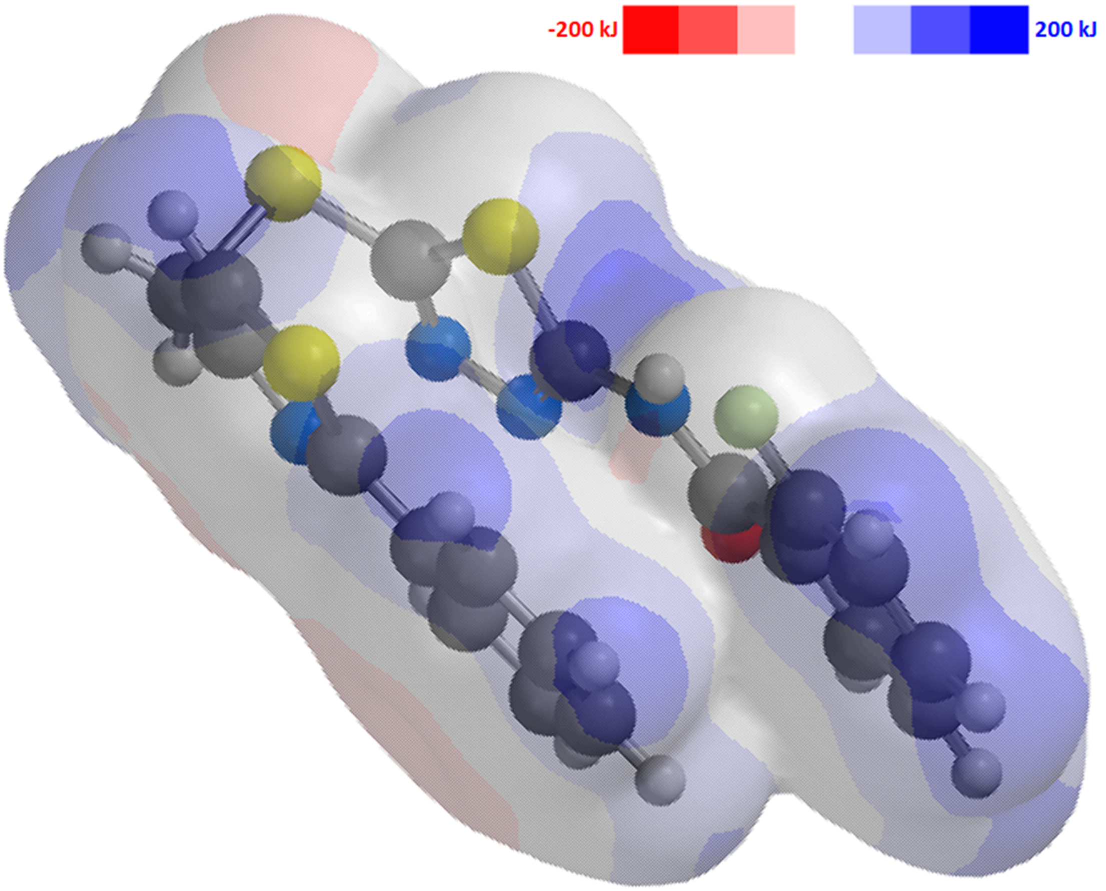

8d

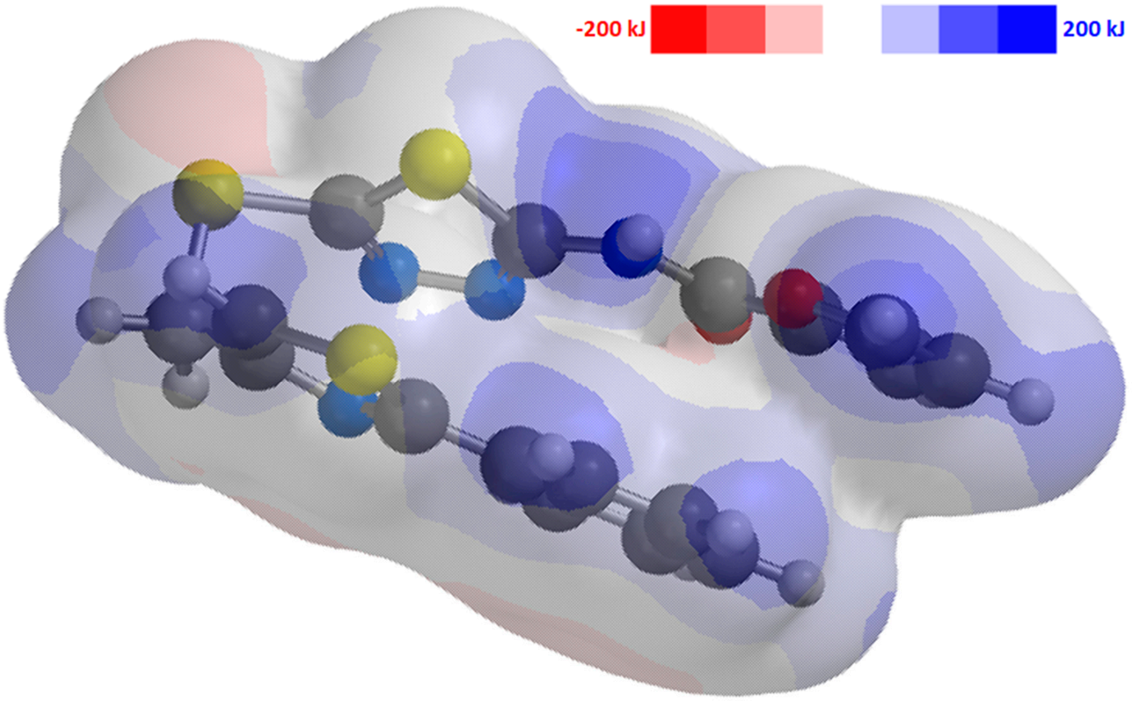

8e

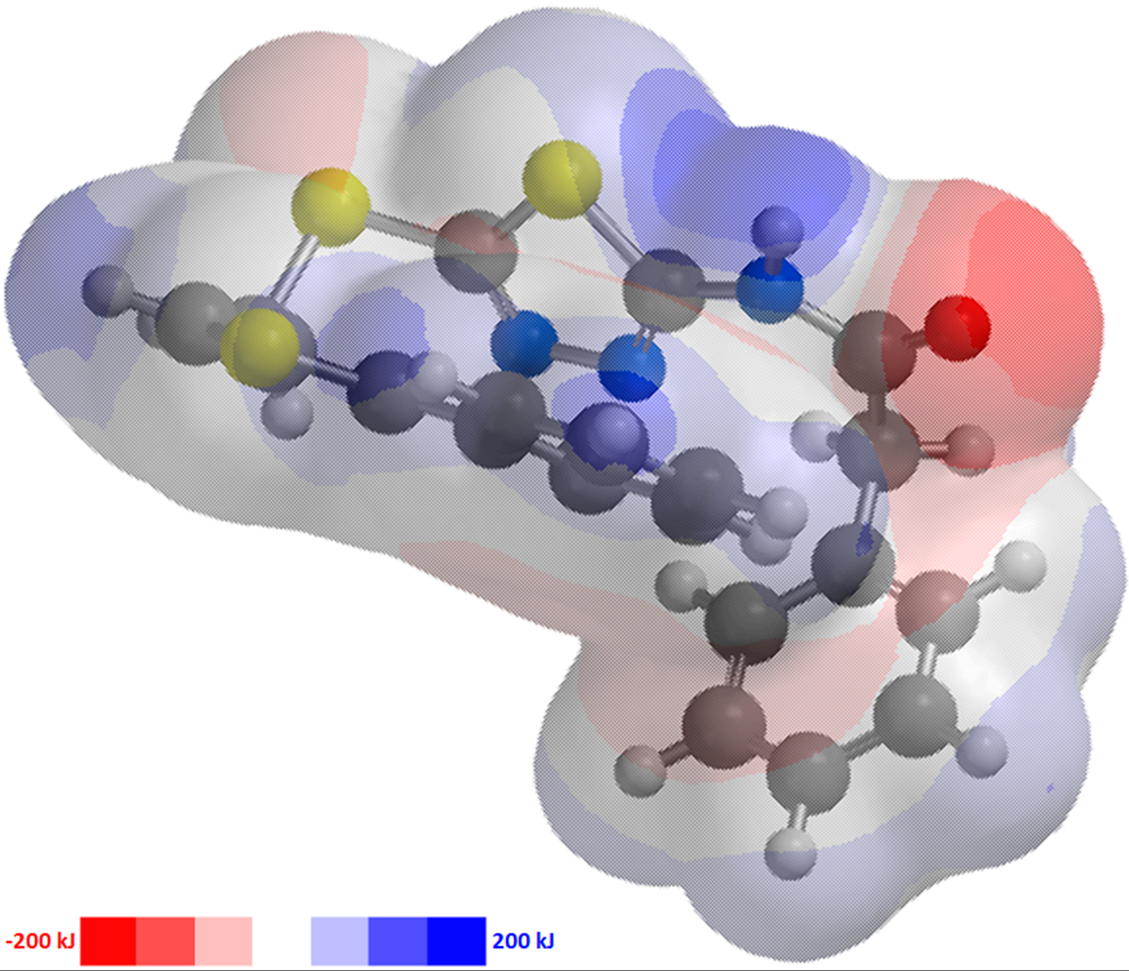

8f

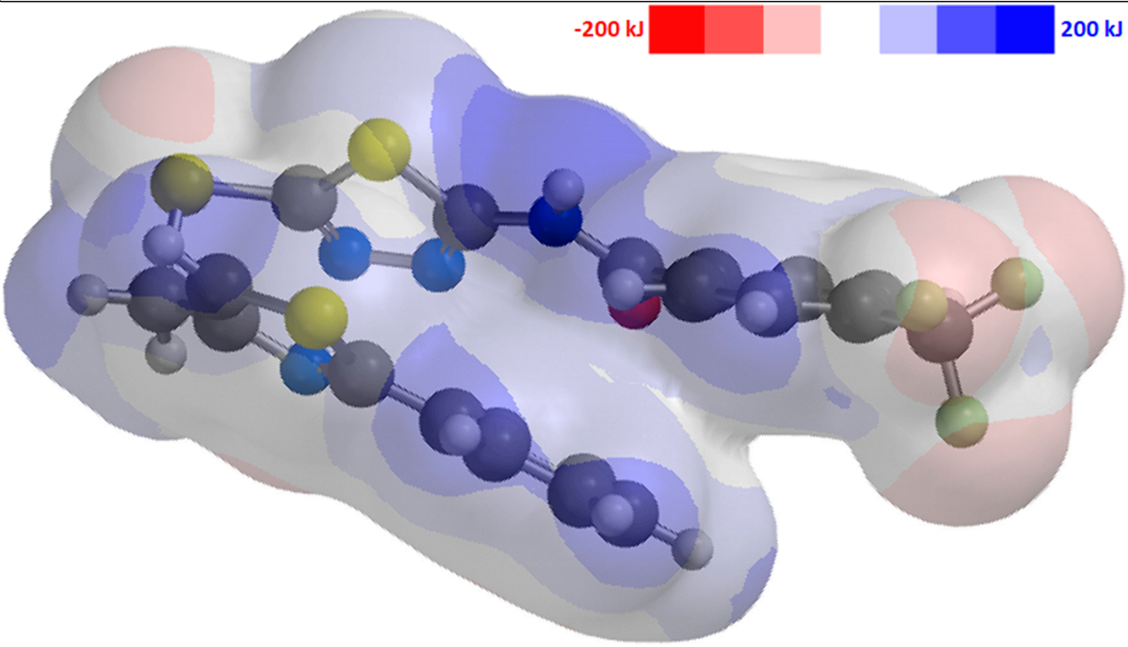

8g

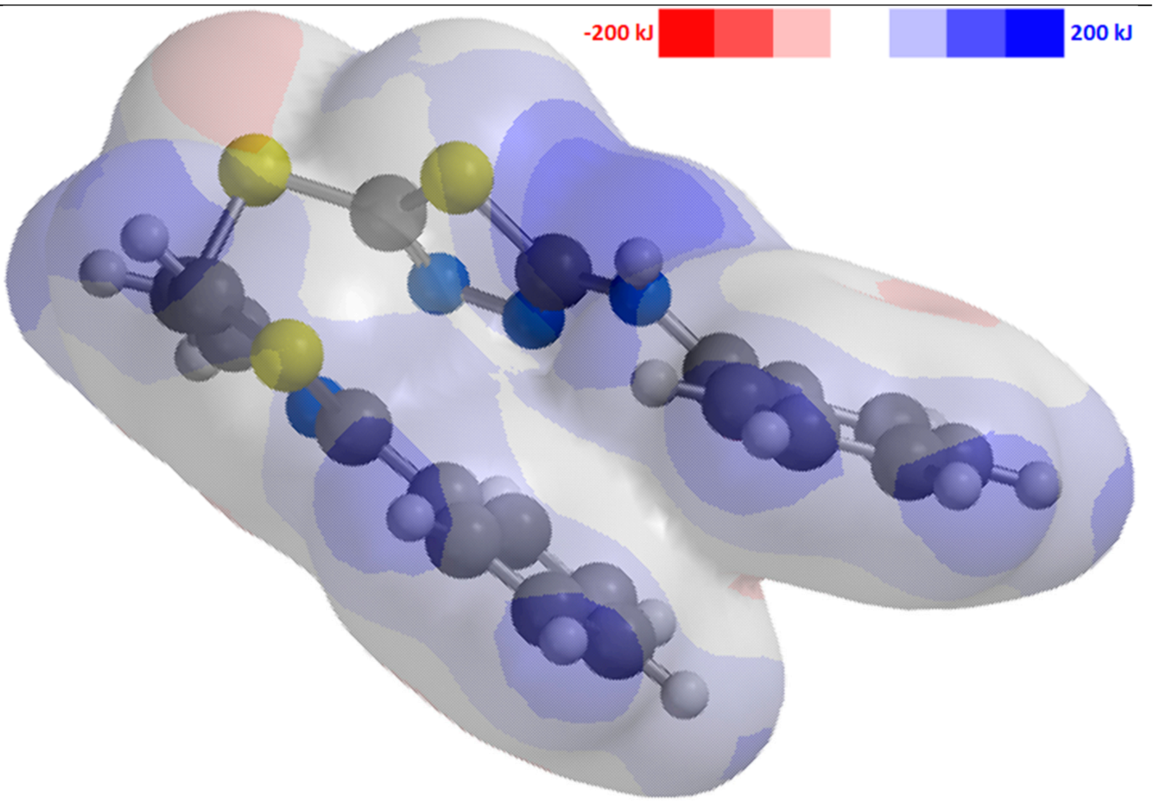

8h

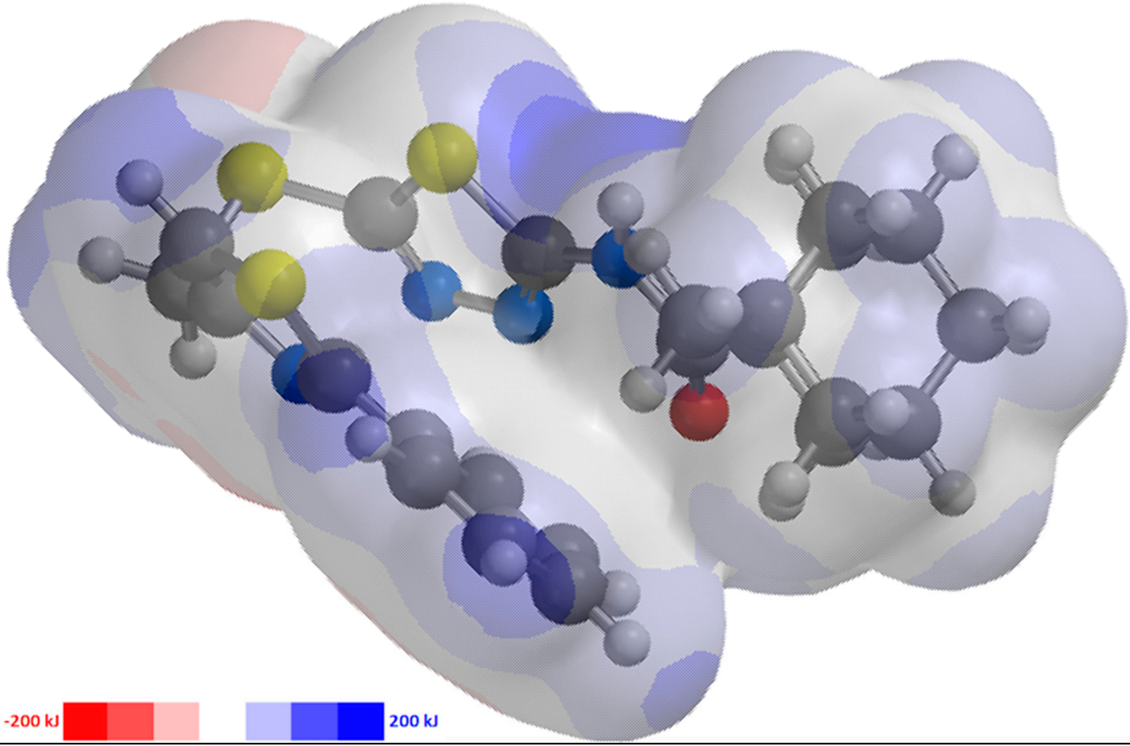

**8i**	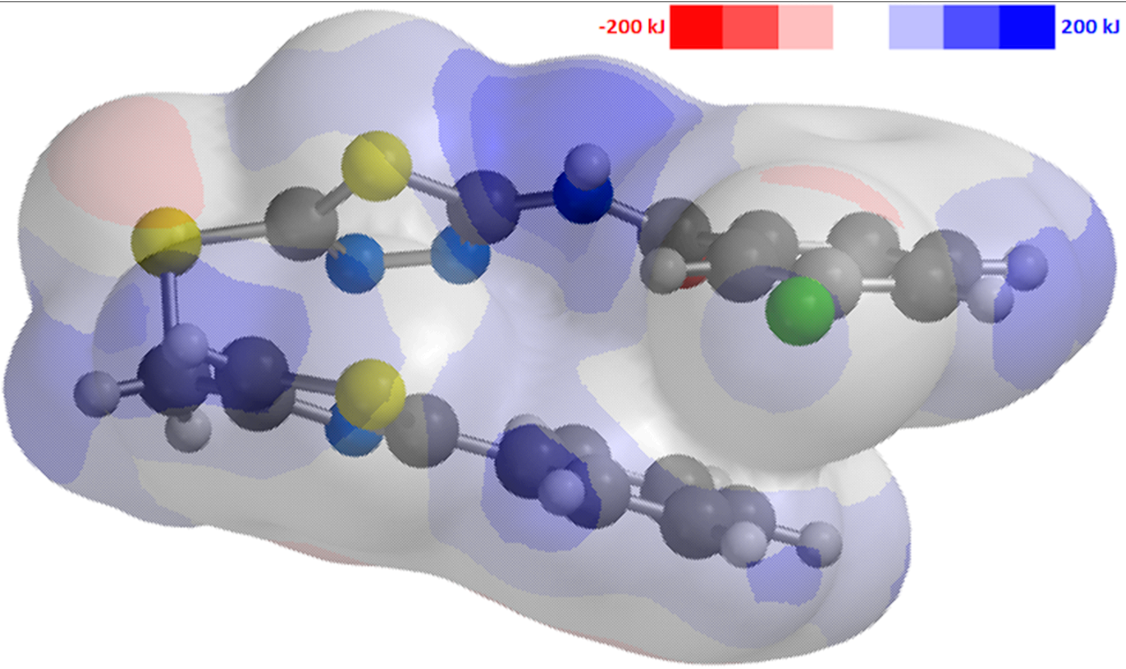
**8j**	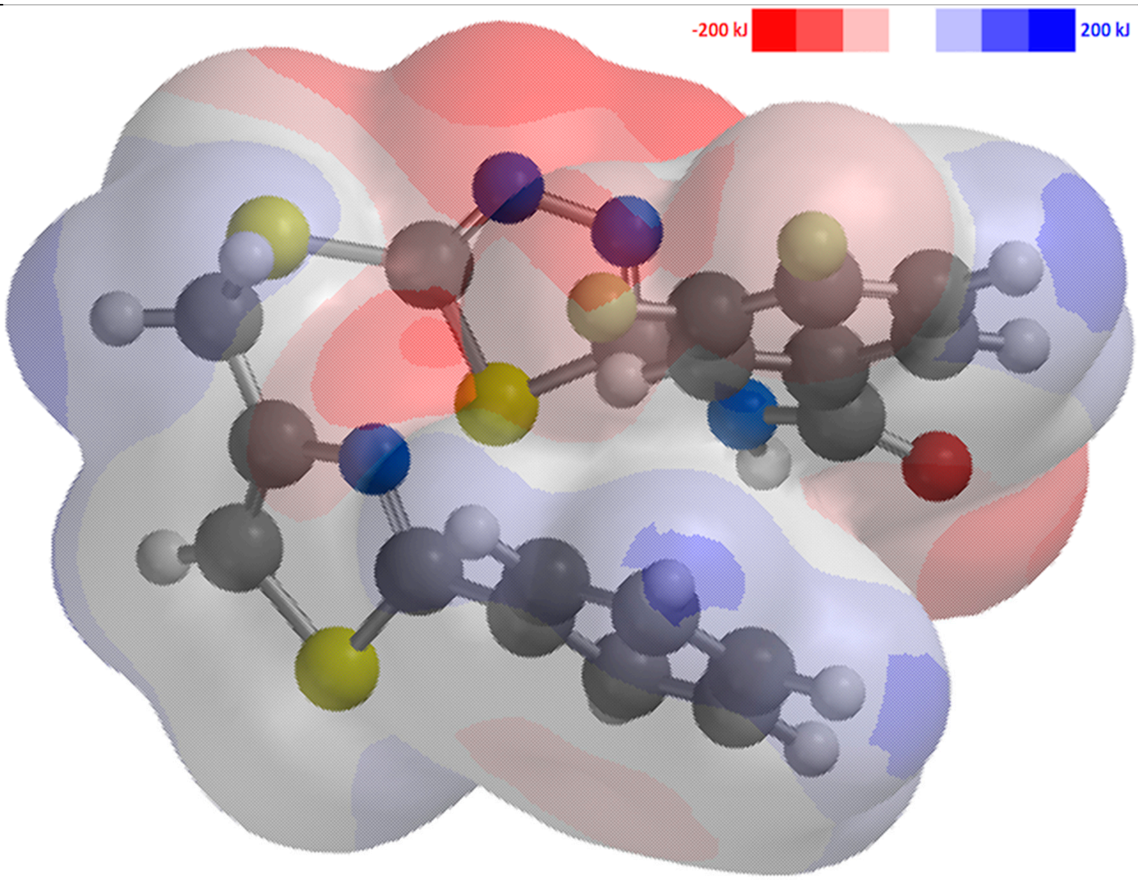

Table S5. The matrix of the thiazolyl-methylthio-thiadiazole compounds.

n	Code	x1	x2	x3	x4	x5	x6	y1	y2	y3	y4	y5	y6	y7	y8	y9	y10	y11	y12
1	x1y1	1	0	0	0	0	0	1	0	0	0	0	0	0	0	0	0	0	0
2	x1y2	1	0	0	0	0	0	0	1	0	0	0	0	0	0	0	0	0	0
3	x2y1	0	1	0	0	0	0	1	0	0	0	0	0	0	0	0	0	0	0
4	x2y2	0	1	0	0	0	0	0	1	0	0	0	0	0	0	0	0	0	0
5	x3y1	0	0	1	0	0	0	1	0	0	0	0	0	0	0	0	0	0	0

6	x3y2	0	0	1	0	0	0	0	1	0	0	0	0	0	0	0	0	0	0
7	x4y1	0	0	0	1	0	0	1	0	0	0	0	0	0	0	0	0	0	0
8	x4y2	0	0	0	1	0	0	0	1	0	0	0	0	0	0	0	0	0	0
9	x5y1	0	0	0	0	1	0	1	0	0	0	0	0	0	0	0	0	0	0
10	x5y2	0	0	0	0	1	0	0	1	0	0	0	0	0	0	0	0	0	0
11	x6y3	0	0	0	0	0	1	0	0	1	0	0	0	0	0	0	0	0	0
12	x6y4	0	0	0	0	0	1	0	0	0	1	0	0	0	0	0	0	0	0
13	x6y5	0	0	0	0	0	1	0	0	0	0	1	0	0	0	0	0	0	0
14	x6y6	0	0	0	0	0	1	0	0	0	0	0	1	0	0	0	0	0	0
15	x6y7	0	0	0	0	0	1	0	0	0	0	0	0	1	0	0	0	0	0
16	x6y8	0	0	0	0	0	1	0	0	0	0	0	0	0	1	0	0	0	0
17	x6y9	0	0	0	0	0	1	0	0	0	0	0	0	0	0	1	0	0	0
18	x6y10	0	0	0	0	0	1	0	0	0	0	0	0	0	0	0	1	0	0
19	x6y11	0	0	0	0	0	1	0	0	0	0	0	0	0	0	0	0	1	0
20	x6y12	0	0	0	0	0	1	0	0	0	0	0	0	0	0	0	0	0	1
